# Supplementary material for: Mechanisms underlying genome instability mediated by formation of foldback inversions in Saccharomyces cerevisiae
Source: eLife. 2020 Aug 7;9:e58223. doi: 10.7554/eLife.58223 (PMC7467729; doi:10.7554/eLife.58223)

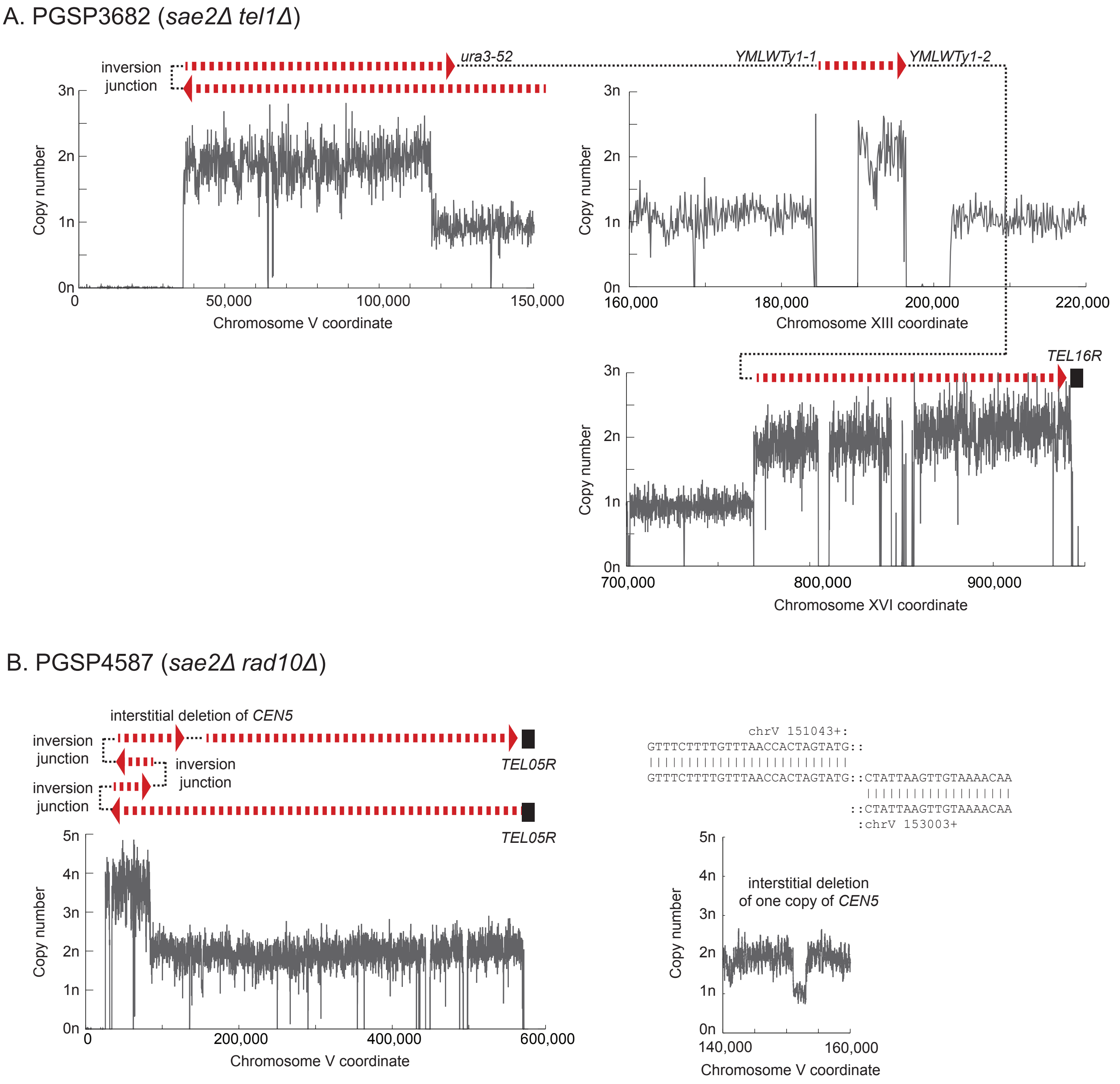

Figure 1 - Source Data 1. Complex GCR structures.

GCR structures that are more complicated than those displayed in Figures 1D and 1E, and Figure 1 - figure supplement 1 are displayed using the same scheme as those displayed in Figure 1 - figure supplement 1. Panels A to U are sorted by the PGSP isolate number. The regions included in the GCR-containing chromosome are displayed using the red dashed arrow (oriented from chrV R to chrV L in the GCR-containing chromosome). The black dotted lines show the connectivity between regions included in the GCR-containing chromosome that are separate in the reference genome. Telomeres added to the chrV L side of the GCR are displayed using black boxes.

Figure 1 - Source Data 1 (page 2 of 8)

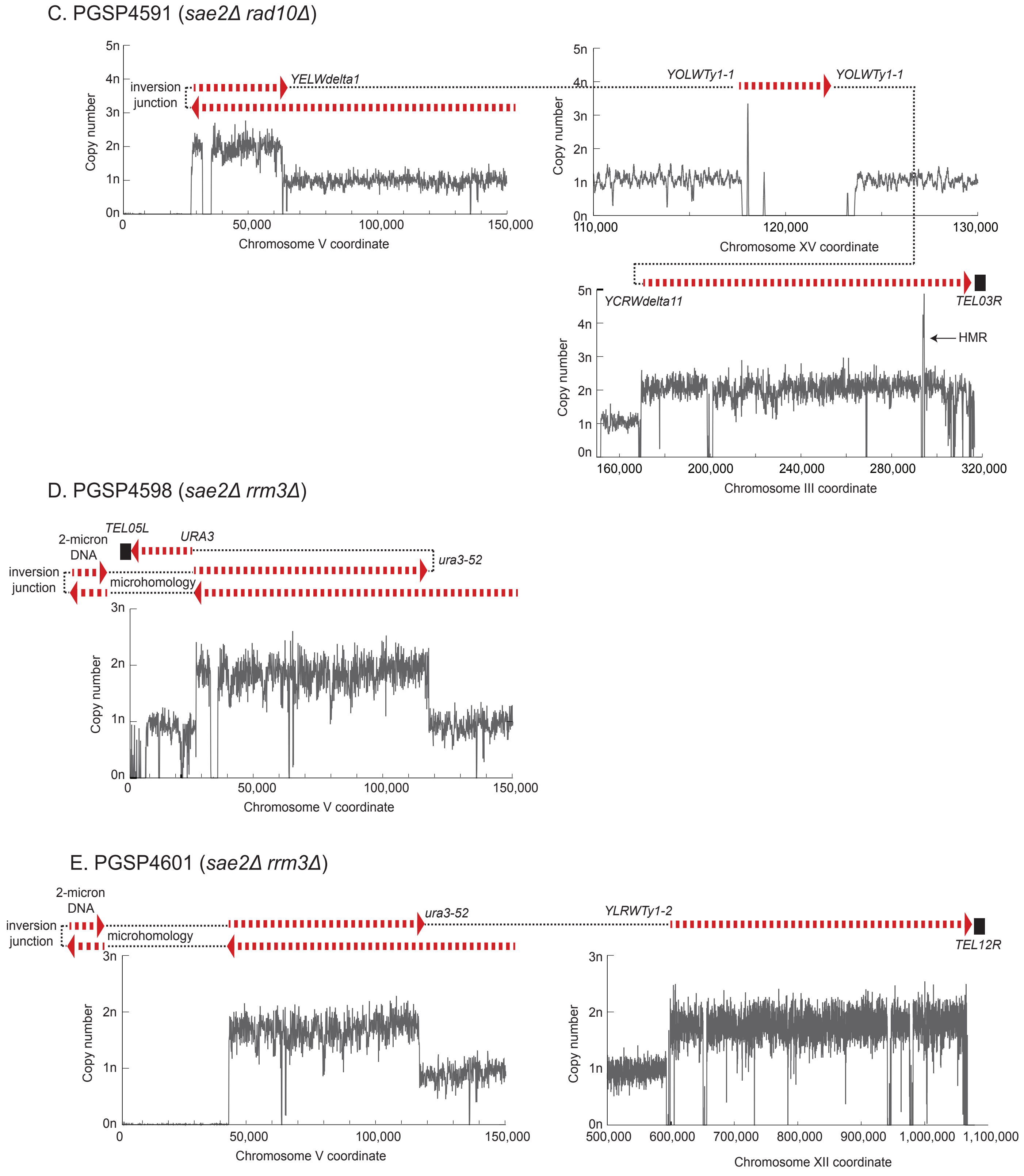

Figure 1 - Source Data 1 (page 3 of 8)

F. PGSP4605 (*sae2Δ rrm3Δ*)

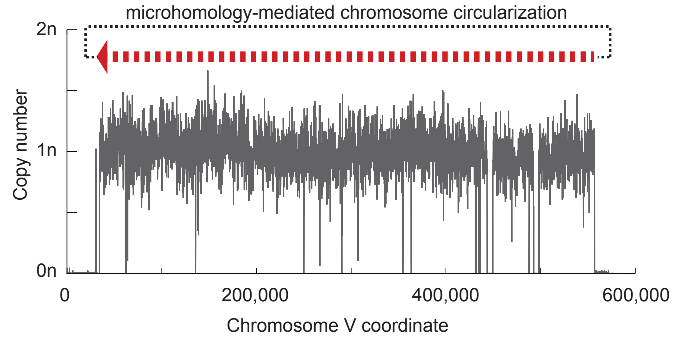


```
          chrV 556943+:
CGAACAACGGAAAATATA:GAA:
||||||||||||||||||  |||
CGAACAACGGAAAATATA:GAA:AAGAAAATAAAACTAGT
                    |||  ||||||||||||||||
                  :GAA:AAGAAAATAAAACTAGT
                  :chrV 30395+
```

G. PGSP4685 (*sae2Δ yku80Δ*)

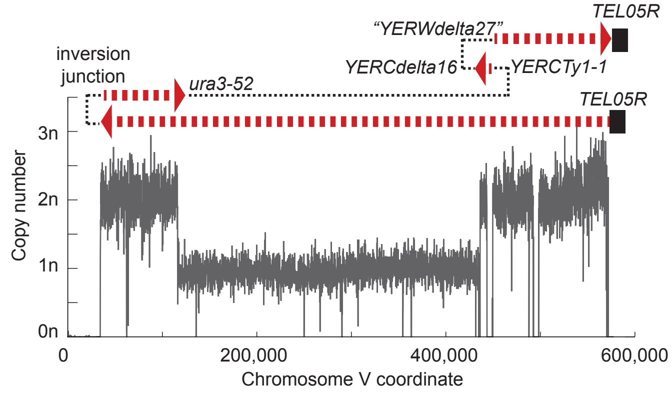

H. PGSP4696 (*sgs1Δ yku80Δ*)

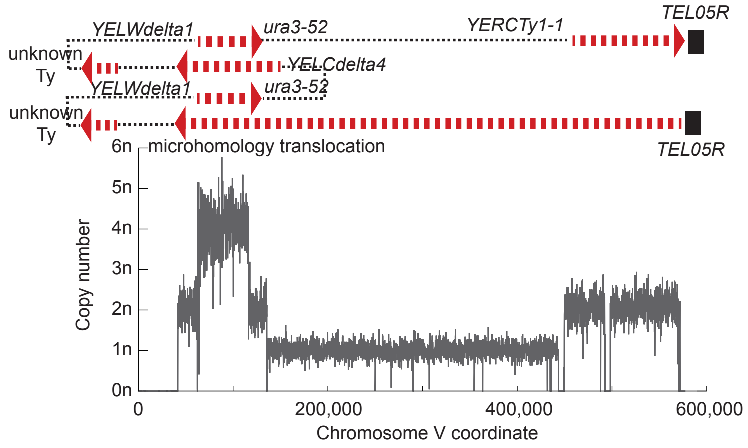

Figure 1 - Source Data 1 (page 4 of 8)

I. PGSP4700 (*sgs1Δ yku80Δ*)

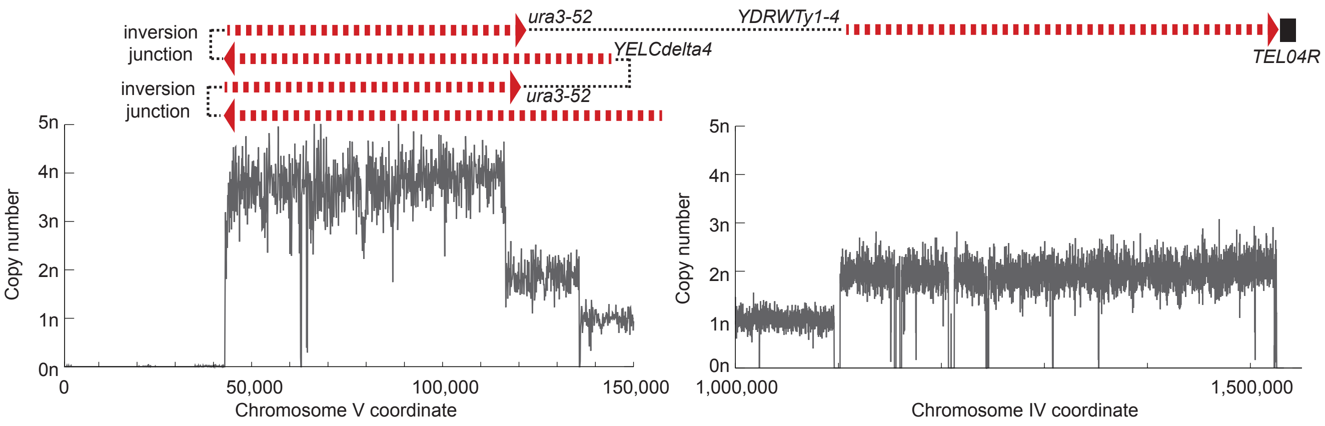

J. PGSP4739 (*sae2Δ sgs1Δ yku80Δ*)

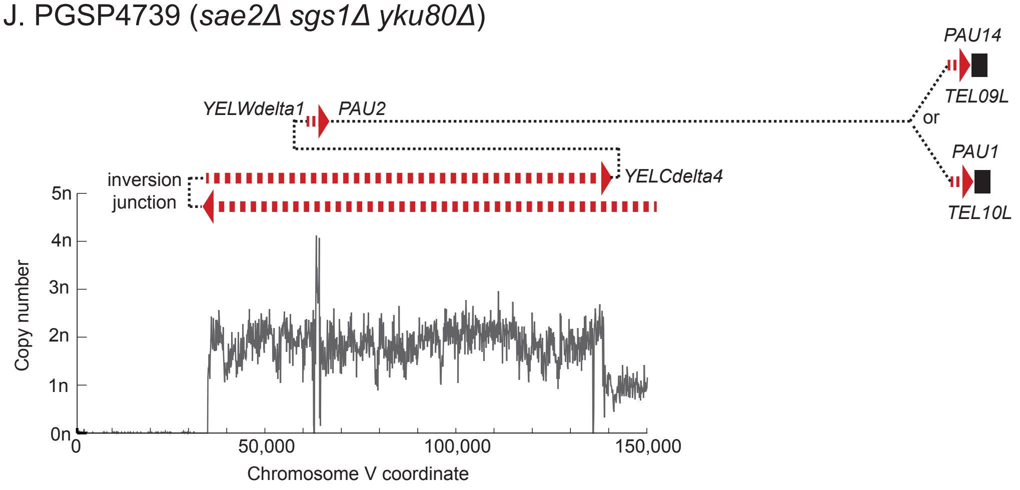

K. PGSP4771 (*sae2-S267A*)

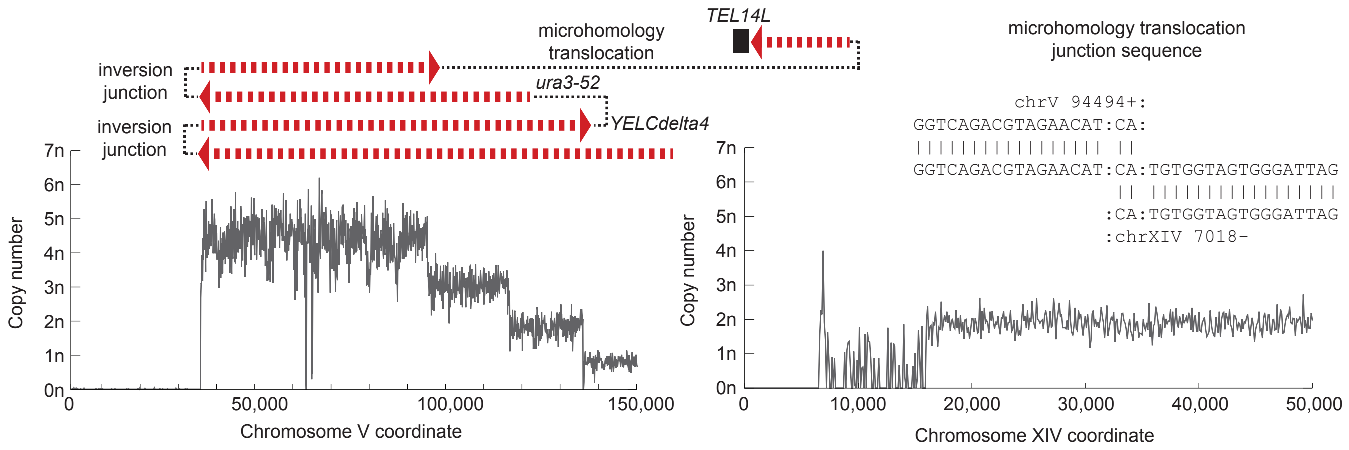

Figure 1 - Source Data 1 (page 5 of 8)

L. PGSP4853 (*sae2Δ yen1Δ*)

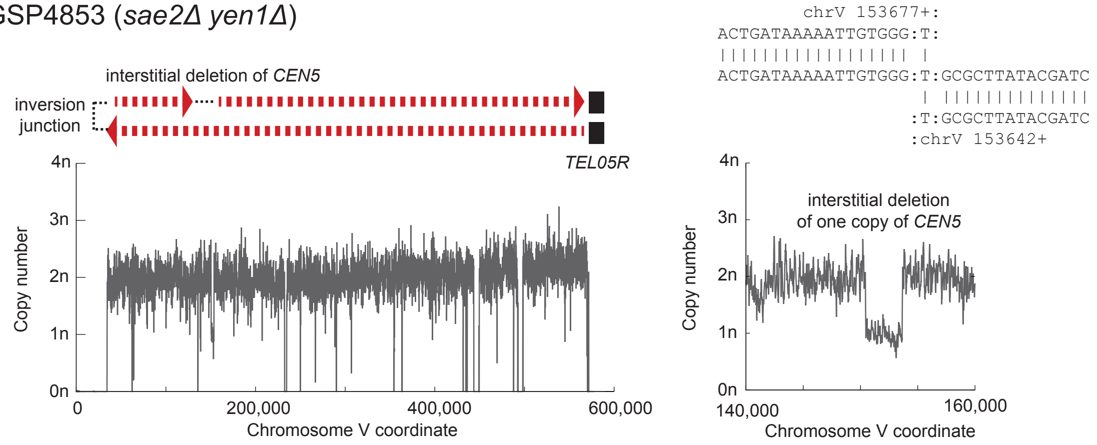

M. PGSP4884 (*sae2Δ rad52Δ*)

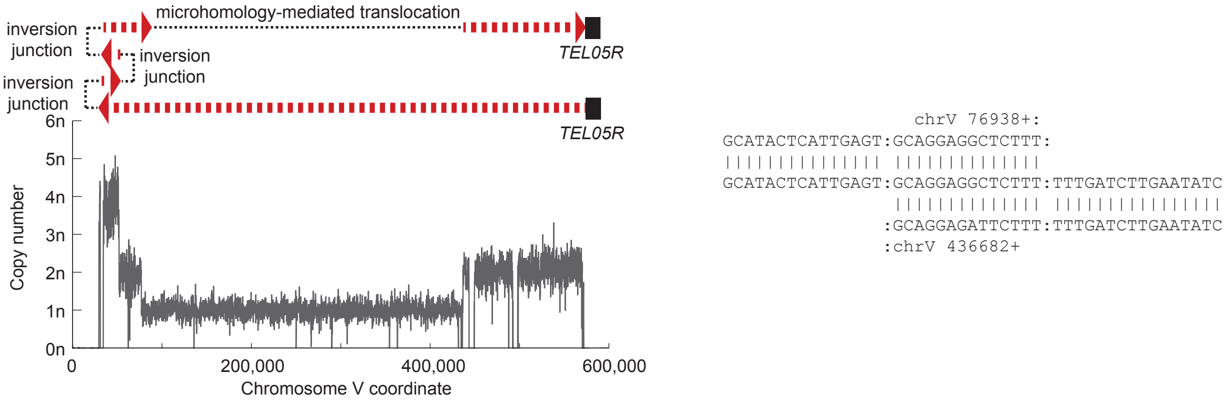

N. PGSP4924 (*sae2Δ sgs1Δ yku80Δ*)

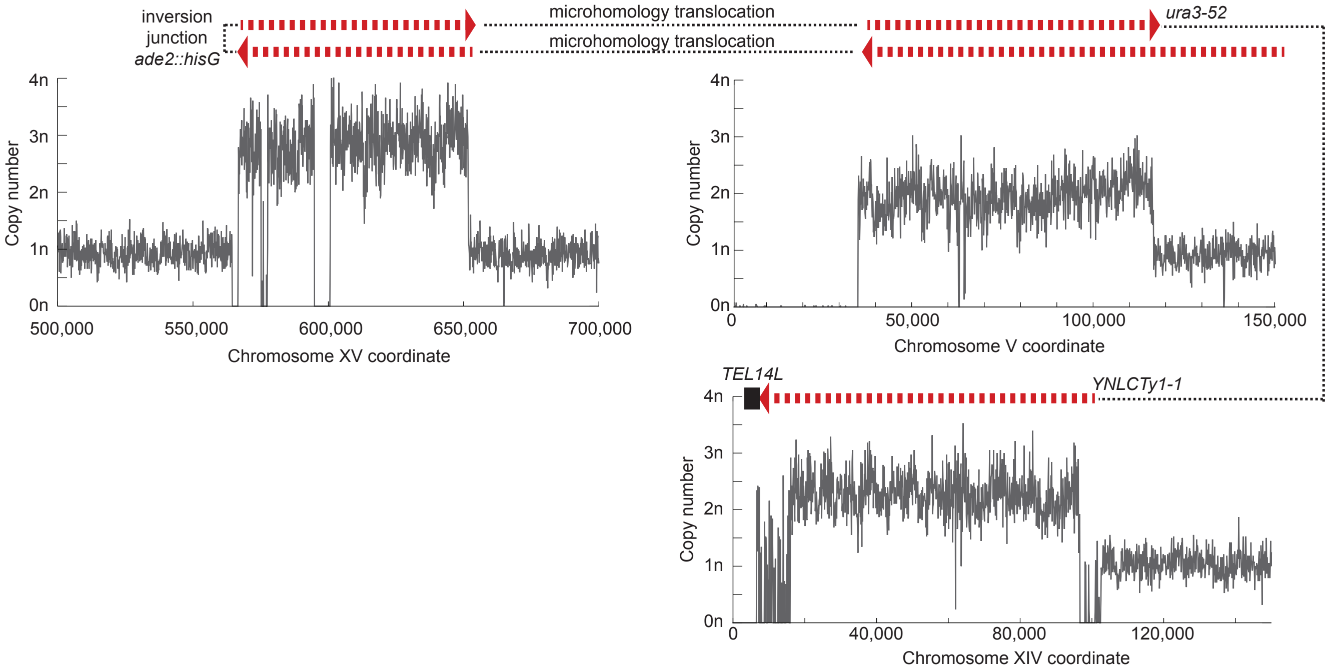

Figure 1 - Source Data 1 (page 6 of 8)

O. PGSP4982 (*sae2Δ chrV:25,817-1,754*)

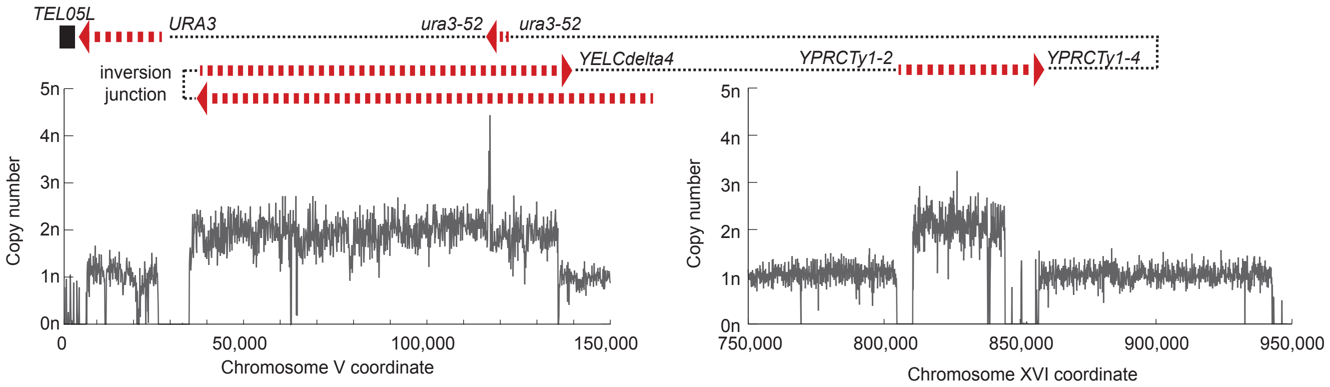

P. PGSP4984 (*sae2Δ chrV:25,817-1,754*)

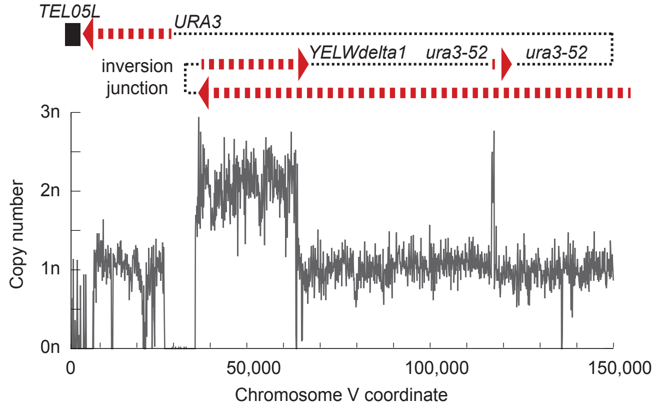

Q. PGSP4995 (*sae2Δ chrV:35,709*)

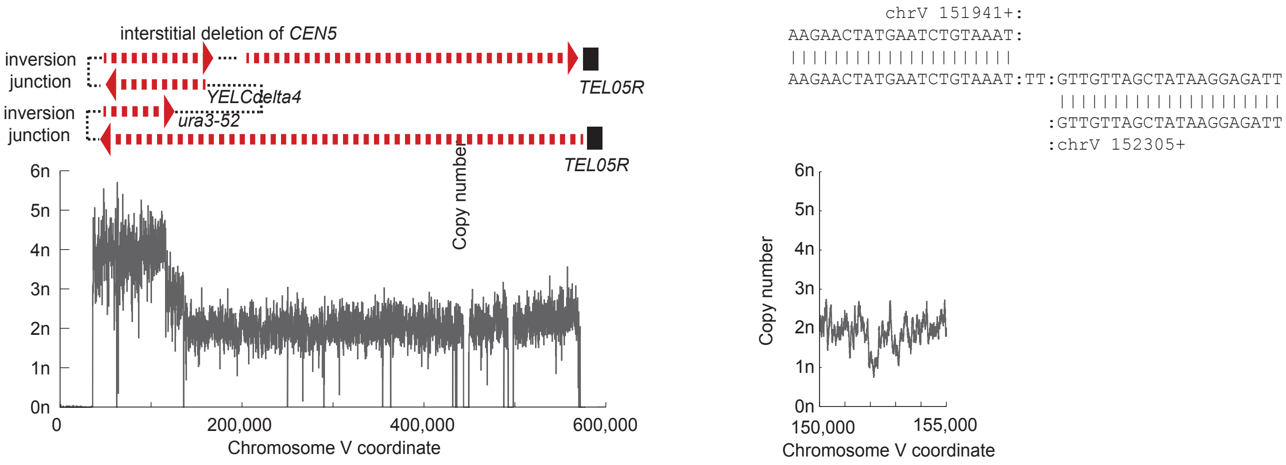

Figure 1 - Sourxe Data 1 (page 7 of 8)

R. PGSP5000 (*sae2Δ hotspotΔ*)

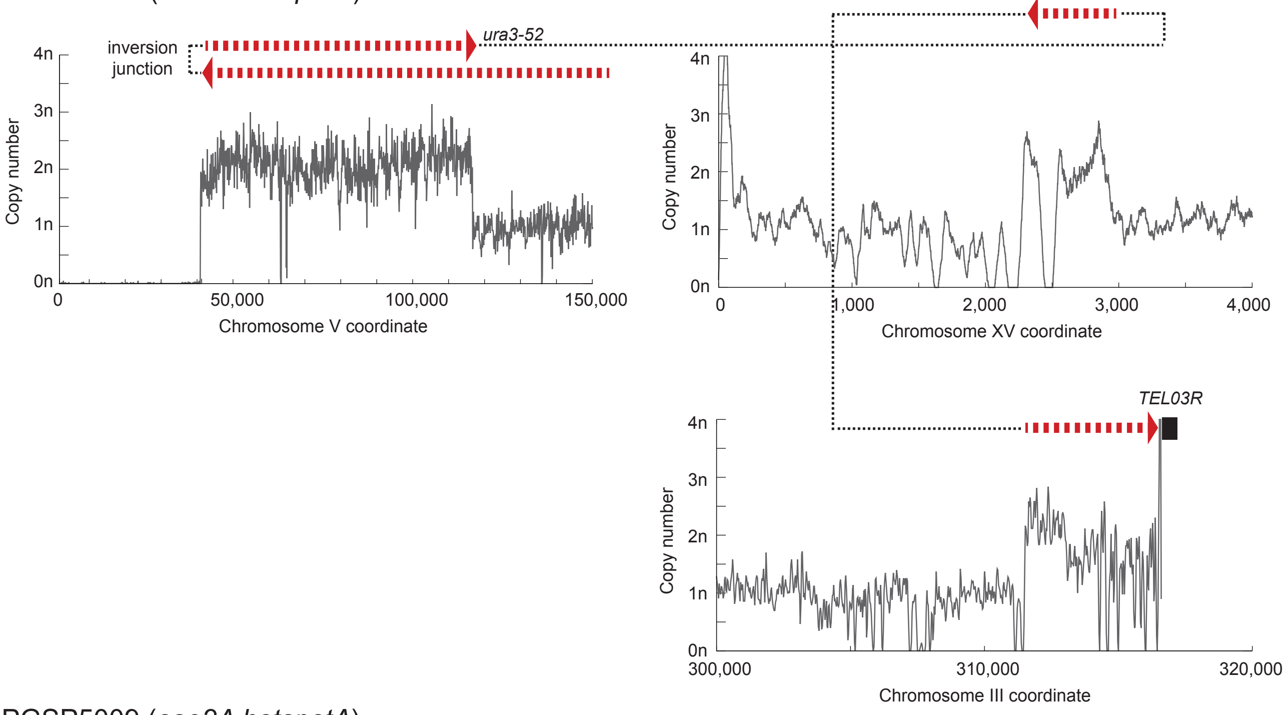

S. PGSP5009 (*sae2Δ hotspotΔ*)

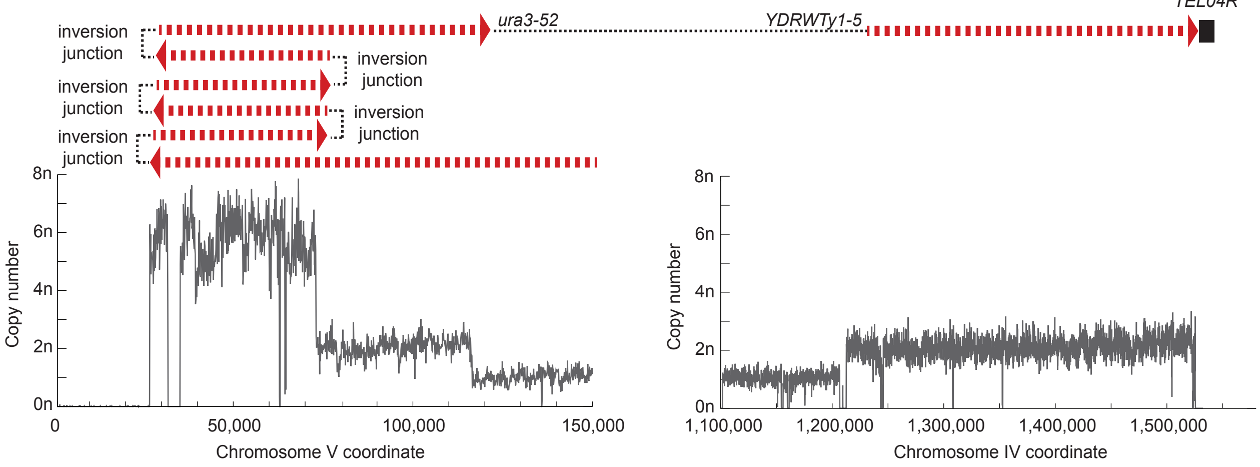

T. PGSP5047 (*sae2Δ exo1Δ yku80Δ*)

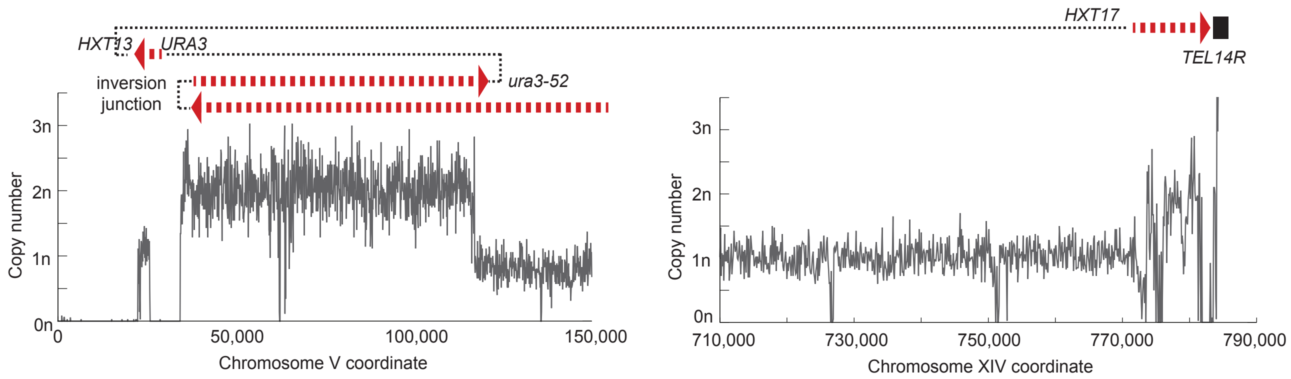

Figure 1 - Source Data 1 (page 8 of 8)

U. PGSP5054 (*sae2Δ exo1Δ yku80Δ*)

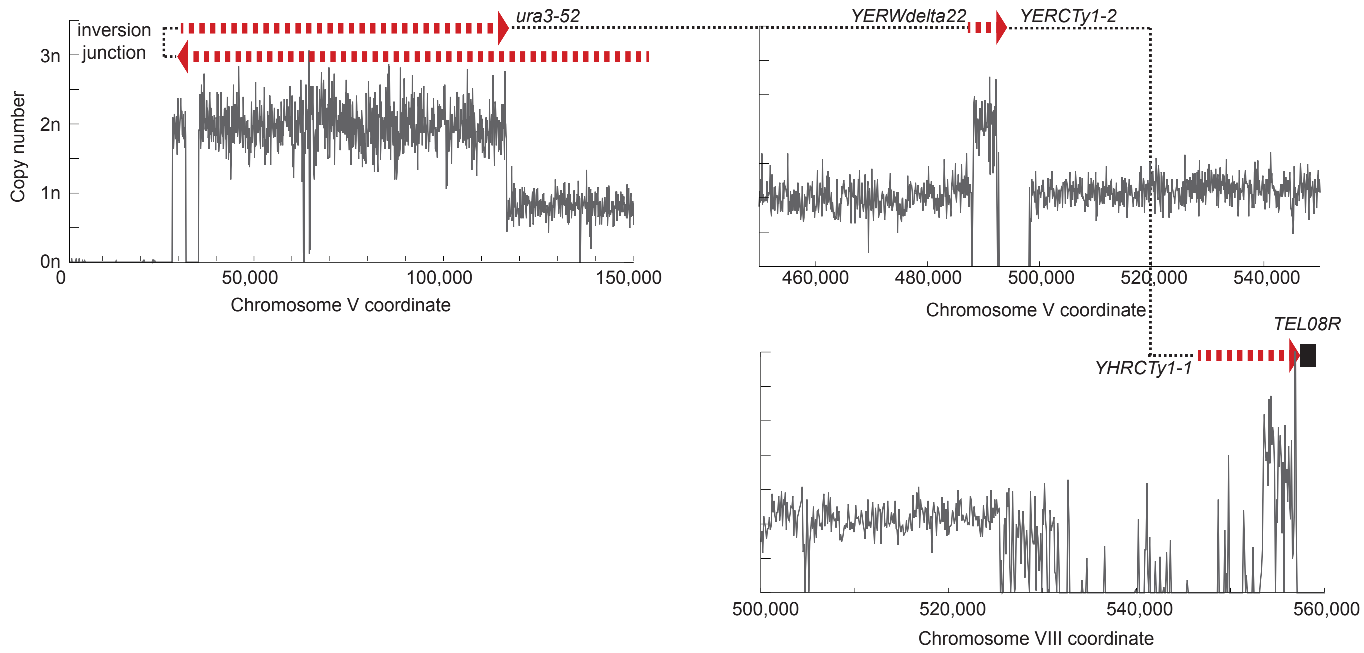

Supplement: Figure 1—source data 1. — GCR structures that are more complicated than those displayed in Figures 1D and 1E, and Figure 1—figure supplement 1 are displayed using the same scheme as those displayed in Figure 1—figure supplement 1. Panels A to U are sorted by the PGSP isolate number. The regions included in the GCR-containing chromosome are displayed using the red dashed arrow (oriented from chrV R to chrV L in the GCR-containing chromosome). The black dotted lines show the connectivity between regions included in the GCR-containing chromosome that are separate in the reference genome. Telomeres added to the chrV L side of the GCR are displayed using black boxes. [file elife-58223-fig1-data1.pdf]
