# Supplementary material for: Mechanisms underlying genome instability mediated by formation of foldback inversions in Saccharomyces cerevisiae
Source: eLife. 2020 Aug 7;9:e58223. doi: 10.7554/eLife.58223 (PMC7467729; doi:10.7554/eLife.58223)

A. chrV L 25,817-2,487_39,967 hairpin

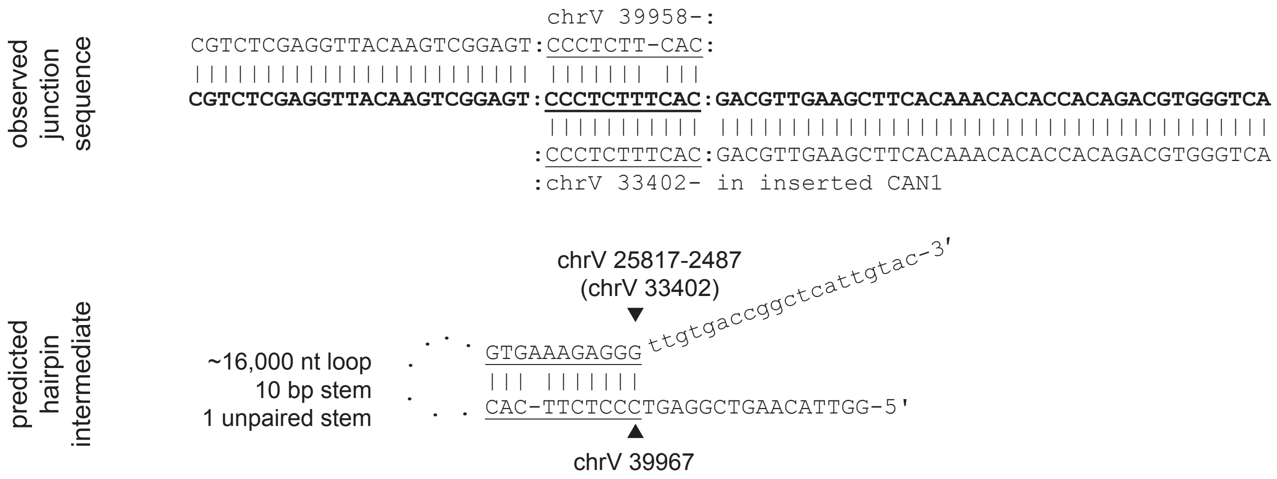

B. chrV L 25,817-1,749_25,817-1,737 hairpin

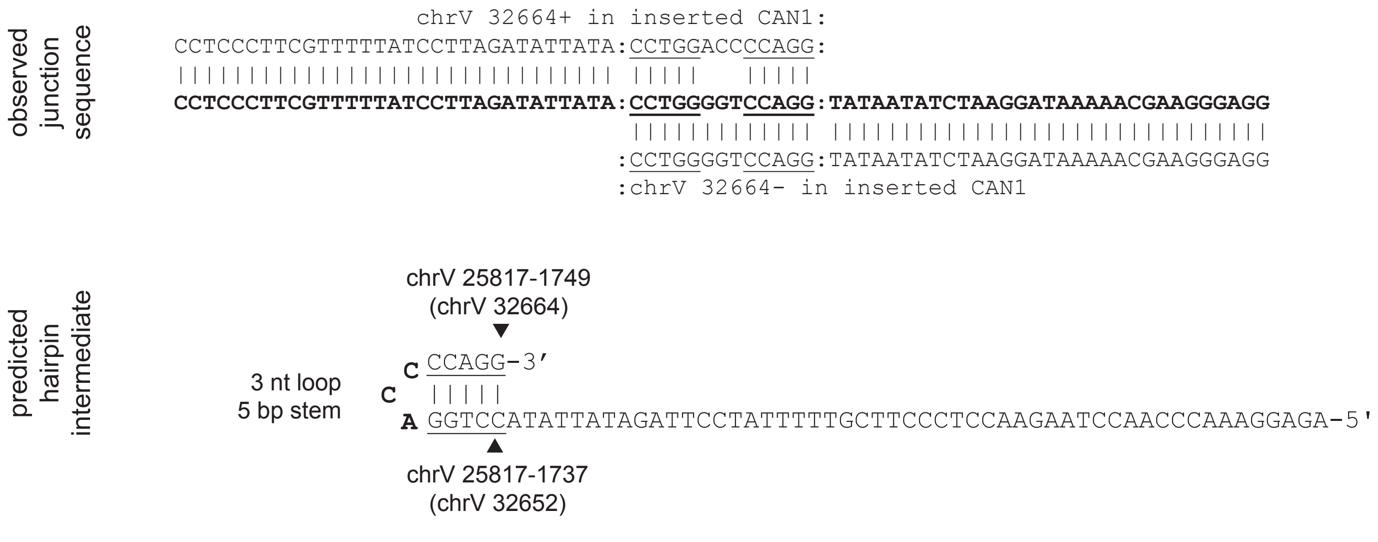

Figure 2 - Source Data 1. Predicted ssDNA hairpin structures for selected observed foldback inversions.

The structures of the key predicted ssDNA hairpin intermediates observed (panels A to BK are sorted by the chromosomal position of the predicted hairpin). The chrV coordinates for each predicted hairpin correspond to the coordinates reported for the foldback inversion-containing GCRs.

C. chrV L 25,817-1,731_25,817_-1,720 hairpin

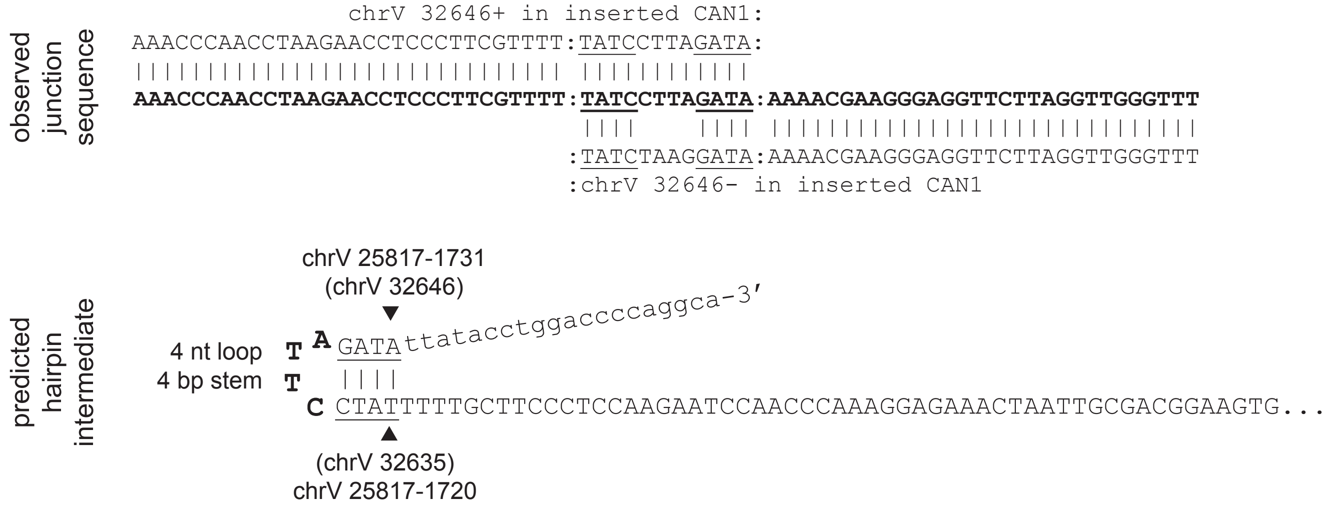

D. chrV L 25,817-1,529_25,817-1,505 hairpin

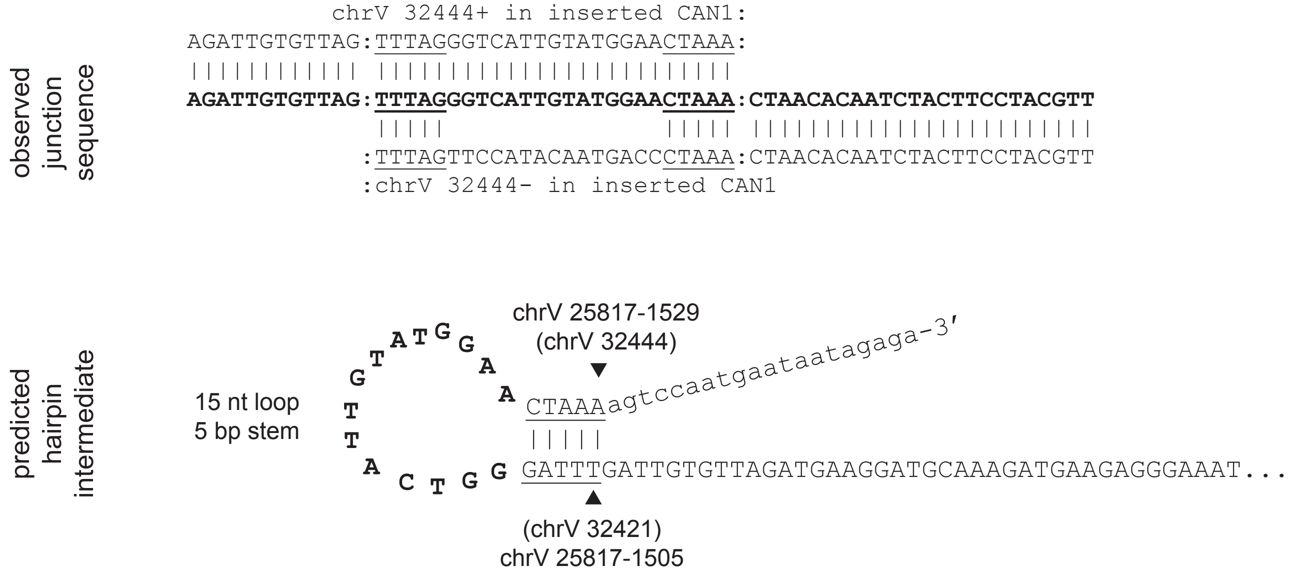

E. chrV L 25,817-1,303_25,817-1,289 hairpin

observed junction sequence

```
               chrV 32218+ in inserted CAN1:
GTAACGAAAACTGCAATGTATGGAAC:ACCACCTTTGGTGGT:
||||||||||||||||||||||||||  |||||||||||||||
GTAACGAAAACTGCAATGTATGGAAC:ACCACCTTTGGTGGT:GTTCCATACATTGCAGTTTTCGTTACTGCTGCATTTGGC
                            ||||||   |||||| ||||||||||||||||||||||||||||||||||||||||
                          :ACCACCAAAGGTGGT:GTTCCATACATTGCAGTTTTCGTTACTGCTGCATTTGGC
                          :chrV 33218- in inserted CAN1
```

predicted hairpin intermediate

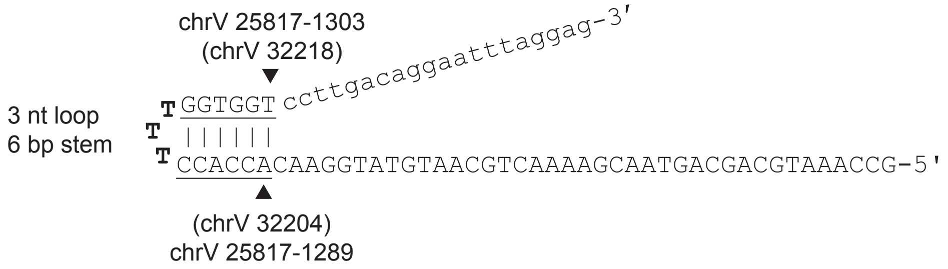

F. chrV L 25,817-592_25,817-570 hairpin

observed junction sequence

```
    chrV 31507+ in CAN1:
C:TTGAAGGTCTGAAGGAGTTTCAA:
| |||||||||||||||||||||||
C:TTGAAGGTCTGAAGGAGTTTCAA:GTATATATATATATATATATATATGTATATGTGTACATTTTCACG
  |||||             ||||| ||||||||||||||||||||||||||||||||||||||||||||||
 :TTGAAACTCCTTCAGACCTTCAA:GTATATATATATATATATATATATGTATATGTGTACATTTTCACG
 :chrV 31507- in inserted CAN1
```

predicted hairpin intermediate

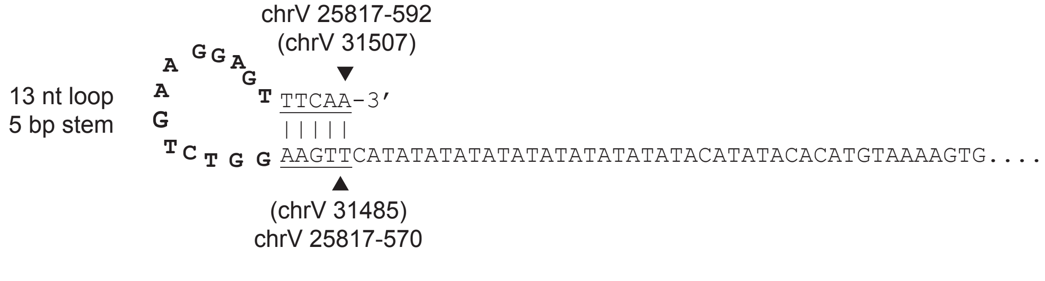

G. chrV L 25,817-568_25,817-546 hairpin

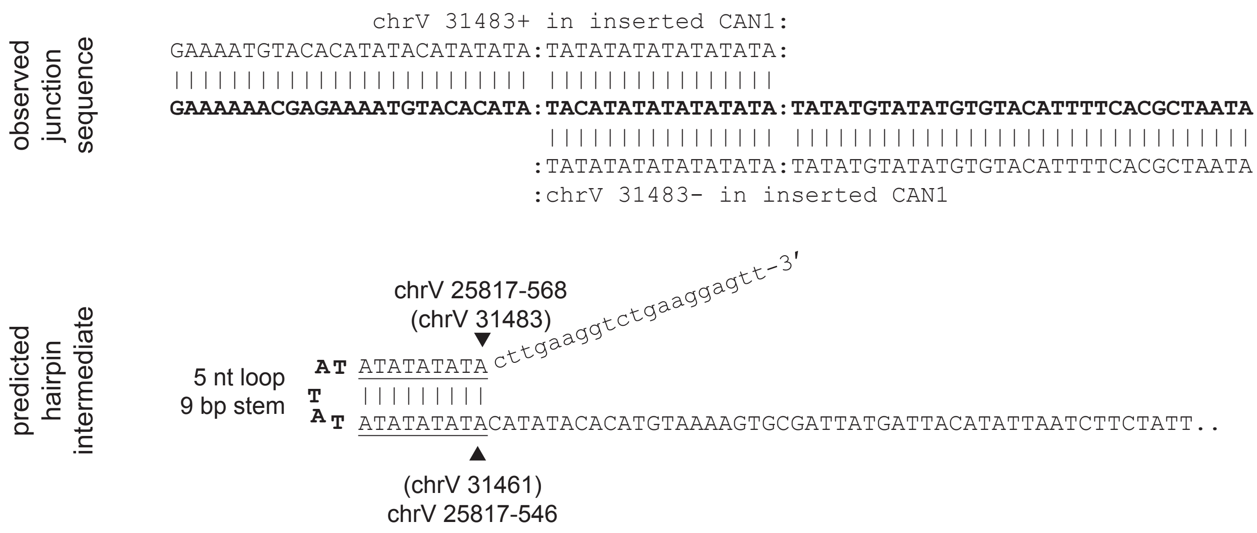

H. chrV L 25,817-43_25,817-20 hairpin

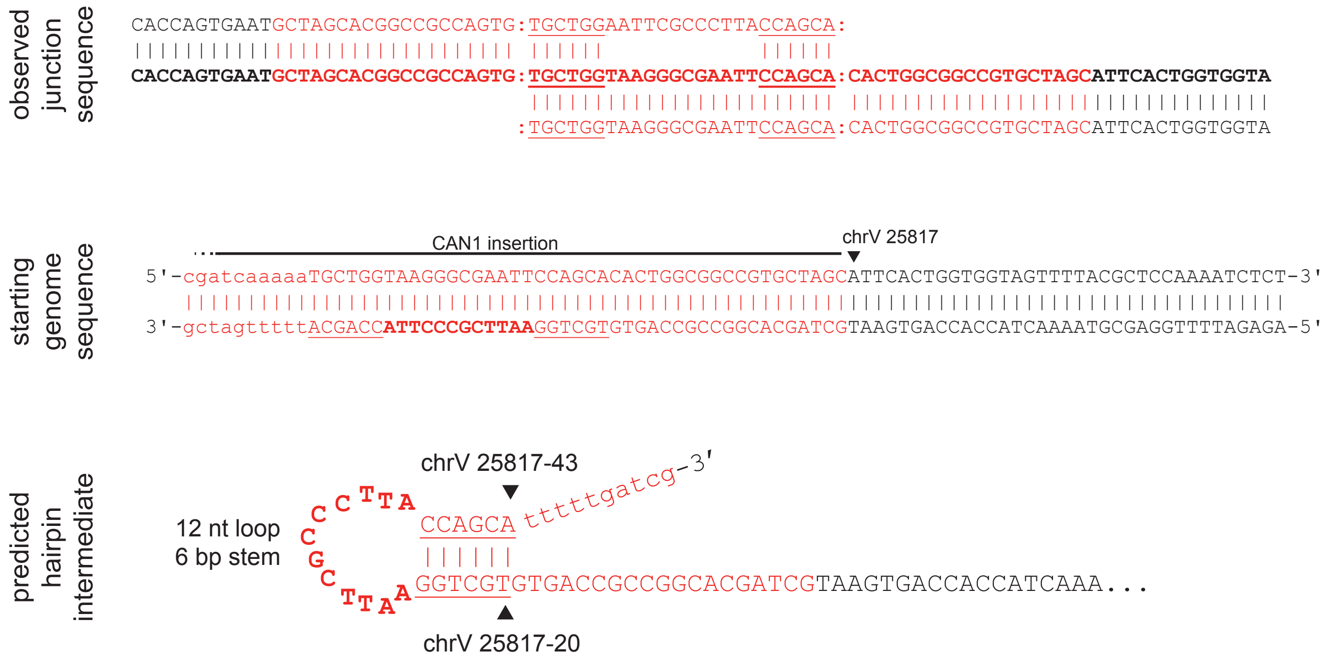

I. chrV L 25,817-30_25,820 hairpin

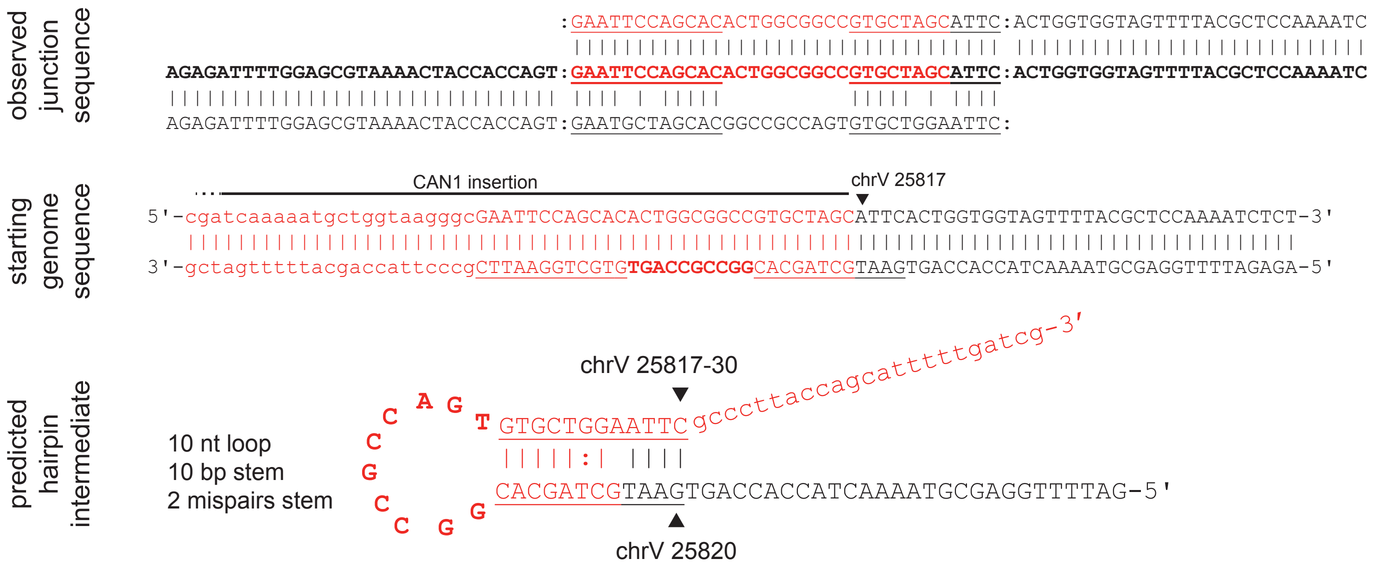

J. chrV L 25,817-25_25,817-14 hairpin

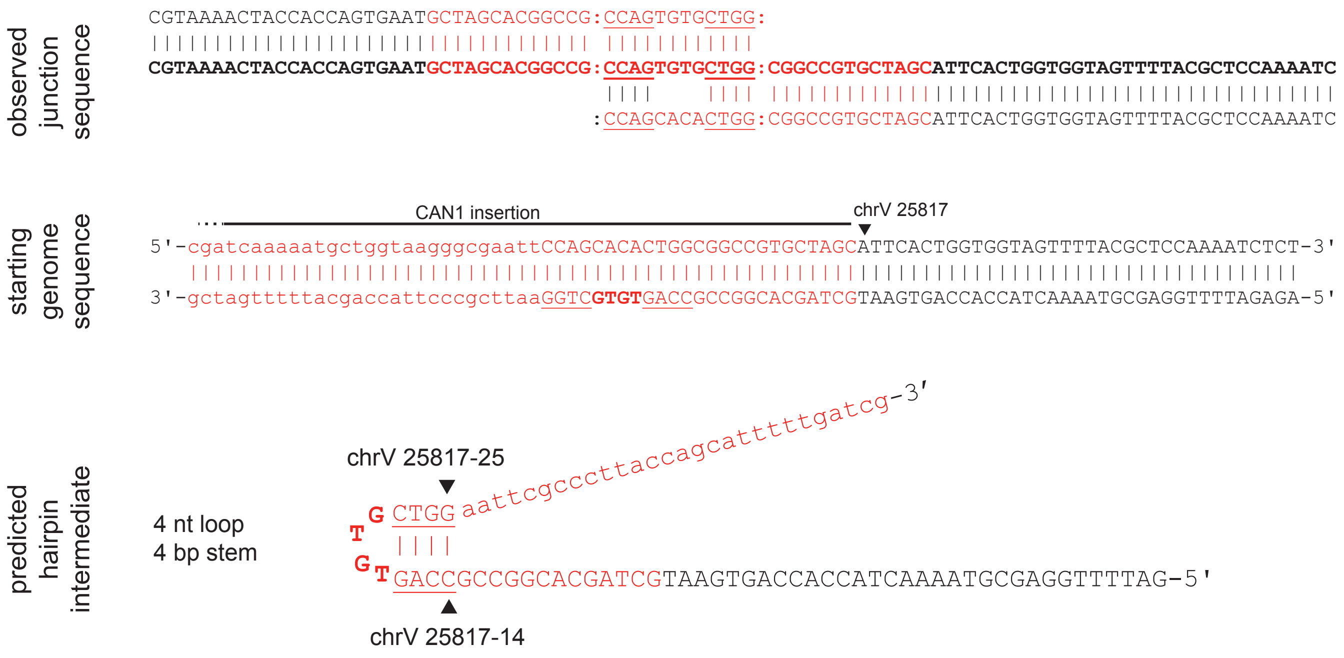

K. chrV L 25,965_26,002 hairpin

observed junction sequence

```
                                        chrV 25965-:
ATAAA:GCCAAAATCAGGTATACTGAAAGGATGAAAATTTTGGC:
||||| ||||||||||||||||||||||||||||||||||||||
ATAAA:GCCAAAATCAGGTATACTGAAAGGATGAAAATTTTGGC:TTTATCCTTTCAATGTGCCTGATTTTGGCTTTCTCGTA
      ||||||||                      |||||||| ||||||||||||||||||||||||||||||||||||||
     :GCCAAAATTTTCATCCTTTCAGTATACCTGATTTTGGC:TTTATCCTTTCAATGTGCCTGATTTTGGCTTTCTCGTA
     :chrV 25965+
```

predicted hairpin intermediate

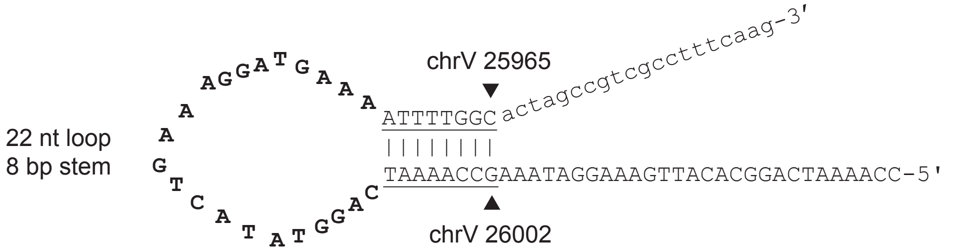

L. chrV L 26,457_26,474 hairpin

observed junction sequence

```
                                          chrV 26457-:
ATGCAATGTACCGCCTGATCATCAGCTGATCGCTTCAGT:GCGATC:
||||||||||||||||||||||||||||||||||||||| ||||||
ATGCAATGTACCGCCTGATCATCAGCTGATCGCTTCAGT:GCGATC:AGCTGATGATCAGGCGGTACATTGCATACTCTGC
                                        |||||| ||||||||||||||||||||||||||||||||||
                                       :GCGATC:AGCTGATGATCAGGCGGTACATTGCATACTCTGC
                                       :chrV 26469+
```

predicted hairpin intermediate

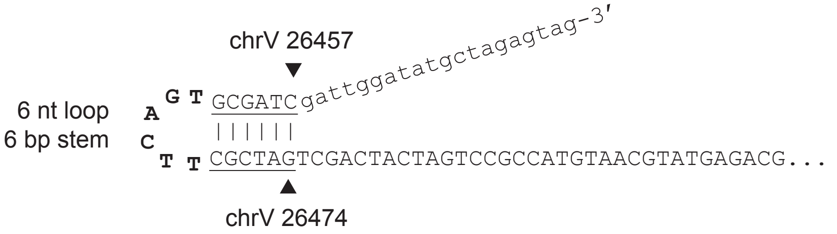

M. chrV L 26,466_26,538 hairpin

observed junction sequence

```
                                 chrV 26466-:
CAGAGTATGCAATGTACCGCCTGATCATCAGC:TGATCGCTTC:
|||||||||||||||||||||||||||||||| ||||||||||
CAGAGTATGCAATGTACCGCCTGATCATCAGC:TGATCGCTTC:GTATATTTCCCAATCCGGTTATTAAATCGTT
                                 ||| ||| ||| |||||||||||||||||||||||||||||||
                                :TGACCGCATC:GTATATTTCCCAATCCGGTTATTAAATCGTT
                                :chrV 26529+
```

predicted hairpin intermediate

```
                          chrV 26466
                               ▼
52 nt loop          . . . TGATCGCTTC-3’
8 bp stem          .      ||| ||| ||
2 mispairs stem     . . . ACTGGCGTAGCATATAAAGGGTTA-5'
                               ▲
                          chrV 26538
```

N. chrV L 26,572_28,017 hairpin

observed junction sequence

```
                                       chrV 26572-:
TCTTGTCGCAACACTCTCCAGTATACTGTTCGCGAAGGT:TTCCGCCTG:
||||||||||||||||||||||||||||||||||||||| || || |||
TCTTGTCGCAACACTCTCCAGTATACTGTTCGCGAAGGT:TTGCGGCTG:TAGGCCAGATCTTCTTTGCCAGATTATCAGATA
                                        ||||||||| ||||||||||||||||||||||||||||||||||
                                       :TTGCGGCTG:TAGGCCAGATCTTCTTTGCCAGATTATCAGATA
                                       :chrV 28009+
```

predicted hairpin intermediate

```
                          chrV 26572
                              ▼
1427 nt loop        . . . TTCCGCCTG-3’
7 bp stem          .      || || |||
2 mispairs stem     . . . AACGCCGACATCCGGTCTAGAAGAAACGGTCTAATAGTCTAT-5'
                              ▲
                          chrV 28017
```

O. chrV L 27,074_27,102 hairpin

observed junction sequence

```
                                       chrV 27074-:
AGTACACTGCAGGCGTACCGAGCTCA:TCGGCACGGCTG:
|||||||||||||||||||||||||| |||||||| |||
AGTACACTGCAGGCGTACCGAGCTCA:TCGGCACGCCTG:CAGTGTACTGGGTTACAGACCATTA
                           |||||||||||| |||||||||||||||||||||||||
                          :TCGGTACGCCTG:CAGTGTACTGGGTTACAGACCATTA
                          :chrV 27091+
```

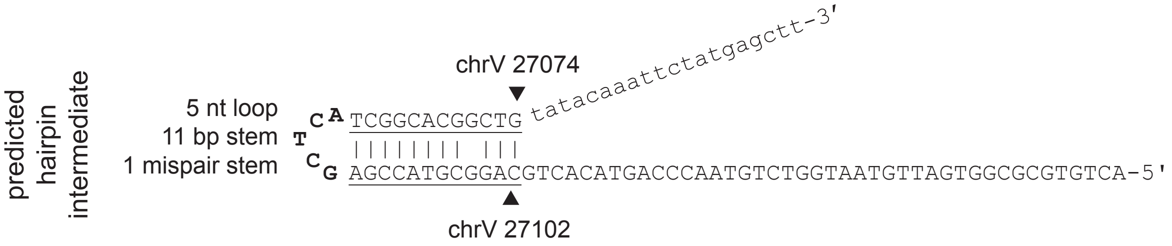

P. chrV L 27,348_27,361 hairpin

observed junction sequence

```
                      chrV 27348-:
GCTGCAAGTCATTCTGTGCCT:GCGCCGTACGGCGC:
 |||||||||||||||||||| ||||||||||||||
ACTGCAAGTCATTCTGTGCCT:GCGCCGTACGGCGC:AGGCACAGAATGACTTGCAGCTTAA
                      |||||||||||||| |||||||||||||||||||||||||
                     :GCGCCGTACGGCGC:AGGCACAGAATGACTTGCAGCTTAA
                     :chrV 27348+
```

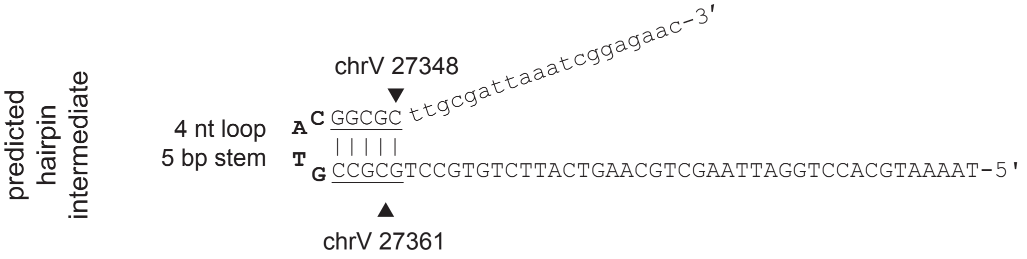

Q. chrV L 27,569_27,583 hairpin

observed junction sequence

```
                                               chrV 27,569-:
AAAGCACTACGATGTTCTAGCCTCATCGGTG:ATGGACGGTGTCCAT:
||||||||||||||||||||||||||||||| ||||||   ||||||
AAAGCACTACGATGTTCTAGCCTCATCGGTG:ATGGACACCGTCCAT:CACCGATGAGGCTAGAACATCGTAGT
                                ||||||||||||||| ||||||||||||||||||||||||||
                               :ATGGACACCGTCCAT:CACCGATGAGGCTAGAACATCGTAGT
                               :chrV 27,569+
```

predicted hairpin intermediate

3 nt loop
6 bp stem

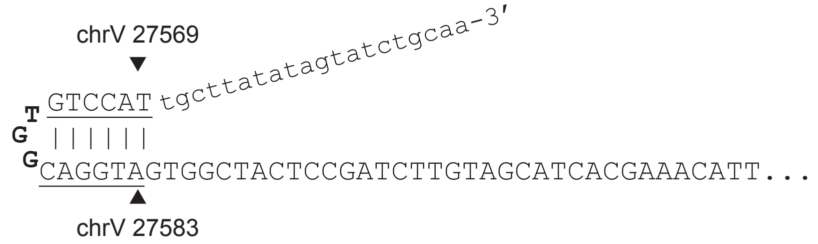

R. chrV L 28,829_28,849 hairpin

observed junction sequence

```
                                        chrV 28829-:
AACGATAACAGAAACAAATGAATACAAGGAGGTAATT:CTTGTA:
||||||||||||||||||||||||||||||||||||| ||||||
AACGATAACAGAAACAAATGAATACAAGGAGGTAATT:CTTGTA:TTCATTTGTTTCTGTTATCGTTGGTACAA
                                      |||||| |||||||||||||||||||||||||||||
                                     :CTTGTA:TTCATTTGTTTCTGTTATCGTTGGTACAA
                                     :chrV 28844+
```

predicted hairpin intermediate

5 nt loop
7 bp stem
1 mispair stem

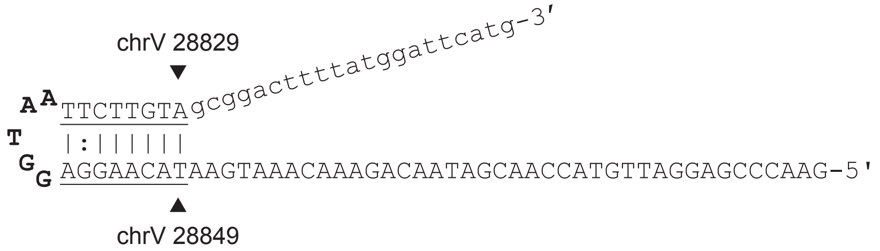

S. chrV L 29,068_29,103 hairpin

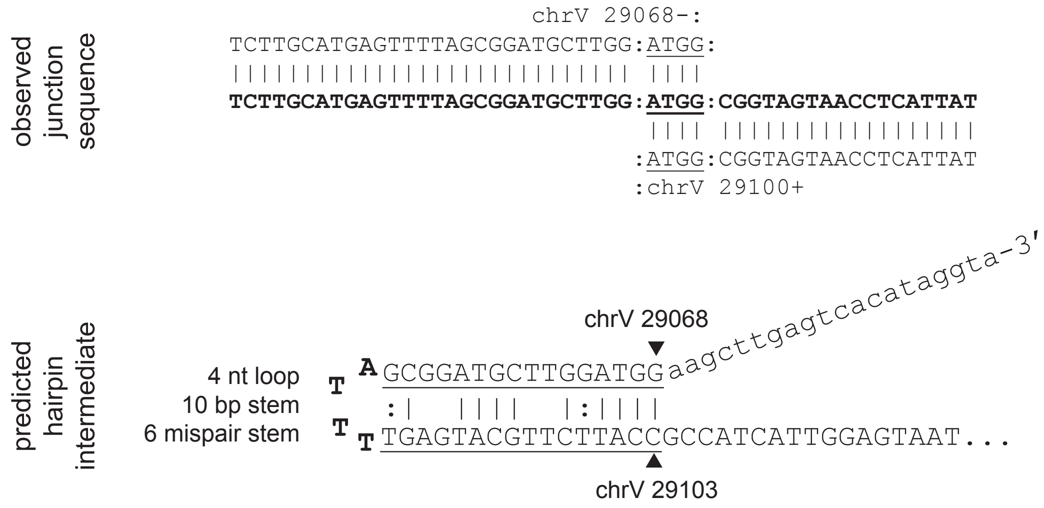

T. chrV L 29,418_29,435 hairpin

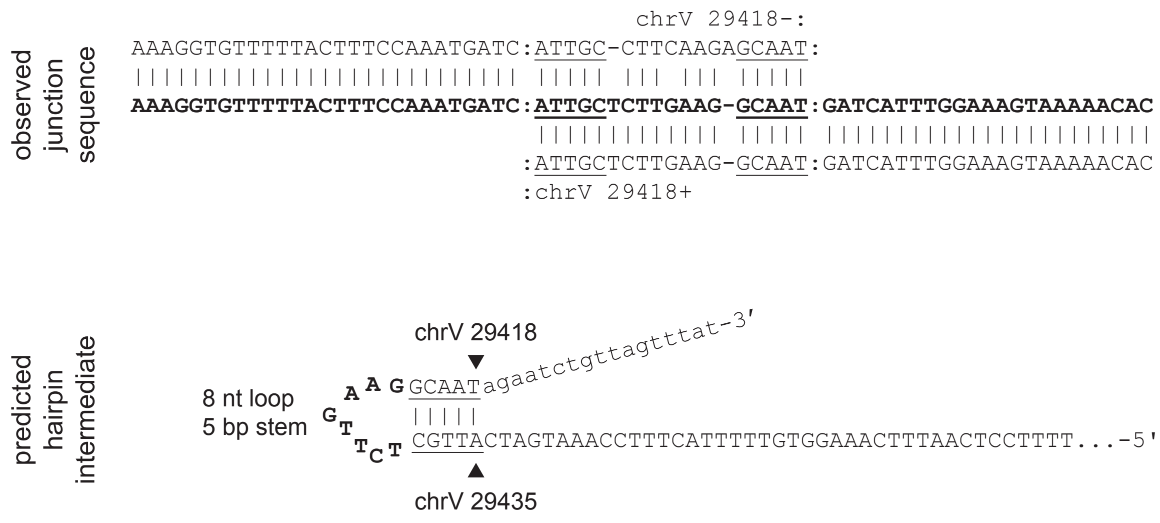

Figure 2 - Source Data 1 (page 11 of 32)

U. chrV L 29,503_29,539 hairpin

observed junction sequence

```
                                 chrV 29535-:
ATGTATGAAATTATTTGGGTGAGATAATATTAA:TCTTT:
||||||||||||||||||||||||||||||||| |||||
ATGTATGAAATTATTTGGGTGAGATAATATTAA:TCTTT:ACGCACTTTACAAGCAGTAAAGATAGGAAAGA
                                  ||||| ||||||||||||||||||||||||||||||||
                                 :TCTTT:ACGCACTTTACAAGCAGTAAAGATAGGAAAGA
                                 :chrV 29503+
```

predicted hairpin intermediate

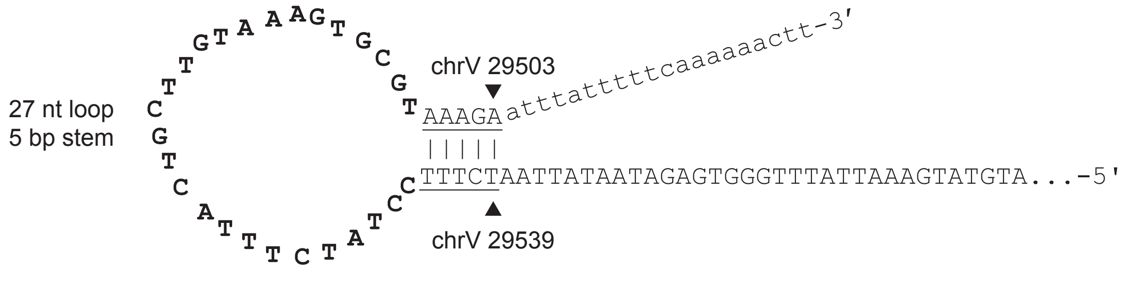

V. chrV L 29,513_29,529 hairpin

observed junction sequence

```
                           chrV 29513-:
TATTAATCTTTCCTATCTTTACTGCTT:GTAAAG:
||||||||||||||||||||||||||| ||||||
TATTAATCTTTCCTATCTTTACTGCTT:GTAAAG:ATAGGAAAGATTAATATTATCT
                            |||||| ||||||||||||||||||||||
                           :GTAAAG:ATAGGAAAGATTAATATTATCT
                           :chrV 29524+
```

predicted hairpin intermediate

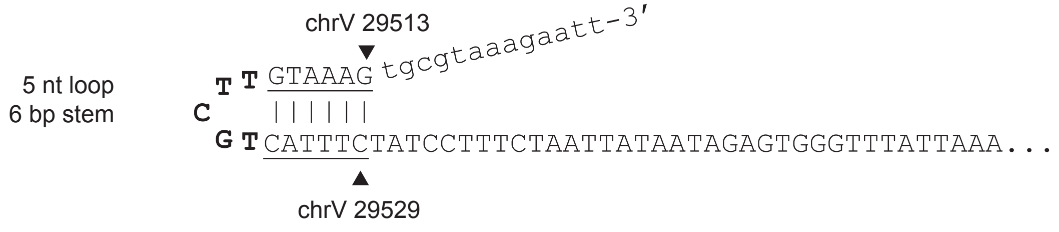

W. chrV L 30,830_30,856 hairpin

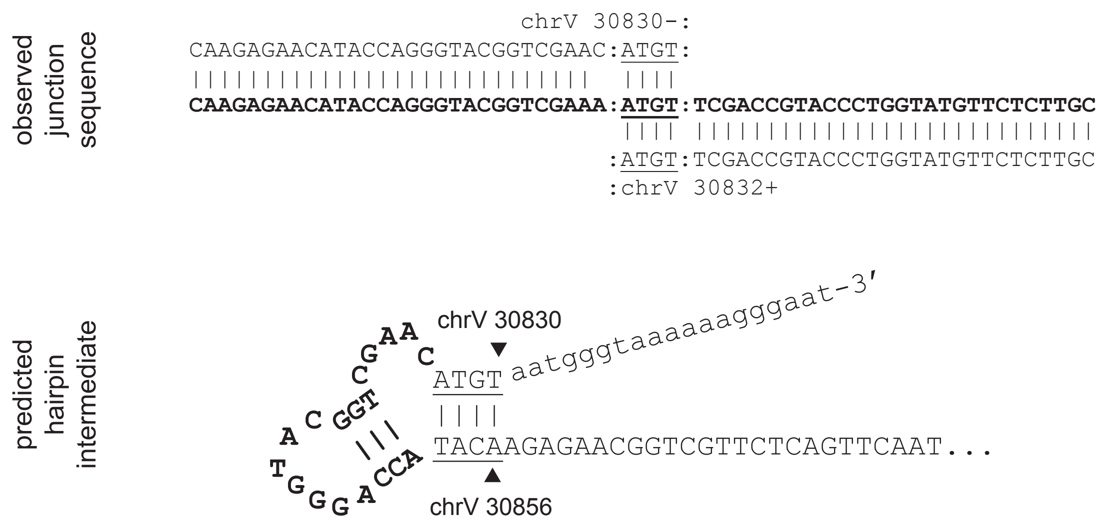

X. chrV L 30,857_30,872 hairpin

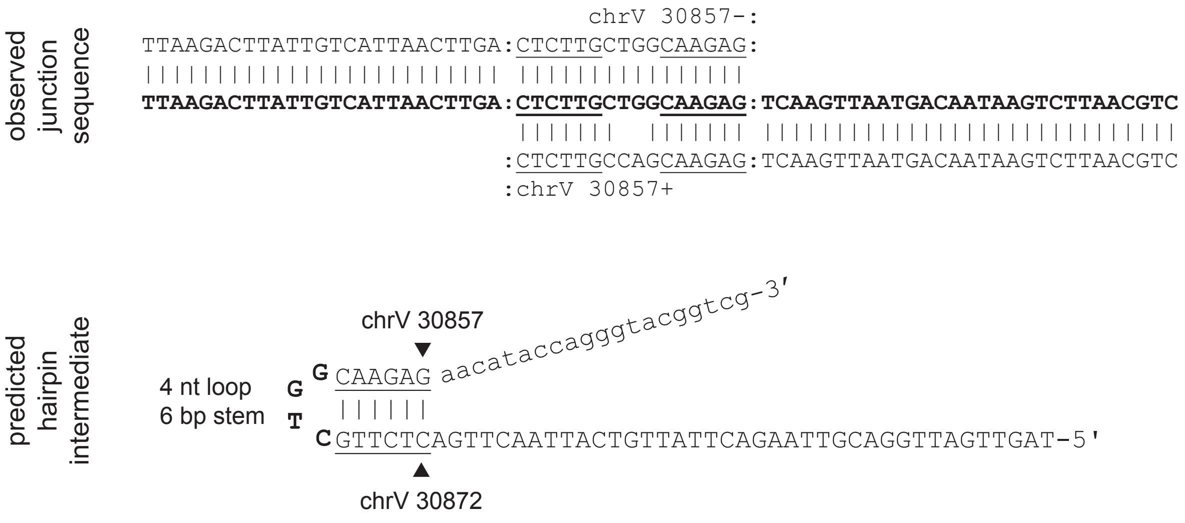

Y. chrV L 34,339-1,219_34,339-501 hairpin

observed junction sequence

```
                    hisG 1149+:                    polylinker:
CTTCAATACCCTGATTGACTGGAACAGCCGGATCTGGCCGGATCC:CCCGGGCT:
||||||||||||||||||||||||||||||||||||||||||||| ||||||||
CTTCAATACCCTGATTGACTGGAACAGCCGGATCTGGCCGGATCC:CCCGGGCT:TCGTCAACCGGTGTTGCCAGCGATAAACGGCA
                                               ||| ||||  ||||||||||||||||||||||||||||||
                                             :CCCAGGCT:TCGTCAACCGGTGTTGCCAGCGATAAACGGCA
                                             :hisG 463-
```

starting genome sequence

```
                                     chrV 30,928+:inserted polylinker
5'-...ATAAGTCTTAACGTCCAATCAACTATAAATCCGAGGGCTACG:CTCGAGGTCGACGGTATCGATAAGCTTGATATCGAATTCCTGCA
      |||||||||||||||||||||||||||||||||||||||||| ||||||||||||||||||||||||||||||||||||||||||||
3'-...TATTCAGAATTGCAGGTTAGTTGATATTTAGGCTCCCGATGC:GAGCTCCAGCTGCCATAGCTATTCGAACTATAGCTTAAGGACGT
                                                                                           ▲
                                                                              chrV 34339-1219
          inserted polylinker:inserted hisG
      GCCCGGGGGATCCGGCCAGATCCG:GCTGTTCCAGTCAATCAGGGTATTGAAGCTCATGGTCTTTACTCCAGCACAGGGTTCCGCCT...-3'
      |||||||||||||||||||||||| ||||||||||||||||||||||||||||||||||||||||||||||||||||||||||||
      CGGGCCCCCTAGGCCGGTCTAGGC:CGACAAGGTCAGTTAGTCCCATAACTTCGAGTACCAGAAATGAGGTCGTGTCCCAAGGCGGA...-5'
```

predicted hairpin intermediate

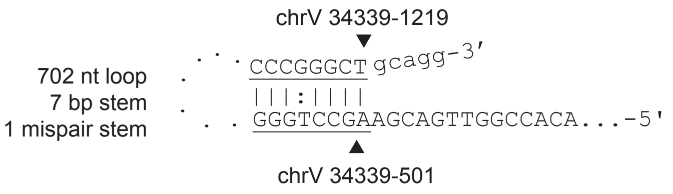

Z. chrV L 34,339-1,211_34,339-1,196 hairpin

observed junction sequence

```
                              hisG 1149:        polylinker:
ATGAGCTTCAATACCCTGATTGACTGGAACAGC:CGGATCTGGCCGGATCC:
||||||||||||||||||||||||||||||||| |||||||||||||||||
ATGAGCTTCAATACCCTGATTGACTGGAACAGC:CGGATCTGGCCGGATCC:G:GCTGTTCCAGTCAATCAGGGTATTGAAGCT
                                              ||||| | ||||||||||||||||||||||||||||||
                                             :GATCC:G:GCTGTTCCAGTCAATCAGGGTATTGAAGCT
                                             :poly   :hisG 1149
```

starting genome sequence

```
                                     chrV 30,928+:inserted polylinker
5'-...ATAAGTCTTAACGTCCAATCAACTATAAATCCGAGGGCTACG:CTCGAGGTCGACGGTATCGATAAGCTTGATATCGAATTCCTGCA
      |||||||||||||||||||||||||||||||||||||||||| ||||||||||||||||||||||||||||||||||||||||||||
3'-...TATTCAGAATTGCAGGTTAGTTGATATTTAGGCTCCCGATGC:GAGCTCCAGCTGCCATAGCTATTCGAACTATAGCTTAAGGACGT

          inserted polylinker:inserted hisG
      GCCCGGGGGATCCGGCCAGATCCG:GCTGTTCCAGTCAATCAGGGTATTGAAGCTCATGGTCTTTACTCCAGCACAGGGTTCCGCCT...-3'
      |||||||||||||||||||||||| ||||||||||||||||||||||||||||||||||||||||||||||||||||||||||||
      CGGGCCCCCTAGGCCGGTCTAGGC:CGACAAGGTCAGTTAGTCCCATAACTTCGAGTACCAGAAATGAGGTCGTGTCCCAAGGCGGA...-5'
              ▲
      chrV 34339-1211
```

predicted hairpin intermediate

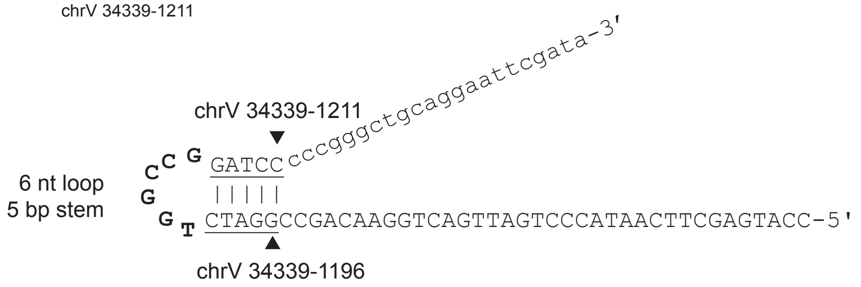

AA. chrV L 34,339-1,126_34,339-1,028 hairpin

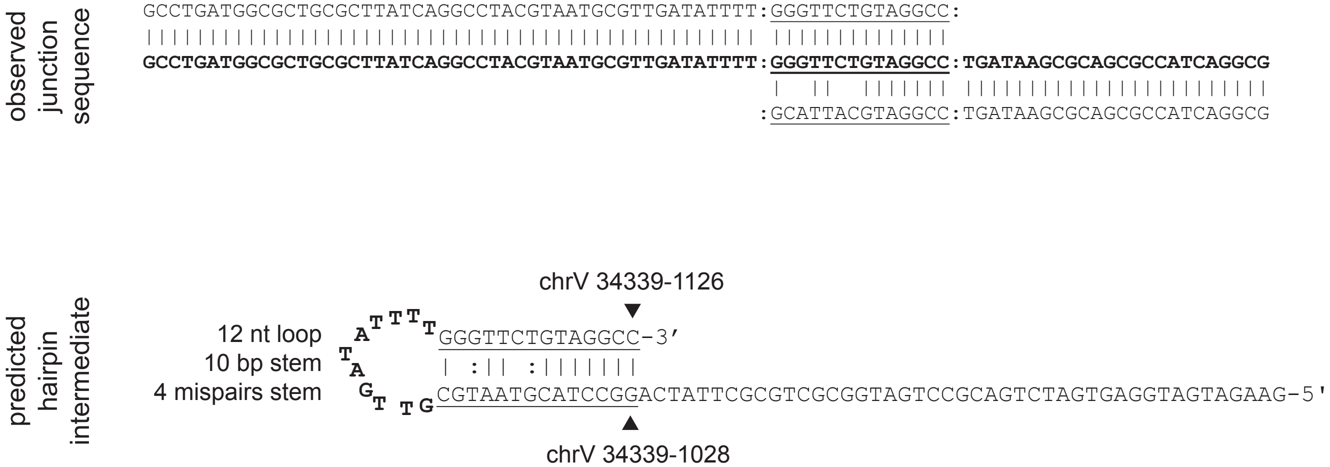

AB. chrV L 34,339-1,091_34,339-1,066 hairpin

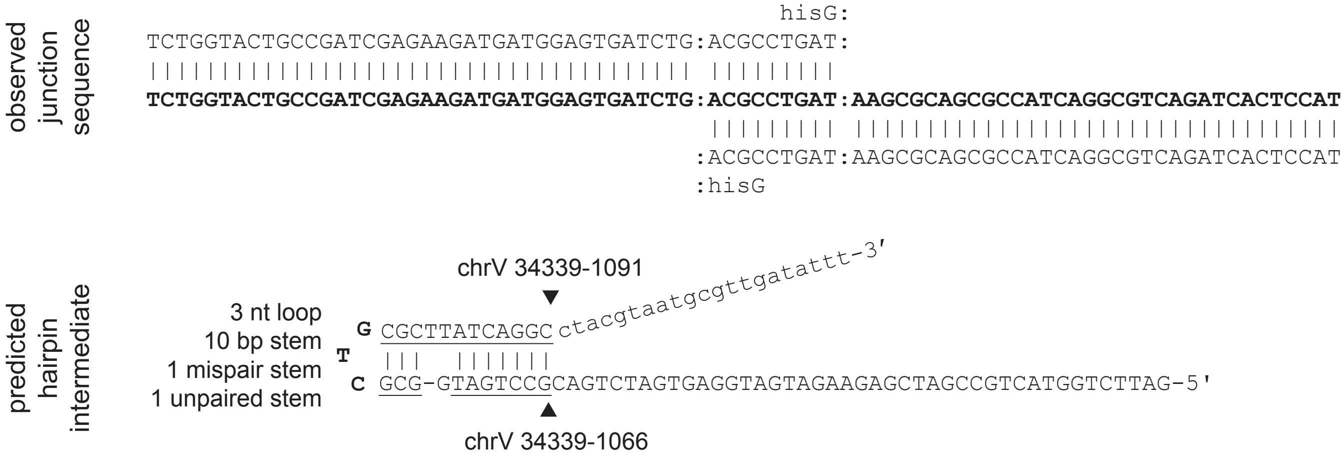

AC. chrV L 34,339-953_34,339-883 hairpin

observed junction sequence

```
                                            hisG 908+:
ACAATTCTGCCGCTGGCAGGCGAGCAACAGCGCGT:GGCGAT:
||||||||||||||||||||||||||||||||||| ||||||
ACAATTCTGCCGCTGGCAGGCGAGCAACAGCGCGT:GGCGAT:AACCTCTTCCAGGCGTTCACTTGGCGCGTG
                                    |||||| ||||||||||||||||||||||||||||||
                                   :GGCGAT:AACCTCTTCCAGGCGTTCACTTGGCGCGTG
                                   :hisG 843-
```

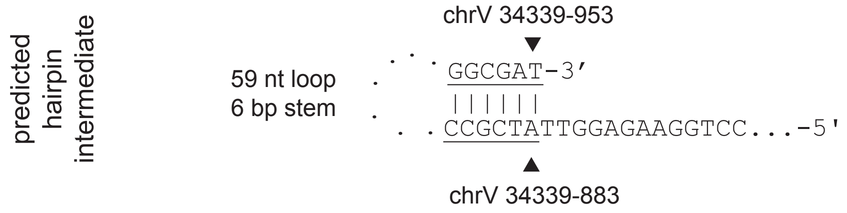

AD. chrV L 34,339-931_34,339-919 hairpin

observed junction sequence

```
                                    hisG:
CAGGCGCCGAAAGGCCGACAATTCTGCCGCT:GGCAG:
||||||||||||||||||||||||||||||| |||||
CAGGCGCCGAAAGGCCGACAATTCTGCCGCT:GGCAG:AATTGTCGGCCTTTCGGCGCCTGGCAGCAGGG
                                ||||| ||||||||||||||||||||||||||||||||
                               :GGCAG:AATTGTCGGCCTTTCGGCGCCTGGCAGCAGGG
```

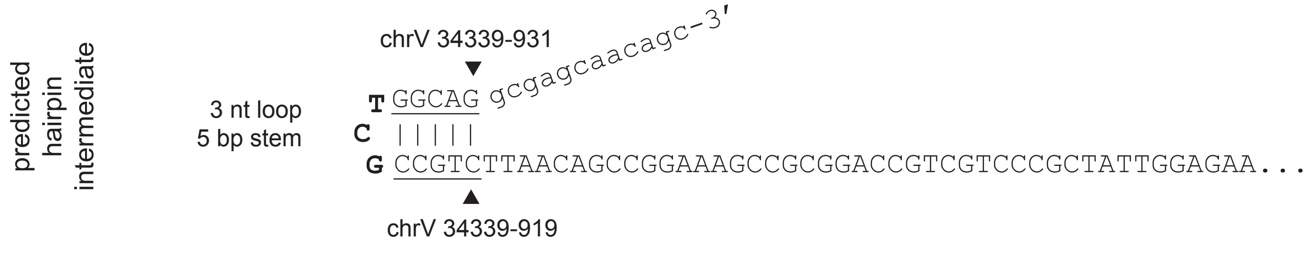

AE. chrV L 34,339-871_34,339-814 hairpin

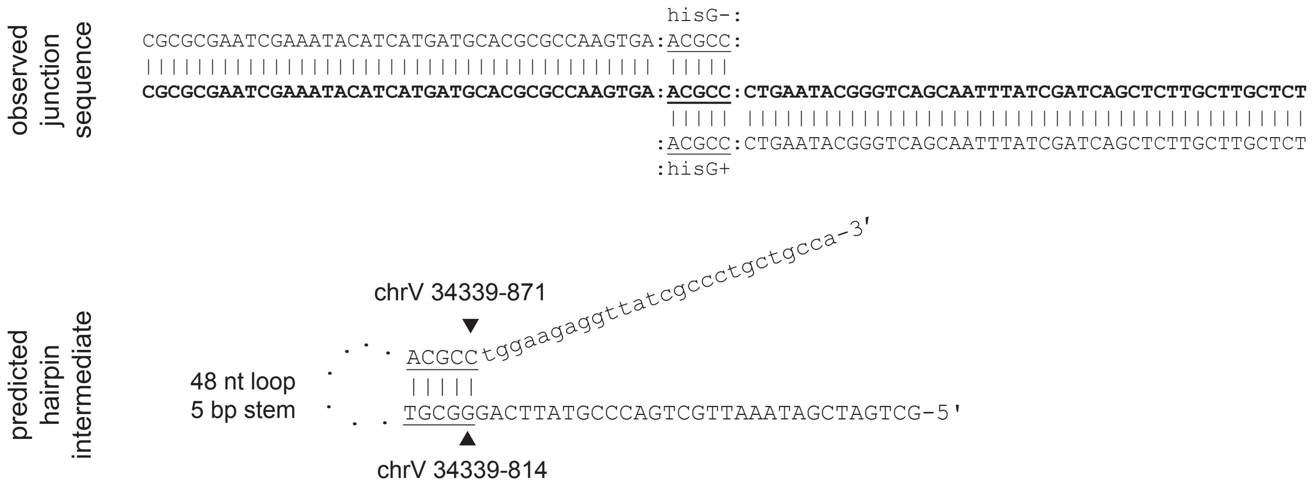

AF. chrV L 34,339-640_34,339-630 hairpin

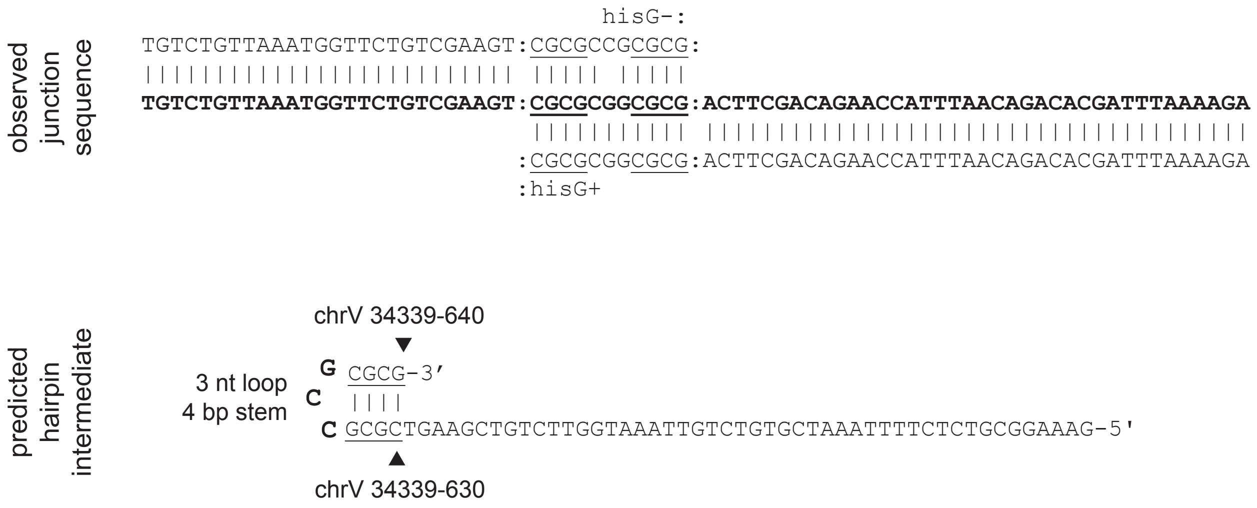

AG. chrV L 34,339-502_34,339-459 hairpin

observed junction sequence

```
                     hisG 457+:
ATCGCTGGCAACACCGGTTGA:CGAAG:
||||||||||||||||||||| |||||
ATCGCTGGCAACACCGGTTGA:CGAAG:TCAAGACGGCGCAGGGTTAAATAGCGT
                      ||||| ||||||||||||||||||||||||||
                     :CGAAG:TCAAGACGGCGCAGGGTTAAATAGCGT
                     :hisG 418-
```

predicted hairpin intermediate

```
                          chrV 34339-502
                                ▼                cctgggacggcccggccgcg-3′
34 nt loop    . . .  CGAAG
5 bp stem     .      |||||
              .  . GCTTCAGTTCTGCCGCGTCCCAATTTATCGCA...-5'
                                ▲
                          chrV 34339-459
```

AH. chrV L 34,339-107_34,339-75 hairpin

observed junction sequence

```
5′-...CAGATCTTCCAGTGGTGCATGAACGCATGAGAAAGCCCCCGGAAGATGATCTTCCGGGGGCTTTCTCATGCGTTCATGCACCACTGG
3′-...GTCTAGAAGGTCACCACGTACTTGCGTACTCTTTCGGGGGCCTTCTACTAGAAGGCCCCCGAAAGAGTACGCAAGTACGTGGTGACC

   AAGATCTGAATTCTTGAAGACGAAAGGGCCTCGTGATACGCCGGGGGATCC:CTGGGTTAGCTTGAAGCGACTTTCTTTCTCTACT...-3′
   TTCTAGACTTAAGAACTTCTGCTTTCCCGGAGCACTATGCGGCCCCCTAGG:GACCCAATCGAACTTCGCTGAAAGAAAGAGATGA...-5′
                                                      :ChrV 34339+
```

predicted hairpin intermediate

```
                    chrV 34339-107
                          ▼
               ATCTTCCGGGGGCTTT-3′
3 nt loop    C  |||||||||||||||
15 bp stem     TAGAAGGCCCCCGAAAGAGTACGCAAGTACGTGGTGACCTTCTAGACTTAAGAACTTCTGCTTTCCCGGAGCAC...-5‛
                          ▲
                    chrV 34339-75
```

AI. chrV L 34,339-91_34,338-46 hairpin

observed junction sequence

```
                                         hisG insertion:
TCTTCCAGTGGTGCATGAACGCATGAGAAAGCCCCC:GGAAGATC:
||||||||||||||||||||||||||||||||||||| ||||||||
TCTTCCAGTGGTGCATGAACGCATGAGAAAGCCCCC:GGAAGATC:TGAATTCTTGAAGACGAAAGGGCCTCGTGATACG
                                      |||||||| ||||||||||||||||||||||||||||||||||
                                     :GGAAGATC:TGAATTCTTGAAGACGAAAGGGCCTCGTGATACG
                                     :hisG insertion
```

predicted hairpin intermediate

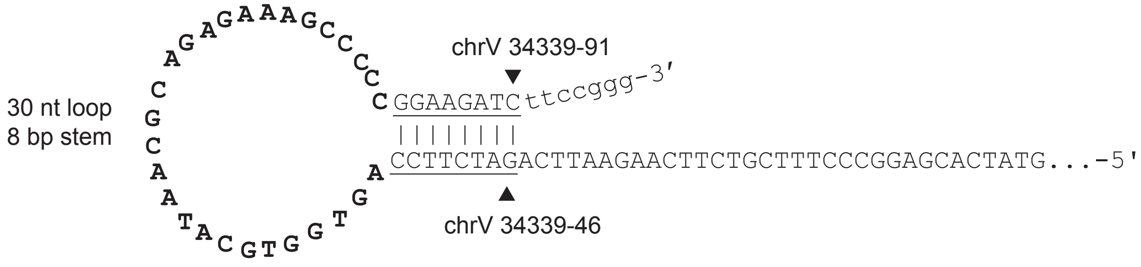

AJ. chrV L 34,759_36,909 hairpin

observed junction sequence

```
                          chrV 36899-:
GAATCGGAGAACGCTATATCCCTT:TTGAAGAACAA:
|||||||||||||||||||||||| |||||||||||
GAATCGGAGAACGCTATATCCCTT:TTGAAGAACAA:AATCAATTGCTATCCAAATCGGAAAGAGAT
                         ||||||||||| ||||||||||||||||||||||||||||||
                        :TTGAAGAACAA:AATCAATTGCTATCCAAATCGGAAAGAGAT
                        :chrV 34759+
```

predicted hairpin intermediate

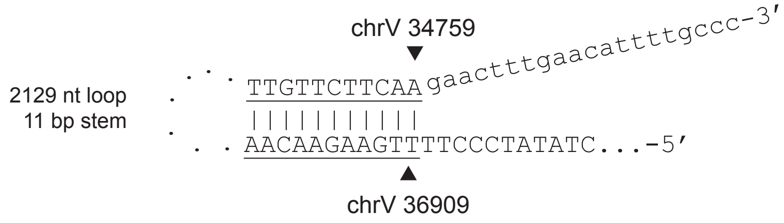

AK. chrV L 35,447_37,677 hairpin

observed junction sequence

```
                                                   chrV 37653-:
CAAATGGCCCTACATGATTACAAAAAAAGA:GAACTTG:ACCTTAATCAAAAGTTTG:
|||||||||||||||||||||||||||||| |||||||  |||||| ||||||| ||
CAAATGGCCCTACATGATTACAAAAAAAGA:GAACTTG:CCCTTAAACAAAAGTCTGGGTTCAGGTGACCAA
                               ||||||| |||||||||||||||||||||||||||||||||
                              :GAACTTG:CCCTTAAACAAAAGTCTGGGTTCAGGTGACCAA
                              :chrV 35447+
```

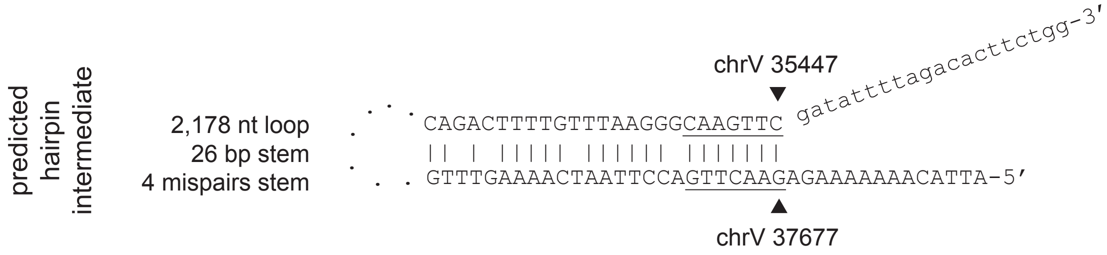

AL. chrV L 35,478_35,494 hairpin

observed junction sequence

```
                                    chrV 35478-:
GGATTTTCTTCGTACAGAAAATGATGGTGAGTCTTGG:TCACC:
||||||||||||||||||||||||||||||||||||| |||||
GGATTTTCTTCGTACAGAAAATGATGGTGAGTCTTGG:TCACC:ATCATTTTCTGTACGAAGAAAATCCAAAT
                                      ||||| |||||||||||||||||||||||||||||
                                     :TCACC:ATCATTTTCTGTACGAAGAAAATCCAAAT
                                     :chrV 35490+
```

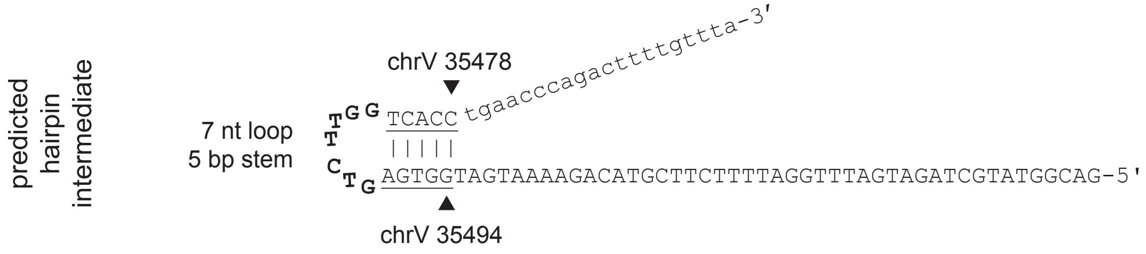

AM. chrV L 35,577_35,595 hairpin

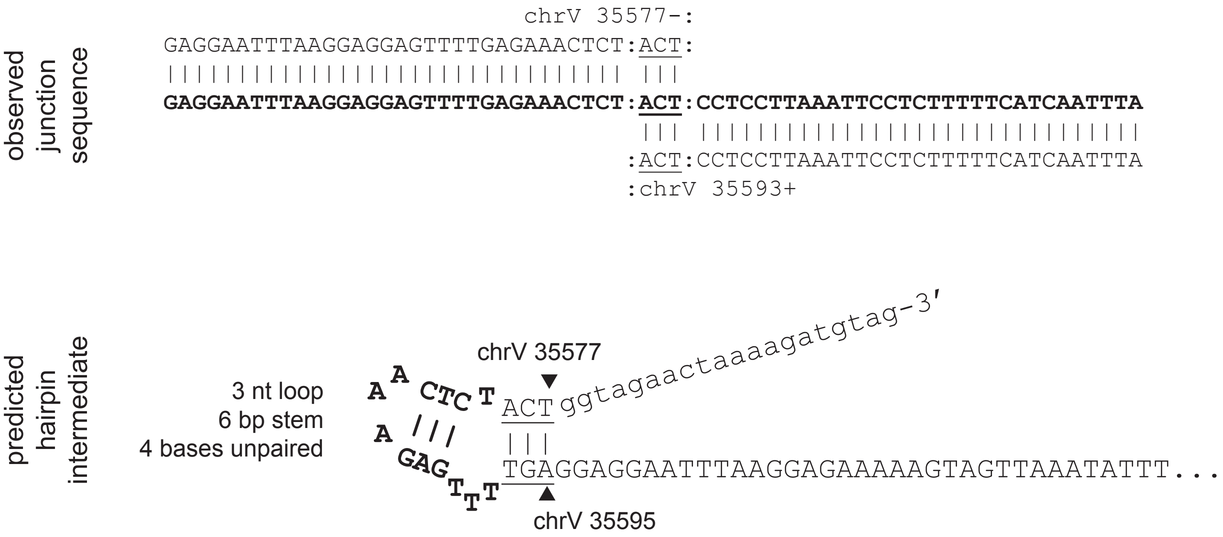

AN. chrV L 35,581_35,596 hairpin

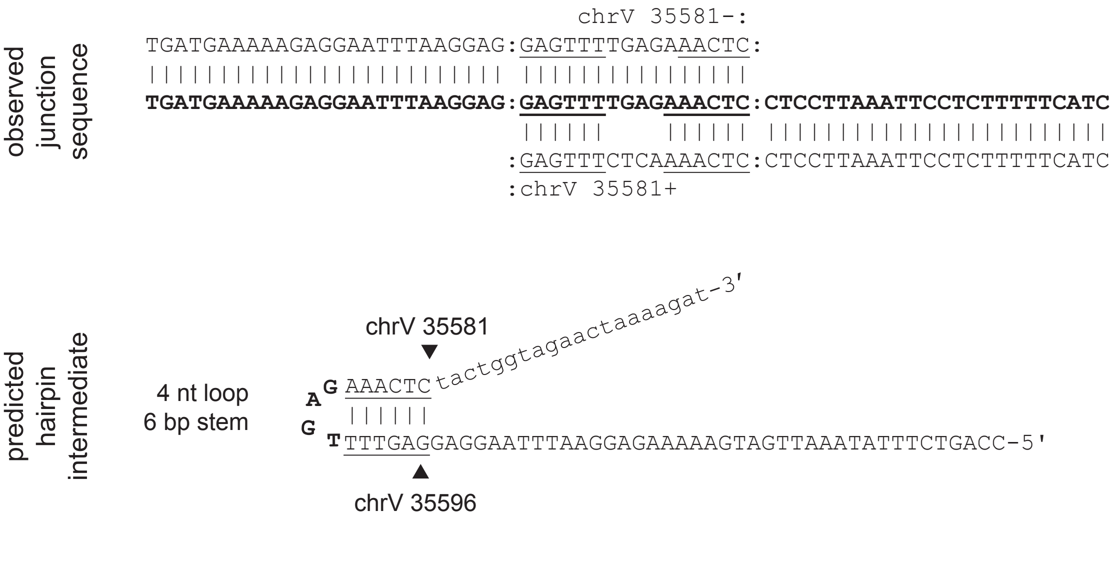

AO. chrV L 35,664_35,725 hairpin

observed junction sequence

```
                                          chrV 35664-:
AATCGGTTACGATTGTTGATGACATGAA:TATTTGA:
|||||||||||||||||||||||||||| |||||||
AATCGGTTACGATTGTTGATGACATGAA:TATTTGA:CCTTTATAGGTCACTTTCACAAGGTCAAAC
                             ||||||| ||||||||||||||||||||||||||||||
                            :TATTTGA:CCTTTATAGGTCACTTTCACAAGGTCAAAC
                            :chrV 35719+
```

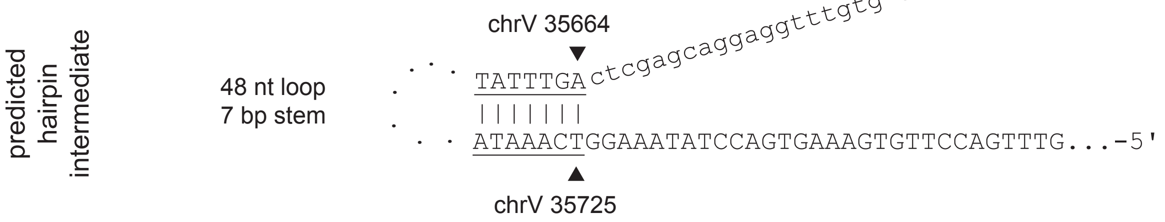

AP. chrV L 35,685_35,698 hairpin

observed junction sequence

```
                                            chrV 35694-:
CCTATAAAGGTCAAATAGGCATGATCTTGTTGGTAGG:AATCG:
||||||||||||||||||||||||||||||||||||| |||||
CCTATAAAGGTCAAATAGGCATGATCTTGTTGGTAGG:AATCG:TAACCGATTCCTACCAACAAGATCATGCCTATTTGA
                                      ||||| ||||||||||||||||||||||||||||||||||||
                                     :AATCG:TAACCGATTCCTACCAACAAGATCATGCCTATTTGA
                                     :chrV 35685+
```

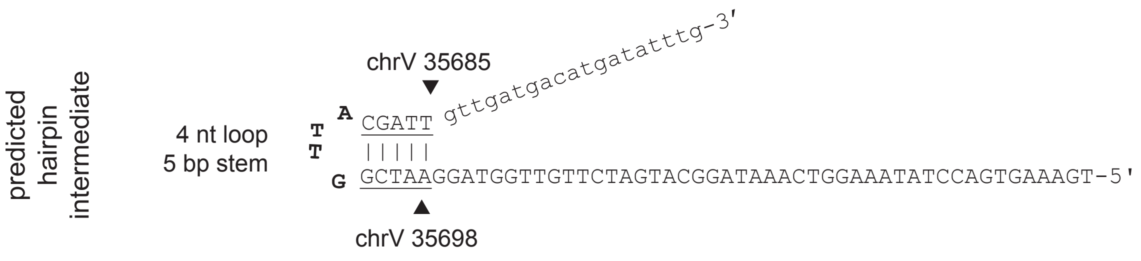

AQ. chrV L 35,694_35,777 hairpin

observed junction sequence

```
                                chrV 35766-:
GTTATTTTCTTTTAAAATCATATATTT:CGACTCATACCA:
||||||||||||||||||||||||||| ||||||||||||
GTTATTTTCTTTTAAAATCATATATTT:CGACTCATACCA:ACAAGATCATGCCTATTTGACCTTTAT
                            ||| || ||||| |||||||||||||||||||||||||||
                           :CGATTCCTACCA:ACAAGATCATGCCTATTTGACCTTTAT
                           :chrV 35694+
```

predicted hiarpin intermediate

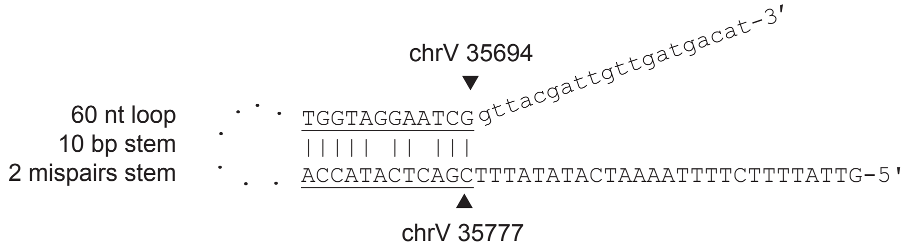

AR. chrV L 35,721_35,754 hairpin

observed junction sequence

```
                          chrV 35721-:
GTTTGACCTTGTGAAAGTGACCTATA:AAGGTCAAA:
|||||||||||||||||||||||||| |||||||||
GTTTGACCTTGTGAAAGTGACCTATA:AAGGTCAAA:CGCTGAAAACATGGTATGAGTCG
                           ||||||||| |||||||||||||||||||||||
                          :AAGGTCAAA:CGCTGAAAACATGGTATGAGTCG
                          :chrV 35746+
```

predicted hairpin intermediate

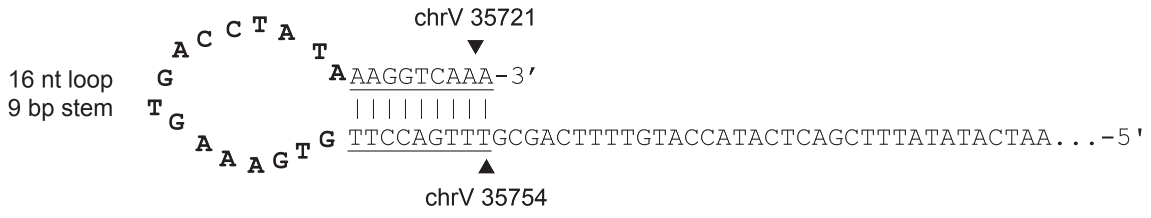

AS. chrV L 35,723_35,738 hairpin

observed junction sequence

```
                                            chrV 35723-:
TTCAGCGTTTGACCTTGTGAAAG:TGACCTATAAAGGTCA:
||||||||||||||||||||||| ||||||||||||||||
TTCAGCGTTTGACCTTGTGAAAG:TGACCTATAAAGGTCA:CTTTCACAAGGTCAAACGCTGAAAACATGG
                         ||||||| | |||||| ||||||||||||||||||||||||||||||
                        :TGACCTTTATAGGTCA:CTTTCACAAGGTCAAACGCTGAAAACATGG
                        :chrV 35723+
```

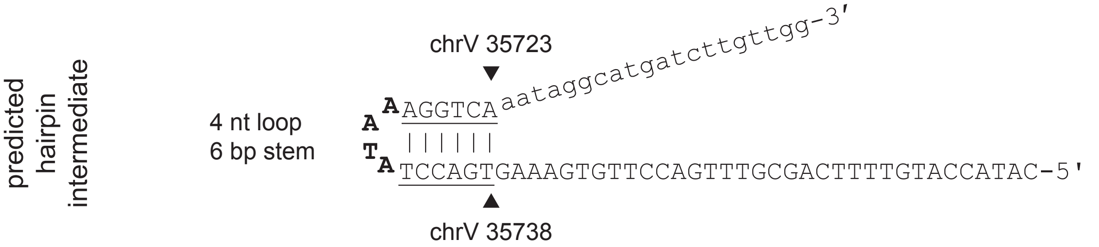

AT. chrV L 35,837_35,872 hairpin

observed junction sequence

```
                                  chrV 35867-:
GTTCACGTGAACCTGCATTTATCATTACA:GGAAAA:
||||||||||||||||||||||||||||| ||||||
GTTCACGTGAACCTGCATTTATCATTACA:GGAAAA:ACGTATCATTTACAGGTGCTACTCTTTTCCTGTA
                               |||||| ||||||||||||||||||||||||||||||||||
                              :GGAAAA:ACGTATCATTTACAGGTGCTACTCTTTTCCTGTA
                              :chrV 35837+
```

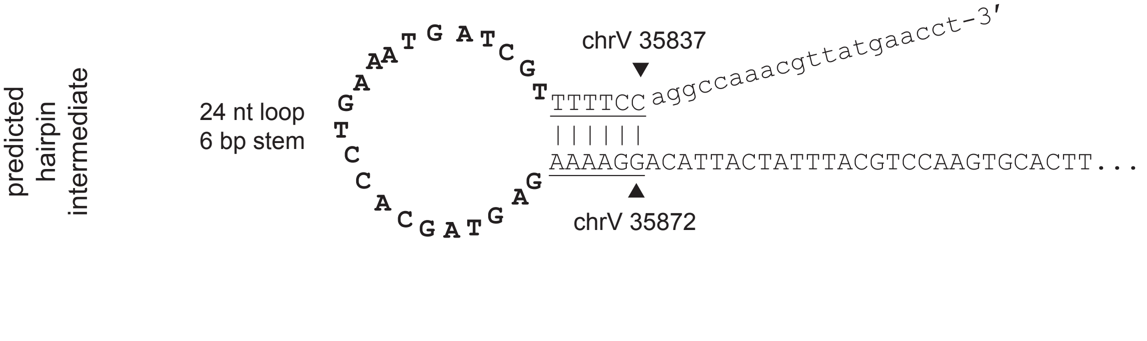

AU. chrV L 35,889_35,902 hairpin

observed junction sequence

```
                                        chrV 35889-:
TTATTATTGGTGTCATTTCTTTT:GGTTCACGTGAACC:
||||||||||||||||||||||| ||||||||||||||
TTATTATTGGTGTCATTTCTTTT:GGTTCACGTGAACC:AAAAGAAATGACACCAATAATAACAAAAG
                        |||||||||||||| |||||||||||||||||||||||||||||
                       :GGTTCACGTGAACC:AAAAGAAATGACACCAATAATAACAAAAG
                       :chrV 35889+
```

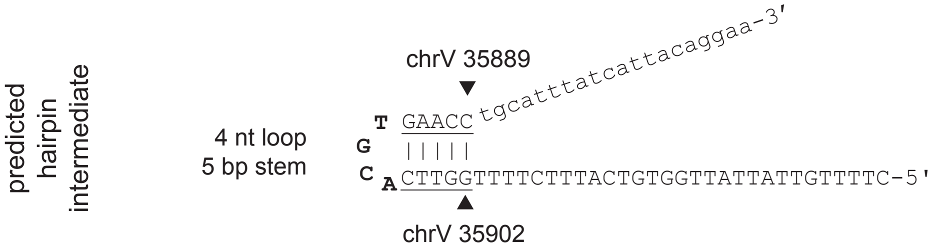

AV. chrV L 37,132_37,150 hairpin

observed junction sequence

```
                                    chrV 37132-:
ATAATGCAGAATCTGACAACTGCAACCAGC:GCGGTT:
|||||||||||||||||||||||||||||| ||||||
ATAATGCAGAATCTGACAACTGCAACCAGC:GCGGTT:GTCAGATTCTGCATTATTAGTTTTTTATTTTC
                               || ||| ||||||||||||||||||||||||||||||||
                              :GCAGTT:GTCAGATTCTGCATTATTAGTTTTTTATTTTC
                              :chrV 37145+
```

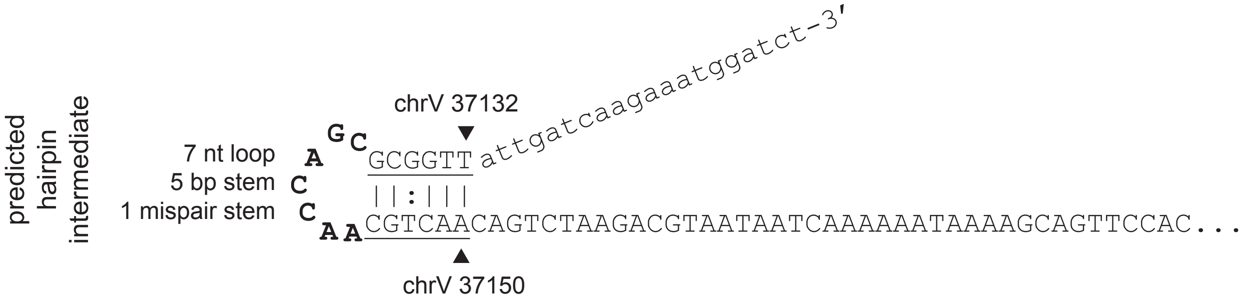

AW. chrV L 38,072_38,086 hairpin

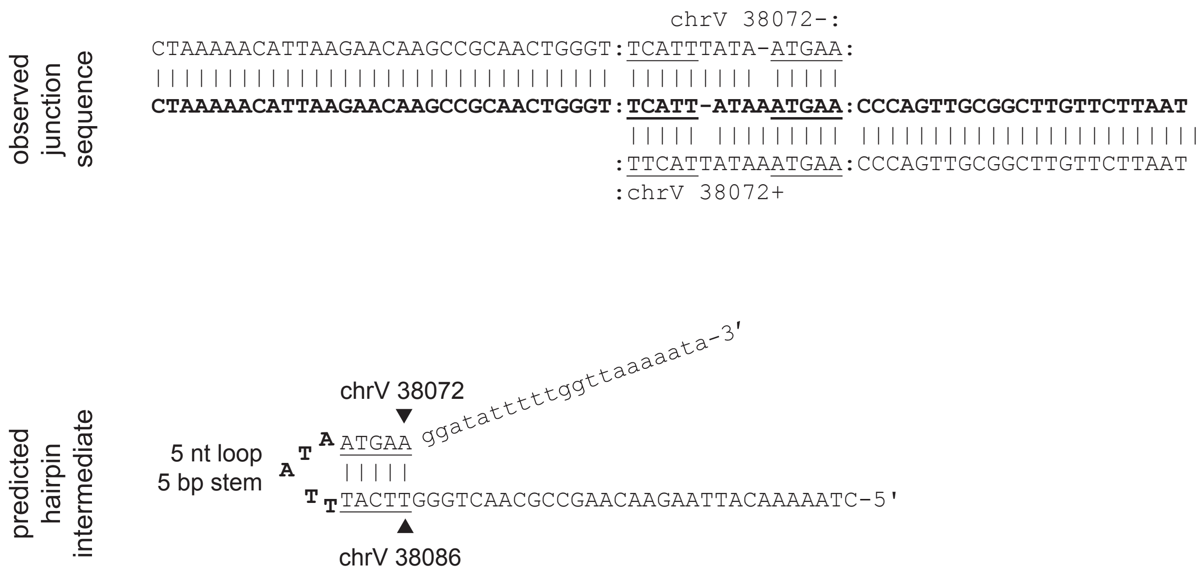

AX. chrV L 38,848_38,871 hairpin

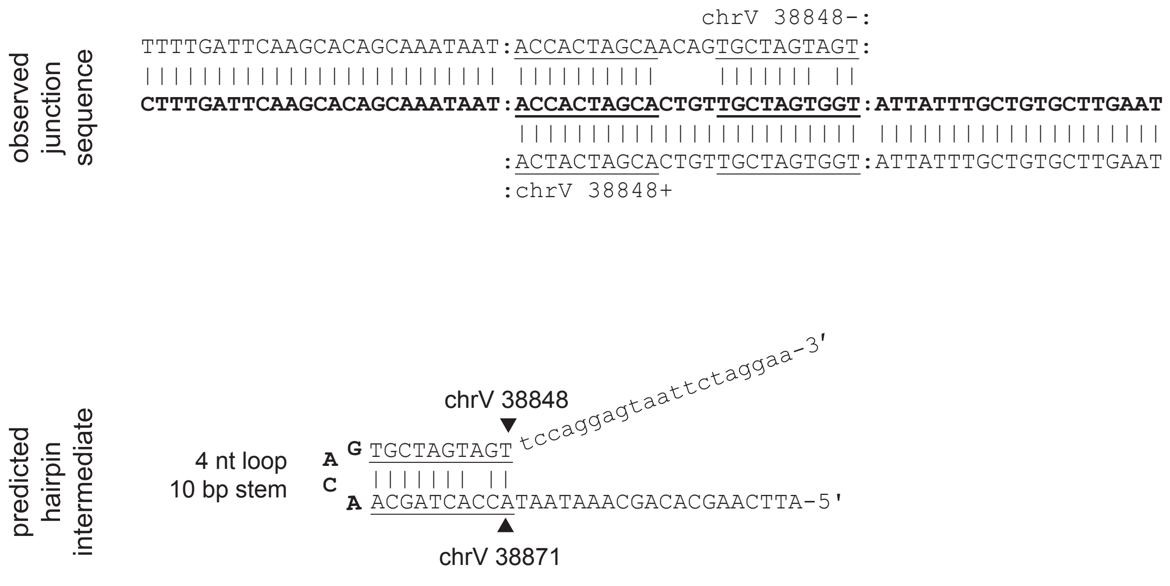

Figure 2 - Source Data 1 (page 26 of 32)

AY. chrV L 39,661_39,681 hairpin

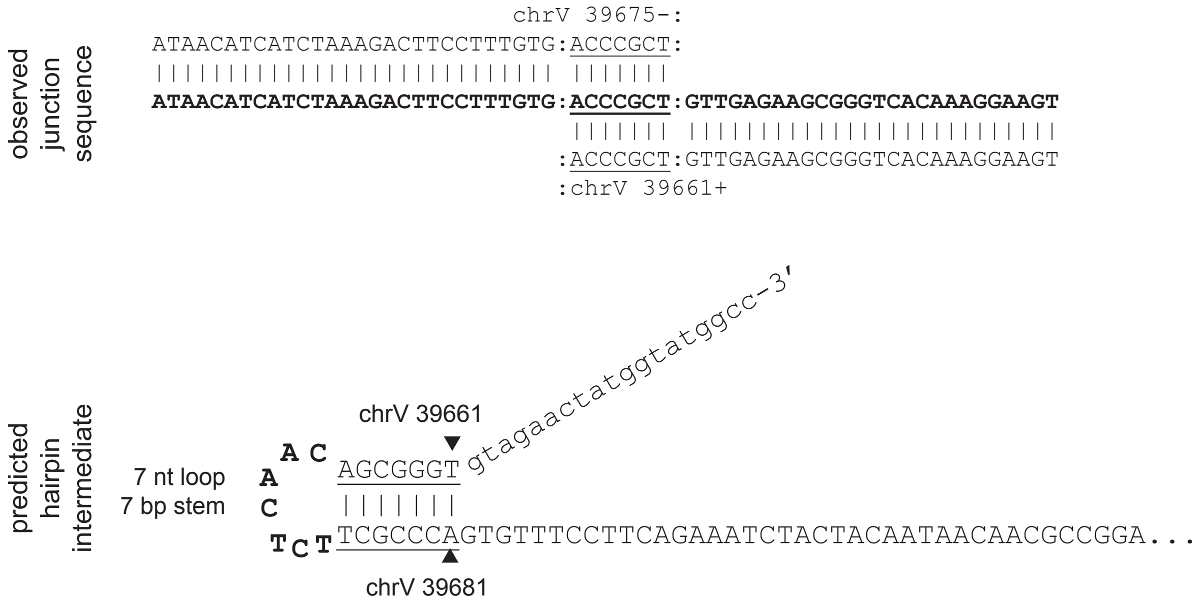

AZ. chrV L 40,281_40,326 hairpin

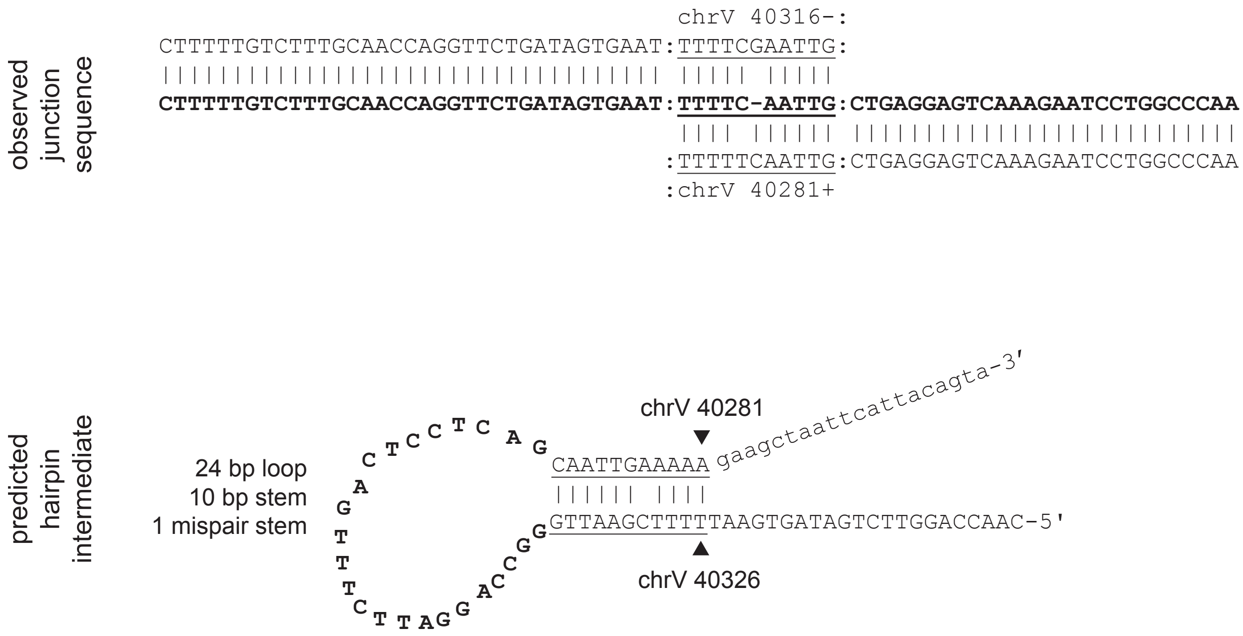

BA. chrV L 40,877_40,894 hairpin

observed junction sequence

```
                                           chrV 40877-:
AACGACGAAGATCTCGACGGTAACGGCCA:CGGTACC:
||||||||||||||||||||||||||||| |||||||
AACGACGAAGATCTCGACGGTAACGGCCA:CGGTACC:GTCGAGATCTTCGTCGTTAAGTGGGAT
                               || |||| |||||||||||||||||||||||||||
                             :CGTTACC:GTCGAGATCTTCGTCGTTAAGTGGGAT
                             :chrV 40888+
```

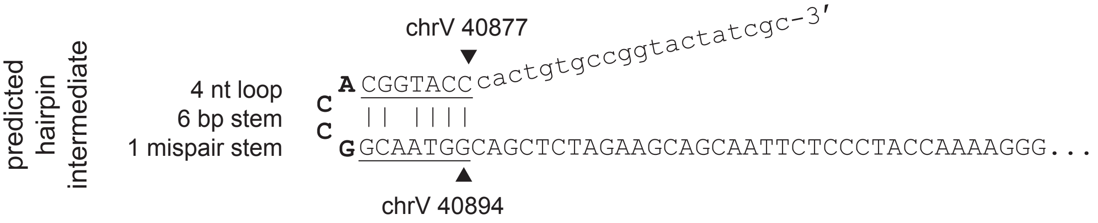

BB. chrV L 40,968_40,983 hairpin

observed junction sequence

```
                                      chrV 40968-:
CGCGGTGTCACGTCCTATGTTA:TTGACACGGGTGTCAA:
|||||||||||||||||||||| ||||||||||||||||
CGCGGTGTCACGTCCTATGTTA:TTGACACGGGTGTCAA:TAACATAGGACGTGACACCGCGACCGGCATCA
                       |||||||| ||||||| ||||||||||||||||||||||||||||||||
                      :TTGACACCCGTGTCAA:TAACATAGGACGTGACACCGCGACCGGCATCA
                      :chrV 40968+
```

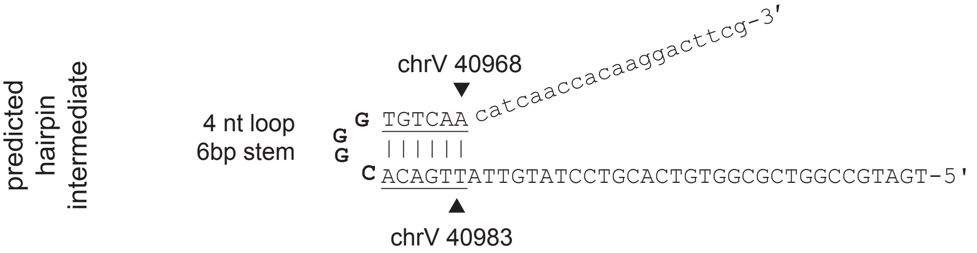

BC. chrV L 40,994_43,019 hairpin

observed junction sequence

```
                                 chrV 43013-:
AATCTGCTAATCGTGCAAGTAAGGGTTCT:CGTGACA:
||||||||||||||||||||||||||||| |||||||
AATCTGCTAATCGTGCAAGTAAGGGTTCT:CGTGACA:CCGCGACCGGCATCATCATCGTAGAGATACT
                               ||||||| |||||||||||||||||||||||||||||||
                              :CGTGACA:CCGCGACCGGCATCATCATCGTAGAGATACT
                              :chrV 40994+
```

predicted hairpin intermediate

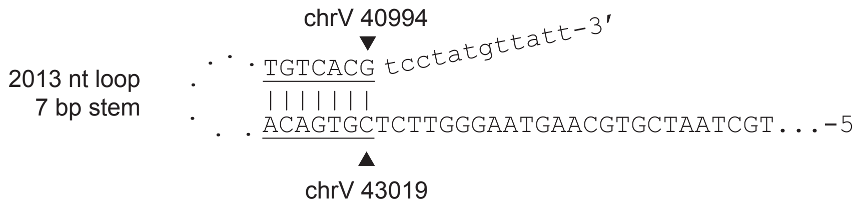

BD. chrV L 41,350_41,363 hairpin

observed junction sequence

```
                                chrV 41350-:
GCCCCTCAAGAAGAGATCGATTTCCACAA:GGAAA:
||||||||||||||||||||||||||||| |||||
GCCCCTCAAGAAGAGATCGATTTCCACAA:GGAAA:TCGATCTCTTCTTGAGGGGCACCTCTCTTGAAGAC
                               ||||| ||||||||||||||||||||||||||||||||||||
                              :GGAAA:TCGATCTCTTCTTGAGGGGCACCTCTCTTGAAGAC
                              :chrV 41359+
```

predicted hairpin intermediate

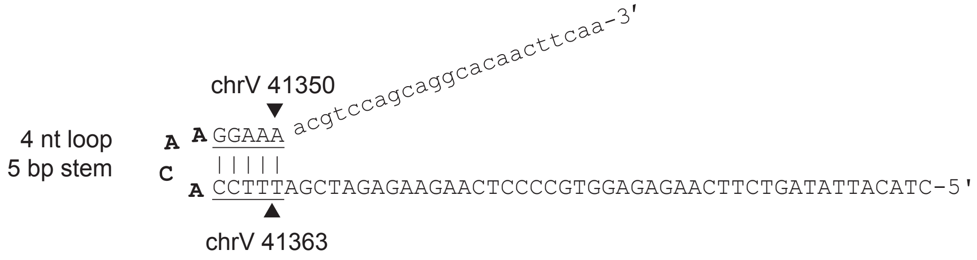

BE. chrV L 41,622_41,681 hairpin

observed junction sequence

```
                                   chrV 41674-:
CAACGGTAAACGTGCAAGTGGCTC:TCATGGGA:
|||||||||||||||||||||||| |||| |||
CAACGGTAAACGTGCAAGTGGCTC:TCATTGGA:ACTTTCATGCTTAGGCTTCATGCCCTTT
                         |||||||| ||||||||||||||||||||||||||||
                        :TCATTGGA:ACTTTCATGCTTAGGCTTCATGCCCTTT
                        :chrV 41622+
```

predicted hairpin intermediate

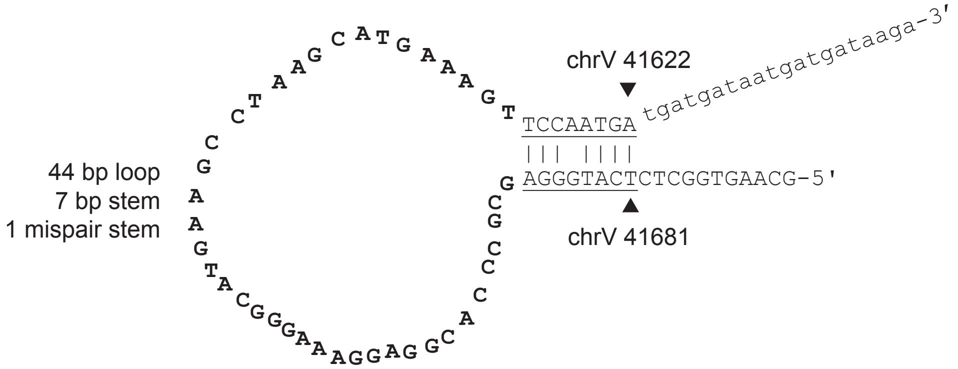

BF. chrV L 42,277_42,290 hairpin

observed junction sequence

```
                                             chrV 42277-:
AGATCAGGTCTGTGCCTACTATGCACTTATG:CCCGGGGTCCCGGG:
||||||||||||||||||||||||||||||| ||||||   ||||||
AGATCAGGTCTGTGCCTACTATGCACTTATG:CCCGGGACCCCGGG:CATAAGTGCATAGTAGGCACAGACC
                                |||||||||||||| ||||||||||||||||||||||||
                               :CCCGGGACCCCGGG:CATAAGTGCATAGTAGGCACAGACC
                               :chrV 42277+
```

predicted hairpin intermediate

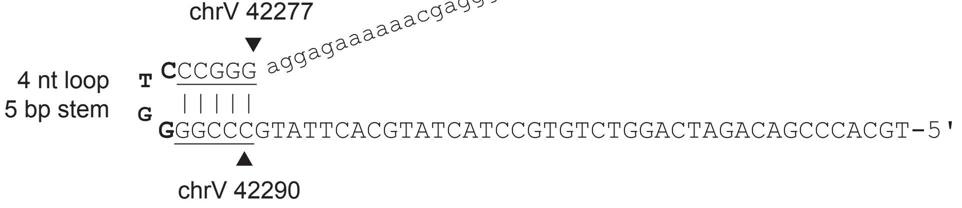

BG. chrV L 42,855_42,869 hairpin

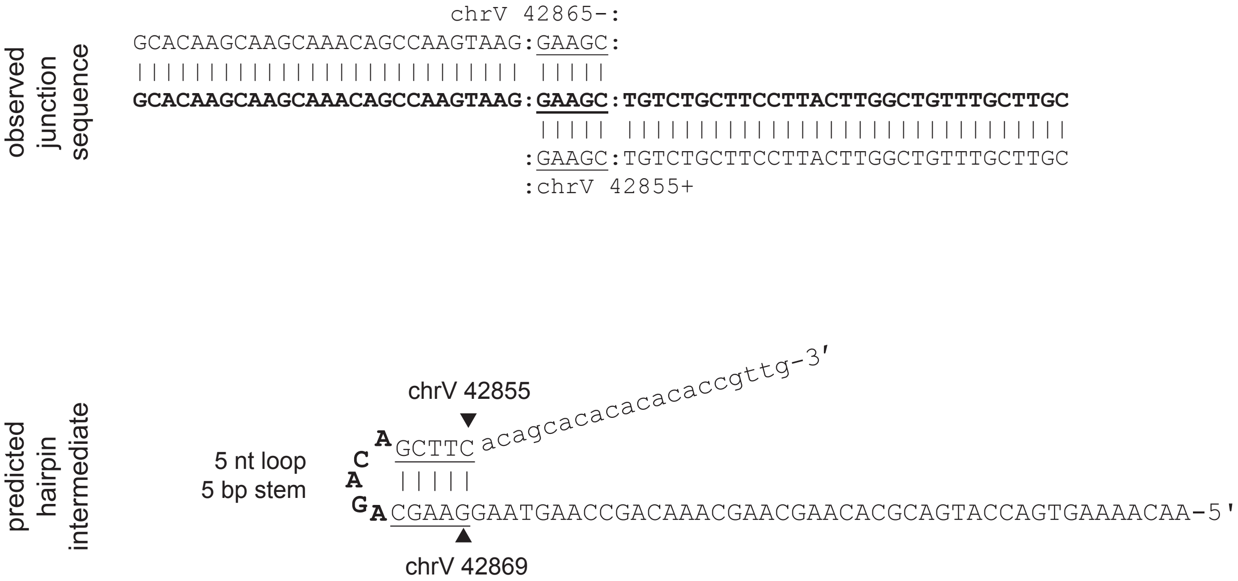

BH. chrV L 42,939_42,955 hairpin

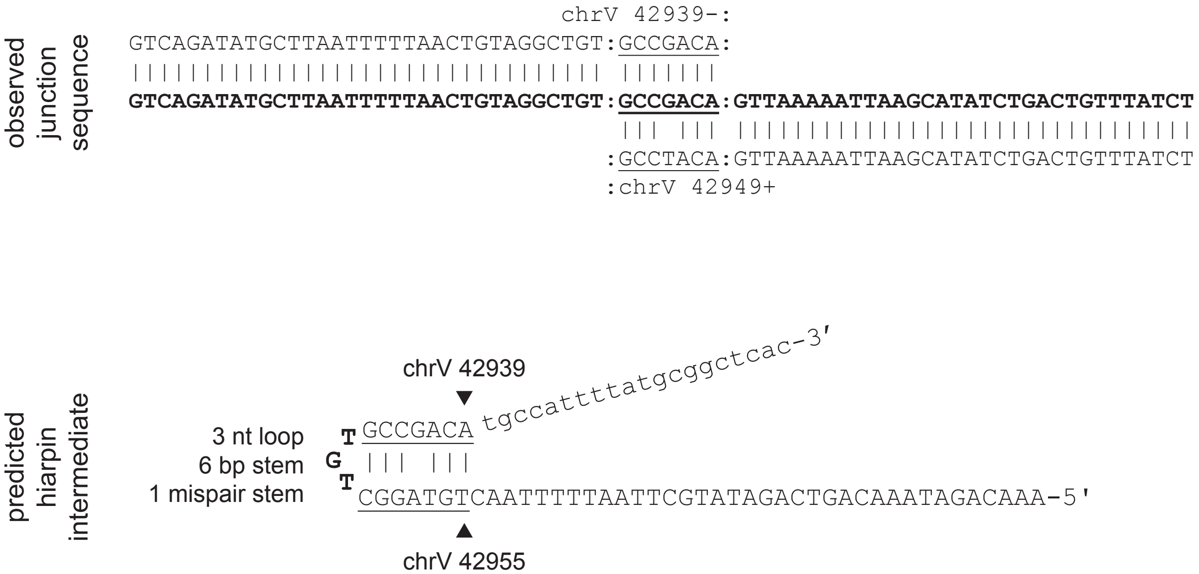

BI. chrV L 51,934_51,962 hairpin

observed junction sequence

```
                        chrV 51962+:
TGAAAA:GCTGATGGGTGT-CAAGCACGCCCATCAGC:
|||||| |||||||||||| ||||||||||||||||
TGAAAA:GCTGATGGGTGT-CAAGCACGCCCATCAGC:TTTTCAAGTTATGTATACGTTAATT
       ||||||||| || |  | || |||||||||  ||||||||||||||||||||||||
      :GCTGATGGGCGTGCTTG-ACACCCATCAGC:TTTTCAAGTTATGTATACGTTAATT
      :chrV 51962-
```

predicted hiarpin intermediate

```
                                         chrV 51934
                                             ▼
5'-...GCAATTAACGTATACATAACTTGAAAAGCTGATGGGTGT C A
                                 ||||||||| ||     A
                                 CGACTACCCGCA C G
                           3'-acg    ▲
                                 chrV 51962
```

5 nt loop
11 bp stem
1 mispair stem

BJ. chrV L 72,380_72,456 hairpin

observed junction sequence

```
                                           chrV 72456+:
ATCAAGCTTTTGAATACGATAA:TGCAAACCACTTAATCAGTGGTTTGCA:
|||||||||||||||||||||| ||||||||||||||||||||||||||
ATCAAGCTTTTGAATACGATAA:TGCAAACCACTTAATCAGTGGTTTGCA:TTATCGTATTCAAAAGCTTGATC
                       ||||||||||| | | ||||||||||| |||||||||||||||||||||||
                      :TGCAAACCACTGATTAAGTGGTTTGCA:TTATCGTATTCAAAAGCTTGATC
                      :chrV 72456-
```

predicted hairpin intermediate

```
                             chrV 72380
                                 ▼
...TGGATCAAGCTTTTGAATACGATAATGCAAACCACT T A
                             |||||||||||    A
                          3'-ACGTTTGGTGA C T
                             ▲
                         chrV 72456
```

5 nt loop
11 bp stem

BK. chrV L 84,266_84,283 hairpin

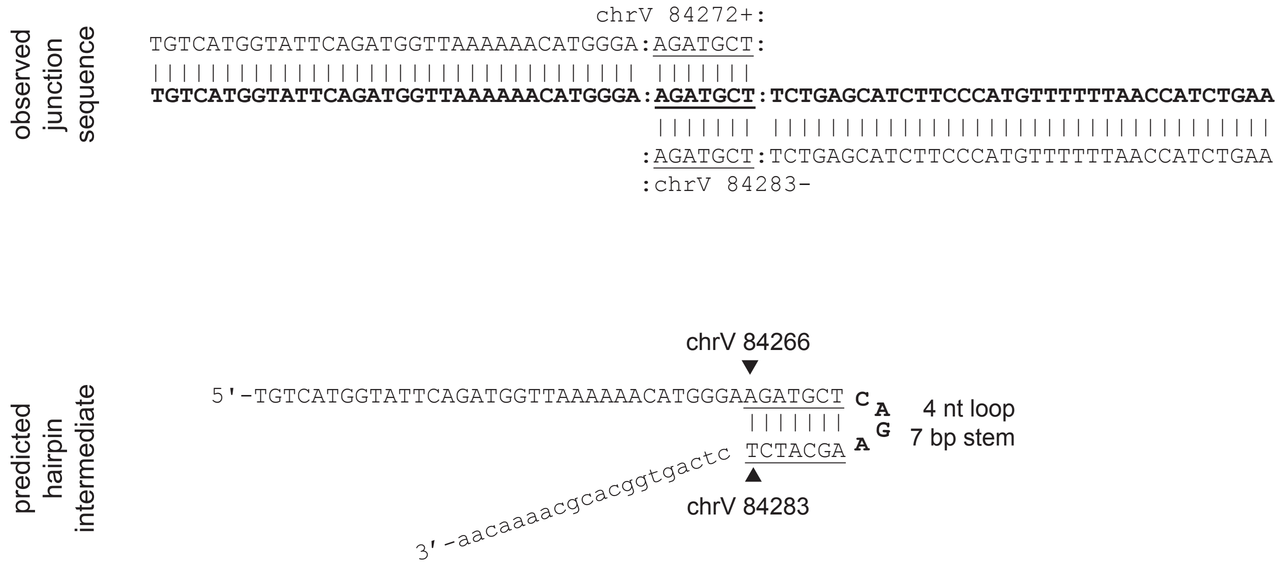

Supplement: Figure 2—source data 1. — The structures of the key predicted ssDNA hairpin intermediates observed (panels A to BK are sorted by the chromosomal position of the predicted hairpin). The chrV coordinates for each predicted hairpin correspond to the coordinates reported for the foldback inversion-containing GCRs. [file elife-58223-fig2-data1.pdf]
