# Supplementary material for: Mechanisms underlying genome instability mediated by formation of foldback inversions in Saccharomyces cerevisiae
Source: eLife. 2020 Aug 7;9:e58223. doi: 10.7554/eLife.58223 (PMC7467729; doi:10.7554/eLife.58223)
Supplement: Supplementary file 2. [file elife-58223-supp2.docx]

**Supplementary File 2. Statistics for Whole Genome Sequencing results.**

**A.** Statistics from *sae2Δ* uGCR strains

| **No.** | **Sample** | **No. Read Pairs** | **No. Uniquely Mapping Read Pairs** | **% Read 1 Mapped** | **% Read 2 Mapped** | **Median Read Pair Distance (bp)** | **Median Read Depth*** |
| --- | --- | --- | --- | --- | --- | --- | --- |
| 1 | PGSP1987 | 29,985,455 | 21,498,852 | 99.55% | 95.21% | 369 | 177 |
| 2 | PGSP1989 | 26,622,019 | 14,826,084 | 95.92% | 65.25% | 570 | 134 |
| 3 | PGSP1990 | 18,084,973 | 9,690,085 | 97.65% | 62.75% | 584 | 91 |
| 4 | PGSP1991 | 12,753,375 | 6,710,977 | 98.29% | 61.63% | 592 | 61 |
| 5 | PGSP1992 | 21,261,456 | 11,272,293 | 97.77% | 62.34% | 586 | 105 |
| 6 | PGSP1993 | 10,752,456 | 7,688,692 | 97.63% | 79.66% | 552 | 70 |
| 7 | PGSP1994 | 30,004,406 | 17,016,473 | 96.12% | 66.13% | 573 | 148 |
| 8 | PGSP1995 | 19,320,397 | 10,349,590 | 96.54% | 61.63% | 584 | 97 |
| 9 | PGSP1996 | 20,794,318 | 11,397,415 | 97.45% | 63.80% | 566 | 104 |
| 10 | PGSP1997 | 19,005,867 | 9,528,746 | 95.63% | 57.73% | 591 | 84 |
| 11 | PGSP1998 | 18,138,629 | 9,440,972 | 96.36% | 60.79% | 587 | 84 |
| 12 | PGSP1999 | 17,485,593 | 94,68,872 | 97.14% | 62.95% | 548 | 83 |
| 13 | PGSP2000 | 23,100,821 | 13,204,540 | 97.72% | 67.50% | 557 | 115 |
| 14 | PGSP3633 | 25,103,916 | 12,415,761 | 97.11% | 57.53% | 571 | 118 |
| 15 | PGSP3634 | 32,172,687 | 23,081,510 | 93.36% | 84.96% | 634 | 141 |
| 16 | PGSP3636 | 24,521,146 | 18,596,471 | 97.57% | 89.62% | 626 | 135 |
| 17 | PGSP3637 | 20,917,170 | 15,858,319 | 98.42% | 88.02% | 668 | 98 |
| 18 | PGSP3638 | 28,920,021 | 21,907,529 | 97.76% | 89.05% | 643 | 149 |
| 19 | PGSP3641 | 38,840,986 | 27,854,072 | 98.35% | 87.43% | 682 | 209 |
| 20 | PGSP3642 | 23,389,280 | 17,356,127 | 96.07% | 87.88% | 653 | 112 |

**B.** Statistics from *sae2-S267A* uGCR strains

| **No.** | **Sample** | **No. Read Pairs** | **No. Uniquely Mapping Read Pairs** | **% Read 1 Mapped** | **% Read 2 Mapped** | **Median Read Pair Distance (bp)** | **Median Read Depth*** |
| --- | --- | --- | --- | --- | --- | --- | --- |
| 1 | PGSP4760 | 11,057,602 | 7,874,721 | 89.60% | 82.80% | 595 | 47 |
| 2 | PGSP4761 | 10,101,839 | 7,382,069 | 93.85% | 86.63% | 570 | 52 |
| 3 | PGSP4762 | 10,111,933 | 7,675,997 | 96.05% | 89.27% | 571 | 55 |
| 4 | PGSP4763 | 9,605,157 | 7,062,590 | 93.82% | 85.87% | 597 | 47 |
| 5 | PGSP4764 | 13,906,746 | 10,450,561 | 94.62% | 88.50% | 562 | 66 |
| 6 | PGSP4765 | 13,388,364 | 9,542,387 | 92.50% | 84.11% | 580 | 65 |
| 7 | PGSP4766 | 12,903,648 | 9,657,017 | 95.42% | 88.72% | 574 | 64 |
| 8 | PGSP4767 | 12,347,600 | 8,936,303 | 92.42% | 85.36% | 575 | 58 |
| 9 | PGSP4768 | 13,987,007 | 10,302,683 | 96.00% | 87.51% | 600 | 78 |
| 10 | PGSP4769 | 12,554,579 | 9,301,237 | 95.20% | 88.87% | 564 | 71 |
| 11 | PGSP4770 | 14,698,559 | 11,165,261 | 96.56% | 90.47% | 559 | 74 |
| 12 | PGSP4771 | 12,404,331 | 9,181,428 | 93.33% | 87.58% | 559 | 62 |

**C.** Statistics from *sae2-MT9* uGCR strains

| **No.** | **Sample** | **No. Read Pairs** | **No. Uniquely Mapping Read Pairs** | **% Read 1 Mapped** | **% Read 2 Mapped** | **Median Read Pair Distance (bp)** | **Median Read Depth*** |
| --- | --- | --- | --- | --- | --- | --- | --- |
| 1 | PGSP4780 | 14,764,999 | 10,654,421 | 94.17% | 87.63% | 557 | 85 |
| 2 | PGSP4781 | 11,169,682 | 8,230,190 | 95.76% | 88.79% | 571 | 65 |
| 3 | PGSP4782 | 11,271,409 | 8,426,666 | 96.90% | 90.07% | 563 | 68 |
| 4 | PGSP4783 | 16,075,408 | 11,903,477 | 95.94% | 89.60% | 559 | 93 |
| 5 | PGSP4784 | 17,047,978) | 12,756,975 | 98.08% | 91.43% | 556 | 104 |
| 6 | PGSP4785 | 12,566,811 | 9,364,038 | 96.20% | 90.35% | 534 | 76 |
| 7 | PGSP4786 | 10,295,756 | 7,440,446 | 96.11% | 89.51% | 561 | 60 |
| 8 | PGSP4787 | 10,526,435 | 7,572,403 | 96.91% | 89.34% | 582 | 58 |
| 9 | PGSP4788 | 13,052,620 | 9,234,436 | 94.89% | 87.97% | 561 | 72 |
| 10 | PGSP4789 | 11,147,186 | 8,669,897 | 96.06% | 90.14% | 531 | 66 |
| 11 | PGSP4790 | 14,111,331 | 10,327,299 | 97.85% | 91.54% | 552 | 81 |
| 12 | PGSP4791 | 15,091,076 | 10,969,713 | 97.20% | 90.67% | 554 | 84 |

**D.** Statistics from *sae2Δ hs-del* uGCR strains

| **No.** | **Sample** | **No. Read Pairs** | **No. Uniquely Mapping Read Pairs** | **% Read 1 Mapped** | **% Read 2 Mapped** | **Median Read Pair Distance (bp)** | **Median Read Depth*** |
| --- | --- | --- | --- | --- | --- | --- | --- |
| 1 | PGSP4998 | 8,625,528 | 3,453,360 | 92.29% | 48.82% | 457 | 41 |
| 2 | PGSP4999 | 6,766,592 | 3,550,724 | 97.26% | 62.41% | 458 | 37 |
| 3 | PGSP5000 | 7,951,960 | 4,524,011 | 98.05% | 67.69% | 437 | 43 |
| 4 | PGSP5001 | 7,374,279 | 3,703,054 | 96.68% | 59.56% | 479 | 40 |
| 5 | PGSP5002 | 9,838,553 | 4,946,700 | 98.19% | 60.92% | 498 | 53 |
| 6 | PGSP5003 | 11,669,668 | 5,903,769 | 98.16% | 61.08% | 466 | 58 |
| 7 | PGSP5004 | 13,885,565 | 7,313,703 | 97.62% | 64.28% | 463 | 72 |
| 8 | PGSP5005 | 8,812,561 | 4,260,492 | 96.87% | 58.66% | 486 | 44 |
| 9 | PGSP5006 | 11,095,897 | 5,502,960 | 98.33% | 59.65% | 494 | 59 |
| 10 | PGSP5007 | 12,894,585 | 6,742,120 | 96.56% | 62.54% | 471 | 63 |
| 11 | PGSP5008 | 21,066,231 | 10,783,566 | 98.89% | 62.59% | 495 | 107 |
| 12 | PGSP5009 | 8,738,152 | 4,742,270 | 98.19% | 64.89% | 477 | 46 |

**E.** Statistics from wild-type chrV:30,843-gRNA (telomeric to hotspot) uGCR strains

| **No.** | **Sample** | **No. Read Pairs** | **No. Uniquely Mapping Read Pairs** | **% Read 1 Mapped** | **% Read 2 Mapped** | **Median Read Pair Distance (bp)** | **Median Read Depth*** |
| --- | --- | --- | --- | --- | --- | --- | --- |
| 1 | PGSP4842 | 7,478,032 | 4,333,030 | 78.35% | 68.26% | 575 | 36 |
| 2 | PGSP4843 | 10,346,729 | 6,676,610 | 86.42% | 76.05% | 569 | 56 |
| 3 | PGSP4844 | 8,608,895 | 5,199,813 | 82.02% | 71.66% | 569 | 43 |
| 4 | PGSP4845 | 7,500,641 | 4,485,816 | 82.12% | 70.26% | 563 | 38 |
| 5 | PGSP4846 | 7,795,931 | 5,385,179 | 92.21% | 81.63% | 558 | 44 |
| 6 | PGSP4847 | 7,630,348 | 4,873,365 | 87.25% | 75.70% | 550 | 40 |
| 7 | PGSP5028 | 9,226,505 | 1,831,974 | 78.50% | 22.44% | 490 | 24 |
| 8 | PGSP5029 | 7,661,008 | 1,459,162 | 77.77% | 21.56% | 485 | 20 |
| 9 | PGSP5030 | 9,774,662 | 2,141,723 | 81.82% | 24.84% | 477 | 28 |
| 10 | PGSP5031 | 8,986,279 | 1,689,432 | 73.28% | 21.26% | 490 | 22 |
| 11 | PGSP5032 | 8,847,960 | 2,060,596 | 91.61% | 26.40% | 499 | 27 |
| 12 | PGSP5033 | 7,681,208 | 1,678,134 | 81.57% | 24.79% | 498 | 21 |

**F.** Statistics from *sae2Δ* chrV:30,843-gRNA (telomeric to hotspot) uGCR strains

| **No.** | **Sample** | **No. Read Pairs** | **No. Uniquely Mapping Read Pairs** | **% Read 1 Mapped** | **% Read 2 Mapped** | **Median Read Pair Distance (bp)** | **Median Read Depth*** |
| --- | --- | --- | --- | --- | --- | --- | --- |
| 1 | PGSP4829 | 14,609,813 | 9,896,335 | 91.37% | 81.71% | 550 | 87 |
| 2 | PGSP4830 | 7,421,458 | 4,546,010 | 85.35% | 75.46% | 550 | 41 |
| 3 | PGSP4831 | 10,306,335 | 6,776,282 | 89.61% | 79.83% | 545 | 60 |
| 4 | PGSP4832 | 9,022,697 | 5,910,450 | 88.86% | 79.29% | 545 | 51 |
| 5 | PGSP4833 | 11,556,235 | 7,994,847 | 94.80% | 84.60% | 548 | 69 |
| 6 | PGSP4834 | 12,044,333 | 7,416,613 | 83.45% | 74.71% | 544 | 65 |
| 7 | PGSP5034 | 11,839,767 | 1,635,975 | 53.73% | 15.94% | 489 | 25 |
| 8 | PGSP5035 | 9,024,003 | 1,671,878 | 69.98% | 21.37% | 492 | 24 |
| 9 | PGSP5036 | 6,181,592 | 1,223,528 | 84.19% | 22.76% | 496 | 20 |
| 10 | PGSP5037 | 8,867,121 | 1,905,947 | 78.51% | 24.64% | 484 | 29 |
| 11 | PGSP5038 | 7,991,479 | 1,857,863 | 91.15% | 26.64% | 503 | 28 |

**G.** Statistics from wild-type chrV:34,470-gRNA (centromeric to hotspot) uGCR strains

| **No.** | **Sample** | **No. Read Pairs** | **No. Uniquely Mapping Read Pairs** | **% Read 1 Mapped** | **% Read 2 Mapped** | **Median Read Pair Distance (bp)** | **Median Read Depth*** |
| --- | --- | --- | --- | --- | --- | --- | --- |
| 1 | PGSP4836 | 12,869,855 | 7,653,321 | 80.58% | 69.64% | 586 | 66 |
| 2 | PGSP4837 | 9,746,462 | 5,786,372 | 79.07% | 69.41% | 572 | 51 |
| 3 | PGSP4838 | 5,965,503 | 4,176,207 | 93.04% | 82.03% | 560 | 36 |
| 4 | PGSP4839 | 7,592,517 | 4,915,684 | 85.53% | 75.40% | 573 | 43 |
| 5 | PGSP4840 | 8,563,421 | 5,825,014 | 91.45% | 80.31% | 570 | 49 |
| 6 | PGSP4841 | 7,773,183 | 5,170,393 | 88.99% | 78.06% | 569 | 46 |
| 7 | PGSP5022 | 5,266,445 | 398,541 | 45.06% | 8.96% | 448 | 8 |
| 8 | PGSP5023 | 6,667,034 | 1,450,875 | 89.26% | 24.43% | 479 | 24 |
| 9 | PGSP5024 | 5,557,125 | 1,195,891 | 91.29% | 24.23% | 516 | 18 |
| 10 | PGSP5025 | 6,705,270 | 1,361,350 | 82.30% | 22.88% | 480 | 23 |
| 11 | PGSP5026 | 9,150,940 | 2,300,553 | 83.93% | 28.48% | 470 | 31 |
| 12 | PGSP5027 | 9,784,748 | 1,798,913 | 77.87% | 20.68% | 473 | 32 |

**H.** Statistics from *sae2Δ* chrV:34,470-gRNA (centromeric to hotspot) uGCR strains

| **No.** | **Sample** | **No. Read Pairs** | **No. Uniquely Mapping Read Pairs** | **% Read 1 Mapped** | **% Read 2 Mapped** | **Median Read Pair Distance (bp)** | **Median Read Depth*** |
| --- | --- | --- | --- | --- | --- | --- | --- |
| 1 | PGSP4822 | 6,610,201 | 4,032,893 | 83.57% | 72.27% | 570 | 35 |
| 2 | PGSP4823 | 7,170,405 | 4,731,581 | 89.26% | 78.47% | 552 | 40 |
| 3 | PGSP4824 | 8,286,046 | 5,621,564 | 93.60% | 82.71% | 565 | 48 |
| 4 | PGSP4825 | 9105,221 | 5,949,222 | 88.24% | 77.56% | 561 | 51 |
| 5 | PGSP4826 | 11,119,458 | 7,498,533 | 91.25% | 81.20% | 561 | 66 |
| 6 | PGSP4827 | 8,549,936 | 5,583,349 | 89.41% | 77.77% | 560 | 48 |
| 7 | PGSP5039 | 7,345,636 | 1,433,259 | 70.30% | 22.54% | 484 | 21 |
| 8 | PGSP5040 | 7,052,082 | 1,442,854 | 82.02% | 23.32% | 483 | 24 |
| 9 | PGSP5041 | 3,230,093 | 380,337 | 69.47% | 13.45% | 544 | 8 |
| 10 | PGSP5042 | 6,750,973 | 1,348,172 | 80.34% | 22.79% | 495 | 22 |
| 11 | PGSP5043 | 5,506,687 | 474,943 | 79.58% | 9.89% | 449 | 15 |
| 12 | PGSP5044 | 8,012,048 | 1,668,050 | 86.36% | 23.75% | 484 | 28 |
| 13 | PGSP5045 | 7,677,015 | 644,586 | 74.89% | 9.60% | 445 | 19 |

**I.** Statistics from *sae2Δ* chrV:25,817-1,749 gRNA (telomeric to hotspot in *CAN1*) uGCR strains

| **No.** | **Sample** | **No. Read Pairs** | **No. Uniquely Mapping Read Pairs** | **% Read 1 Mapped** | **% Read 2 Mapped** | **Median Read Pair Distance (bp)** | **Median Read Depth*** |
| --- | --- | --- | --- | --- | --- | --- | --- |
| 1 | PGSP4974 | 10,099,904 | 6,737,513 | 95.65% | 79.89% | 622 | 59 |
| 2 | PGSP4975 | 9,867,740 | 6,531,348 | 95.76% | 78.97% | 627 | 56 |
| 3 | PGSP4976 | 8,443,981 | 5,854,776 | 96.15% | 81.66% | 610 | 48 |
| 4 | PGSP4977 | 8,707,456 | 5,481,198 | 90.86% | 74.57% | 641 | 48 |
| 5 | PGSP4978 | 11,295,963 | 7,539,387 | 96.43% | 79.73% | 650 | 64 |
| 6 | PGSP4979 | 11,837,669 | 7,549,818 | 94.40% | 75.15% | 641 | 65 |
| 7 | PGSP4980 | 14,127,806 | 9,312,139 | 94.77% | 78.93% | 640 | 80 |
| 8 | PGSP4981 | 10,144,017 | 6,499,133 | 90.68% | 76.09% | 616 | 58 |
| 9 | PGSP4982 | 13,390,467 | 8,768,359 | 97.42% | 78.52% | 658 | 78 |
| 10 | PGSP4983 | 12,055,154 | 7,900,788 | 96.20% | 78.79% | 654 | 69 |
| 11 | PGSP4984 | 14,247,091 | 9,734,151 | 97.74% | 82.74% | 629 | 86 |
| 12 | PGSP4985 | 11,137,547 | 7,488,346 | 95.76% | 80.27% | 645 | 64 |

**J.** Statistics from *sae2Δ* chrV:35,709 gRNA (centromeric to hotspot) uGCR strains

| **No.** | **Sample** | **No. Read Pairs** | **No. Uniquely Mapping Read Pairs** | **% Read 1 Mapped** | **% Read 2 Mapped** | **Median Read Pair Distance (bp)** | **Median Read Depth*** |
| --- | --- | --- | --- | --- | --- | --- | --- |
| 1 | PGSP4986 | 11,427,967 | 7,640,753 | 94.50% | 78.67% | 626 | 69 |
| 2 | PGSP4987 | 9,401,476 | 6,364,465 | 96.43% | 80.65% | 636 | 54 |
| 3 | PGSP4988 | 9,300,356 | 6,291,204 | 96.89% | 80.62% | 628 | 55 |
| 4 | PGSP4989 | 12,669,002 | 8,421,368 | 95.78% | 79.43% | 641 | 71 |
| 5 | PGSP4990 | 13,224,648 | 8,884,937 | 98.08% | 80.76% | 645 | 75 |
| 6 | PGSP4991 | 9,535,226 | 6,198,865 | 92.30% | 77.75% | 625 | 50 |
| 7 | PGSP4992 | 9,795,754 | 6,597,547 | 96.50% | 80.80% | 638 | 57 |
| 8 | PGSP4993 | 9,228,573 | 6,285,669 | 96.15% | 81.15% | 619 | 54 |
| 9 | PGSP4994 | 9,813,373 | 6,444,193 | 93.87% | 78.83% | 619 | 56 |
| 10 | PGSP4995 | 9,854,176 | 6,390,718 | 94.63% | 78.41% | 613 | 56 |
| 11 | PGSP4996 | 11,996,428 | 8,213,565 | 97.24% | 81.72% | 623 | 70 |
| 12 | PGSP4997 | 12,558,054 | 8,322,145 | 96.02% | 79.28% | 622 | 69 |

**K.** Statistics from *sae2Δ* chrV:34,339-110 gRNA (telomeric to hotspot) uGCR strains

| **No.** | **Sample** | **No. Read Pairs** | **No. Uniquely Mapping Read Pairs** | **% Read 1 Mapped** | **% Read 2 Mapped** | **Median Read Pair Distance (bp)** | **Median Read Depth*** |
| --- | --- | --- | --- | --- | --- | --- | --- |
| 1 | PGSP5010 | 9,023,757 | 4,114,793 | 89.30% | 53.46% | 509 | 36 |
| 2 | PGSP5011 | 6,842,718 | 3,347,929 | 93.89% | 58.11% | 489 | 32 |
| 3 | PGSP5012 | 7,743,157 | 3,586,954 | 96.50% | 54.65% | 516 | 37 |
| 4 | PGSP5013 | 11,807,289 | 5,506,449 | 90.15% | 54.91% | 509 | 54 |
| 5 | PGSP5014 | 12,220,140 | 5,948,074 | 94.70% | 57.60% | 502 | 56 |
| 6 | PGSP5015 | 8,580,046 | 4,433,596 | 95.25% | 60.38% | 484 | 40 |
| 7 | PGSP5016 | 7,139,462 | 3,502,603 | 93.96% | 58.17% | 496 | 34 |
| 8 | PGSP5017 | 6,078,128 | 3,063,051 | 96.85% | 58.99% | 502 | 32 |
| 9 | PGSP5018 | 8,880,624 | 4,383,844 | 97.00% | 57.02% | 515 | 36 |
| 10 | PGSP5019 | 8,010,394 | 2,548,169 | 87.37% | 37.31% | 479 | 30 |
| 11 | PGSP5020 | 10,689,964 | 5,184,968 | 96.12% | 56.60% | 504 | 48 |
| 12 | PGSP5021 | 10,052,056 | 3,508,231 | 95.19% | 40.82% | 489 | 37 |

**L.** Statistics from *sae2Δ tel1Δ* uGCR strains

| **No.** | **Sample** | **No. Read Pairs** | **No. Uniquely Mapping Read Pairs** | **% Read 1 Mapped** | **% Read 2 Mapped** | **Median Read Pair Distance (bp)** | **Median Read Depth*** |
| --- | --- | --- | --- | --- | --- | --- | --- |
| 1 | PGSP3669 | 25,124,468 | 18,848,597 | 98.27% | 90.53% | 638 | 145 |
| 2 | PGSP3670 | 23,990,038 | 17,685,467 | 97.17% | 89.56% | 610 | 130 |
| 3 | PGSP3671 | 23,416,910 | 17,596,424 | 98.24% | 90.36% | 616 | 137 |
| 4 | PGSP3672 | 23,761,302 | 17,657,320 | 98.60% | 90.97% | 597 | 133 |
| 5 | PGSP3674 | 17,458,174 | 13,157,758 | 98.09% | 90.88% | 621 | 102 |
| 6 | PGSP3675 | 29,564,787 | 22,301,814 | 98.96% | 91.99% | 600 | 175 |
| 7 | PGSP3678 | 30,113,622 | 20,282,031 | 97.10% | 81.26% | 706 | 168 |
| 8 | PGSP3681 | 21,478,934 | 15,594,717 | 97.58% | 86.42% | 630 | 124 |
| 9 | PGSP3682 | 17,100,218 | 11,561,099 | 96.58% | 82.50% | 659 | 89 |
| 10 | PGSP4010 | 22,675,869 | 16,421,598 | 98.34% | 86.31% | 648 | 133 |
| 11 | PGSP4045 | 23,277,614 | 16,442,133 | 98.70% | 84.94% | 649 | 133 |

**M.** Statistics from *exo1Δ* uGCR strains

| **No.** | **Sample** | **No. Read Pairs** | **No. Uniquely Mapping Read Pairs** | **% Read 1 Mapped** | **% Read 2 Mapped** | **Median Read Pair Distance (bp)** | **Median Read Depth*** |
| --- | --- | --- | --- | --- | --- | --- | --- |
| 1 | PGSP773 | 25,575,782 | 17,352,263 | 98.98% | 82.35% | 550 | 151 |
| 2 | PGSP774 | 23,904,529 | 16,549,634 | 99.01% | 85.56% | 506 | 147 |
| 3 | PGSP775 | 22,864,310 | 16,000,091 | 98.92% | 85.47% | 524 | 135 |
| 4 | PGSP776 | 28,643,378 | 19,950,478 | 97.26% | 86.27% | 457 | 166 |
| 5 | PGSP777 | 27,109,424 | 18,448,168 | 98.18% | 83.01% | 539 | 154 |
| 6 | PGSP778 | 23,456,255 | 15,674,325 | 98.67% | 81.96% | 528 | 141 |
| 7 | PGSP779 | 21,922,780 | 14,545,490 | 97.85% | 81.25% | 552 | 124 |
| 8 | PGSP780 | 25,532,000 | 17,400,000 | 99.04% | 84.80% | 528 | 153 |
| 9 | PGSP781 | 23,516,113 | 15,260,780 | 98.11% | 80.46% | 560 | 131 |
| 10 | PGSP782 | 23,065,373 | 15,521,419 | 98.49% | 82.53% | 550 | 130 |
| 11 | PGSP783 | 24,019,224 | 16,063,724 | 97.92% | 83.32% | 540 | 138 |

**N.** Statistics from *sae2Δ* *exo1Δ* uGCR strains

| **No.** | **Sample** | **No. Read Pairs** | **No. Uniquely Mapping Read Pairs** | **% Read 1 Mapped** | **% Read 2 Mapped** | **Median Read Pair Distance (bp)** | **Median Read Depth*** |
| --- | --- | --- | --- | --- | --- | --- | --- |
| 1 | PGSP3683 | 26,770,694 | 18,219,990 | 92.08% | 80.81% | 645 | 144 |
| 2 | PGSP3690 | 30,413,923 | 22,289,284 | 98.90% | 87.80% | 631 | 181 |
| 3 | PGSP3696 | 23,275,035 | 15,369,769 | 92.44% | 78.02% | 667 | 123 |
| 4 | PGSP4107 | 20,907,961 | 14,909,916 | 97.10% | 87.03% | 613 | 121 |
| 5 | PGSP4116 | 21,917,349 | 15,415,573 | 97.92% | 84.65% | 630 | 127 |
| 6 | PGSP4127 | 28,474,255 | 20,526,623 | 98.94% | 85.98% | 625 | 159 |
| 7 | PGSP4665 | 15,627,812 | 9,384,304 | 88.83% | 73.51% | 576 | 79 |
| 8 | PGSP4666 | 10,727,476 | 7,120,332 | 95.76% | 79.44% | 571 | 63 |
| 9 | PGSP4667 | 10,571,919 | 7,054,103 | 97.54% | 79.65% | 581 | 61 |
| 10 | PGSP4668 | 16,091,435 | 10,555,588 | 94.79% | 79.81% | 572 | 88 |
| 11 | PGSP4669 | 15,792,515 | 10,624,189 | 97.13% | 81.15% | 573 | 90 |
| 12 | PGSP4670 | 10,490,170 | 6,951,780 | 95.90% | 80.48% | 575 | 59 |
| 13 | PGSP4671 | 12,377,942 | 8,202,928 | 94.57% | 79.32% | 579 | 68 |
| 14 | PGSP4672 | 11,012,046 | 7,592,367 | 96.91% | 81.75% | 573 | 66 |
| 15 | PGSP4673 | 11,458,989 | 7,903,589 | 97.46% | 82.47% | 554 | 69 |
| 16 | PGSP4674 | 11,282,655 | 7,214,558 | 94.46% | 76.03% | 545 | 62 |
| 17 | PGSP4675 | 15,280,961 | 10,591,544 | 98.85% | 82.77% | 556 | 95 |
| 18 | PGSP4676 | 17,237,309 | 11,087,384 | 98.03% | 77.71% | 569 | 97 |

**O.** Statistics from *sae2Δ* *exo1Δ* chrV:25,817-1,749 gRNA (telomeric to hotspot) uGCR strains

| **No.** | **Sample** | **No. Read Pairs** | **No. Uniquely Mapping Read Pairs** | **% Read 1 Mapped** | **% Read 2 Mapped** | **Median Read Pair Distance (bp)** | **Median Read Depth*** |
| --- | --- | --- | --- | --- | --- | --- | --- |
| 1 | PGSP5086 | 9,976,023 | 7,881,422 | 98.17% | 95.52% | 531 | 61 |
| 2 | PGSP5087 | 9,464,014 | 7,493,820 | 98.87% | 95.87% | 549 | 57 |
| 3 | PGSP5088 | 9,471,665 | 7,529895 | 98.80% | 95.57% | 546 | 59 |
| 4 | PGSP5089 | 12,022,905 | 9,446,326 | 98.63% | 95.74% | 542 | 73 |
| 5 | PGSP5090 | 10,319,341 | 8,505,361 | 98.42% | 95.28% | 567 | 53 |
| 6 | PGSP5091 | 8,451,893 | 6,892,271 | 98.41% | 94.50% | 575 | 54 |
| 7 | PGSP5092 | 9,954,411 | 8,070,147 | 98.65% | 95.67% | 540 | 63 |
| 8 | PGSP5093 | 8,931,358 | 7,204,341 | 99.13% | 96.56% | 543 | 55 |
| 9 | PGSP5094 | 10,570,001 | 8,464,451 | 98.95% | 96.05% | 482 | 66 |
| 10 | PGSP5095 | 8,135,314 | 6,399,416 | 98.59% | 94.36% | 556 | 49 |
| 11 | PGSP5096 | 9,661,366 | 7,869,861 | 99.08% | 96.11% | 563 | 62 |
| 12 | PGSP5097 | 9,685,873 | 7,648,054 | 98.57% | 94.43% | 565 | 60 |

**P.** Statistics from *rrm3Δ* uGCR strains

| **No.** | **Sample** | **No. Read Pairs** | **No. Uniquely Mapping Read Pairs** | **% Read 1 Mapped** | **% Read 2 Mapped** | **Median Read Pair Distance (bp)** | **Median Read Depth*** |
| --- | --- | --- | --- | --- | --- | --- | --- |
| 1 | PGSP420 | 27,949,143 | 20,205,167 | 98.88% | 86.81% | 495 | 170 |
| 2 | PGSP421 | 21,0984,00 | 10,141,311 | 98.20% | 58.75% | 531 | 99 |
| 3 | PGSP422 | 21,542,527 | 11,821,708 | 98.68% | 65.35% | 531 | 113 |
| 4 | PGSP423 | 23,902,429 | 13,313,813 | 98.65% | 68.50% | 514 | 127 |
| 5 | PGSP424 | 23,150,775 | 11,594,166 | 97.47% | 61.06% | 546 | 104 |
| 6 | PGSP425 | 28,129,384 | 15,947,076 | 99.00% | 69.63% | 514 | 145 |
| 7 | PGSP426 | 28,418,609 | 14,068,555 | 98.06% | 60.79% | 524 | 129 |
| 8 | PGSP427 | 29,175,462 | 14,764,445 | 98.50% | 60.14% | 557 | 148 |
| 9 | PGSP4569 | 23,718,178 | 12,952,850 | 98.60% | 66.34% | 507 | 121 |
| 10 | PGSP4570 | 30,079,918 | 14,766,600 | 99.05% | 59.71% | 562 | 142 |
| 11 | PGSP4571 | 28,309,794 | 14,139,200 | 98.91% | 60.54% | 552 | 135 |
| 12 | PGSP4572 | 33,176,724 | 18,255,105 | 99.09% | 69.11% | 523 | 168 |
| 13 | PGSP4573 | 28,908,422 | 17,323,066 | 98.40% | 73.32% | 481 | 156 |
| 14 | PGSP4575 | 22,131,176 | 143,39,311 | 98.70% | 77.69% | 464 | 124 |

**Q.** Statistics from *sae2Δ rrm3Δ* uGCR strains

| **No.** | **Sample** | **No. Read Pairs** | **No. Uniquely Mapping Read Pairs** | **% Read 1 Mapped** | **% Read 2 Mapped** | **Median Read Pair Distance (bp)** | **Median Read Depth*** |
| --- | --- | --- | --- | --- | --- | --- | --- |
| 1 | PGSP4595 | 17,399,554 | 10,723,777 | 95.84% | 74.13% | 508 | 87 |
| 2 | PGSP4596 | 22,538,490 | 14,771,797 | 99.06% | 77.97% | 511 | 119 |
| 3 | PGSP4597 | 22,813,296 | 14,554,168 | 98.17% | 79.61% | 496 | 127 |
| 4 | PGSP4598 | 25,227,698 | 15,268,370 | 94.94% | 73.75% | 518 | 127 |
| 5 | PGSP4599 | 35,407,969 | 22,217,963 | 96.94% | 77.74% | 519 | 200 |
| 6 | PGSP4600 | 31,810,700 | 17,982,839 | 93.70% | 70.55% | 520 | 151 |
| 7 | PGSP4601 | 30,081,933 | 18,037,290 | 97.14% | 74.49% | 541 | 130 |
| 8 | PGSP4602 | 23,011,966 | 12,391,391 | 82.56% | 64.89% | 515 | 88 |
| 9 | PGSP4603 | 27,118,703 | 16,992,757 | 98.78% | 76.59% | 527 | 142 |
| 10 | PGSP4604 | 28,671,968 | 16,194,829 | 93.17% | 70.45% | 538 | 134 |
| 11 | PGSP4605 | 31,949,078 | 20,230,909 | 98.33% | 77.10% | 539 | 170 |
| 12 | PGSP4606 | 34,734,461 | 21,186,686 | 96.90% | 76.13% | 541 | 166 |

**R.** Statistics from *pol32Δ* uGCR strains

| **No.** | **Sample** | **No. Read Pairs** | **No. Uniquely Mapping Read Pairs** | **% Read 1 Mapped** | **% Read 2 Mapped** | **Median Read Pair Distance (bp)** | **Median Read Depth*** |
| --- | --- | --- | --- | --- | --- | --- | --- |
| 1 | PGSP752 | 23,968,909 | 14,86,7831 | 99.22% | 75.70% | 491 | 145 |
| 2 | PGSP754 | 21,530,983 | 14,455,450 | 99.42% | 79.16% | 476 | 135 |
| 3 | PGSP755 | 19,571,487 | 12,321,973 | 98.86% | 75.23% | 506 | 110 |
| 4 | PGSP756 | 25,964,344 | 15,796,664 | 98.87% | 74.07% | 520 | 156 |
| 5 | PGSP757 | 29,342,771 | 17,807,693 | 98.44% | 72.95% | 490 | 171 |
| 6 | PGSP758 | 32,975,826 | 22,211,283 | 99.09% | 78.72% | 478 | 195 |
| 7 | PGSP4564 | 29,943,406 | 18,879,728 | 98.92% | 75.83% | 494 | 173 |
| 8 | PGSP4565 | 34,656,323 | 22,410,066 | 99.43% | 77.02% | 491 | 201 |
| 9 | PGSP4566 | 33,473,465 | 21,272,222 | 99.09% | 76.58% | 493 | 192 |
| 10 | PGSP4567 | 34,571,993 | 22,108,892 | 99.42% | 78.35% | 504 | 200 |
| 12 | PGSP4568 | 27,883,195 | 18,903,615 | 99.11% | 81.62% | 465 | 162 |

**S.** Statistics from *sae2Δ pol32Δ* uGCR strains

| **No.** | **Sample** | **No. Read Pairs** | **No. Uniquely Mapping Read Pairs** | **% Read 1 Mapped** | **% Read 2 Mapped** | **Median Read Pair Distance (bp)** | **Median Read Depth*** |
| --- | --- | --- | --- | --- | --- | --- | --- |
| 1 | PGSP4609 | 32,696,052 | 24,438,283 | 96.55% | 87.52% | 550 | 159 |
| 2 | PGSP4610 | 16,502,876 | 12,458,355 | 97.09% | 86.89% | 575 | 81 |
| 3 | PGSP4611 | 15,565,574 | 11,464,545 | 94.80% | 85.31% | 559 | 67 |
| 4 | PGSP4612 | 18,548,407 | 12,875,103 | 88.82% | 80.05% | 571 | 69 |
| 5 | PGSP4613 | 22,430,743 | 16,491,440 | 95.09% | 85.97% | 560 | 93 |
| 6 | PGSP4614 | 17,607,047 | 12,814,549 | 93.78% | 83.92% | 574 | 77 |
| 7 | PGSP4615 | 22,168,282 | 16,400,529 | 96.52% | 86.69% | 570 | 93 |
| 8 | PGSP4617 | 29,076,892 | 19,512,011 | 87.70% | 77.84% | 568 | 122 |
| 9 | PGSP4618 | 18,759,539 | 13,287,797 | 92.91% | 81.87% | 552 | 86 |
| 10 | PGSP4619 | 25,041,204 | 18,263,530 | 96.13% | 86.20% | 557 | 122 |
| 11 | PGSP4620 | 18,550,883 | 13,429,847 | 96.94% | 84.31% | 562 | 98 |
| 12 | PGSP4621 | 14,497,309 | 10,906,029 | 95.99% | 85.81% | 560 | 73 |
| 13 | PGSP4622 | 15,097,066 | 11,148,542 | 97.33% | 85.53% | 570 | 66 |
| 14 | PGSP4623 | 18,521,474 | 14,268,625 | 97.79% | 88.34% | 556 | 92 |
| 15 | PGSP4624 | 20,670,585 | 15,316,641 | 95.39% | 85.83% | 553 | 93 |

**T.** Statistics from *rad10Δ* uGCR strains

| **No.** | **Sample** | **No. Read Pairs** | **No. Uniquely Mapping Read Pairs** | **% Read 1 Mapped** | **% Read 2 Mapped** | **Median Read Pair Distance (bp)** | **Median Read Depth*** |
| --- | --- | --- | --- | --- | --- | --- | --- |
| 1 | PGSP461 | 23,490,104 | 16,399,977 | 97.74% | 80.83% | 421 | 161 |
| 2 | PGSP462 | 21,784,901 | 13,226,274 | 98.86% | 73.57% | 509 | 133 |
| 3 | PGSP463 | 19,467,789 | 12,376,867 | 98.73% | 74.66% | 518 | 115 |
| 4 | PGSP464 | 19,654,719 | 11,246,945 | 96.86% | 66.35% | 554 | 107 |
| 5 | PGSP465 | 27,327,564 | 16,398,875 | 98.37% | 72.33% | 532 | 165 |
| 6 | PGSP466 | 29,288,732 | 17,109,074 | 97.31% | 70.20% | 510 | 162 |
| 7 | PGSP467 | 29,053,166 | 17,179,753 | 97.35% | 70.79% | 524 | 173 |
| 8 | PGSP4576 | 28,367,850 | 16,670,383 | 98.55% | 69.85% | 534 | 150 |
| 9 | PGSP4577 | 35,140,020 | 22,037,900 | 98.56% | 73.30% | 512 | 201 |
| 10 | PGSP4578 | 31,541,216 | 19,418,967 | 98.64% | 73.04% | 515 | 172 |
| 11 | PGSP4579 | 34,145,506 | 21,532,910 | 98.48% | 74.78% | 526 | 197 |
| 12 | PGSP4580 | 24,733,670 | 14,750,897 | 96.48% | 71.43% | 539 | 139 |

**U.** Statistics from *sae2Δ rad10Δ* uGCR strains

| **No.** | **Sample** | **No. Read Pairs** | **No. Uniquely Mapping Read Pairs** | **% Read 1 Mapped** | **% Read 2 Mapped** | **Median Read Pair Distance (bp)** | **Median Read Depth*** |
| --- | --- | --- | --- | --- | --- | --- | --- |
| 1 | PGSP4581 | 25,528,596 | 15,746,634 | 95.37% | 74.78% | 473 | 139 |
| 2 | PGSP4582 | 24,166,390 | 14,845,361 | 96.87% | 74.54% | 508 | 133 |
| 3 | PGSP4583 | 21,606,266 | 13,704,809 | 98.45% | 76.39% | 515 | 124 |
| 4 | PGSP4584 | 20,665,941 | 11,184,645 | 87.60% | 65.72% | 517 | 109 |
| 5 | PGSP4585 | 25,040,166 | 15,945,410 | 96.89% | 76.56% | 509 | 159 |
| 6 | PGSP4586 | 26,508,540 | 15,356,456 | 97.64% | 71.71% | 513 | 148 |
| 7 | PGSP4587 | 34,929,938 | 19,132,285 | 85.92% | 65.66% | 516 | 179 |
| 8 | PGSP4588 | 27,501,339 | 16,342,943 | 94.91% | 71.93% | 519 | 151 |
| 9 | PGSP4589 | 33,305,906 | 20,139,460 | 98.40% | 75.80% | 510 | 192 |
| 10 | PGSP4590 | 31,560,337 | 18,106,293 | 95.06% | 70.04% | 534 | 166 |
| 11 | PGSP4591 | 28,096,591 | 17,230,038 | 97.39% | 75.22% | 533 | 153 |
| 12 | PGSP4592 | 25,130,218 | 15,929,713 | 97.08% | 76.17% | 521 | 147 |

**V.** Statistics from *mus81Δ* uGCR strains

| **No.** | **Sample** | **No. Read Pairs** | **No. Uniquely Mapping Read Pairs** | **% Read 1 Mapped** | **% Read 2 Mapped** | **Median Read Pair Distance (bp)** | **Median Read Depth**** |
| --- | --- | --- | --- | --- | --- | --- | --- |
| 1 | PGSP222 | 33,118,676 | 21,716,550 | 89.60% | 79.63% | 543 | 172 |
| 2 | PGSP223 | 19,540,855 | 14,002,823 | 99.18% | 86.73% | 577 | 152 |
| 3 | PGSP224 | 23,558,682 | 15,642,835 | 96.57% | 85.33% | 543 | 136 |
| 4 | PGSP225 | 21,156,814 | 15,613,162 | 98.69% | 88.59% | 553 | 144 |
| 5 | PGSP226 | 32,475,548 | 22,392,109 | 97.17% | 86.11% | 557 | 258 |
| 6 | PGSP227 | 24,435,836 | 17,444,073 | 95.88% | 84.98% | 560 | 140 |
| 7 | PGSP228 | 28,097,918 | 20,124,570 | 97.89% | 85.89% | 573 | 189 |
| 8 | PGSP229 | 28,195,913 | 19,578,634 | 97.35% | 87.24% | 547 | 181 |
| 9 | PGSP230 | 25,063,453 | 17,016,029 | 94.95% | 84.59% | 539 | 160 |
| 10 | PGSP231 | 27,317,992 | 18,086,489 | 96.78% | 82.12% | 564 | 166 |
| 11 | PGSP232 | 33,230,952 | 23,109,864 | 98.72% | 85.41% | 582 | 198 |
| 12 | PGSP233 | 30,799,632 | 20,548,946 | 98.06% | 82.47% | 575 | 184 |

**W.** Statistics from *sae2Δ mus81Δ* uGCR strains

| **No.** | **Sample** | **No. Read Pairs** | **No. Uniquely Mapping Read Pairs** | **% Read 1 Mapped** | **% Read 2 Mapped** | **Median Read Pair Distance (bp)** | **Median Read Depth*** |
| --- | --- | --- | --- | --- | --- | --- | --- |
| 1 | PGSP4649 | 9,408,028 | 4,457,804 | 84.03% | 54.40% | 544 | 40 |
| 2 | PGSP4650 | 11,143,121 | 5,957,614 | 95.38% | 61.29% | 552 | 56 |
| 3 | PGSP4651 | 15,094,668 | 7,792,273 | 93.48% | 59.59% | 540 | 66 |
| 4 | PGSP4652 | 20,572,978 | 10,357,758 | 88.39% | 58.13% | 541 | 84 |
| 5 | PGSP4653 | 19,289,410 | 9,955,130 | 87.77% | 59.15% | 524 | 87 |
| 6 | PGSP4654 | 13,080,889 | 6,915,873 | 91.87% | 61.20% | 540 | 58 |
| 7 | PGSP4655 | 18,240,029 | 9,537,444 | 94.05% | 60.05% | 561 | 88 |
| 8 | PGSP4656 | 17,869,922 | 8,996,928 | 95.11% | 59.67% | 561 | 84 |
| 9 | PGSP4657 | 19,206,754 | 9,702,474 | 89.71% | 58.45% | 546 | 83 |
| 10 | PGSP4658 | 14,964,335 | 6,136,634 | 82.81% | 47.70% | 541 | 56 |
| 11 | PGSP4659 | 17,973,456 | 9,341,979 | 94.29% | 60.41% | 539 | 80 |
| 12 | PGSP4660 | 14,346,803 | 6,612,177 | 93.49% | 53.26% | 558 | 66 |
| 13 | PGSP4661 | 7,723,560 | 3,614,008 | 89.89% | 54.15% | 544 | 33 |
| 14 | PGSP4662 | 13,584,744 | 7,025,241 | 94.28% | 59.99% | 538 | 60 |
| 15 | PGSP4663 | 9,926,317 | 5,399,375 | 96.59% | 63.65% | 545 | 41 |
| 16 | PGSP4664 | 10,626,187 | 5,401,601 | 93.81% | 58.67% | 558 | 45 |

**X.** Statistics from *sae2Δ mus81Δ* chrV:25,817-1,749 gRNA uGCR strains

| **No.** | **Sample** | **No. Read Pairs** | **No. Uniquely Mapping Read Pairs** | **% Read 1 Mapped** | **% Read 2 Mapped** | **Median Read Pair Distance (bp)** | **Median Read Depth*** |
| --- | --- | --- | --- | --- | --- | --- | --- |
| 1 | PGSP5074 | 10,250,449 | 8,243,418 | 98.38% | 95.42% | 544 | 69 |
| 2 | PGSP5075 | 7,456,785 | 6,129,309 | 98.75% | 96.22% | 526 | 50 |
| 3 | PGSP5076 | 10,560,899 | 8,528,733 | 99.21% | 94.98% | 551 | 70 |
| 4 | PGSP5077 | 9,299,209 | 6,757,868 | 88.75% | 85.20% | 540 | 55 |
| 5 | PGSP5078 | 10,507,726 | 8,294,259 | 93.81% | 91.42% | 541 | 61 |
| 6 | PGSP5079 | 5,974,284 | 4,455,340 | 89.86% | 86.99% | 525 | 36 |
| 7 | PGSP5080 | 11,102,164 | 8,493,521 | 92.69% | 90.26% | 532 | 69 |
| 8 | PGSP5081 | 11,951,286 | 8,978,803 | 92.86% | 89.93% | 527 | 71 |
| 9 | PGSP5082 | 11,838,150 | 8,877,250 | 91.37% | 88.09% | 551 | 71 |
| 10 | PGSP5083 | 9,278,695 | 70,33,211 | 89.69% | 87.46% | 538 | 53 |
| 11 | PGSP5084 | 9,108,055 | 7,376,707 | 98.91% | 94.98% | 589 | 58 |
| 12 | PGSP5085 | 9,885,977 | 7,951,757 | 96.29% | 93.24% | 573 | 58 |

**Y.** Statistics from *slx1Δ* uGCR strains

| **No.** | **Sample** | **No. Read Pairs** | **No. Uniquely Mapping Read Pairs** | **% Read 1 Mapped** | **% Read 2 Mapped** | **Median Read Pair Distance (bp)** | **Median Read Depth*** |
| --- | --- | --- | --- | --- | --- | --- | --- |
| 1 | PGSP4904 | 6,095,628 | 3,712,232 | 87.24% | 70.47% | 623 | 33 |
| 2 | PGSP4905 | 5,011,998 | 3,168,347 | 90.22% | 74.67% | 601 | 29 |
| 3 | PGSP4907 | 4,916,546 | 2,999,753 | 90.57% | 71.16% | 648 | 26 |
| 4 | PGSP4908 | 7,104,028 | 4,373,815 | 87.34% | 71.95% | 622 | 38 |
| 5 | PGSP4909 | 6,843,990 | 4,343,986 | 91.94% | 74.37% | 625 | 38 |
| 6 | PGSP4910 | 5,074,448 | 3,254,306 | 90.49% | 74.72% | 619 | 27 |
| 7 | PGSP4912 | 6,031,962 | 3,788,900 | 88.41% | 73.49% | 609 | 32 |
| 8 | PGSP4913 | 4,900,742 | 3,223,123 | 94.15% | 77.00% | 626 | 28 |
| 9 | PGSP4914 | 6,095,851 | 3,759,455 | 87.78% | 72.08% | 612 | 32 |
| 10 | PGSP4915 | 5,164,055 | 2,927,405 | 82.82% | 66.82% | 604 | 26 |
| 11 | PGSP4916 | 7,756,840 | 4,562,306 | 84.31% | 69.19% | 603 | 40 |
| 12 | PGSP4917 | 6,491,194 | 3,949,798 | 89.54% | 71.46% | 619 | 34 |

**Z.** Statistics from *sae2Δ slx1Δ* uGCR strains

| **No.** | **Sample** | **No. Read Pairs** | **No. Uniquely Mapping Read Pairs** | **% Read 1 Mapped** | **% Read 2 Mapped** | **Median Read Pair Distance (bp)** | **Median Read Depth**** |
| --- | --- | --- | --- | --- | --- | --- | --- |
| 1 | PGSP4864 | 9,496,937 | 6,472,620 | 97.34% | 84.12% | 616 | 58 |
| 2 | PGSP4865 | 9,852,419 | 6,837,116 | 98.72% | 84.96% | 609 | 61 |
| 3 | PGSP4866 | 8,984,624 | 6,232,866 | 98.88% | 85.66% | 612 | 55 |
| 4 | PGSP4867 | 7,948,210 | 5,376,325 | 96.70% | 82.63% | 623 | 50 |
| 5 | PGSP4869 | 11,333,858 | 7,857,040 | 97.86% | 85.19% | 616 | 70 |
| 6 | PGSP4870 | 15,330,473 | 9,978,921 | 98.50% | 80.15% | 655 | 91 |
| 7 | PGSP4871 | 11,805,332 | 7,816,737 | 97.64% | 81.15% | 665 | 71 |
| 8 | PGSP4872 | 10,685,646 | 7,080,338 | 97.92% | 82.00% | 636 | 63 |
| 9 | PGSP4873 | 12,410,776 | 8,202,912 | 98.03% | 80.81% | 655 | 76 |
| 10 | PGSP4874 | 11,759,700 | 7,835,592 | 97.85% | 82.62% | 637 | 70 |
| 11 | PGSP4875 | 11,202,438 | 7,732,728 | 98.55% | 85.45% | 609 | 71 |
| 12 | PGSP4876 | 9,640,196 | 6,151,492 | 95.65% | 82.95% | 612 | 55 |

**AA.** Statistics from *yen1Δ* uGCR strains

| **No.** | **Sample** | **No. Read Pairs** | **No. Uniquely Mapping Read Pairs** | **% Read 1 Mapped** | **% Read 2 Mapped** | **Median Read Pair Distance (bp)** | **Median Read Depth*** |
| --- | --- | --- | --- | --- | --- | --- | --- |
| 1 | PGSP4891 | 12,669,334 | 8,780,924 | 94.06% | 83.59% | 576 | 76 |
| 2 | PGSP4892 | 9,708,207 | 6,774,961 | 92.44% | 83.62% | 554 | 58 |
| 3 | PGSP4893 | 10,378,759 | 7,767,988 | 97.59% | 89.09% | 528 | 66 |
| 4 | PGSP4894 | 14,071,332 | 10,499,073 | 97.19% | 89.09% | 525 | 90 |
| 5 | PGSP4895 | 18,270,982 | 13,941,153 | 97.63% | 92.54% | 439 | 120 |
| 6 | PGSP4896 | 9,755,767 | 7,422,788 | 96.89% | 91.57% | 458 | 67 |
| 7 | PGSP4897 | 11,609,812 | 8,827,896 | 97.36% | 91.73% | 457 | 75 |
| 8 | PGSP4898 | 10,918,632 | 8,507,751 | 98.06% | 93.45% | 440 | 70 |
| 9 | PGSP4899 | 13,959,516 | 10,656,668 | 98.16% | 92.30% | 463 | 87 |
| 10 | PGSP4900 | 9,730,505 | 7,405,263 | 97.65% | 91.33% | 461 | 64 |
| 11 | PGSP4901 | 13,014,988 | 9,525,355 | 98.14% | 88.22% | 582 | 83 |

**AB.** Statistics from *sae2Δ yen1Δ* uGCR strains

| **No.** | **Sample** | **No. Read Pairs** | **No. Uniquely Mapping Read Pairs** | **% Read 1 Mapped** | **% Read 2 Mapped** | **Median Read Pair Distance (bp)** | **Median Read Depth*** |
| --- | --- | --- | --- | --- | --- | --- | --- |
| 1 | PGSP4849 | 12,187,719 | 8,436,231 | 94.88% | 84.48% | 600 | 70 |
| 2 | PGSP4850 | 9,638,742 | 6,955,109 | 96.65% | 87.32% | 560 | 55 |
| 3 | PGSP4851 | 7,780,531 | 5,698,991 | 98.72% | 89.73% | 549 | 47 |
| 4 | PGSP4852 | 6,758,365 | 4,993,097 | 96.70% | 89.05% | 500 | 42 |
| 5 | PGSP4853 | 24,208,304 | 18,045,356 | 98.36% | 91.29% | 506 | 153 |
| 6 | PGSP4854 | 13,010,706 | 9,477,765 | 97.09% | 88.71% | 502 | 80 |
| 7 | PGSP4855 | 17,494,838 | 13,224,498 | 98.19% | 91.78% | 487 | 108 |
| 8 | PGSP4856 | 11,951,467 | 8,695,845 | 96.85% | 88.65% | 516 | 72 |
| 9 | PGSP4857 | 14,230,838 | 10,767,178 | 97.77% | 92.29% | 454 | 91 |
| 10 | PGSP4858 | 14,179,239 | 10,671,325 | 97.31% | 91.94% | 461 | 89 |
| 11 | PGSP4859 | 15,300,354 | 11,385,182 | 98.51% | 92.44% | 475 | 91 |
| 12 | PGSP4860 | 10,373,416 | 7,338,770 | 95.86% | 86.34% | 590 | 63 |

**AC.** Statistics from *pif1Δ* uGCR strains

| **No.** | **Sample** | **No. Read Pairs** | **No. Uniquely Mapping Read Pairs** | **% Read 1 Mapped** | **% Read 2 Mapped** | **Median Read Pair Distance (bp)** | **Median Read Depth*** |
| --- | --- | --- | --- | --- | --- | --- | --- |
| 1 | PGSP2214 | 12,679,488 | 7,992,741 | 96.87% | 75.24% | 554 | 80 |
| 2 | PGSP2215 | 11,464,219 | 7,532,466 | 97.82% | 78.11% | 539 | 74 |
| 3 | PGSP2216 | 9,801,625 | 6,221,287 | 98.41% | 76.18% | 544 | 61 |
| 4 | PGSP2217 | 15,002,240 | 9,302,195 | 97.98% | 74.37% | 570 | 94 |
| 5 | PGSP2218 | 15,406,316 | 10,260,362 | 98.83% | 80.18% | 529 | 100 |
| 6 | PGSP2219 | 10,462,889 | 6,869,122 | 98.50% | 78.07% | 540 | 67 |
| 7 | PGSP2220 | 11,402,565 | 7,733,204 | 98.00% | 80.18% | 531 | 75 |
| 8 | PGSP2221 | 15,112,130 | 10,143,212 | 99.07% | 80.64% | 531 | 99 |
| 9 | PGSP2222 | 10,757,358 | 6,674,924 | 97.59% | 74.35% | 549 | 68 |
| 10 | PGSP2223 | 13,606,988 | 8,227,335 | 98.63% | 71.81% | 510 | 84 |
| 11 | PGSP2224 | 15,222,465 | 9,882,041 | 97.89% | 77.07% | 548 | 93 |
| 12 | PGSP2225 | 13,175,854 | 7,606,019 | 98.91% | 68.15% | 533 | 79 |

**AD.** Statistics from *sae2Δ pif1Δ* uGCR strains

| **No.** | **Sample** | **No. Read Pairs** | **No. Uniquely Mapping Read Pairs** | **% Read 1 Mapped** | **% Read 2 Mapped** | **Median Read Pair Distance (bp)** | **Median Read Depth*** |
| --- | --- | --- | --- | --- | --- | --- | --- |
| 1 | PGSP4703 | 8,170,848 | 3,902,530 | 91.20% | 59.40% | 556 | 41 |
| 2 | PGSP4704 | 10,283,481 | 5,923,179 | 96.01% | 69.52% | 576 | 59 |
| 3 | PGSP4705 | 9,988,691 | 5,586,743 | 97.63% | 73.42% | 564 | 51 |
| 4 | PGSP4706 | 9,857,043 | 5,357,267 | 94.40% | 69.65% | 568 | 50 |
| 5 | PGSP4707 | 16,163,240 | 9,360,422 | 95.48% | 72.55% | 572 | 87 |
| 6 | PGSP4709 | 14,375,932 | 8,319,106 | 95.12% | 70.64% | 557 | 82 |
| 7 | PGSP4710 | 13,270,293 | 7,718,515 | 95.17% | 71.63% | 574 | 73 |
| 8 | PGSP4711 | 12,273,873 | 6,846,250 | 94.35% | 68.93% | 577 | 67 |
| 9 | PGSP4713 | 13,109,194 | 7,681,145 | 94.76% | 72.37% | 556 | 75 |
| 10 | PGSP4714 | 13,281,326 | 7,664,286 | 94.68% | 71.55% | 571 | 75 |
| 11 | PGSP4715 | 13,677,128 | 8,278,184 | 97.33% | 75.52% | 572 | 79 |
| 12 | PGSP4716 | 11,130,169 | 6,764,227 | 95.42% | 74.67% | 565 | 63 |

**AE.** Statistics from *yku80Δ* uGCR strains

| **No.** | **Sample** | **No. Read Pairs** | **No. Uniquely Mapping Read Pairs** | **% Read 1 Mapped** | **% Read 2 Mapped** | **Median Read Pair Distance (bp)** | **Median Read Depth*** |
| --- | --- | --- | --- | --- | --- | --- | --- |
| 1 | PGSP3606 | 12,595,378 | 7,259,434 | 86.14% | 67.99% | 574 | 57 |
| 2 | PGSP3607 | 12,412,631 | 7,965,253 | 96.46% | 76.76% | 595 | 66 |
| 3 | PGSP3608 | 11,340,298 | 7,763,015 | 98.09% | 81.52% | 568 | 64 |
| 4 | PGSP3609 | 97,78,436 | 6,586,747 | 97.80% | 80.16% | 572 | 56 |
| 5 | PGSP3610 | 14,860,895 | 9,912,997 | 94.49% | 80.97% | 560 | 76 |
| 6 | PGSP3611 | 14,927,102 | 9,562,478 | 96.94% | 77.75% | 567 | 77 |
| 7 | PGSP3612 | 15,465,662 | 10,486,131 | 98.55% | 84.08% | 551 | 85 |
| 8 | PGSP3613 | 14,485,202 | 9,442,685 | 96.31% | 79.33% | 570 | 75 |
| 9 | PGSP3614 | 15,357,037 | 10,661,187 | 99.05% | 82.37% | 569 | 93 |
| 10 | PGSP3615 | 16,947,674 | 11,361,631 | 97.57% | 80.82% | 586 | 89 |
| 11 | PGSP3616 | 18,917,468 | 13,186,429 | 97.74% | 83.81% | 567 | 97 |
| 12 | PGSP3617 | 14,110,801 | 9,800,000 | 96.84% | 82.91% | 576 | 69 |

**AF.** Statistics from *exo1Δ* *yku80Δ* uGCR strains

| **No.** | **Sample** | **No. Read Pairs** | **No. Uniquely Mapping Read Pairs** | **% Read 1 Mapped** | **% Read 2 Mapped** | **Median Read Pair Distance (bp)** | **Median Read Depth*** |
| --- | --- | --- | --- | --- | --- | --- | --- |
| 1 | PGSP5060 | 6,134,933 | 3,636,134 | 94.92% | 67.72% | 548 | 33 |
| 2 | PGSP5061 | 5,394,008 | 3,201,407 | 96.75% | 69.38% | 564 | 25 |
| 3 | PGSP5062 | 6,230,580 | 4,279,069 | 97.66% | 78.72% | 552 | 35 |
| 4 | PGSP5063 | 8,601,643 | 5,376,670 | 91.28% | 72.37% | 526 | 39 |
| 5 | PGSP5064 | 8,142,472 | 5,577,886 | 97.82% | 79.92% | 541 | 53 |
| 6 | PGSP5065 | 6,536,665 | 4,440,925 | 93.65% | 78.94% | 547 | 39 |
| 7 | PGSP5066 | 6,736,616 | 4,123,228 | 93.27% | 70.47% | 544 | 36 |
| 8 | PGSP5067 | 5,642,311 | 3,742,614 | 97.36% | 77.81% | 544 | 28 |
| 9 | PGSP5068 | 5,639,059 | 2,950,975 | 92.07% | 60.45% | 536 | 30 |
| 10 | PGSP5069 | 5,838,216 | 3,824,464 | 87.86% | 75.29% | 540 | 27 |
| 11 | PGSP5070 | 6,459,874 | 4,378,468 | 94.83% | 78.40% | 533 | 37 |
| 12 | PGSP5071 | 6,569,698 | 4,399,199 | 94.90% | 78.04% | 534 | 40 |

**AG.** Statistics from *pif1Δ* *yku80Δ* uGCR strains

| **No.** | **Sample** | **No. Read Pairs** | **No. Uniquely Mapping Read Pairs** | **% Read 1 Mapped** | **% Read 2 Mapped** | **Median Read Pair Distance (bp)** | **Median Read Depth*** |
| --- | --- | --- | --- | --- | --- | --- | --- |
| 1 | PGSP4932 | 6,532,120 | 4,018,160 | 90.17% | 72.52% | 621 | 36 |
| 2 | PGSP4933 | 5,618,951 | 3,679,709 | 94.06% | 76.77% | 618 | 35 |
| 3 | PGSP4934 | 5,272,832 | 3,440,231 | 95.73% | 76.96% | 622 | 34 |
| 4 | PGSP4935 | 6,876,112 | 4,454,899 | 93.03% | 76.47% | 611 | 42 |
| 5 | PGSP4936 | 7,881,382 | 5,290,059 | 95.69% | 79.77% | 582 | 52 |
| 6 | PGSP4937 | 5,576,120 | 3,565,859 | 91.27% | 75.84% | 595 | 34 |
| 7 | PGSP4938 | 5,623,483 | 3,750,024 | 93.71% | 78.09% | 584 | 36 |
| 8 | PGSP4939 | 4,970,980 | 3,097,327 | 88.78% | 72.92% | 592 | 28 |
| 9 | PGSP4940 | 5,592,974 | 3,499,665 | 90.94% | 72.88% | 631 | 24 |
| 10 | PGSP4941 | 5,929,764 | 3,400,356 | 86.34% | 67.67% | 611 | 30 |
| 11 | PGSP4942 | 7,316,808 | 4,744,465 | 91.59% | 75.85% | 591 | 44 |
| 12 | PGSP4943 | 8,167,373 | 4,929,790 | 90.15% | 70.95% | 615 | 40 |

**AH.** Statistics from *sae2Δ yku80Δ* uGCR strains

| **No.** | **Sample** | **No. Read Pairs** | **No. Uniquely Mapping Read Pairs** | **% Read 1 Mapped** | **% Read 2 Mapped** | **Median Read Pair Distance (bp)** | **Median Read Depth*** |
| --- | --- | --- | --- | --- | --- | --- | --- |
| 1 | PGSP4677 | 13,270,860 | 8,821,769 | 98.78% | 83.77% | 586 | 78 |
| 2 | PGSP4678 | 13,064,584 | 8,480,809 | 98.30% | 83.68% | 575 | 73 |
| 3 | PGSP4679 | 11,604,935 | 7,808,882 | 98.47% | 83.22% | 600 | 68 |
| 4 | PGSP4680 | 9,662,510 | 6,413,323 | 98.33% | 82.12% | 611 | 56 |
| 5 | PGSP4682 | 14,983,516 | 10,090,057 | 98.66% | 86.17% | 570 | 80 |
| 6 | PGSP4684 | 13,680,715 | 8,858,847 | 98.53% | 81.43% | 591 | 82 |
| 7 | PGSP4685 | 15,492,169 | 10,371,104 | 98.72% | 86.57% | 575 | 89 |
| 8 | PGSP4686 | 14,905,843 | 9,890,777 | 98.49% | 82.91% | 603 | 89 |
| 9 | PGSP4687 | 15,876,016 | 10,583,410 | 98.85% | 84.27% | 594 | 93 |
| 10 | PGSP4688 | 12,752,446 | 8,700,851 | 98.51% | 86.98% | 572 | 70 |
| 11 | PGSP4689 | 15,583,809 | 10,703,806 | 98.61% | 87.21% | 574 | 96 |
| 12 | PGSP4690 | 15,214,256 | 10,446,458 | 98.88% | 86.68% | 588 | 90 |

**AI.** Statistics from *sae2Δ pif1Δ yku80Δ* uGCR strains

| **No.** | **Sample** | **No. Read Pairs** | **No. Uniquely Mapping Read Pairs** | **% Read 1 Mapped** | **% Read 2 Mapped** | **Median Read Pair Distance (bp)** | **Median Read Depth*** |
| --- | --- | --- | --- | --- | --- | --- | --- |
| 1 | PGSP4717 | 12,530,481 | 8,052,102 | 96.97% | 74.61% | 551 | 60 |
| 2 | PGSP4718 | 8,639,301 | 5,591,509 | 98.30% | 75.29% | 559 | 55 |
| 3 | PGSP4719 | 8,740,618 | 5,333,414 | 98.44% | 70.47% | 582 | 42 |
| 4 | PGSP4720 | 11,481,669 | 7,324,149 | 96.76% | 73.79% | 575 | 52 |
| 5 | PGSP4721 | 13,171,367 | 7,993,785 | 98.69% | 70.17% | 603 | 84 |
| 6 | PGSP4722 | 9,822,158 | 5,896,954 | 97.19% | 70.24% | 590 | 60 |
| 7 | PGSP4723 | 10,260,656 | 6,352,925 | 96.02% | 72.65% | 570 | 64 |
| 8 | PGSP4724 | 8,978,545 | 5,536,685 | 96.13% | 71.91% | 581 | 40 |
| 9 | PGSP4725 | 10,776,756 | 6,387,823 | 96.36% | 69.98% | 576 | 65 |
| 10 | PGSP4726 | 11,465,510 | 5,828,390 | 93.47% | 59.71% | 547 | 53 |
| 11 | PGSP4727 | 13,132,408 | 8,355,618 | 96.08% | 73.99% | 553 | 62 |
| 12 | PGSP4728 | 12,819,623 | 7,320,691 | 97.56% | 66.47% | 534 | 61 |

**AJ.** Statistics from *sgs1Δ* uGCR strains

| **No.** | **Sample** | **No. Read Pairs** | **No. Uniquely Mapping Read Pairs** | **% Read 1 Mapped** | **% Read 2 Mapped** | **Median Read Pair Distance (bp)** | **Median Read Depth*** |
| --- | --- | --- | --- | --- | --- | --- | --- |
| 1 | PGSP937 | 9,223,680 | 5,468,518 | 98.55% | 68.50% | 534 | 55 |
| 2 | PGSP939 | 9,642,613 | 6,080,852 | 99.11% | 74.79% | 559 | 59 |
| 3 | PGSP940 | 6,981,958 | 4,413,284 | 98.68% | 73.53% | 566 | 40 |
| 4 | PGSP941 | 13,096,674 | 8,638,133 | 98.13% | 77.49% | 558 | 81 |
| 5 | PGSP942 | 11,060,270 | 6,911,061 | 98.91% | 73.49% | 550 | 66 |
| 6 | PGSP943 | 13,901,592 | 8,939,130 | 97.28% | 75.91% | 562 | 84 |
| 7 | PGSP944 | 15,805,985 | 9,869,248 | 97.41% | 72.40% | 572 | 93 |
| 8 | PGSP945 | 16,694,897 | 10,578,884 | 99.04% | 75.13% | 566 | 94 |
| 9 | PGSP946 | 12,452,900 | 8,016,418 | 97.83% | 75.94% | 575 | 77 |
| 10 | PGSP948 | 14,020,301 | 9,238,943 | 98.60% | 78.00% | 571 | 87 |
| 11 | PGSP949 | 10,290,294 | 6,704,084 | 98.65% | 77.44% | 573 | 62 |

**AK.** Statistics from *exo1Δ* *sgs1Δ* uGCR strains

| **No.** | **Sample** | **No. Read Pairs** | **No. Uniquely Mapping Read Pairs** | **% Read 1 Mapped** | **% Read 2 Mapped** | **Median Read Pair Distance (bp)** | **Median Read Depth*** |
| --- | --- | --- | --- | --- | --- | --- | --- |
| 1 | PGSP4350 | 10,652,122 | 5,408,814 | 93.17% | 62.46% | 560 | 53 |
| 2 | PGSP4351 | 18,207,107 | 10,796,569 | 97.14% | 70.26% | 583 | 102 |
| 3 | PGSP4352 | 9,004,164 | 5,441,547 | 98.34% | 71.00% | 588 | 51 |
| 4 | PGSP4353 | 8,446,095 | 4,944,936 | 94.49% | 70.17% | 573 | 43 |
| 5 | PGSP4354 | 13,326,897 | 8,404,652 | 96.66% | 75.00% | 565 | 69 |
| 6 | PGSP4356 | 12,765,169 | 7,613,044 | 96.93% | 70.14% | 577 | 69 |
| 7 | PGSP4357 | 13,665,295 | 8,388,224 | 98.37% | 73.97% | 583 | 77 |
| 8 | PGSP4358 | 12,545,287 | 7,558,574 | 97.35% | 70.89% | 586 | 70 |
| 9 | PGSP4359 | 12,809,536 | 7,847,265 | 98.33% | 73.75% | 572 | 68 |
| 10 | PGSP4360 | 11,743,868 | 7,324,744 | 97.86% | 73.85% | 577 | 66 |
| 11 | PGSP4361 | 14,164,951 | 9,191,156 | 98.41% | 78.22% | 559 | 80 |
| 12 | PGSP4362 | 10,690,200 | 6,702,969 | 96.48% | 74.49% | 578 | 60 |

**AL.** Statistics from *sae2Δ* *exo1Δ* *yku80Δ* uGCR strains

| **No.** | **Sample** | **No. Read Pairs** | **No. Uniquely Mapping Read Pairs** | **% Read 1 Mapped** | **% Read 2 Mapped** | **Median Read Pair Distance (bp)** | **Median Read Depth*** |
| --- | --- | --- | --- | --- | --- | --- | --- |
| 1 | PGSP5046 | 8,865,312 | 6,299,581 | 93.47% | 82.20% | 551 | 46 |
| 2 | PGSP5047 | 5,297,182 | 3,533,008 | 95.27% | 76.85% | 530 | 29 |
| 3 | PGSP5048 | 6,131,429 | 3,755,543 | 98.76% | 70.63% | 536 | 32 |
| 4 | PGSP5049 | 5,470,029 | 3,556,560 | 95.39% | 74.58% | 523 | 32 |
| 5 | PGSP5050 | 6,915,443 | 4,797,584 | 95.97% | 80.14% | 527 | 36 |
| 6 | PGSP5051 | 3,864,171 | 2,358,421 | 93.98% | 69.84% | 515 | 21 |
| 7 | PGSP5052 | 7,786,448 | 5,021,087 | 91.80% | 74.73% | 537 | 41 |
| 8 | PGSP5053 | 7,981,284 | 4,783,539 | 95.62% | 69.04% | 529 | 39 |
| 9 | PGSP5054 | 9,054,122 | 5,722,194 | 94.96% | 73.26% | 525 | 47 |
| 10 | PGSP5055 | 6,824,011 | 4,639,646 | 94.01% | 79.05% | 536 | 36 |
| 11 | PGSP5058 | 6,541,357 | 4,452,844 | 98.54% | 78.26% | 537 | 37 |
| 12 | PGSP5059 | 6,889,979 | 4,919,113 | 97.39% | 83.09% | 544 | 40 |

**AM.** Statistics from *sgs1Δ yku80Δ* uGCR strains

| **No.** | **Sample** | **No. Read Pairs** | **No. Uniquely Mapping Read Pairs** | **% Read 1 Mapped** | **% Read 2 Mapped** | **Median Read Pair Distance (bp)** | **Median Read Depth*** |
| --- | --- | --- | --- | --- | --- | --- | --- |
| 1 | PGSP4691 | 13,962,795 | 9,508,721 | 98.56% | 83.16% | 605 | 79 |
| 2 | PGSP4692 | 11,802,443 | 7,823,566 | 98.77% | 85.65% | 586 | 64 |
| 3 | PGSP4693 | 10,208,738 | 7,011,857 | 98.87% | 85.06% | 589 | 63 |
| 4 | PGSP4694 | 14,950,248 | 10,408,293 | 98.48% | 85.16% | 590 | 91 |
| 5 | PGSP4695 | 16,025,248 | 11,018,036 | 99.09% | 85.29% | 591 | 95 |
| 6 | PGSP4696 | 11,943,487 | 8,287,003 | 99.14% | 85.65% | 597 | 73 |
| 7 | PGSP4697 | 13,003,755 | 8,979,681 | 98.95% | 84.60% | 602 | 80 |
| 8 | PGSP4698 | 12,326,211 | 8,715,081 | 98.79% | 85.69% | 597 | 71 |
| 9 | PGSP4699 | 13,466,775 | 9,428,427 | 98.72% | 83.90% | 591 | 79 |
| 10 | PGSP4700 | 13,733,125 | 9,402,571 | 98.90% | 82.46% | 600 | 79 |
| 11 | PGSP4701 | 15,340,463 | 10,792,111 | 98.69% | 84.84% | 594 | 94 |
| 12 | PGSP4702 | 16,864,043 | 11,607,943 | 98.93% | 82.97% | 606 | 106 |

**AN.** Statistics from *sae2Δ sgs1Δ yku80Δ* uGCR strains

| **No.** | **Sample** | **No. Read Pairs** | **No. Uniquely Mapping Read Pairs** | **% Read 1 Mapped** | **% Read 2 Mapped** | **Median Read Pair Distance (bp)** | **Median Read Depth*** |
| --- | --- | --- | --- | --- | --- | --- | --- |
| 1 | PGSP4739 | 11,000,295 | 6,697,079 | 95.12% | 72.00% | 588 | 55 |
| 2 | PGSP4740 | 11,107,061 | 6,613,774 | 94.96% | 70.53% | 594 | 53 |
| 3 | PGSP4742 | 13,615,279 | 8,297,081 | 97.81% | 72.15% | 586 | 69 |
| 4 | PGSP4744 | 13,492,196 | 6,692,863 | 97.24% | 58.68% | 569 | 60 |
| 5 | PGSP4918 | 5,758,811 | 3,667,894 | 90.67% | 75.49% | 602 | 29 |
| 6 | PGSP4919 | 5,544,340 | 3,775,148 | 95.87% | 78.28% | 606 | 31 |
| 7 | PGSP4920 | 5,339,542 | 3,675,956 | 96.37% | 79.84% | 610 | 28 |
| 8 | PGSP4921 | 5,265,469 | 3,517,672 | 94.80% | 77.53% | 609 | 29 |
| 9 | PGSP4922 | 6,977,384 | 4,622,159 | 94.09% | 77.68% | 616 | 37 |
| 10 | PGSP4924 | 7,280,268 | 4,601,452 | 93.20% | 74.06% | 609 | 38 |

**AO.** Statistics from *rad52Δ* uGCR strains

| **No.** | **Sample** | **No. Read Pairs** | **No. Uniquely Mapping Read Pairs** | **% Read 1 Mapped** | **% Read 2 Mapped** | **Median Read Pair Distance (bp)** | **Median Read Depth*** |
| --- | --- | --- | --- | --- | --- | --- | --- |
| 1 | PGSP329 | 7,784,540 | 2,817,411 | 48.44% | 41.93% | 580 | 22 |
| 2 | PGSP330 | 7,608,553 | 3,680,426 | 67.48% | 54.73% | 604 | 30 |
| 3 | PGSP331 | 6,981,400 | 3,804,668 | 75.61% | 64.12% | 572 | 30 |
| 4 | PGSP332 | 7,475,067 | 3,011,973 | 56.11% | 46.19% | 598 | 23 |
| 5 | PGSP333 | 10,214,107 | 4,510,183 | 60.82% | 50.74% | 604 | 35 |
| 6 | PGSP334 | 9,475,218 | 4,857,948 | 71.67% | 57.99% | 597 | 39 |
| 7 | PGSP335 | 14,059,304 | 5,154,880 | 49.11% | 41.96% | 580 | 42 |
| 8 | PGSP336 | 8,205,341 | 4,046,442 | 67.06% | 56.06% | 588 | 33 |
| 9 | PGSP337 | 9,394,323 | 5,527,477 | 79.77% | 67.19% | 589 | 44 |
| 10 | PGSP338 | 9,162,689 | 42,68,174 | 62.79% | 53.46% | 584 | 33 |
| 11 | PGSP339 | 11,396,117 | 6,273,439 | 73.72% | 63.51% | 573 | 49 |
| 12 | PGSP340 | 12,968,494 | 4,100,487 | 42.75% | 36.36% | 589 | 32 |

**AP.** Statistics from *sae2Δ* *rad52Δ* uGCR strains

| **No.** | **Sample** | **No. Read Pairs** | **No. Uniquely Mapping Read Pairs** | **% Read 1 Mapped** | **% Read 2 Mapped** | **Median Read Pair Distance (bp)** | **Median Read Depth*** |
| --- | --- | --- | --- | --- | --- | --- | --- |
| 1 | PGSP4877 | 11,540,294 | 7,584,712 | 96.75% | 81.32% | 608 | 67 |
| 2 | PGSP4878 | 9,006,587 | 6,237,557 | 97.11% | 84.00% | 587 | 55 |
| 3 | PGSP4881 | 9,025,878 | 6,049,049 | 97.73% | 80.85% | 646 | 55 |
| 4 | PGSP4882 | 11,213,617 | 7,511,926 | 97.24% | 81.32% | 636 | 69 |
| 5 | PGSP4883 | 12,196,811 | 8,164,996 | 98.15% | 81.69% | 646 | 76 |
| 6 | PGSP4884 | 9,851,823 | 6,701,807 | 96.84% | 82.47% | 622 | 61 |
| 7 | PGSP4885 | 12,311,991 | 8,103,805 | 96.44% | 80.19% | 644 | 72 |
| 8 | PGSP4886 | 9,105,380 | 6,162,865 | 97.28% | 82.77% | 610 | 55 |
| 9 | PGSP4887 | 10,343,410 | 7,372,653 | 96.11% | 81.38% | 630 | 60 |
| 10 | PGSP4888 | 9,460,555 | 6,087,313 | 94.61% | 77.80% | 615 | 55 |
| 11 | PGSP4889 | 12,605,817 | 8,352,965 | 96.92% | 80.73% | 638 | 75 |
| 12 | PGSP4890 | 12,600,689 | 8,351,364 | 95.85% | 80.57% | 637 | 70 |

*Median read depth is the median number of times that each base in uniquely mapping regions of the nuclear genome was present within a read.

**Statistics for these samples are combined from two separate WGS runs.
