# Supplementary material for: Mechanisms underlying genome instability mediated by formation of foldback inversions in Saccharomyces cerevisiae
Source: eLife. 2020 Aug 7;9:e58223. doi: 10.7554/eLife.58223 (PMC7467729; doi:10.7554/eLife.58223)
Supplement: Supplementary file 3. [file elife-58223-supp3.docx]

**Supplementary File 3. GCR structures**

**A.** GCR structures from *sae2Δ* uGCR strains

| ***No.*** | ***Sample**** | ***GCR description*** | ***GCR evidence***** |
| --- | --- | --- | --- |
| 1 | PGSP1987  [bzg048] | Hairpin-mediated inversion chrV L 34,339-107_34,339-75 (in *can1::hisG*) | Copy number  Discordant read pairs (83 read pairs)  Junction sequence (159 reads) |
|  |  | Homology-mediated translocation between chrV L *ura3-52* and chrV L *yel068c::URA3* | Copy number  *hph*^+^ |
| 2 | PGSP1989  [bzg049] | Hairpin-mediated inversion chrV L 42,855_42,869 | Copy number  Discordant read pairs (326 read pairs)  Junction sequence (52 reads) |
|  |  | Homology-mediated translocation between chrV L *ura3-52* and chrV L *yel068c::URA3* | Copy number  *hph*^+^ |
| 3 | PGSP1990  [bzg050] | Hairpin-mediated inversion chrV L 34,339-107_34,339-75 (in *can1::hisG*) | Copy number  Discordant read pairs (126 read pairs)  Junction sequence (171 reads) |
|  |  | Homology-mediated translocation between chrV L *ura3-52* and chrV L *yel068c::URA3* | Copy number  *hph*^+^ |
| 4 | PGSP1991  [bzg051] | Hairpin-mediated inversion chrV L 42,939_42,955 | Copy number  Discordant read pairs (126 read pairs)  Junction sequence (55 reads) |
|  |  | Homology-mediated translocation between chrV L *ura3-52* and chrXIV L *YNLCTy1-1* | Copy number  *hph*^-^ |
| 5 | PGSP1992  [bzg052] | Hairpin-mediated inversion chrV L 34,339-107_34,339-75 (in *can1::hisG*) | Copy number  Discordant read pairs (157 read pairs)  Junction sequence (13 reads) |
|  |  | Homology-mediated translocation between chrV L *ura3-52* and chrV L *yel068c::URA3* | Copy number  *hph*^+^ |
| 6 | PGSP1993 | *De novo* telomere addition chrV L 42,807_42,810 | Copy number  Discordant read pairs (114 read pairs)  Junction sequence (213 reads) |
| 7 | PGSP1994  [bzg053] | Hairpin-mediated inversion chrV L 34,339-107_34,339-75 (in *can1::hisG*) | Copy number  Discordant read pairs (262 read pairs)  Junction sequence (201 reads) |
|  |  | Homology-mediated translocation between chrV L *ura3-52* and chrV L *yel068c::URA3* | Copy number  *hph*^+^ |
| 8 | PGSP1995  [bzg054] | Hairpin-mediated inversion chrV L 27,444_27,465 two-step hairpin | Copy number  Discordant read pairs (192 read pairs)  Junction sequence (151 reads) |
|  |  | Homology-mediated translocation between chrV L *PAU2* and chrII R *PAU24* | Copy number  Discordant read pairs (225 read pairs)  *hph*^-^ |
| 9 | PGSP1996  [bzg055] | Hairpin-mediated inversion chrV L 34,339-107_34,339-75 (in *can1::hisG*) | Copy number  Discordant read pairs (126 read pairs)  Junction sequence (253 reads) |
|  |  | Homology-mediated translocation between chrV L *ura3-52* and chrV L *yel068c::URA3* | Copy number  *hph*^+^ |
| 10 | PGSP1997  [bzg056] | Hairpin-mediated inversion chrV L 34,339-107_34,339-75 (in *can1::hisG*) | Copy number  Discordant read pairs (135 read pairs)  Junction sequence (223 reads) |
|  |  | translocation chrV L *GCN4* with chrV L *iYEL072w::hph* | Copy number  Discordant read pairs (410 read pairs)  *hph*^+^ |
| 11 | PGSP1998  [bzg057] | Hairpin-mediated inversion chrV L 34,339-107_34,339-75 (in *can1::hisG*) | Copy number  Discordant read pairs (109 read pairs)  Junction sequence (153 reads) |
|  |  | Homology-mediated translocation between chrV L *ura3-52* and chrXIV L *YNLCTy1-1* | Copy number  *hph*^-^ |
| 12 | PGSP1999  [bzg058] | Hairpin-mediated inversion chrV L 34,339-107_34,339-75 (in *can1::hisG*) | Copy number  Discordant read pairs (111 read pairs)  Junction sequence (144 reads) |
|  |  | Homology-mediated translocation between chrV L *ura3-52* and chrIV R *YDRWTy1-4* | Copy number  *hph*^-^ |
| 13 | PGSP2000  [bzg059] | Hairpin-mediated inversion chrV L 34,339-107_34,339-75 (in *can1::hisG*) | Copy number  Discordant read pairs (145 read pairs)  Junction sequence (246 reads) |
|  |  | Homology-mediated translocation between chrV L *ura3-52* and chrXIV L *YNLCTy1-1* | Copy number  *hph*^-^ |
| 14 | PGSP3633  [bzg060] | Hairpin-mediated inversion chrV L 25,817-25_25,817-14 | Copy number  Discordant read pairs (223 read pairs)  Junction sequence (122 reads) |
|  |  | Homology-mediated translocation between chrV L *ura3-52* and chrIII R *YCRWdelta11* | Copy number  *hph*^-^ |
| 15 | PGSP3634  [bzg061] | Hairpin-mediated inversion chrV L 34,339-107_34,339-75 (in *can1::hisG*) | Copy number  Discordant read pairs (320 read pairs)  Junction sequence (328 reads) |
|  |  | Homology-mediated translocation between chrV L *ura3-52* and chrV L *yel068c::URA3* | Copy number  *hph*^+^ |
| 16 | PGSP3636  [bzg062] | Hairpin-mediated inversion chrV L 34,339-107_34,339-75 (in *can1::hisG*) | Copy number  Discordant read pairs (230 read pairs)  Junction sequence (265 reads) |
|  |  | Homology-mediated translocation between chrV L *ura3-52* and chrV L *yel068c::URA3* | Copy number  *hph*^+^ |
| 17 | PGSP3637  [bzg063] | Hairpin-mediated inversion chrV L 34,339-107_34,339-75 (in *can1::hisG*) | Copy number  Discordant read pairs (222 read pairs)  Junction sequence (414 reads) |
|  |  | Homology-mediated translocation between chrV L *ura3-52* and chrV L *yel068c::URA3* | Copy number  *hph*^+^ |
| 18 | PGSP3638  [bzg064] | Hairpin-mediated inversion chrV L 34,339-107_34,339-75 (in *can1::hisG*) | Copy number  Discordant read pairs (243 read pairs)  Junction sequence (379 reads) |
|  |  | Homology-mediated translocation between chrV L *ura3-52* and chrXIV L *YNLCTy1-1* | Copy number  *hph*^-^ |
| 19 | PGSP3641  [bzg065] | Hairpin-mediated inversion chrV L 34,339-107_34,339-75 (in *can1::hisG*) | Copy number  Discordant read pairs (275 read pairs)  Junction sequence (282 reads) |
|  |  | Homology-mediated translocation between chrV L *ura3-52* and chrIII R *YCRWdelta8/9/10* | Copy number  *hph*^-^ |
| 20 | PGSP3642  [bzg066] | Hairpin-mediated inversion chrV L 34,339-107_34,339-75 (in *can1::hisG*) | Copy number  Discordant read pairs (212 read pairs)  Junction sequence (224 reads) |
|  |  | Homology-mediated translocation between chrV L *ura3-52* and chrV L *yel068c::URA3* | Copy number  *hph*^+^ |

**B.** GCR structures from *sae2-S267A* uGCR strains

| ***No.*** | ***Sample**** | ***GCR description*** | ***GCR evidence***** |
| --- | --- | --- | --- |
| 1 | PGSP4760 | Hairpin-mediated inversion chrV L 34,339-107_34,339-75 (in *can1::hisG*) | Copy number  Discordant read pairs (81 read pairs)  Junction sequence (136 reads) |
|  |  | Homology-mediated translocation between chrV L *ura3-52* and the unannotated chrV R “*YERWdelta27”* (chrV:449,322..449,631; Nene et al. 2018) | Copy number |
| 2 | PGSP4761 | Hairpin-mediated inversion chrV L 34,339-107_34,339-75 (in *can1::hisG*) | Copy number  Discordant read pairs (55 read pairs)  Junction sequence (125 reads) |
|  |  | Homology-mediated translocation between chrV L *ura3-52* and chrIV R *YDRWTy1-4* | Copy number |
| 3 | PGSP4762 | Hairpin-mediated inversion chrV L 34,339-107_34,339-75 (in *can1::hisG*) | Copy number  Discordant read pairs (70 read pairs)  Junction sequence (155 reads) |
|  |  | Homology-mediated translocation between chrV L *ura3-52* and the unannotated chrXII R *“YLRWTy1-4”*; in this strain background there is a full-length Ty element, “*YLRWTy1-4*” telomeric to *YLRCdelta21* and in the opposite orientation (Liang et al. 2018) | Copy number |
| 4 | PGSP4763 | Hairpin-mediated inversion chrV L 34,339-107_34,339-75 (in *can1::hisG*) | Copy number  Discordant read pairs (86 read pairs)  Junction sequence (149 reads) |
|  |  | Homology-mediated translocation between chrV L *YELWdelta1* and the unannotated chrV R “*YERWdelta27”* (chrV:449,322..449,631; Nene et al. 2018) | Copy number  Discordant read pairs (210 read pairs) |
| 5 | PGSP4764 | Hairpin-mediated inversion chrV L 34,339-107_34,339-75 (in *can1::hisG*) | Copy number  Discordant read pairs (100 read pairs)  Junction sequence (187 reads) |
|  |  | Homology-mediated translocation between chrV L *YELCdelta4* and chrV R *YERCdelta16* | Copy number  Discordant read pairs (267 read pairs) |
| 6 | PGSP4765 | Hairpin-mediated inversion chrV L 34,339-107_34,339-75 (in *can1::hisG*) | Copy number  Discordant read pairs (149 read pairs)  Junction sequence (287 reads) |
|  |  | Homology-mediated translocation between chrV L *ura3-52* and chrV L *yel068c::URA3* | Copy number |
| 7 | PGSP4766 | Hairpin-mediated inversion chrV L 34,339-107_34,339-75 (in *can1::hisG*) | Copy number  Junction sequence |
|  |  | Homology-mediated translocation between chrV L *YERCdelta4* and chrII L *YBLWTy2-1* | Copy number |
| 8 | PGSP4767 | Hairpin-mediated inversion chrV L 34,339-107_34,339-75 (in *can1::hisG*) | Copy number  Discordant read pairs (75 read pairs)  Junction sequence (156 reads) |
|  |  | Homology-mediated translocation between chrV L *YELWdelta6* and chrX R *YJRWdelta18* | Copy number  Discordant read pairs (195 read pairs) |
| 9 | PGSP4768 | Hairpin-mediated inversion chrV L 34,339-107_34,339-75 (in *can1::hisG*) | Copy number  Discordant read pairs (127 read pairs)  Junction sequence (223 reads) |
|  |  | Homology-mediated translocation between chrV L *ura3-52* and chrIII R *YCRWdelta11* | Copy number |
| 10 | PGSP4769 | Hairpin-mediated inversion chrV L 34,339-107_34,339-75 (in *can1::hisG*) | Copy number  Discordant read pairs (91 read pairs)  Junction sequence (189 reads) |
|  |  | Homology-mediated translocation between chrV L *ura3-52* and chrIII R *YCRWdelta11* | Copy number |
| 11 | PGSP4770 | Hairpin-mediated inversion chrV L 34,339-107_34,339-75 (in *can1::hisG*) | Copy number  Discordant read pairs (95 read pairs)  Junction sequence (254 reads) |
|  |  | Homology-mediated translocation between chrV L *ura3-52* and chrX R *YJRWTy1-1/YJRWTy1-2* | Copy number |
| 12 | PGSP4771 | Hairpin-mediated inversion chrV L 34,339-107_34,339-75 (in *can1::hisG*) | Copy number  Discordant read pairs (215 read pairs)  Junction sequence (346 reads) |
|  |  | Homology-mediated translocation between chrV L *YELCdelta4* and chrV L *ura3-52* | Copy number |
|  |  | Hairpin-mediated inversion chrV L 34,339-107_34,339-75 (in *can1::hisG*) | Copy number  Discordant read pairs (215 read pairs)  Junction sequence (346 reads) |
|  |  | Microhomology-mediated translocation between chrV L 94,494 and chrXIV 7,018 | Copy number  Discordant read pairs (247 read pairs)  Junction sequence (47 reads) |

**C.** GCR structures from *sae2-MT9* uGCR strains

| ***No.*** | ***Sample**** | ***GCR description*** | ***GCR evidence***** |
| --- | --- | --- | --- |
| 1 | PGSP4780 | Hairpin-mediated inversion chrV L 34,339-107_34,339-75 (in *can1::hisG*) | Copy number  Discordant read pairs (131 read pairs)  Junction sequence (258 reads) |
|  |  | Homology-mediated translocation between chrV L *ura3-52* and chrV L *yel068c::URA3* | Copy number |
| 2 | PGSP4781 | Hairpin-mediated inversion chrV L 34,339-107_34,339-75 (in *can1::hisG*) | Copy number  Discordant read pairs (79 read pairs)  Junction sequence (228 reads) |
|  |  | Homology-mediated translocation between chrV L *YELCdelta4* and chrXVI L *YPLWTy1-1* | Copy number |
| 3 | PGSP4782 | Hairpin-mediated inversion chrV L 25,817-1,303_25,817-1,289 (in inserted *CAN1*) | Copy number  Discordant read pairs (172 read pairs)  Junction sequence (27 reads) |
|  |  | Homology-mediated translocation between chrV L *PAU2* and chrI L *PAU8* | Copy number |
| 4 | PGSP4783 | Hairpin-mediated inversion chrV L 34,339-107_34,339-75 (in *can1::hisG*) | Copy number  Discordant read pairs (128 read pairs)  Junction sequence (191 reads) |
|  |  | Homology-mediated translocation between chrV L *ura3-52* and chrIII R *YCRWdelta11* | Copy number |
| 5 | PGSP4784 | Hairpin-mediated inversion chrV L 34,339-107_34,339-75 (in *can1::hisG*) | Copy number  Discordant read pairs (145 read pairs)  Junction sequence (277 reads) |
|  |  | Homology-mediated translocation between chrV L *ura3-52* and the unannotated chrV R “*YERWdelta27”* (chrV:449,322..449,631; Nene et al. 2018) | Copy number |
| 6 | PGSP4785 | Hairpin-mediated inversion chrV L 34,339-107_34,339-75 (in *can1::hisG*) | Copy number  Discordant read pairs (108 read pairs)  Junction sequence (194 reads) |
|  |  | Homology-mediated translocation between chrV L *ura3-52* and the unannotated chrV R “*YERWdelta27”* (chrV:449,322..449,631; Nene et al. 2018) | Copy number |
| 7 | PGSP4786 | Hairpin-mediated inversion chrV L 34,339-107_34,339-75 (in *can1::hisG*) | Copy number  Discordant read pairs (96 read pairs)  Junction sequence (151 reads) |
|  |  | Homology-mediated translocation between chrV L *ura3-52* and chrI R *YARWdelta6* | Copy number  Discordant read pairs (56 read pairs) |
| 8 | PGSP4787 | Hairpin-mediated inversion chrV L 34,339-107_34,339-75 (in *can1::hisG*) | Copy number  Discordant read pairs (84 read pairs)  Junction sequence (143 reads) |
|  |  | Homology-mediated translocation between chrV L *ura3-52* and chrIII R *YCRWdelta8*, *9,* or *10* | Copy number |
| 9 | PGSP4788 | Hairpin-mediated inversion chrV L 34,339-107_34,339-75 (in *can1::hisG*) | Copy number  Discordant read pairs (90 read pairs)  Junction sequence (221 reads) |
|  |  | Homology-mediated translocation between chrV L *YELCdelta4* and chrIV R *YDRWTy2-3/Ty1*-3 | Copy number |
| 10 | PGSP4789 | Translocation chrV L 42,392 and chrXII R 457,225 (rDNA repeats) | Copy number  Junction sequence |
| 11 | PGSP4790 | Hairpin-mediated inversion chrV L 34,339-107_34,339-75 (in *can1::hisG*) | Copy number  Discordant read pairs (110 read pairs)  Junction sequence (214 reads) |
|  |  | Homology-mediated translocation between chrV L *ura3-52* and chrIII R *YCRWdelta8*, *9,* or *10* | Copy number |
| 12 | PGSP4791 | Hairpin-mediated inversion chrV L 34,339-931_34,339-919 (in *can1::hisG*) | Copy number  Discordant read pairs (153 read pairs)  Junction sequence (34 reads) |
|  |  | Homology-mediated translocation between chrV L *ura3-52* and chrVI R *YDRWTy1-5* | Copy number |

**D.** GCR structures from *sae2Δ hs-del* uGCR strains

| ***No.*** | ***Sample**** | ***GCR description*** | ***GCR evidence***** |
| --- | --- | --- | --- |
| 1 | PGSP4998 | Interstitial deletion between chrV L 35,940 and chrV L 3,699 | Copy number  Discordant read pairs (60 read pairs)  Junction sequence (8 reads) |
| 2 | PGSP4999 | Interstitial deletion between chrV L 39,606 and chrV L 1,726 (or translocation to other subtelomeric region) | Copy number  Discordant read pairs (114 read pairs)  Junction sequence (10 reads) |
| 3 | PGSP5000 | Hairpin-mediated inversion chrV L 39,661_39,681 | Copy number  Discordant read pairs (114 read pairs)  Junction sequence (20 reads) |
|  |  | Translocation between chrV L *ura3-52* and chrXV *YOLCdelta1* | Copy number  Discordant read pairs (166 read pairs) |
|  |  | Homology-mediated translocation between chrXV 2,247 and chrIII R 311,207 | Copy number  Discordant read pairs (87 read pairs) |
| 4 | PGSP5001 | Microhomology-mediated translocation between chrV L 29,062 and chrIV R 220,696 | Copy number  Discordant read pairs (70 read pairs)  Junction sequence (33 reads) |
| 5 | PGSP5002 | Hairpin-mediated inversion chrV L 35,577_35,595 | Copy number  Discordant read pairs (144 read pairs)  Junction sequence (54 reads) |
|  |  | Homology-mediated translocation between chrV L *ura3-52* and chrIII *YCRWdelta8/9/10* | Copy number |
| 6 | PGSP5003 | Hairpin-mediated inversion chrV L 29,068_29,103 | Copy number  Discordant read pairs (233 read pairs)  Junction sequence (43 reads) |
|  |  | Homology-mediated translocation between chrV L *ura3-52* and chrIV R *YDRWTy2-2* | Copy number |
| 7 | PGSP5004 | Hairpin-mediated inversion chrV L 30,830_30,856 | Copy number  Discordant read pairs (26 read pairs)  Junction sequence (29 reads) |
|  |  | Homology-mediated translocation between chrV L *ura3-52* and the unannotated chrV R “*YERWdelta27”* (chrV:449,322..449,631; Nene et al. 2018) | Copy number |
| 8 | PGSP5005 | Hairpin-mediated inversion chrV L 25,817-592_25,817-570 (in inserted *CAN1*) | Copy number  Discordant read pairs (94 read pairs)  Junction sequence (21 reads) |
|  |  | Homology-mediated translocation between chrV L *ura3-52* and chrIV R *YDRWTy1-4* | Copy number |
| 9 | PGSP5006 | Hairpin-mediated inversion chrV L 25,817-30_25,820 (in inserted *CAN1*) | Copy number  Discordant read pairs (188 read pairs)  Junction sequence (94 reads) |
|  |  | Homology-mediated translocation between chrV L *YELCdelta4* and chrXII R *YLRCTy2-2* | Copy number |
| 10 | PGSP5007 | Hairpin-mediated inversion chrV L 34,339-1,211_34,339-1,196 (in *can1::hisG*) | Copy number  Discordant read pairs (70 read pairs)  Junction sequence (64 reads) |
|  |  | Homology-mediated translocation between chrV L *ura3-52* and chrVII R *YGRWTy1-1* | Copy number |
| 11 | PGSP5008 | Hairpin-mediated inversion chrV L 37,132_37,150 | Copy number  Discordant read pairs (422 read pairs)  Junction sequence (132 reads) |
|  |  | Homology-mediated translocation between chrV L *ura3-52* and chrIII *YCRWdelta8/9/10* | Copy number |
| 12 | PGSP5009 | Hairpin-mediated inversion chrV L 25,817-43_25,817-20 | Copy number  Discordant read pairs (308 read pairs)  Junction sequence (223 reads) |
|  |  | Hairpin-mediated inversion chrV L 72,380_72,456 | Copy number  Discordant read pairs (194 read pairs)  Junction sequence (223 reads) |
|  |  | Hairpin-mediated inversion chrV L 25,817-43_25,817-20 | Copy number  Discordant read pairs (308 read pairs)  Junction sequence (223 reads) |
|  |  | Hairpin-mediated inversion chrV L 72,380_72,456 | Copy number  Discordant read pairs (194 read pairs)  Junction sequence (223 reads) |
|  |  | Hairpin-mediated inversion chrV L 25,817-43_25,817-20 | Copy number  Discordant read pairs (308 read pairs)  Junction sequence (223 reads) |
|  |  | Homology-mediated translocation between chrV L *ura3-52* and chrIV R *YDRWTy1-5* | Copy number |

**E.** GCR structures from wild-type chrV:30,843-gRNA (telomeric to hotspot) uGCR strains

| ***No.*** | ***Sample**** | ***GCR description*** | ***GCR evidence***** |
| --- | --- | --- | --- |
| 1 | PGSP4842 | *De novo* telomere addition chrV L 30,844_30,847 | Copy number  Discordant read pairs (49 read pairs)  Junction sequence (8 reads) |
| 2 | PGSP4843 | *De novo* telomere addition chrV L 30,844_30,847 | Copy number  Discordant read pairs (21 read pairs)  Junction sequence (13 reads) |
| 3 | PGSP4844 | *De novo* telomere addition chrV L 30,844_30,847 | Copy number  Discordant read pairs (22 read pairs)  Junction sequence (51 reads) |
| 4 | PGSP4845 | *De novo* telomere addition chrV L 30,844_30,847 | Copy number  Discordant read pairs (46 read pairs)  Junction sequence (3 reads) |
| 5 | PGSP4846 | *De novo* telomere addition chrV L 30,844_30,847 | Copy number  Discordant read pairs (45 read pairs)  Junction sequence (15 reads) |
| 6 | PGSP4847 | *De novo* telomere addition chrV L 30,844_30,847 | Copy number  Discordant read pairs (17 read pairs)  Junction sequence (6 reads) |
| 7 | PGSP5028 | *De novo* telomere addition chrV L 30,890_30,890 | Copy number  Discordant read pairs (20 read pairs)  Junction sequence (4 reads) |
| 8 | PGSP5029 | *De novo* telomere addition chrV L 30,844_30,847 | Copy number  Discordant read pairs (13 read pairs)  Junction sequence (5 reads) |
| 9 | PGSP5030 | *De novo* telomere addition chrV L 30,844_30,847 | Copy number  Discordant read pairs (32 read pairs)  Junction sequence (5 reads) |
| 10 | PGSP5031 | Microhomology-mediated translocation from chrV L 34,339-1140 (in *can1::hisG*) to repetitive subtelomeric region | Copy number  Discordant read pairs (69 read pairs)  Junction sequence (7 reads) |
| 11 | PGSP5032 | *De novo* telomere addition chrV L 30,844_30,847 | Copy number  Discordant read pairs (30 read pairs)  Junction sequence (4 reads) |
| 12 | PGSP5033 | *De novo* telomere addition chrV L 30,844_30,847 | Copy number  Discordant read pairs (16 reads)  Junction sequence (18 reads) |

**F.** GCR structures from *sae2Δ* chrV:30,843-gRNA (telomeric to hotspot) uGCR strains

| ***No.*** | ***Sample**** | ***GCR description*** | ***GCR evidence***** |
| --- | --- | --- | --- |
| 1 | PGSP4829 | Hairpin-mediated inversion chrV L 30,857_30,872 | Copy number  Junction sequence (89 reads) |
|  |  | Homology-mediated translocation between chrV L *ura3-52* and chrV L *yel068c::URA3* | Copy number |
| 2 | PGSP4830 | Hairpin-mediated inversion chrV L 30,857_30,872 | Copy number  Discordant read pairs (32 read pairs)  Junction sequence (138 reads) |
|  |  | Homology-mediated translocation between chrV L *ura3-52* and chrV L *yel068c::URA3* | Copy number |
| 3 | PGSP4831 | Hairpin-mediated inversion chrV L 30,857_30,872 | Copy number  Discordant read pairs (43 read pairs)  Junction sequence (13 reads) |
|  |  | Homology-mediated translocation between chrV L *ura3-52* and chrV L *yel068c::URA3* | Copy number |
| 4 | PGSP4832 | Hairpin-mediated inversion chrV L 34,339-107_34,339-75 (in *can1::hisG*) | Copy number  Discordant read pairs (128 read pairs)  Junction sequence (220 reads) |
|  |  | Homology-mediated translocation between chrV L *ura3-52* and chrV L *yel068c::URA3* | Copy number |
| 5 | PGSP4833 | Hairpin-mediated inversion chrV L 30,857_30,872 | Copy number  Discordant read pairs (43 read pairs)  Junction sequence (155 reads) |
|  |  | Homology-mediated translocation between chrV L *ura3-52* and the unannotated chrXII R *“YLRWTy1-4”*; in this strain background there is a full-length Ty element, “*YLRWTy1-4*” telomeric to *YLRCdelta21* and in the opposite orientation (Liang et al. 2018) | Copy number |
| 6 | PGSP4834 | Hairpin-mediated inversion chrV L 30,857_30,872 | Copy number  Junction sequence (61 reads) |
|  |  | Homology-mediated translocation between chrV L *ura3-52* and chrV L *yel068c::URA3* | Copy number |
| 7 | PGSP5034 | Hairpin-mediated inversion chrV L 34,339-107_34,339-75 (in *can1::hisG*) | Copy number  Discordant read pairs (29 read pairs)  Junction sequence |
|  |  | Homology-mediated translocation between chrV L *ura3-52* and chrV L *yel068c::URA3* | Copy number |
| 8 | PGSP5035 | Hairpin-mediated inversion chrV L 30,857_30,872 | Copy number  Discordant read pairs (12 read pairs)  Junction sequence |
|  |  | Homology-mediated translocation between chrV L *ura3-52* and chrV L *yel068c::URA3* | Copy number |
| 9 | PGSP5036 | Hairpin-mediated inversion chrV L 30,857_30,872 | Copy number  Discordant read pairs (14 read pairs)  Junction sequence |
|  |  | Homology-mediated translocation between chrV L *ura3-52* and chrV L *yel068c::URA3* | Copy number |
| 10 | PGSP5037 | Microhomology-mediated inversion translocation between chrV L 30,849 and *ura3-52.* The junction sequence maps to a common Ty-related sequence; however, the duplication of *URA3* after the Ty insertion in the *ura3-52* allele and the retention of the region from chrV L *yel068c::URA3* to the chrV L telomere indicates that the target *ura3-52*. | Copy number  Discordant read pairs (19 read pairs)  Junction sequence |
|  |  | Homology-mediated translocation between chrV L *ura3-52* and chrV L *yel068c::URA3* | Copy number |
| 11 | PGSP5038 | Nonhomology-mediated translocation between chrV L 30,845 and repetitive subtelomeric region | Copy number  Discordant read pairs (40 read pairs)  Junction sequence (8 reads) |

**G.** GCR structures from wild-type chrV:34,470-gRNA (centromeric to hotspot) uGCR strains

| ***No.*** | ***Sample**** | ***GCR description*** | ***GCR evidence***** |
| --- | --- | --- | --- |
| 1 | PGSP4836 | *De novo* telomere addition chrV L 38,086_38,090 | Copy number  Discordant read pairs (90 read pairs)  Junction sequence (12 reads) |
| 2 | PGSP4837 | *De novo* telomere addition chrV L 34,860_34,861 | Copy number  Discordant read pairs (83 read pairs)  Junction sequence (21 reads) |
| 3 | PGSP4838 | *De novo* telomere addition chrV L 34,833 | Copy number  Discordant read pairs (60 read pairs)  Junction sequence (16 reads) |
| 4 | PGSP4839 | *De novo* telomere addition chrV L 34,831_34,832 | Copy number  Discordant read pairs (65 read pairs)  Junction sequence (11 reads) |
| 5 | PGSP4840 | *De novo* telomere addition chrV L 34,441_34,445 | Copy number  Discordant read pairs (80 read pairs)  Junction sequence (13 reads) |
| 6 | PGSP4841 | *De novo* telomere addition chrV L 35,388_35,391 | Copy number  Discordant read pairs (86 read pairs)  Junction sequence (11 reads) |
| 7 | PGSP5022 | *De novo* telomere addition chrV L ~34,904 | Copy number  Discordant read pairs (14 read pairs) |
| 8 | PGSP5023 | Microhomology-mediated translocation between chrV L 36,845 and chrIII R 295,060 | Copy number  Discordant read pairs (75 read pairs)  Junction sequence (22 reads) |
| 9 | PGSP5024 | Microhomology-mediated interstitial deletion between chrV L 34,520 and 24,295 | Copy number  Discordant read pairs (76 read pairs)  Junction sequence (9 reads) |
| 10 | PGSP5025 | Microhomology-mediated inversion translocation between chrV L 34,487 and *ura3-52.* The junction sequence maps to a common Ty-related sequence; however, the duplication of *URA3* after the Ty insertion in the *ura3-52* allele and the retention of the region from chrV L *yel068c::URA3* to the chrV L telomere indicates that the target *ura3-52*. | Copy number  Discordant read pairs (56 read pairs)  Junction sequence (9 reads) |
|  |  | Homology-mediated translocation between chrV L *ura3-52* and chrV L *yel068c::URA3* | Copy number |
| 11 | PGSP5026 | Microhomology-mediated inversion translocation between chrV L 34,500 and *ura3-52.* The junction sequence maps to a common Ty-related sequence; however, the duplication of *URA3* after the Ty insertion in the *ura3-52* allele and the retention of the region from chrV L *yel068c::URA3* to the chrV L telomere indicates that the target *ura3-52*. | Copy number  Discordant read pairs (97 read pairs) |
|  |  | Homology-mediated translocation between chrV L *ura3-52* and chrV L *yel068c::URA3* | Copy number |
| 12 | PGSP5027 | *De novo* telomere addition chrV L 39,089_39,089 | Copy number  Discordant read pairs (12 read pairs)  Junction sequence (6 reads) |

**H.** GCR structures from *sae2Δ* chrV:34,470-gRNA (centromeric to hotspot) uGCR strains

| ***No.*** | ***Sample**** | ***GCR description*** | ***GCR evidence***** |
| --- | --- | --- | --- |
| 1 | PGSP4822 | *De novo* telomere addition chrV L 34,849_34,852 | Copy number  Discordant read pairs (58 read pairs)  Junction sequence (6 reads) |
| 2 | PGSP4823 | Microhomology-mediated translocation between chrVL 34,471 and chrVII R 1,081,930 | Copy number  Discordant read pairs (127 read pairs)  Junction sequence (24 reads) |
| 3 | PGSP4824 | *De novo* telomere addition chrV L 34,891_34,900 | Copy number  Discordant read pairs (82 read pairs)  Junction sequence (11 reads) |
| 4 | PGSP4825 | Microhomology-mediated translocation chrV L 34,497 and chrI R 225,635 | Copy number  Discordant read pairs (30 read pairs)  Junction sequence (11 reads) |
| 5 | PGSP4826 | *De novo* telomere addition chrV L 34,842_34,847 | Copy number  Discordant read pairs (88 read pairs)  Junction sequence (32 reads) |
| 6 | PGSP4827 | Microhomology-mediated translocation chrV L 34,471 to subtelomeric Y’ element | Copy number  Junction sequence (16 reads) |
| 7 | PGSP5039 | Hairpin-mediated inversion chrV L 30,857_30,872 | Copy number  Junction sequence |
|  |  | Homology-mediated translocation between chrV L *ura3-52* and chrV L *yel068c::URA3* | Copy number |
| 8 | PGSP5040 | Microhomology-mediated interstitial deletion between chrV L 34,484 and chrV L 6,656 | Copy number  Discordant read pairs (93 read pairs)  Junction sequence (9 reads) |
| 9 | PGSP5041 | Microhomology-mediated translocation chrV L ~34,518 to chrXI R ~642,659 | Copy number  Discordant read pairs (17 read pairs) |
| 10 | PGSP5042 | Microhomology-mediated inversion translocation between chrV L 34,480 and *ura3-52.* The junction sequence maps to a common Ty-related sequence; however, the duplication of *URA3* after the Ty insertion in the *ura3-52* allele and the retention of the region from chrV L *yel068c::URA3* to the chrV L telomere indicates that the target *ura3-52*. | Copy number  Discordant read pairs (56 read pairs)  Junction sequence (8 reads) |
|  |  | Homology-mediated translocation between chrV L *ura3-52* and chrV L *yel068c::URA3* | Copy number |
| 11 | PGSP5043 | Microhomology-mediated inversion translocation between chrV L ~34,474 and *ura3-52.* The junction sequence maps to a common Ty-related sequence; however, the duplication of *URA3* after the Ty insertion in the *ura3-52* allele and the retention of the region from chrV L *yel068c::URA3* to the chrV L telomere indicates that the target *ura3-52*. | Copy number  Discordant read pairs (19 read pairs) |
|  |  | Homology-mediated translocation between chrV L *ura3-52* and chrV L *yel068c::URA3* | Copy number |
| 12 | PGSP5044 | Microhomology-mediated inversion translocation between chrV L 34,487 and *ura3-52.* The junction sequence maps to a common Ty-related sequence; however, the duplication of *URA3* after the Ty insertion in the *ura3-52* allele and the retention of the region from chrV L *yel068c::URA3* to the chrV L telomere indicates that the target *ura3-52*. | Copy number  Discordant read pairs (67 read pairs)  Junction sequence |
|  |  | Homology-mediated translocation between chrV L *ura3-52* and chrV L *yel068c::URA3* | Copy number |
| 13 | PGSP5045 | Hairpin-mediated inversion chrV L 35,581_35,596 | Copy number  Discordant read pairs (18 read pairs)  Junction sequence (3 reads) |
|  |  | Homology-mediated translocation between chrV L *ura3-52* and the unannotated chrV R “*YERWdelta27”* (chrV:449,322..449,631; Nene et al. 2018) | Copy number |

**I.** GCR structures from *sae2Δ* chrV:25,817-1,749 gRNA (telomeric to hotspot in *CAN1*) uGCR strains

| ***No.*** | ***Sample**** | ***GCR description*** | ***GCR evidence***** |
| --- | --- | --- | --- |
| 1 | PGSP4974 | Homology mediated translocation between chrV L 32,679 (in inserted *CAN1*) and chrXIV L 139,457 *LYP1* | Copy number  Discordant read pairs (299 read pairs)  Junction sequence (20 reads) |
| 2 | PGSP4975 | Hairpin-mediated inversion chrV L 34,339-107_34,339-75 (in *can1::hisG*) | Copy number  Discordant read pairs (92 read pairs)  Junction sequence (116 reads) |
|  |  | Homology-mediated translocation between chrV L *ura3-52* and chrV L *yel068c::URA3* | Copy number |
| 3 | PGSP4976 | Hairpin-mediated inversion chrV L 34,339-107_34,339-75 (in *can1::hisG*) | Copy number  Discordant read pairs (39 read pairs)  Junction sequence (79 reads) |
|  |  | Homology-mediated translocation between chrV L *ura3-52* and chrIV R *YDRWTy1-5* | Copy number |
| 4 | PGSP4977 | Hairpin-mediated inversion chrV L 34,339-107_34,339-75 (in *can1::hisG*) | Copy number  Discordant read pairs (78 read pairs)  Junction sequence (125 reads) |
|  |  | Homology-mediated translocation between chrV L *ura3-52* and chrV L *yel068c::URA3* | Copy number |
| 5 | PGSP4978 | Hairpin-mediated inversion chrV L 34,339-107_34,339-75 (in *can1::hisG*) | Copy number  Discordant read pairs (99 read pairs)  Junction sequence |
|  |  | Homology-mediated translocation between chrV L *ura3-52* and chrXIII R *YMRWdelta21* | Copy number |
| 6 | PGSP4979 | Hairpin-mediated inversion chrV L 34,339-107_34,339-75 (in *can1::hisG*) | Copy number  Discordant read pairs (86 read pairs)  Junction sequence |
|  |  | Homology-mediated translocation between chrV L *ura3-52* and chrII R *YBRWTy1-2* | Copy number |
| 7 | PGSP4980 | Hairpin-mediated inversion chrV L 25,817-1,731_25,817-1,720 | Copy number  Discordant read pairs (169 read pairs)  Junction sequence (63 reads) |
|  |  | Homology-mediated translocation between chrV L *ura3-52* and chrV L *yel068c::URA3* | Copy number |
| 8 | PGSP4981 | Hairpin-mediated inversion chrV L 34,339-107_34,339-75 (in *can1::hisG*) | Copy number  Discordant read pairs (91 read pairs)  Junction sequence (157 reads) |
|  |  | Homology-mediated translocation between chrV L *YELWdelta6* and chrXV L *YOLCdelta3* | Copy number  Discordant read pairs (357 reads) |
| 9 | PGSP4982 | Hairpin-mediated inversion chrV L 34,339-107_34,339-75 (in *can1::hisG*) | Copy number  Discordant read pairs (122 read pairs)  Junction sequence |
|  |  | Homology-mediated translocation between chrV L *YELCdelta4* and chrXVI R *YPRCTy1-2* | Copy number  Discordant read pairs (205 read pairs) |
|  |  | Homology-mediated translocation between chrXVI R *YPRCTy1-4* and chrV L *ura3-52* | Copy number |
|  |  | Homology-mediated translocation between chrV L *ura3-52* and chrV L *yel068c::URA3* | Copy number |
| 10 | PGSP4983 | Hairpin-mediated inversion chrV L 25,817-1,749_25,817-1,737 (in inserted *CAN1* sequence) | Copy number  Discordant read pairs (139 read pairs)  Junction sequence (37 reads) |
|  |  | Homology-mediated translocation between chrV L *ura3-52* and chrV L *yel068c::URA3* | Copy number |
| 11 | PGSP4984 | Hairpin-mediated inversion chrV L 34,339-107_34,339-75 (in *can1::hisG*) | Copy number  Discordant read pairs (164 read pairs)  Junction sequence (222 reads) |
|  |  | Homology-mediated translocation between chrV L *YELWdelta1* and chrV L *ura3-52* | Copy number  Discordant read pairs (258 read pairs) |
|  |  | Homology-mediated translocation between chrV L *ura3-52* and chrV L *yel068c::URA3* | Copy number |
| 12 | PGSP4985 | Hairpin-mediated inversion chrV L 34,339-107_34,339-75 (in *can1::hisG*) | Copy number  Discordant read pairs (106 read pairs)  Junction sequence (120 reads) |
|  |  | Homology-mediated translocation between chrV L *ura3-52* and chrV L *yel068c::URA3* | Copy number |

**J.** GCR structures from *sae2Δ* chrV:35,709 gRNA (centromeric to hotspot) uGCR strains

| ***No.*** | ***Sample**** | ***GCR description*** | ***GCR evidence***** |
| --- | --- | --- | --- |
| 1 | PGSP4986 | Hairpin-mediated inversion chrV L 35,721_35,754 | Copy number  Discordant read pairs (210 read pairs)  Junction sequence |
|  |  | Homology-mediated translocation between chrV L *PAU2* and chr I L *PAU8* | Copy number  Discordant read pairs (244 read pairs) |
| 2 | PGSP4987 | Hairpin-mediated inversion chrV L 35,721_35,754 | Copy number  Discordant read pairs (154 read pairs)  Junction sequence |
|  |  | Homology-mediated translocation between chrV L *ura3-52* and chrXIV L *YNLCTy1-1* | Copy number |
| 3 | PGSP4988 | Hairpin-mediated inversion chrV L 35,723_35,738 | Copy number  Discordant read pairs (127 read pairs)  Junction sequence |
|  |  | Homology-mediated translocation between chrV L *ura3-52* and chrIII R *YCRWdelta8*, *9*, or *10* | Copy number |
| 4 | PGSP4989 | Hairpin-mediated inversion chrV L 35,723_35,738 | Copy number  Discordant read pairs (116 read pairs)  Junction sequence |
|  |  | Homology-mediated translocation between chrV L *YELWdelta6* and chrX R *YJRWTy1-1/Ty1-2* | Copy number  Discordant read pairs (252 read pairs) |
| 5 | PGSP4990 | Hairpin-mediated inversion chrV L 35,721_35,754 | Copy number  Discordant read pairs (203 read pairs)  Junction sequence |
|  |  | Homology-mediated translocation between chrV L *ura3-52* and chrX R *YJRWTy1-1/Ty1-2* | Copy number |
| 6 | PGSP4991 | Hairpin-mediated inversion chrV L 35,723_35,738 | Copy number  Discordant read pairs (120 read pairs)  Junction sequence (23 reads) |
|  |  | Homology-mediated translocation between chrV L *ura3-52* and chrXV R *YORWTy2-2* | Copy number |
| 7 | PGSP4992 | Hairpin-mediated inversion chrV L 35,723_35,738 | Copy number  Discordant read pairs (116 read pairs)  Junction sequence |
|  |  | Homology-mediated translocation between chrV L *ura3-52* and chrXII R *YLRWTy2-1* | Copy number |
| 8 | PGSP4993 | Hairpin-mediated inversion chrV L 35,721_35,754 | Copy number  Discordant read pairs (145 read pairs)  Junction sequence |
|  |  | Homology-mediated translocation between chrV L *ura3-52* and chrV L *yel068c::URA3* | Copy number |
| 9 | PGSP4994 | Hairpin-mediated inversion chrV L 35,721_35,754 | Copy number  Discordant read pairs (154 read pairs)  Junction sequence |
|  |  | Homology-mediated translocation between chrV L *ura3-52* and chrIII R *YCRWdelta*8, *9*, or *10* | Copy number |
| 10 | PGSP4995 | Hairpin-mediated inversion chrV L 35,723_35,738 | Copy number  Discordant read pairs (244 read pairs)  Junction sequence |
|  |  | Homology-mediated translocation between chrV L *ura3-52* and chrV L *YELCdelta4* | Copy number |
|  |  | Hairpin-mediated inversion chrV L 35,723_35,738 | Copy number  Discordant read pairs (244 read pairs)  Junction sequence |
|  |  | Nonhomology-mediated interstitial deletion of the region including *CEN5* between chrV L 151,941 and chrV R 152,305 | Copy number  Discordant read pairs (121 read pairs)  Junction sequence (22 reads) |
| 11 | PGSP4996 | Hairpin-mediated inversion chrV L 35,723_35,738 | Copy number  Discordant read pairs (126 read pairs)  Junction sequence |
|  |  | Homology-mediated translocation between chrV L *ura3-52* and chrX R *YJRWTy1-1/1-2* | Copy number |
| 12 | PGSP4997 | Hairpin-mediated inversion chrV L 35,721_35,754 | Copy number  Discordant read pairs (219 read pairs)  Junction sequence |
|  |  | Homology-mediated translocation between chrV L *ura3-52* and chrIV R *YDRWTy1-4* | Copy number |

**K.** GCR structures from *sae2Δ* chrV:34,339-110 gRNA (telomeric to hotspot) uGCR strains

| ***No.*** | ***Sample**** | ***GCR description*** | ***GCR evidence***** |
| --- | --- | --- | --- |
| 1 | PGSP5010 | Hairpin-mediated inversion chrV L 34,339-107_34,339-75 (in *can1::hisG*) | Copy number  Discordant read pairs (57 read pairs)  Junction sequence (71 reads) |
|  |  | Homology-mediated translocation between chrV L *ura3-52* and chrX R *YJRWTy1-1* | Copy number |
| 2 | PGSP5011 | Hairpin-mediated inversion chrV L 34,339-107_34,339-75 (in *can1::hisG*) | Copy number  Discordant read pairs (31 read pairs)  Junction sequence (69 reads) |
|  |  | Homology-mediated translocation between chrV L *YELCdelta4* and chrIV R *YDRWTy1-4* | Copy number |
| 3 | PGSP5012 | Hairpin-mediated inversion chrV L 34,339-107_34,339-75 (in *can1::hisG*) | Copy number  Discordant read pairs (65 read pairs)  Junction sequence (107 reads) |
|  |  | Homology-mediated translocation between chrV L *ura3-52* and chrV L *yel068c::URA3* | Copy number |
| 4 | PGSP5013 | Hairpin-mediated inversion chrV L 34,339-107_34,339-75 (in *can1::hisG*) | Copy number  Discordant read pairs (95 read pairs)  Junction sequence |
|  |  | Homology-mediated translocation between chrV L *ura3-52* and chrV L *yel068c::URA3* | Copy number |
| 5 | PGSP5014 | Hairpin-mediated inversion chrV L 34,339-107_34,339-75 (in *can1::hisG*) | Copy number  Discordant read pairs (62 read pairs)  Junction sequence (155 reads) |
|  |  | Homology-mediated translocation between chrV L *ura3-52* and chrXIV L *YNLCTy1-1* | Copy number |
| 6 | PGSP5015 | Hairpin-mediated inversion chrV L 34,339-107_34,339-75 (in *can1::hisG*) | Copy number  Discordant read pairs (47 read pairs)  Junction sequence (92 reads) |
|  |  | Homology-mediated translocation between chrV L *ura3-52* and chrVII L *YGLWdelta8/9* | Copy number  Discordant read pairs (29 read pairs) |
|  |  | Homology-mediated translocation between chrVII L *YGLCsigma3* and *YGLWsigma2* | Copy number  Discordant read pairs (31 read pairs) |
| 7 | PGSP5016 | Hairpin-mediated inversion chrV L 34,339-107_34,339-75 (in *can1::hisG*) | Copy number  Discordant read pairs (45 read pairs)  Junction sequence |
|  |  | Homology-mediated translocation between chrV L *ura3-52* and chrV L *yel068c::URA3* | Copy number |
| 8 | PGSP5017 | Hairpin-mediated inversion chrV L 34,339-107_34,339-75 (in *can1::hisG*) | Copy number  Discordant read pairs (58 read pairs)  Junction sequence (109 reads) |
|  |  | Homology-mediated translocation between chrV L *ura3-52* and chrV L *yel068c::URA3* | Copy number |
| 9 | PGSP5018 | Hairpin-mediated inversion chrV L 34,339-107_34,339-75 (in *can1::hisG*) | Copy number  Discordant read pairs (56 read pairs)  Junction sequence (57 reads) |
|  |  | Microhomology-mediated interstitial deletion of the region including *CEN5* between *ura3-52* and chrV R 152,468 | Copy number  Discordant read pairs (109 read pairs)  Junction sequence (4 reads) |
| 10 | PGSP5019 | Hairpin-mediated inversion chrV L 34,339-107_34,339-75 (in *can1::hisG*) | Copy number  Discordant read pairs (49 read pairs)  Junction sequence (88 reads) |
|  |  | Homology-mediated translocation between chrV L *ura3-52* and chrV L *yel068c::URA3* | Copy number |
| 11 | PGSP5020 | Hairpin-mediated inversion chrV L 34,339-107_34,339-75 (in *can1::hisG*) | Copy number  Discordant read pairs (92 read pairs)  Junction sequence |
|  |  | Homology-mediated translocation between chrV L *ura3-52* and chrV L *yel068c::URA3* | Copy number |
| 12 | PGSP5021 | Hairpin-mediated inversion chrV L 34,339-107_34,339-75 (in *can1::hisG*) | Copy number  Discordant read pairs (63 read pairs)  Junction sequence (118 reads) |
|  |  | Homology-mediated translocation between chrV L *ura3-52* and chrV L *yel068c::URA3* | Copy number |

**L.** GCR structures from *sae2Δ tel1Δ* uGCR strains

| ***No.*** | ***Sample**** | ***GCR description*** | ***GCR evidence***** |
| --- | --- | --- | --- |
| 1 | PGSP3669  [bzg067] | Hairpin-mediated inversion chrV L 25,817-30_25,820 | Copy number  Discordant read pairs (531 read pairs)  Junction sequence (217 reads) |
|  |  | Homology-mediated translocation between chrV L *ura3-52* and chrV L *yel068c::URA3* | Copy number  *hph*^+^ |
| 2 | PGSP3670  [bzg068] | Hairpin-mediated inversion chrV L 25,817-1,303_25,817-1,289 | Copy number  Discordant read pairs (267 read pairs)  Junction sequence (75 reads) |
|  |  | Homology-mediated translocation between chrV L *ura3-52* and chrXVI *YPRWTy1-3* | Copy number  *hph*^-^ |
| 3 | PGSP3671  [bzg069] | Hairpin-mediated inversion chrV L 25,817-568_25,817-546 | Copy number  Discordant read pairs (205 read pairs)  Junction sequence (22 reads) |
|  |  | Homology-mediated translocation between chrV L *ura3-52* and chrV L *yel068c::URA3* | Copy number  *hph*^+^ |
| 4 | PGSP3672  [bzg070] | Hairpin-mediated inversion chrV L 34,339-107_34,339-75 (in *can1::hisG*) | Copy number  Discordant read pairs (176 read pairs)  Junction sequence (322 reads) |
|  |  | Homology-mediated translocation between chrV L *ura3-52* and chrXIV L *YNLCTy1-1* | Copy number  *hph*^-^ |
| 5 | PGSP3674  [bzg071] | Hairpin-mediated inversion chrV L 34,339-107_34,339-75 (in *can1::hisG*) | Copy number  Discordant read pairs (117 read pairs)  Junction sequence (258 reads) |
|  |  | Homology-mediated translocation between chrV L *YELCdelta4* and chrXII R *YLRCdelta26* | Copy number  Discordant read pairs (332 read pairs)  *hph*^-^ |
| 6 | PGSP3675  [bzg072] | Hairpin-mediated inversion chrV L 34,339-107_34,339-75 (in *can1::hisG*) | Copy number  Discordant read pairs (206 read pairs)  Junction sequence (377 reads) |
|  |  | Homology-mediated translocation between chrV L *ura3-52* and chrV L *yel068c::URA3* | Copy number  *hph*^+^ |
| 7 | PGSP3678  [bzg073] | Hairpin-mediated inversion chrV L 34,339-107_34,339-75 (in *can1::hisG*) | Copy number  Discordant read pairs (434 read pairs)  Junction sequence (428 reads) |
|  |  | Homology-mediated translocation between chrV L *ura3-52* and chrV L *yel068c::URA3* | Copy number  *hph*^+^ |
| 8 | PGSP3681  [bzg074] | Hairpin-mediated inversion chrV L 34,339-107_34,339-75 (in *can1::hisG*) | Copy number  Discordant read pairs (259 read pairs)  Junction sequence (389 reads) |
|  |  | Microhomology-mediated translocation between chrV L *ura3-52* and chrXII R 818,470 | Copy number  Discordant read pairs (27 read pairs)  Junction sequence (50 reads)  *hph*^-^ |
| 9 | PGSP3682  [bzg075] | Hairpin-mediated inversion chrV L 34,339-107_34,339-75 (in *can1::hisG*) | Copy number  Discordant read pairs (182 read pairs)  Junction sequence (219 reads) |
|  |  | Homology-mediated translocation between chrV L *ura3-52* and chrXIII L *YMLWTy1-1* | Copy number  *hph*^-^ |
|  |  | Translocation between chrXIII L *YMLWTy1-2* chrXVI R *tK(UUU)P/YPRWsigma2* | Copy number |
| 10 | PGSP4010  [bzg079] | Hairpin-mediated inversion chrV L 34,339-107_34,339-75 (in *can1::hisG*) | Copy number  Discordant read pairs (291 read pairs)  Junction sequence (327 reads) |
|  |  | Homology-mediated translocation between chrV L *ura3-52* and chrV L *yel068c::URA3* | Copy number  *hph*^+^ |
| 11 | PGSP4045  [bzg080] | Hairpin-mediated inversion chrV L 34,339-107_34,339-75 (in *can1::hisG*) | Copy number  Discordant read pairs (313 read pairs)  Junction sequence (312 reads) |
|  |  | Homology-mediated translocation between chrV L *ura3-52* and chrVIII L *YHLCdelta1* | Copy number  *hph*^-^ |

**M.** GCR structures from *exo1Δ* uGCR strains

| ***No.*** | ***Sample**** | ***GCR description*** | ***GCR evidence***** |
| --- | --- | --- | --- |
| 1 | PGSP773  [bzg085] | *De novo* telomere addition chrV L 34,339-344_34,339-341 (in *can1::hisG*) | Copy number  Discordant read pairs (84 read pairs)  Junction sequence (12 reads)  *hph*^-^ |
| 2 | PGSP774  [bzg086] | *De novo* telomere addition chrV L 34,860_34,861 | Copy number  Discordant read pairs (79 read pairs)  Junction sequence (57 reads)  *hph*^-^ |
| 3 | PGSP775  [bzg087] | *De novo* telomere addition chrV L 34,339-497 (in *can1::hisG*) | Copy number  Discordant read pairs (160 read pairs)  Junction sequence (20 reads)  *hph*^-^ |
| 4 | PGSP776  [bzg088] | *De novo* telomere addition chrV L 38,543_38,545 | Copy number  Discordant read pairs (134 read pairs)  Junction sequence (29 reads)  *hph*^-^ |
| 5 | PGSP777  [bzg089] | *De novo* telomere addition chrV L 29,259_29,261 | Copy number  Discordant read pairs (193 read pairs)  Junction sequence (48 reads)  *hph*^-^ |
| 6 | PGSP778  [bzg090] | *De novo* telomere addition chrV L 34,397_34,398 | Copy number  Discordant read pairs (217 read pairs)  Junction sequence (40 reads)  *hph*^-^ |
| 7 | PGSP779  [bzg091] | Non-homology translocation between chrV L 33,065 (in *CAN1* insertion) and chr X R 728,598 (in *THI11*) | Copy number  Discordant read pairs (190 read pairs)  Junction sequence (58 reads)  *hph*^-^ |
| 8 | PGSP780  [bzg092] | *De novo* telomere addition chrV L 34,868_34,870 | Copy number  Discordant read pairs (115 read pairs)  Junction sequence (39 reads)  *hph*^-^ |
| 9 | PGSP781  [bzg093] | *De novo* telomere addition chrV L 26,581_26,583 | Copy number  Discordant read pairs (175 read pairs)  Junction sequence (36 reads)  *hph*^-^ |
| 10 | PGSP782  [bzg094] | *De novo* telomere addition chrV L 39,982_39,983 | Copy number  Discordant read pairs (67 read pairs)  Junction sequence (20 reads)  *hph*^-^ |
| 11 | PGSP783  [bzg095] | *De novo* telomere addition chrV L 25,817-639_25,817-635 (in *CAN1* insertion) | Copy number  Discordant read pairs (175 read pairs)  Junction sequence (36 reads)  *hph*^-^ |

**N.** GCR structures from *sae2Δ* *exo1Δ* uGCR strains

| ***No.*** | ***Sample**** | ***GCR description*** | ***GCR evidence***** |
| --- | --- | --- | --- |
| 1 | PGSP3683  [bzg076] | Hairpin-mediated inversion chrV L 34,339-107_34,339-75 (in *can1::hisG*) | Copy number  Discordant read pairs (349 read pairs)  Junction sequence (358 reads) |
|  |  | Homology-mediated translocation between chrV L *ura3-52* and chrV L *yel068c::URA3* | Copy number  *hph*^+^ |
| 2 | PGSP3690  [bzg077] | Hairpin-mediated inversion chrV L 34,339-107_34,339-75 (in *can1::hisG*) | Copy number  Discordant read pairs (426 read pairs)  Junction sequence (391 reads) |
|  |  | Homology-mediated translocation between chrV L *ura3-52* and chrV L *yel068c::URA3* | Copy number  *hph*^+^ |
| 3 | PGSP3696  [bzg078] | Hairpin-mediated inversion chrV L 34,339-107_34,339-75 (in *can1::hisG*) | Copy number  Discordant read pairs (276 read pairs)  Junction sequence (278 reads) |
|  |  | Homology-mediated translocation between chrV L *YELCdelta4* and chrVIII R *YHRCdelta10* | Copy number  Discordant read pairs (418 read pairs)  *hph*^-^ |
| 4 | PGSP4107  [bzg081] | Hairpin-mediated inversion chrV L 34,339-107_34,339-75 (in *can1::hisG*) | Copy number  Discordant read pairs (263 read pairs)  Junction sequence (373 reads) |
|  |  | Homology-mediated translocation between chrV L *ura3-52* and chrV L *yel068c::URA3* | Copy number  *hph*^+^ |
| 5 | PGSP4116  [bzg082] | Hairpin-mediated inversion chrV L 27,074_27,102 | Copy number  Discordant read pairs (424 read pairs)  Junction sequence (70 reads) |
|  |  | Homology-mediated translocation between chrV L *YERWdelta1* and chrX L *YJLCdelta3* | Copy number  Discordant read pairs (114 read pairs)  *hph*^-^ |
| 6 | PGSP4127  [bzg084] | Hairpin-mediated inversion chrV L 34,339-1,126_34,339-1,028 (in *can1::hisG*) | Copy number  Discordant read pairs (486 read pairs)  Junction sequence (63 reads) |
|  |  | Homology-mediated translocation between chrV L *YELCdelta4* and chrIV R *YDRCTy1-3* | Copy number  Discordant read pairs (55 read pairs)  *hph*^-^ |
| 7 | PGSP4665 | Hairpin-mediated inversion chrV L 34,339-107_34,339-75 (in *can1::hisG*) | Copy number  Discordant read pairs (91 read pairs)  Junction sequence (168 reads) |
|  |  | Homology-mediated translocation between chrV L *ura3-52* and chrV L *yel068c::URA3* | Copy number |
| 8 | PGSP4666 | *De novo* telomere addition chrV L 34,441_34,445 | Copy number  Discordant read pairs (70 read pairs)  Junction sequence (6 reads) |
| 9 | PGSP4667 | Hairpin-mediated inversion chrV L 34,339-107_34,339-75 (in *can1::hisG*) | Copy number  Discordant read pairs (79 read pairs)  Junction sequence (133 reads) |
|  |  | Homology-mediated translocation between chrV L *ura3-52* and chrV L *yel068c::URA3* | Copy number |
| 10 | PGSP4668 | Hairpin-mediated inversion chrV L 34,339-107_34,339-75 (in *can1::hisG*) | Copy number  Discordant read pair (86 read pairs)  Junction sequence (154 reads) |
|  |  | Homology-mediated translocation between chrV L *ura3-52* and chrX R *YJRWTy1-1* or *YJRWTy1-2* | Copy number |
| 11 | PGSP4669 | Hairpin-mediated inversion chrV L 28,829_28,849 | Copy number  Discordant read pairs (203 read pairs)  Junction sequence (73 reads) |
|  |  | Homology-mediated translocation between chrV L *ura3-52* and chrV L *yel068c::URA3* | Copy number |
| 12 | PGSP4670 | Hairpin-mediated inversion chrV L 34,339-1,091_34,339-1,066 (in *can1::hisG*) | Copy number  Discordant read pairs (92 read pairs)  Junction sequence (19 reads) |
|  |  | Homology-mediated translocation between chrV L *ura3-52* and chrX R *YJRWTy1-1* or *YJRWTy1-2* | Copy number |
| 13 | PGSP4671 | Hairpin-mediated inversion chrV L 34,339-107_34,339-75 (in *can1::hisG*) | Copy number  Discordant read pairs (71 read pairs)  Junction sequence (162 reads) |
|  |  | Homology-mediated translocation between chrV L *ura3-52* and chrV L *yel068c::URA3* | Copy number |
| 14 | PGSP4672 | Hairpin-mediated inversion chrV L 35,723_35,738 | Copy number  Discordant read pairs (134 read pairs)  Junction sequence (32 reads) |
|  |  | Homology-mediated translocation between chrV L *ura3-52* and chrV L *yel068c::URA3* | Copy number |
| 15 | PGSP4673 | Hairpin-mediated inversion chrV L 35,723_35,738 | Copy number  Discordant read pairs (111 read pairs)  Junction sequence (32 reads) |
|  |  | Homology-mediated translocation between chrV L *ura3-52* and chrV L *yel068c::URA3* | Copy number |
| 16 | PGSP4674 | Hairpin-mediated inversion chrV L 34,339-107_34,339-75 (in *can1::hisG*) | Copy number  Discordant read pairs (74 read pairs)  Junction sequence (152 reads) |
|  |  | Homology-mediated translocation between chrV L *ura3-52* and chrV L *yel068c::URA3* | Copy number |
| 17 | PGSP4675 | *De novo* telomere addition chrV L 34,404_34,405 | Copy number  Discordant read pairs (71 read pairs)  Junction sequence (25) |
| 18 | PGSP4676 | Hairpin-mediated inversion chrV L 34,339-107_34,339-75 (in *can1::hisG*) | Copy number  Discordant read pairs (108 read pairs)  Junction sequence (211 read pairs) |
|  |  | Homology-mediated translocation between chrV L *YELWdelta6* and chrXV L *YOLCdelta1* | Copy number  Discordant read pairs (488 read pairs)  Junction sequence (34 read pairs) |

**O.** GCR structures from *sae2Δ* *exo1Δ* chrV:25,817-1,749 gRNA (telomeric to hotspot) uGCR strains

| ***No.*** | ***Sample**** | ***GCR description*** | ***GCR evidence***** |
| --- | --- | --- | --- |
| 1 | PGSP5086 | Microhomology-mediated translocation between chrV L 25,817-1,751 (in inserted *CAN1*) and chrXIV L 139,490 (in *LYP1*) | Copy number  Discordant read pairs (340 read pairs)  Junction sequence |
| 2 | PGSP5087 | Hairpin-mediated inversion chrV L 25,817-1,303_25,817-1,289 | Copy number  Discordant read pairs (249 read pairs)  Junction sequence (42 reads) |
|  |  | Homology-mediated translocation between chrV L *ura3-52* and chrV L *yel068c::URA3* | Copy number |
| 3 | PGSP5088 | Hairpin-mediated inversion chrV L 25,817-1,303_25,817-1,289 | Copy number  Discordant read pairs (237 read pairs)  Junction sequence (36 reads) |
|  |  | Homology-mediated translocation between chrV L *ura3-52* and chrV L *yel068c::URA3* | Copy number |
| 4 | PGSP5089 | Hairpin-mediated inversion chrV L 34,339-107_34,339-75 (in *can1::hisG*) | Copy number  Discordant read pairs (192 read pairs)  Junction sequence (42 reads) |
|  |  | Homology-mediated translocation between chrV L *ura3-52* and chrV L *yel068c::URA3* | Copy number |
| 5 | PGSP5090 | Interstitial deletion between chrV L 25,817-1,746 (in inserted *CAN1*) and chrV 116,148 (in inserted *URA3*) | Copy number  Discordant read pairs (546 read pairs)  Junction sequence (60 reads) |
| 6 | PGSP5091 | Hairpin-mediated inversion chrV L 34,339-107_34,339-75 (in *can1::hisG*) | Copy number  Discordant read pairs (137 read pairs)  Junction sequence (183 reads) |
|  |  | Homology-mediated translocation between chrV L *ura3-52* and chrV L *yel068c::URA3* | Copy number |
| 7 | PGSP5092 | Hairpin-mediated inversion chrV L 25,817-1,749_25,817-1,737 | Copy number  Discordant read pairs (279 read pairs)  Junction sequence (48 reads) |
|  |  | Homology-mediated translocation between chrV L *ura3-52* and chrV L *yel068c::URA3* | Copy number |
| 8 | PGSP5093 | Hairpin-mediated inversion chrV L 34,339-107_34,339-75 (in *can1::hisG*) | Copy number  Discordant read pairs (148 read pairs)  Junction sequence (203 reads) |
|  |  | Homology-mediated translocation between chrV L *ura3-52* and chrV L *yel068c::URA3* | Copy number |
| 9 | PGSP5094 | Hairpin-mediated inversion chrV L 34,339-107_34,339-75 (in *can1::hisG*) | Copy number  Discordant read pairs (127 read pairs)  Junction sequence (203 reads) |
|  |  | Homology-mediated translocation between chrV L *ura3-52* and chrV L *yel068c::URA3* | Copy number |
| 10 | PGSP5095 | Hairpin-mediated inversion chrV L 34,339-107_34,339-75 (in *can1::hisG*) | Copy number  Discordant read pairs (118 read pairs)  Junction sequence (182 reads) |
|  |  | Homology-mediated translocation between chrV L *ura3-52* and chrV L *yel068c::URA3* | Copy number |
| 11 | PGSP5096 | *De novo* telomere addition chrV L 25,817-1,749_25,817_1,748 | Copy number  Discordant read pairs (35 read pairs)  Junction sequence (182 reads) |
| 12 | PGSP5097 | Hairpin-mediated inversion chrV L 25,817-1,749_25,817-1,737 | Copy number  Discordant read pairs (254 read pairs)  Junction sequence (40 reads) |
|  |  | Homology-mediated translocation between chrV L *ura3-52* and chrXVI R *YPRWTy1-3* | Copy number |

**P.** GCR structures from *rrm3Δ* uGCR strains

| ***No.*** | ***Sample**** | ***GCR description*** | ***GCR evidence***** |
| --- | --- | --- | --- |
| 1 | PGSP420  [bzg096] | *De novo* telomere addition chrV L 42,185_42,188 | Copy number  Discordant read pairs (132 read pairs)  Junction sequence (31 reads)  *hph*^-^ |
| 2 | PGSP421  [bzg097] | Hairpin-mediated inversion chrV L 25,965_26,002 | Copy number  Discordant read pairs (196 read pairs)  Junction sequence (71 reads) |
|  |  | Homology-mediated translocation between chrV L *ura3-52* and chrV L *yel068c::URA3* | Copy number  *hph*^+^ |
| 3 | PGSP422  [bzg098] | *De novo* telomere addition chrV L 34,867_34,870 | Copy number  Discordant read pairs (129 read pairs)  Junction sequence (19 reads)  *hph*^-^ |
| 4 | PGSP423  [bzg099] | *De novo* telomere addition chrV L 34,867_34,870 | Copy number  Discordant read pairs (82 read pairs)  Junction sequence (24 reads)  *hph*^-^ |
| 5 | PGSP424  [bzg100] | Microhomology-mediated translocation between chrV L 42,079 (in *PRB1*) and chrX R 475,484 (in *YJRWTy1-1*) | Copy number  Discordant read pairs (20 read pairs)  Junction sequence (24 reads)  *hph*^-^ |
| 6 | PGSP425  [bzg101] | *De novo* telomere addition chrV L 42,677_42,680 | Copy number  Discordant read pairs (111 read pairs)  Junction sequence (29 reads)  *hph*^-^ |
| 7 | PGSP426  [bzg102] | Hairpin-mediated inversion chrV L 25,817-2,487_39,967 | Copy number  Discordant read pairs (429 read pairs)  Junction sequence (29 reads) |
|  |  | Homology-mediated translocation between chrV L *ura3-52* and chrV L *yel068c::URA3* | Copy number  *hph*^+^ |
| 8 | PGSP427  [bzg103] | *De novo* telomere addition chrV L 34,872_34,874 | Copy number  Discordant read pairs (152 read pairs)  Junction sequence (33 reads)  *hph*^-^ |
| 9 | PGSP4569  [bzg104] | *De novo* telomere addition chrV L 25,817-1,103_25,817-1,103 (in *CAN1*) | Copy number  Discordant read pairs (126 read pairs)  Junction sequence (20 reads)  *hph*^-^ |
| 10 | PGSP4570  [bzg105] | Hairpin-mediated inversion chrV L 41,622_41,681 | Copy number  Discordant read pairs (230 read pairs)  Junction sequence (33 reads) |
|  |  | Homology-mediated translocation between chrV L *ura3-52* and chrV L *yel068c::URA3* | Copy number  *hph*^+^ |
| 11 | PGSP4571  [bzg106] | Hairpin-mediated inversion chrV L 40,281_40,326 | Copy number  Discordant read pairs (289 read pairs)  Junction sequence (106 reads) |
|  |  | Homology-mediated translocation between chrV L *ura3-52* and chrV L *yel068c::URA3* | Copy number  *hph*^+^ |
| 12 | PGSP4572  [bzg107] | *De novo* telomere addition chrV L 27,759_27,761 | Copy number  Discordant read pairs (141 read pairs)  Junction sequence (36 reads)  *hph*^-^ |
| 13 | PGSP4573  [bzg108] | *De novo* telomere addition chrV L 34,842_34,847 | Copy number  Discordant read pairs (133 read pairs)  Junction sequence (36 reads)  *hph*^-^ |
| 14 | PGSP4575  [bzg109] | Microhomology-mediated interstitial deletion between chrV L 18,499 and chrV L 37,590 | Copy number  Discordant read pairs (507 read pairs)  Junction sequence (82 reads)  *hph*^+^ |

**Q.** GCR structures from *sae2Δ rrm3Δ* uGCR strains

| ***No.*** | ***Sample**** | ***GCR description*** | ***GCR evidence***** |
| --- | --- | --- | --- |
| 1 | PGSP4595  [bzg145] | Hairpin-mediated inversion chrV L 34,339-107_34,339-75 (in *can1::hisG*) | Copy number  Discordant read pairs (53 read pairs)  Junction sequence (135 reads) |
|  |  | Homology-mediated translocation between chrV L *ura3-52* and chrV L *yel068c::URA3* | Copy number |
| 2 | PGSP4596  [bzg146] | Hairpin-mediated inversion chrV L 42,939_42,955 | Copy number  Discordant read pairs (183 read pairs)  Junction sequence (63 reads) |
|  |  | Homology-mediated translocation between chrV L *ura3-52* and chrX R *YJRWTy1-1/YJRWTy1-2* | Copy number |
| 3 | PGSP4597  [bzg147] | *De novo* telomere addition chrV L 26,678_26,681 | Copy number  Discordant read pairs (78 read pairs)  Junction sequence (27 reads) |
| 4 | PGSP4598  [bzg148] | Microhomology-mediated translocation chrV L 31,598 to 2-micron 5,888 | Discordant read pairs (925 read pairs)  Junction sequence (102 reads) |
|  |  | Inverted duplication in 2-micron | Copy number change in chrV |
|  |  | Homology-mediated translocation between chrV L *ura3-52* and chrV L *yel068c::URA3* | Copy number |
| 5 | PGSP4599  [bzg149] | *De novo* telomere addition chrV L 34,339-494_34,339-492 (in *can1::hisG*) | Copy number  Discordant read pairs (133 read pairs)  Junction sequence (21 reads) |
| 6 | PGSP4600  [bzg150] | Hairpin-mediated inversion chrV L 30,857_30,872 | Copy number  Discordant read pairs (40 read pairs)  Junction sequence (47 reads) |
|  |  | Homology-mediated translocation between chrV L *ura3-52* and chrV L *yel068c::URA3* | Copy number |
| 7 | PGSP4601  [bzg151] | Microhomology-mediated translocation chrV L 41,620 and 2-micron 2,464 | Discordant read pairs (880 read pairs)  Junction sequence (102 reads) |
|  |  | Inverted duplication in the 2 micron sequence | Copy number change in chrV |
|  |  | Homology-mediated translocation between chrV L *ura3-52* and chrXII R *YLRWTy1-2* | Copy number |
| 8 | PGSP4602  [bzg152] | Hairpin-mediated inversion chrV L 27,348_27,361 | Copy number  Discordant read pairs (95 read pairs)  Junction sequence (29 reads) |
|  |  | Homology-mediated translocation between chrV L *ura3-52* and chrV L *yel068c::URA3* | Copy number |
| 9 | PGSP4603  [bzg153] | Hairpin-mediated inversion chrV L 34,339-107_34,339-75 (in *can1::hisG*) | Copy number  Discordant read pairs (107 read pairs)  Junction sequence (282 reads) |
|  |  | Homology-mediated translocation between chrV L *ura3-52* and chrV L *yel068c::URA3* | Copy number |
| 10 | PGSP4604  [bzg154] | Hairpin-mediated inversion chrV L 34,339-107_34,339-75 (in *can1::hisG*) | Copy number  Discordant read pairs (117 read pairs)  Junction sequence (272 reads) |
|  |  | Homology-mediated translocation between chrV L *ura3-52* and chrXII R *YLRWTy1-2* | Copy number |
| 11 | PGSP4605  [bzg155] | circular chromosome:  microhomology-mediated translocation between chrV L 30,395 (in *AVT2*) and chrV R 556,943 (in *TOG1*) | Copy number  Discordant read pairs (662 read pairs)  Junction sequence (93 reads) |
| 12 | PGSP4606  [bzg156] | Hairpin-mediated inversion chrV L 25,817-568_25,817-546 (in *CAN1* insertion) | Copy number  Discordant read pairs (237 read pairs)  Junction sequence (27 reads) |
|  |  | Homology-mediated translocation between chrV L *ura3-52* and chrV L *yel068c::URA3* | Copy number |

**R.** GCR structures from *pol32Δ* uGCR strains

| ***No.*** | ***Sample**** | ***GCR description*** | ***GCR evidence***** |
| --- | --- | --- | --- |
| 1 | PGSP752  [bzg110] | Nonhomology-mediated interstitial deletion between chrV L 8,879 and chrV L 27,385 | Copy number  Discordant read pairs (560 read pairs)  Junction sequence (30 reads)  *hph^-^* |
| 2 | PGSP754  [bzg111] | *De novo* telomere addition chrV L 34,847_34,847 | Copy number  Discordant read pairs (82 read pairs)  Junction sequence (30 reads)  *hph^-^* |
| 3 | PGSP755  [bzg112] | *De novo* telomere addition chrV L 34,834_84,838 | Copy number  Discordant read pairs (75 read pairs)  Junction sequence (24 reads)  *hph^-^* |
| 4 | PGSP756  [bzg113] | *De novo* telomere addition chrV L 26,384_26,388 | Copy number  Discordant read pairs (137 read pairs)  Junction sequence (20 reads)  *hph^-^* |
| 5 | PGSP757  [bzg114] | *De novo* telomere addition chrV L 34,339-78_34,339-78 (in *can1::hisG*) | Copy number  Discordant read pairs (81 read pairs)  Junction sequence (142 reads)  *hph^-^* |
| 6 | PGSP758  [bzg115] | *De novo* telomere addition chrV L 34,842_34,847 | Copy number  Discordant read pairs (206 read pairs)  Junction sequence (43 reads)  *hph^-^* |
| 7 | PGSP4564  [bzg116] | *De novo* telomere addition chrV L 34,339-78_34,339-78 (in *can1::hisG*) | Copy number  Discordant read pairs (96 read pairs)  Junction sequence (139 reads)  *hph^-^* |
| 8 | PGSP4565  [bzg117] | *De novo* telomere addition chrV L 34,831_34,832 | Copy number  Discordant read pairs (124 read pairs)  Junction sequence (39 reads)  *hph^-^* |
| 9 | PGSP4566  [bzg118] | *De novo* telomere addition chrV L 25,817-1,263_25,817-1,263 (in *CAN1* insertion) | Copy number  Discordant read pairs (132 read pairs)  Junction sequence (58 reads)  *hph^-^* |
| 10 | PGSP4567  [bzg119] | *De novo* telomere addition chrV L 34,339-78_34,339-78 | Copy number  Discordant read pairs (117 read pairs)  Junction sequence (171 reads)  *hph^-^* |
| 11 | PGSP4568  [bzg120] | *De novo* telomere addition chrV L 39,682_39,685 | Copy number  Discordant read pairs (105 read pairs)  Junction sequence (60 reads)  *hph^-^* |

**S.** GCR structures from *sae2Δ pol32Δ* uGCR strains

| ***No.*** | ***Sample**** | ***GCR description*** | ***GCR evidence***** |
| --- | --- | --- | --- |
| 1 | PGSP4609 | Microhomology-mediated interstitial deletion between chrV L 15,946 and chrV L 36,019 | Copy number  Discordant read pairs (1111 read pairs)  Junction sequence (179 reads) |
| 2 | PGSP4610 | Hairpin-mediated inversion chrV L 34,339-107_34,339-75 (in *can1::hisG*) | Copy number  Discordant read pairs (82 read pairs)  Junction sequence (140 reads) |
|  |  | Homology-mediated translocation between chrV L *ura3-52* and chrXII R *YLRWTy1-2* | Copy number |
| 3 | PGSP4611 | Hairpin-mediated inversion chrV L 34,339-107_34,339-75 (in *can1::hisG*) | Copy number  Discordant read pairs (144 read pairs)  Junction sequence (244 reads) |
|  |  | Homology-mediated translocation between chrV L *ura3-52* and chrV L *yel068c::URA3* | Copy number |
| 4 | PGSP4612 | Microhomology-mediated translocation from chrV L 30,090 to repetitive subtelomeric region (for example chrXII 1,068,740) | Copy number  Junction sequence |
| 5 | PGSP4613 | *De novo* telomere addition chrV L 25,817-1,321_25,817-1,320 (in *CAN1* insertion) | Copy number  Discordant read pairs (228 read pairs)  Junction sequence (62 reads) |
| 6 | PGSP4614 | Hairpin-mediated inversion chrV L 34,339-107_34,339-75 (in *can1::hisG*) | Copy number  Discordant read pairs (116 read pairs)  Junction sequence (235 reads) |
|  |  | Homology-mediated translocation between chrV L *ura3-52* and the unannotated chrV R “*YERWdelta27”* (chrV:449,322..449,631; Nene et al. 2018) | Copy number |
| 7 | PGSP4615 | Hairpin-mediated inversion chrV L 34,339-107_34,339-75 (in *can1::hisG*) | Copy number  Discordant read pairs (180 read pairs)  Junction sequence (279 reads) |
|  |  | Homology-mediated translocation between chrV L *ura3-52* and chrV L *yel068c::URA3* | Copy number |
| 8 | PGSP4617 | Hairpin-mediated inversion chrV L 34,339-640_34,339-630 (in *can1::hisG*) | Copy number  Discordant read pairs (219 read pairs)  Junction sequence (46 reads) |
|  |  | Homology-mediated translocation between chrV L *ura3-52* and chrV L *yel068c::URA3* | Copy number |
| 9 | PGSP4618 | Hairpin-mediated inversion chrV L 34,339-107_34,339-75 (in *can1::hisG*) | Copy number  Discordant read pairs (102 read pairs)  Junction sequence (193 reads) |
|  |  | Homology-mediated translocation between chrV L *ura3-52* and chrXII R *YLRWTy1-2* | Copy number |
| 10 | PGSP4619 | Hairpin-mediated inversion chrV L 34,339-107_34,339-75 (in *can1::hisG*) | Copy number  Discordant read pairs (220 read pairs)  Junction sequence (323 reads) |
|  |  | Homology-mediated translocation between chrV L *ura3-52* and chrV L *yel068c::URA3* | Copy number |
| 11 | PGSP4620 | Hairpin-mediated inversion chrV L 34,339-107_34,339-75 (in *can1::hisG*) | Copy number  Discordant read pairs (168 read pairs)  Junction sequence (330 reads) |
|  |  | Homology-mediated translocation between chrV L *ura3-52* and chrV L *yel068c::URA3* | Copy number |
| 12 | PGSP4621 | Microhomology-mediated translocation from chrV L 36,135 (in *NPR2*) to chrV R 477,329 (in *BEM2*) | Copy number  Discordant read pairs (493 read pairs)  Junction sequence (69 reads) |
| 13 | PGSP4622 | Hairpin-mediated inversion chrV L 34,339-107_34,339-75 (in *can1::hisG*) | Copy number  Discordant read pairs (143 read pairs)  Junction sequence (255 reads) |
|  |  | *De novo* telomere addition chrV L 140,749_140,750 | Copy number  Discordant read pairs (160 read pairs)  Junction sequence (19 reads) |
| 14 | PGSP4623 | *De novo* telomere addition chrV L 34,888_34,898 | Copy number  Discordant read pairs (178 read pairs)  Junction sequence (40 reads) |
| 15 | PGSP4624 | Hairpin-mediated inversion chrV L 34,339-107_34,339-75 (in *can1::hisG*) | Copy number  Discordant read pairs (152 read pairs)  Junction sequence (252 reads) |
|  |  | Homology-mediated translocation between chrV L *YELWdelta1* and the unannotated chrV R “*YERWdelta27”* (chrV:449,322..449,631; Nene et al. 2018) | Copy number  Discordant read pairs (384 read pairs) |

**T.** GCR structures from *rad10Δ* uGCR strains

| ***No.*** | ***Sample**** | ***GCR description*** | ***GCR evidence***** |
| --- | --- | --- | --- |
| 1 | PGSP461  [bzg121] | *De novo* telomere addition chrV L 34,834_34,838 | Copy number  Discordant read pairs (133 read pairs)  Junction sequence (43 reads)  *hph^-^* |
| 2 | PGSP462  [bzg122] | *De novo* telomere addition chrV L 26,329_26,330 | Copy number  Discordant read pairs (66 read pairs)  Junction sequence (38 reads)  *hph^-^* |
| 3 | PGSP463  [bzg123] | *De novo* telomere addition chrV L 34,831_34,832 | Copy number  Discordant read pairs (78 read pairs)  Junction sequence (31 reads)  *hph^-^* |
| 4 | PGSP464  [bzg124] | *De novo* telomere addition chrV L 25,817-2,793_25,817-2,790 (in inserted *CAN1*) | Copy number  Discordant read pairs (70 read pairs)  Junction sequence (37 reads)  *hph^-^* |
| 5 | PGSP465  [bzg125] | *De novo* telomere addition chrV L 34,878_34,879 | Copy number  Discordant read pairs (92 read pairs)  Junction sequence (44 reads)  *hph^-^* |
| 6 | PGSP466  [bzg126] | *De novo* telomere addition chrV L 38,004_38,005 | Copy number  Discordant read pairs (128 read pairs)  Junction sequence (29 reads)  *hph^-^* |
| 7 | PGSP467  [bzg127] | *De novo* telomere addition chrV L 34,878_34,879 | Copy number  Discordant read pairs (119 read pairs)  Junction sequence (41 reads)  *hph^-^* |
| 8 | PGSP4576  [bzg128] | *De novo* telomere addition chrV L 34,888_34,898 | Copy number  Discordant read pairs (107 read pairs)  Junction sequence (24 reads)  *hph^-^* |
| 9 | PGSP4577  [bzg129] | *De novo* telomere addition chrV L 28,643_28,647 | Copy number  Discordant read pairs (139 read pairs)  Junction sequence (45 reads)  *hph^-^* |
| 10 | PGSP4578  [bzg130] | Microhomology-mediated interstitial deletion between chrV L 24,041 and chrV L 40,500 | Copy number  Discordant read pairs (623 read pairs)  Junction sequence (85 reads)  *hph^+^* |
| 11 | PGSP4579  [bzg131] | *De novo* telomere addition chrV L 26,401_26,403 | Copy number  Discordant read pairs (126 read pairs)  Junction sequence (36 reads)  *hph^-^* |
| 12 | PGSP4580  [bzg132] | Hairpin-mediated inversion chrV L 35,447_37,677 | Copy number  Discordant read pairs (654 read pairs)  Junction sequence (44 reads) |
|  |  | Homology-mediated translocation between chrV L *PAU2* and chrXI R *PAU15* | Copy number  Discordant read pairs (533 read pairs)  *hph^-^* |

**U.** GCR structures from *sae2Δ rad10Δ* uGCR strains

| ***No.*** | ***Sample**** | ***GCR description*** | ***GCR evidence***** |
| --- | --- | --- | --- |
| 1 | PGSP4581  [bzg133] | Hairpin-mediated inversion chrV L 34,339-107_34,339-75 (in *can1::hisG*) | Copy number  Discordant read pairs (61 read pairs)  Junction sequence (257 reads) |
|  |  | Homology-mediated translocation between chrV L *PAU2* and chrII L *PAU9* | Copy number  Discordant read pairs (107 read pairs) |
| 2 | PGSP4582  [bzg134] | Hairpin-mediated inversion chrV L 34,339-107_34,339-75 (in *can1::hisG*) | Copy number  Discordant read pairs (64 read pairs)  Junction sequence (237 reads) |
|  |  | Homology-mediated translocation between chrV L *ura3-52* and chrIII R *YCRWdelta8/9/10* | Copy number |
| 3 | PGSP4583  [bzg135] | Hairpin-mediated inversion chrV L 34,339-107_34,339-75 (in *can1::hisG*) | Copy number  Discordant read pairs (65 read pairs)  Junction sequence (195 reads) |
|  |  | Homology-mediated translocation between chrV L *ura3-52* and chrXVI R *YPRWTy1-3* | Copy number |
| 4 | PGSP4584  [bzg136] | Hairpin-mediated inversion chrV L 38,848_38,871 | Copy number  Discordant read pairs (48 read pairs)  Junction sequence (17 reads) |
|  |  | Homology-mediated translocation between chrV L *ura3-52* and chrXVI R *YPRWTy1-3* | Copy number |
| 5 | PGSP4585  [bzg137] | Hairpin-mediated inversion chrV L 35,478_35,494 | Copy number  Discordant read pairs (101 read pairs)  Junction sequence (56 reads) |
|  |  | Homology-mediated translocation between chrV L *ura3-52* and unknown target | Copy number |
| 6 | PGSP4586  [bzg138] | Hairpin-mediated inversion chrV L 34,339-107_34,339-75 (in *can1::hisG*) | Copy number  Discordant read pairs (74 read pairs)  Junction sequence (204 reads) |
|  |  | Homology-mediated translocation between chrV L *ura3-52* and chrXIV L *YNLCTy1-1* | Copy number |
| 7 | PGSP4587  [bzg139] | Hairpin-mediated inversion chrV L 25,817-568_25,817-546 (in *CAN1* insertion) | Copy number  Discordant read pairs (424 read pairs)  Junction sequence (74 reads) |
|  |  | Hairpin-mediated inversion chrV L 84,266_84,283 | Copy number  Discordant read pairs (143 read pairs)  Junction sequence (86 reads) |
|  |  | Hairpin-mediated inversion chrV L 25,817-568_25,817-546 (in *CAN1* insertion) | Copy number  Discordant read pairs (424 read pairs)  Junction sequence (74 reads) |
|  |  | Non-homology interstitial deletion of the second copy of *CEN5* chrV L 151,043 to chrV R 153,003 | Copy number  Discordant read pairs (462 read pairs)  Junction sequence (82 reads) |
| 8 | PGSP4588  [bzg140] | Hairpin-mediated inversion chrV L 25,817-592_25,817-570 (in *CAN1* insertion) | Copy number  Discordant read pairs (237 read pairs)  Junction sequence (100 reads) |
|  |  | Homology-mediated translocation between chrV L *YELCdelta4* and chrII L *YBLWdelta1/YBLWdelta2* | Copy number  Discordant read pairs (263 read pairs) |
| 9 | PGSP4589  [bzg141] | Hairpin-mediated inversion chrV L 34,339-1,091_34,339-1,066 (in *can1::hisG*) | Copy number  Discordant read pairs (237 read pairs)  Junction sequence (422 reads) |
|  |  | Homology-mediated translocation between chrV L *YELCdelta4* and chrII L *YBLWdelta1/YBLWdelta2* | Copy number  Discordant read pairs (263 read pairs) |
| 10 | PGSP4590  [bzg142] | Hairpin-mediated inversion chrV L 26,572_28,017 | Copy number  Discordant read pairs (676 read pairs)  Junction sequence (87 reads) |
|  |  | Homology-mediated translocation between chrV L *PAU2* and chrXII L *PAU18* | Copy number  Discordant read pairs (118 read pairs) |
| 11 | PGSP4591  [bzg143] | Hairpin-mediated inversion chrV L 26,466_26,538 | Copy number  Discordant read pairs (315 read pairs)  Junction sequence (97 reads) |
|  |  | Homology-mediated translocation between chrV L *YELWdelta1* and chrXV L *YOLWTy1-1* | Copy number  Discordant read pairs (53 read pairs) |
|  |  | Homology-mediated translocation between chrXV L *YOLWTy1-1* and chrIII R *YCRWdelta11* | Copy number |
| 12 | PGSP4592  [bzg144] | Microhomology-mediated translocation between chrV L 34,522 (in *NPR2*) and chrVII L 251,283 (in *SEC27*) | Copy number  Discordant read pairs (582 read pairs)  Junction sequence (36 reads) |

**V.** GCR structures from *mus81Δ* uGCR strains

| ***No.*** | ***Sample**** | ***GCR description*** | ***GCR evidence***** |
| --- | --- | --- | --- |
| 1 | PGSP222 | *De novo* telomere addition chrV L 28,683_28,685 | Copy number  Discordant read pairs (267 read pairs)  Junction sequence (61 reads)  *hph^-^* |
| 2 | PGSP223 | *De novo* telomere addition chrV L 35,542_35,545 | Copy number  Discordant read pairs (162 read pairs)  Junction sequence (28 reads)  *hph^-^* |
| 3 | PGSP224 | *De novo* telomere addition chrV L 41,207_41,209 | Copy number  Discordant read pairs (320 read pairs)  Junction sequence (57 reads)  *hph^-^* |
| 4 | PGSP225 | *De novo* telomere addition chrV L 35,187_35,193 | Copy number  Discordant read pairs (154 read pairs)  Junction sequence (53 reads)  *hph^-^* |
| 5 | PGSP226 | *De novo* telomere addition chrV L 28,840_28,841 | Copy number  Discordant read pairs (302 read pairs)  Junction sequence (87 reads)  *hph^-^* |
| 6 | PGSP227 | *De novo* telomere addition chrV L 26,384_26,388 | Copy number  Discordant read pairs (231 read pairs)  Junction sequence (87 reads)  *hph^-^* |
| 7 | PGSP228 | *De novo* telomere addition chrV L 36,214_36,216 | Copy number  Discordant read pairs (194 read pairs)  Junction sequence (34 reads)  *hph^-^* |
| 8 | PGSP229 | *De novo* telomere addition chrV L 39,749_39750 | Copy number  Discordant read pairs (175 read pairs)  Junction sequence (43 reads)  *hph^-^* |
| 9 | PGSP230 | *De novo* telomere addition chrV L 41,578_41,582 | Copy number  Discordant read pairs (135 read pairs)  Junction sequence (31 reads)  *hph^-^* |
| 10 | PGSP231 | *De novo* telomere addition chrV L 25,817-2,208_25,817-2,204 (in *CAN1* insertion) | Copy number  Discordant read pairs (184 read pairs)  Junction sequence (20 reads)  *hph^-^* |
| 11 | PGSP232 | Microhomology-mediated translocation between chrV L 39,999 (in *PRB1*) and chrXII R 459,803 (in rDNA repeats) | Copy number  Discordant read pairs (644 read pairs)  Junction sequence (220 reads)  *hph^-^* |
| 12 | PGSP233 | *De novo* telomere addition chrV L 39,155_39,157 | Copy number  Discordant read pairs (190 read pairs)  Junction sequence (70 reads)  *hph^-^* |
|  |  | Unrelated to GCR: Disomy chrVIII | Copy number |

**W.** GCR structures from *sae2Δ mus81Δ* uGCR strains

| ***No.*** | ***Sample**** | ***GCR description*** | ***GCR evidence***** |
| --- | --- | --- | --- |
| 1 | PGSP4649 | *De novo* telomere addition chrV L 34,842_34,847 | Copy number  Discordant read pairs (33 read pairs)  Junction sequence (10 reads) |
| 2 | PGSP4650 | Microhomology-mediated translocation between chrV L 35,141 (in *NPR2*) and chrI L 135,925 (in *SPO7*) | Copy number  Discordant read pairs (203 read pairs)  Junction sequence (32 reads) |
| 3 | PGSP4651 | Hairpin-mediated inversion chrV L 34,339-107_34,339-75 (in *can1::hisG*) | Copy number  Discordant read pairs (119 read pairs)  Junction sequence (163 reads) |
|  |  | Homology-mediated translocation between chrV L *YELWdelta6* and chrXVI R *YPRWTy1-3* | Copy number  Discordant read pairs (264 read pairs) |
| 4 | PGSP4652 | Hairpin-mediated inversion chrV L 34,339-107_34,339-75 (in *can1::hisG*) | Copy number  Discordant read pairs (191 read pairs)  Junction sequence (272 reads) |
|  |  | Homology-mediated translocation between chrV L *ura3-52* and chrV L *yel068c::URA3* | Copy number |
| 5 | PGSP4653 | *De novo* telomere addition chrV L 40,403_40,406 | Copy number  Discordant read pairs (66 read pairs)  Junction sequence (17 reads) |
| 6 | PGSP4654 | Hairpin-mediated inversion chrV L 34,339-107_34,339-75 (in *can1::hisG*) | Copy number  Discordant read pairs (125 read pairs)  Junction sequence (202 reads) |
|  |  | Homology-mediated translocation between chrV L *ura3-52* and chrV L *yel068c::URA3* | Copy number |
| 7 | PGSP4655 | *De novo* telomere addition chrV L 36,424_36,430 | Copy number  Discordant read pairs (120 read pairs)  Junction sequence (44 reads) |
| 8 | PGSP4656 | Non-homology translocation between chrV L 35,652 (in *NPR2*) and an unknown full-length Ty element | Copy number  Junction sequence (735 reads) |
|  |  | Homology-mediated translocation between the full-length Ty element and chrIII R *YCRWdelta 8/9/10* | Copy number |
| 9 | PGSP4657 | Hairpin-mediated inversion chrV L 34,339-107_34,339-75 (in *can1::hisG*) | Copy number  Discordant read pairs (171 read pairs)  Junction sequence (206 reads) |
|  |  | Homology-mediated translocation between chrV L *ura3-52* and chrXII R *YLRWTy1-2* | Copy number |
| 10 | PGSP4658 | Hairpin-mediated inversion chrV L 34,339-107_34,339-75 (in *can1::hisG*) | Copy number  Discordant read pairs (123 read pairs)  Junction sequence (182 reads) |
|  |  | Homology-mediated translocation between chrV L *ura3-52* and chrV L *yel068c::URA3* | Copy number |
| 11 | PGSP4659 | Hairpin-mediated inversion chrV L 34,339-107_34,339-75 (in *can1::hisG*) | Copy number  Discordant read pairs (148 read pairs)  Junction sequence (204 reads) |
|  |  | Homology-mediated translocation between chrV L *ura3-52* and chrXII R *YLRWTy1-3* | Copy number |
| 12 | PGSP4660 | *De novo* telomere addition chrV L 34,834_34,838 | Copy number  Discordant read pairs (118 read pairs)  Junction sequence (21 reads) |
| 13 | PGSP4661 | *De novo* telomere addition chrV L 30,823_30,827 | Copy number  Discordant read pairs (32 read pairs)  Junction sequence (21 reads) |
| 14 | PGSP4662 | Hairpin-mediated inversion chrV L 34,339-107_34,339-75 (in *can1::hisG*) | Copy number  Discordant read pairs (125 read pairs)  Junction sequence (171 reads) |
|  |  | Homology-mediated translocation between chrV L *ura3-52* and chrV L *yel068c::URA3* | Copy number |
| 15 | PGSP4663 | Microhomology-mediated translocation between chrV L 31,124 (in *CAN1* insertion) and chrXIV L 333,145 (in *CBK1*) | Copy number  Discordant read pairs (79 read pairs)  Junction sequence (13 reads) |
| 16 | PGSP4664 | Hairpin-mediated inversion chrV L 34,339-107_34,339-75 (in *can1::hisG*) | Copy number  Discordant read pairs (70 read pairs)  Junction sequence (123 reads) |
|  |  | Homology-mediated translocation between chrV L *YELWdelta1* and chrV R *YDRWTy1-5* | Copy number |

**X.** GCR structures from *sae2Δ mus81Δ* chrV:25,817-1,749 gRNA uGCR strains

| ***No.*** | ***Sample**** | ***GCR description*** | ***GCR evidence***** |
| --- | --- | --- | --- |
| 1 | PGSP5074 | Microhomology-mediated interstitial deletion from chrV L 25,817-1,747 (in inserted *CAN1*) to chrV L 116,323 (in inserted *URA3*) | Copy number  Discordant read pairs (330 read pairs)  Junction sequence (61 reads) |
| 2 | PGSP5075 | Microhomology-mediated interstitial deletion from chrV L 22,578 to chrV L 27,619 | Copy number  Discordant read pairs (174 read pairs)  Junction sequence (27 reads) |
| 3 | PGSP5076 | Microhomology-mediated translocation from chrV L 25,817-1,750 (in inserted *CAN1*) to chrIII R 174,064 | Copy number  Discordant read pairs (383 read pairs)  Junction sequence (42 reads) |
| 4 | PGSP5077 | Nonhomology-mediated translocation from chrV L 25,817-1,749 (in inserted *CAN1*) to chrXV L 130,138 | Copy number  Discordant read pairs (254 read pairs)  Junction sequence (44 reads) |
| 5 | PGSP5078 | De novo telomere addition chrV L 25,817-636_25,817-635 (in inserted *CAN1*) | Copy number  Discordant read pairs (103+96 read pairs)  Junction sequence (44 reads) |
| 6 | PGSP5079 | Microhomology-mediated translocation from chrV L 25,817-1,751 (in inserted *CAN1*) to chrXV R 1,027,898 | Copy number  Discordant read pairs (194 read pairs)  Junction sequence (24 reads) |
| 7 | PGSP5080 | Hairpin-mediated inversion chrV L 34,339-107_34,339-75 (in *can1::hisG*) | Copy number  Discordant read pairs (209 read pairs)  Junction sequence (24 reads) |
|  |  | Homology-mediated translocation between chrV L *ura3-52* and chrV L *yel068c::URA3* | Copy number |
| 8 | PGSP5081 | Nonhomology-mediated translocation from chrV L 25,817-1,711 (in inserted *CAN1*) and chrXII R 459,512 (in the rDNA repeats) | Copy number  Discordant read pairs (125 read pairs)  Junction sequence (24 reads) |
| 9 | PGSP5082 | Hairpin-mediated inversion chrV L 34,339-107_34,339-75 (in *can1::hisG*) | Copy number  Discordant read pairs (193 read pairs)  Junction sequence (293 reads) |
|  |  | Homology-mediated translocation between chrV L *ura3-52* and chrV L *yel068c::URA3* | Copy number |
| 10 | PGSP5083 | Microhomology-mediated translocation from chrV L 25,817-1,711 (in inserted *CAN1*) and chrVII L 145,950 | Copy number  Discordant read pairs (285 read pairs)  Junction sequence (34 reads) |
| 11 | PGSP5084 | Hairpin-mediated inversion chrV L 34,339-107_34,339-75 (in *can1::hisG*) | Copy number  Discordant read pairs (165 read pairs)  Junction sequence (34 reads) |
|  |  | Homology-mediated translocation between chrV L *ura3-52* and chrX L *YJLCdelta3* | Copy number |
| 12 | PGSP5085 | Nonhomology-mediated translocation from chrV L 25,817-1,750 (in inserted *CAN1*) and chrIV L 79,993 | Copy number  Discordant read pairs (393 read pairs)  Junction sequence (44 reads) |

**Y.** GCR structures from *slx1Δ* uGCR strains

| ***No.*** | ***Sample**** | ***GCR description*** | ***GCR evidence***** |
| --- | --- | --- | --- |
| 1 | PGSP4904 | *De novo* telomere addition chrV L 34,834_34,847 | Copy number  Discordant read pairs (63 read pairs)  Junction sequence (123 reads) |
| 2 | PGSP4905 | Microhomology-mediated interstitial deletion between chrV L 25,256 and chrV L 34,339-80 | Copy number  Discordant read pairs (168 read pairs)  Junction sequence (76 reads) |
| 3 | PGSP4907 | *De novo* telomere addition chrV L 25,817-2220_25,817-2,214 | Copy number  Discordant read pairs (51 read pairs)  Junction sequence (3 reads) |
| 4 | PGSP4908 | Microhomology-mediated interstitial deletion between chrV L 363 (or other subtelomeric region) and chrV L 40,995 | Copy number  Discordant read pairs (139 read pairs)  Junction sequence (6 reads) |
| 5 | PGSP4909 | Microhomology-mediated interstitial deletion between chrV L 528 (or other subtelomeric region) and chrV L 40,375 | Copy number  Discordant read pairs (73 read pairs)  Junction sequence (6 reads) |
| 6 | PGSP4910 | *De novo* telomere addition chrV L 27,423_27,427 | Copy number  Discordant read pairs (47 read pairs)  Junction sequence (6 reads) |
| 7 | PGSP4912 | *De novo* telomere addition chrV L 26,401_26,403 | Copy number  Discordant read pairs (42 read pairs)  Junction sequence (7 reads) |
| 8 | PGSP4913 | *De novo* telomere addition chrV L 41,578_41,582 | Copy number  Discordant read pairs (55 read pairs)  Junction sequence |
| 9 | PGSP4914 | Microhomology-mediated interstitial deletion between chrV L 23,966 and chrV L 27,113 | Copy number  Discordant read pairs (170 read pairs)  Junction sequence (13 reads) |
| 10 | PGSP4915 | Microhomology-mediated interstitial deletion between chrV L 3,634 (or other subtelomeric region) and chrV L 38,081 | Copy number  Discordant read pairs (64 read pairs)  Junction sequence |
| 11 | PGSP4916 | *De novo* telomere addition chrV L 30,170_30,176 | Copy number  Discordant read pairs (76 read pairs)  Junction sequence (8 reads) |
| 12 | PGSP4917 | Microhomology-mediated interstitial deletion between chrV L 528 (or other subtelomeric region) and chrV L 40,375 | Copy number  Discordant read pairs (79 read pairs)  Junction sequence (6 reads) |

**Z.** GCR structures from *sae2Δ slx1Δ* uGCR strains

| ***No.*** | ***Sample**** | ***GCR description*** | ***GCR evidence***** |
| --- | --- | --- | --- |
| 1 | PGSP4864 | Hairpin-mediated inversion chrV L 34,339-107_34,339-75 (in *can1::hisG*) | Copy number  Discordant read pairs (102 read pairs)  Junction sequence (129 reads) |
|  |  | Homology-mediated translocation between chrV L *ura3-52* and chrX R *YJRWTy1-1/YJRWTy1-2* | Copy number |
| 2 | PGSP4865 | Hairpin-mediated inversion chrV L 34,339-107_34,339-75 (in *can1::hisG*) | Copy number  Discordant read pairs (101 read pairs)  Junction sequence (157 reads) |
|  |  | Homology-mediated translocation between chrV L *ura3-52* and chrX R *YJRWTy1-1/YJRWTy1-2* | Copy number |
| 3 | PGSP4866 | Hairpin-mediated inversion chrV L 34,339-107_34,339-75 (in *can1::hisG*) | Copy number  Discordant read pairs (80 read pairs)  Junction sequence (118 reads) |
|  |  | Homology-mediated translocation between chrV L *ura3-52* and chrIII R *YCRWdelta8/9/10* | Copy number |
| 4 | PGSP4867 | Hairpin-mediated inversion chrV L 34,339-107_34,339-75 (in *can1::hisG*) | Copy number  Discordant read pairs (72 read pairs)  Junction sequence (109 reads) |
|  |  | Homology-mediated translocation between chrV L *ura3-52* and chrV L *yel068c::URA3* | Copy number |
| 5 | PGSP4869 | Hairpin-mediated inversion chrV L 34,339-107_34,339-75 (in *can1::hisG*) | Copy number  Discordant read pairs (100 read pairs)  Junction sequence (170 reads) |
|  |  | Homology-mediated translocation between chrV L *YELWdelta1* and chrX L *YJLCdelta3* | Copy number |
| 6 | PGSP4870 | Hairpin-mediated inversion chrV L 34,339-107_34,339-75 (in *can1::hisG*) | Copy number  Discordant read pairs (151 read pairs)  Junction sequence (185 reads) |
|  |  | Microhomology-mediated translocation between chrV L 141,668 and chrX R 474,022 (*YJRWTy1-1*) | Copy number  Discordant read pairs (371 read pairs)  Junction sequence (24 reads) |
| 7 | PGSP4871 | Hairpin-mediated inversion chrV L 34,339-107_34,339-75 (in *can1::hisG*) | Copy number  Discordant read pairs (93 read pairs)  Junction sequence (154 reads) |
|  |  | Homology-mediated translocation between chrV L *ura3-52* and chrIII R *YCRWdelta11* | Copy number |
| 8 | PGSP4872 | Hairpin-mediated inversion chrV L 34,339-107_34,339-75 (in *can1::hisG*) | Copy number  Discordant read pairs (88 read pairs)  Junction sequence (139 reads) |
|  |  | Homology-mediated translocation between chrV L *ura3-52* and chrX R *YJRWTy1-1/YJRWTy1-2* | Copy number |
| 9 | PGSP4873 | Hairpin-mediated inversion chrV L 27,074_27,102 | Copy number  Discordant read pairs (210 read pairs)  Junction sequence (54 reads) |
|  |  | Homology-mediated translocation between chrV L *PAU2* and another subtelomeric *PAU* sequence | Copy number  Discordant read pairs (425 read pairs) |
| 10 | PGSP4874 | Hairpin-mediated inversion chrV L 29,513_29,529 | Copy number  Discordant read pairs (169 read pairs)  Junction sequence (41 reads) |
|  |  | Homology-mediated translocation between chrV L *ura3-52* and chrX R *YJRWTy1-1/YJRWTy1-2* | Copy number |
| 11 | PGSP4875 | Hairpin-mediated inversion chrV L 34,339-107_34,339-75 (in *can1::hisG*) | Copy number  Discordant read pairs (88 read pairs)  Junction sequence (205 reads) |
|  |  | Homology-mediated translocation between chrV L *YELCdelta4* and chrVIII R *YHRCdelta16* | Copy number  Discordant read pairs (56 read pairs) |
| 12 | PGSP4876 | Hairpin-mediated inversion chrV L 34,339-107_34,339-75 (in *can1::hisG*) | Copy number  Discordant read pairs (93 read pairs)  Junction sequence (147 reads) |
|  |  | Homology-mediated translocation between chrV L *ura3-52* and chrV L *yel068c::URA3* | Copy number |

**AA.** GCR structures from *yen1Δ* uGCR strains

| ***No.*** | ***Sample**** | ***GCR description*** | ***GCR evidence***** |
| --- | --- | --- | --- |
| 1 | PGSP4891 | Inversion chrV L 38,270; cannot be explained by a simple DNA hairpin with a stable stem-loop. | Copy number  Discordant read pairs (135 read pairs)  Junction sequence (25 reads) |
|  |  | Homology-mediated translocation between chrV L *ura3-52* and chrV L *yel068c::URA3* | Copy number |
| 2 | PGSP4892 | Hairpin-mediated inversion chrV L 29,503_29,539 | Copy number  Discordant read pairs (168 read pairs)  Junction sequence (48 reads) |
|  |  | Homology-mediated translocation between chrV L *ura3-52* and chrXVI R *YPRWTy1-3* | Copy number |
| 3 | PGSP4893 | *De novo* telomere addition chrV L 42,064_42,066 | Copy number  Discordant read pairs (101 read pairs)  Junction sequence (10 reads) |
| 4 | PGSP4894 | *De novo* telomere addition chrV L 25,817-491_25,817-490 (in inserted *CAN1*) | Copy number  Discordant read pairs (127 read pairs)  Junction sequence (15 reads) |
| 5 | PGSP4895 | *De novo* telomere addition chrV L 34,839_34,847 | Copy number  Discordant read pairs (129 read pairs)  Junction sequence (31 reads) |
| 6 | PGSP4896 | *De novo* telomere addition chrV L 34,868_34,870 | Copy number  Discordant read pairs (64 read pairs)  Junction sequence (11 reads) |
| 7 | PGSP4897 | Hairpin-mediated inversion chrV L 34,759_36,909 | Copy number  Discordant read pairs (310 read pairs)  Junction sequence (48 reads) |
|  |  | Homology-mediated translocation between chrV L *ura3-52* and chrIII R *YCRWdelta13* | Copy number |
| 8 | PGSP4898 | *De novo* telomere addition chrV L 27,932_27,934 | Copy number  Discordant read pairs (80 read pairs)  Junction sequence (18 reads) |
| 9 | PGSP4899 | Hairpin-mediated inversion chrV L 40,994_43,019 | Copy number  Discordant read pairs (307 read pairs)  Junction sequence (44 reads) |
|  |  | Homology-mediated translocation between chrV L *ura3-52* and and the unannotated chrV R “*YERWdelta27”* (chrV:449,322..449,631; Nene et al. 2018) | Copy number |
| 10 | PGSP4900 | Microhomology-mediated translocation between chrV L 41,236 and chrXIV R 709,088 | Copy number  Discordant read pairs (246 read pairs)  Junction sequence (74 reads) |
| 11 | PGSP4901 | *De novo* telomere addition chrV L 34,339-58_34,339-55 (in inserted *can1::hisG*) | Copy number  Discordant read pairs (86 read pairs)  Junction sequence (74 reads) |

**AB.** GCR structures from *sae2Δ yen1Δ* uGCR strains

| ***No.*** | ***Sample**** | ***GCR description*** | ***GCR evidence***** |
| --- | --- | --- | --- |
| 1 | PGSP4849 | Hairpin-mediated inversion chrV L 34,339-107_34,339-75 (in *can1::hisG*) | Copy number  Discordant read pairs (88 read pairs)  Junction sequence (162 reads) |
|  |  | Homology-mediated translocation between chrV L *ura3-52* and chrX R *YJRWTy1-1/YJRWTy1-2* | Copy number |
| 2 | PGSP4850 | Hairpin-mediated inversion chrV L 34,339-107_34,339-75 (in *can1::hisG*) | Copy number  Discordant read pairs (66 read pairs)  Junction sequence (147 reads) |
|  |  | Homology-mediated translocation between chrV L *ura3-52* and chrVII R *YGRWTy1-1* | Copy number |
| 3 | PGSP4851 | Hairpin-mediated inversion chrV L 34,339-107_34,339-75 (in *can1::hisG*) | Copy number  Discordant read pairs (72 read pairs)  Junction sequence (125 reads) |
|  |  | Homology-mediated translocation between chrV L *ura3-52* and chrX R *YJRWTy1-1/YJRWTy1-2* | Copy number |
| 4 | PGSP4852 | Hairpin-mediated inversion chrV L 34,339-107_34,339-75 (in *can1::hisG*) | Copy number  Discordant read pairs (45 read pairs)  Junction sequence (135 reads) |
|  |  | Homology-mediated translocation between chrV L *ura3-52* and chrXII R *YLRWdelta11* | Copy number  Discordant read pairs (40 read pairs) |
| 5 | PGSP4853 | Hairpin-mediated inversion chrV L 34,339-107_34,339-75 (in *can1::hisG*) | Copy number  Discordant read pairs (171 read pairs)  Junction sequence (377 reads) |
|  |  | Microhomology-mediated interstitial deletion of the region including *CEN5* between chrV L 153,677 and chrV 153,642 | Copy number  Discordant read pairs (731 read pairs)  Junction sequence (88 reads) |
| 6 | PGSP4854 | Hairpin-mediated inversion chrV L 34,339-107_34,339-75 (in *can1::hisG*) | Copy number  Discordant read pairs (75 read pairs)  Junction sequence (225 reads) |
|  |  | Homology-mediated translocation between chrV L *ura3-52* and chrXVI R *YPRWTy1-3* | Copy number |
| 7 | PGSP4855 | Hairpin-mediated inversion chrV L 34,339-107_34,339-75 (in *can1::hisG*) | Copy number  Discordant read pairs (130 read pairs)  Junction sequence |
|  |  | Homology-mediated translocation between chrV L *ura3-52* and chrIV R *YDRWTy1-4* | Copy number |
| 8 | PGSP4856 | Hairpin-mediated inversion chrV L 34,339-107_34,339-75 (in *can1::hisG*) | Copy number  Discordant read pairs (70 read pairs)  Junction sequence |
|  |  | Homology-mediated translocation between chrV L *ura3-52* and chrXII R *YLRWTy1-3* | Copy number |
| 9 | PGSP4857 | Hairpin-mediated inversion chrV L 34,339-107_34,339-75 (in *can1::hisG*) | Copy number  Discordant read pairs (98 read pairs)  Junction sequence (236 reads) |
|  |  | Homology-mediated translocation between chrV L *ura3-52* and chrIII R *YCRWdelta11* | Copy number |
| 10 | PGSP4858 | Hairpin-mediated inversion chrV L 34,339-91_34,339-46 (in *can1::hisG*) | Copy number  Discordant read pairs (218 read pairs)  Junction sequence |
|  |  | Homology-mediated translocation between chrV L *ura3-52* and the unannotated chrV R “*YERWdelta27”* (chrV:449,322..449,631; Nene et al. 2018) | Copy number |
| 11 | PGSP4859 | Hairpin-mediated inversion chrV L 34,339-107_34,339-75 (in *can1::hisG*) | Copy number  Discordant read pairs (102 read pairs)  Junction sequence (220 reads) |
|  |  | Homology-mediated translocation between chrV L *ura3-52* and chrIV R *YDRWTy1-4* | Copy number |
| 12 | PGSP4860 | Hairpin-mediated inversion chrV L 34,339-107_34,339-75 (in *can1::hisG*) | Copy number  Discordant read pairs (107 read pairs)  Junction sequence (140 reads) |
|  |  | Homology-mediated translocation between chrV L *ura3-52* and chrIII R *YCRWdelta8/9/10* | Copy number |

**AC.** GCR structures from *pif1Δ* uGCR strains

| ***No.*** | ***Sample**** | ***GCR description*** | ***GCR evidence***** |
| --- | --- | --- | --- |
| 1 | PGSP2214 | *De novo* telomere addition chrV L 39,764_39,771 | Copy number  Discordant read pairs (63 read pairs)  Junction sequence (24 reads)  *hph^-^* |
| 2 | PGSP2215 | *De novo* telomere addition chrV L 40,726_40,726 | Copy number  Discordant read pairs (85 read pairs)  Junction sequence (26 reads)  *hph^-^* |
| 3 | PGSP2216 | *De novo* telomere addition chrV L 39,251_39,252 | Copy number  Discordant read pairs (55 read pairs)  Junction sequence (28 reads)  *hph^-^* |
| 4 | PGSP2217 | *De novo* telomere addition chrV L 38,297_38,300 | Copy number  Discordant read pairs (110 read pairs)  Junction sequence (40 reads)  *hph^-^* |
| 5 | PGSP2218 | *De novo* telomere addition chrV L 41,538_41,539 | Copy number  Discordant read pairs (124 read pairs)  Junction sequence (33 reads)  *hph^-^* |
| 6 | PGSP2219 | *De novo* telomere addition chrV L 37,086_37,089 | Copy number  Discordant read pairs (86 read pairs)  Junction sequence (22 reads)  *hph^-^* |
| 7 | PGSP2220 | *De novo* telomere addition chrV L 40,758_40,762 | Copy number  Discordant read pairs (66 read pairs)  Junction sequence (24 reads)  *hph^-^* |
| 8 | PGSP2221 | *De novo* telomere addition chrV L 39,765_39,771 | Copy number  Discordant read pairs (83 read pairs)  Junction sequence (19 reads)  *hph^-^* |
| 9 | PGSP2222 | *De novo* telomere addition chrV L 39,363_39,369 | Copy number  Discordant read pairs (64 read pairs)  Junction sequence (32 reads)  *hph^-^* |
| 10 | PGSP2223 | *De novo* telomere addition chrV L 39,762_39,771 | Copy number  Discordant read pairs (97 read pairs)  Junction sequence (26 reads)  *hph^-^* |
| 11 | PGSP2224 | *De novo* telomere addition chrV L 39,659_39,264 | Copy number  Discordant read pairs (74 read pairs)  Junction sequence (36 reads)  *hph^-^* |
| 12 | PGSP2225 | *De novo* telomere addition chrV L 38,086_38,091 | Copy number  Discordant read pairs (107 read pairs)  Junction sequence (36 reads)  *hph^-^* |

**AD.** GCR structures from *sae2Δ pif1Δ* uGCR strains

| ***No.*** | ***Sample**** | ***GCR description*** | ***GCR evidence***** |
| --- | --- | --- | --- |
| 1 | PGSP4703 | *De novo* telomere addition chrV L 27,423_27,427 | Copy number  Discordant read pairs (74 read pairs)  Junction sequence (36 reads) |
| 2 | PGSP4704 | Hairpin-mediated inversion chrV L 34,339-107_34,339-75 (in *can1::hisG*) | Copy number  Discordant read pairs (119 read pairs)  Junction sequence (187 reads) |
|  |  | *De novo* telomere addition chrV L 107,567_107,571 | Copy number  Discordant read pairs (56 read pairs)  Junction sequence (16 reads) |
| 3 | PGSP4705 | *De novo* telomere addition chrV L 26,384_26,388 | Copy number  Discordant read pairs (60 read pairs)  Junction sequence (35 reads) |
| 4 | PGSP4706 | *De novo* telomere addition chrV L 38,543_38,545 | Copy number  Discordant read pairs (76 read pairs)  Junction sequence (21 reads) |
| 5 | PGSP4707 | *De novo* telomere addition chrV L 34,339-59_34,339-55 (in *can1::hisG* insertion) | Copy number  Discordant read pairs (146 read pairs)  Junction sequence (21 reads) |
| 6 | PGSP4709 | *De novo* telomere addition chrV L 34,339-645_34,339-643 (in *can1::hisG* insertion) | Copy number  Discordant read pairs (106 read pairs)  Junction sequence (21 reads) |
| 7 | PGSP4710 | *De novo* telomere addition chrV L 30,742_30,745 | Copy number  Discordant read pairs (63 read pairs)  Junction sequence (12 reads) |
| 8 | PGSP4711 | *De novo* telomere addition chrV L 34,339-645_34,339-643 (in *can1::hisG* insertion) | Copy number  Discordant read pairs (94 read pairs)  Junction sequence (20 reads) |
| 9 | PGSP4713 | *De novo* telomere addition chrV L 39,764_39,771 | Copy number  Discordant read pairs (73 read pairs)  Junction sequence (56 reads) |
| 10 | PGSP4714 | *De novo* telomere addition chrV L 40,197_40,204 | Copy number  Discordant read pairs (108 read pairs)  Junction sequence (30 reads) |
| 11 | PGSP4715 | *De novo* telomere addition chrV L 29,511_29,513 | Copy number  Discordant read pairs (79 read pairs)  Junction sequence (30 reads) |
| 12 | PGSP4716 | *De novo* telomere addition chrV L 30,742_30,745 | Copy number  Discordant read pairs (87 read pairs)  Junction sequence (18 reads) |

**AE.** GCR structures from *yku80Δ* uGCR strains

| ***No.*** | ***Sample**** | ***GCR description*** | ***GCR evidence***** |
| --- | --- | --- | --- |
| 1 | PGSP3606 | Microhomology-mediated translocation between chrV L 31,733 (in *CAN1* insertion) and chrV R 466,011 | Copy number  Discordant read pairs (368 read pairs)  Junction sequence (41 reads) |
| 2 | PGSP3607 | Microhomology-mediated interstitial deletion between chrV L 13,549 and 31,202 (in *CAN1* insertion) | Copy number  Discordant read pairs (229 read pairs)  Junction sequence (57 reads) |
| 3 | PGSP3608 | Microhomology-mediated interstitial deletion chrV L 5473 to chrV L 27270 | Copy number  Junction sequence (951 reads) |
| 4 | PGSP3609 | Hairpin-mediated inversion chrV L 35,889_35,902 | Copy number  Discordant read pairs (111 read pairs)  Junction sequence (19 reads) |
|  |  | Homology-mediated translocation between chrV L *ura3-52* and the unannotated chrV R “*YERWdelta27”* (chrV:449,322..449,631; Nene et al. 2018) | Copy number  Discordant read pairs (136 read pairs)  Junction sequence (42 reads) |
| 5 | PGSP3610 | Hairpin-mediated inversion chrV L 34,339-107_34,339-75 (in *can1::hisG*) | Copy number  Discordant read pairs (34 read pairs)  Junction sequence (116 reads) |
|  |  | Homology-mediated translocation between chrV L *ura3-52* and chrV L *yel068c::URA3* | Copy number |
| 6 | PGSP3611 | Hairpin-mediated inversion chrV L 34,339-871_34,339-814 (in *can1::hisG*) | Copy number  Discordant read pairs (152 read pairs)  Junction sequence (58 reads) |
|  |  | Homology-mediated translocation between chrV L *can1::hisG* and chrXV R *ade2Δ::hisG* | Copy number |
|  |  | Unrelated to GCR: disomy of chrVIII |  |
| 7 | PGSP3612 | Microhomology-mediated translocation between chrV L 36,848 and chrX L 81,482 | Copy number  Discordant read pairs (529 read pairs)  Junction sequence (87 reads) |
| 8 | PGSP3613 | Microhomology-mediated translocation between chrV L 36,848 and chrX L 81,482 | Copy number  Discordant read pairs (534 read pairs)  Junction sequence (87 reads) |
| 9 | PGSP3614 | *De novo* telomere addition chrV L 39,718_39,719 | Copy number  Discordant read pairs (156 read pairs)  Junction sequence (12 reads) |
| 10 | PGSP3615 | Hairpin-mediated inversion chrV L 34,339-107_34,339-75 (in *can1::hisG*) | Copy number  Discordant read pairs (65 read pairs)  Junction sequence (141 reads) |
|  |  | Homology-mediated translocation between chrV L *ura3-52* and chrV L *yel068c::URA3* | Copy number |
| 11 | PGSP3616 | Microhomology-mediated translocation between chrV L 32,538 (in *CAN1* insertion) and chrXIV R 763,417 | Copy number  Discordant read pairs (652 read pairs)  Junction sequence (47 reads) |
| 12 | PGSP3617 | Hairpin-mediated inversion chrV L 34,339-107_34,339-75 (in *can1::hisG*) | Copy number  Discordant read pairs (38 read pairs)  Junction sequence (98 reads) |
|  |  | Homology-mediated translocation between chrV L *ura3-52* and chrV L *yel068c::URA3* | Copy number |

**AF.** GCR structures from *exo1Δ* *yku80Δ* uGCR strains

| ***No.*** | ***Sample**** | ***GCR description*** | ***GCR evidence***** |
| --- | --- | --- | --- |
| 1 | PGSP5060 | *De novo* telomere addition chrV L 43,023_43,026 | Copy number  Discordant read pairs (29 read pairs)  Junction sequence (7 reads) |
| 2 | PGSP5061 | *De novo* telomere addition chrV L 34,849_34,852 | Copy number  Discordant read pairs (52 read pairs)  Junction sequence (4 reads) |
| 3 | PGSP5062 | *De novo* telomere addition chrV L 40,186_40,190 | Copy number  Discordant read pairs (40 read pairs)  Junction sequence (7 reads) |
| 4 | PGSP5063 | Hairpin-mediated inversion chrV L 34,339-953_34,339-883 (in *can1::hisG*) | Copy number  Discordant read pairs (111 read pairs)  Junction sequence |
|  |  | Homology-mediated translocation between chrV L *ura3-52* and the unannotated chrV R “*YERWdelta27”* (chrV:449,322..449,631; Nene et al. 2018) | Copy number  Discordant read pairs (94 read pairs) |
| 5 | PGSP5064 | *De novo* telomere addition chrV L 42,160_42,165 | Copy number  Discordant read pairs (68 read pairs)  Junction sequence (6 reads) |
| 6 | PGSP5065 | Interstitial deletion between chrV L 16,194 and chrV L 34,575 | Copy number  Discordant read pairs (243 read pairs)  Junction sequence (24 reads) |
| 7 | PGSP5066 | Hairpin-mediated inversion chrV L 34,339-107_34,339-75 (in *can1::hisG*) | Copy number  Discordant read pairs (39 read pairs)  Junction sequence (78 reads) |
|  |  | Homology-mediated translocation between chrV L *YELWdelta1* and the unannotated chrV R “*YERWdelta27”* (chrV:449,322..449,631; Nene et al. 2018) | Copy number  Discordant read pairs (122 read pairs) |
| 8 | PGSP5067 | Hairpin-mediated inversion chrV L 34,339-502_34,339-459 (in *can1::hisG*) | Copy number  Discordant read pairs (68 read pairs)  Junction sequence |
|  |  | Homology-mediated translocation between chrV L *ura3-52* and chrV L *yel068c::URA3* | Copy number |
| 9 | PGSP5068 | *De novo* telomere addition chrV L 34,831_34,832 | Copy number  Discordant read pairs (25 read pairs)  Junction sequence (4 reads) |
| 10 | PGSP5069 | Hairpin-mediated inversion chrV L 34,339-107_34,339-75 (in *can1::hisG*) | Copy number  Discordant read pairs (33 read pairs)  Junction sequence (75 reads) |
|  |  | Homology-mediated translocation between chrV L *ura3-52* and the unannotated chrV R “*YERWdelta27”* (chrV:449,322..449,631; Nene et al. 2018) | Copy number  Discordant read pairs (86 read pairs) |
| 11 | PGSP5070 | Hairpin-mediated inversion chrV L 35,664_35,725 | Copy number  Discordant read pairs (207 read pairs)  Junction sequence (30 reads) |
|  |  | *De novo* telomere addition chrV L 75,107_75,110 | Copy number  Discordant read pairs (34 read pairs)  Junction sequence (13 reads) |
| 12 | PGSP5071 | *De novo* telomere addition chrV L 34,834_34,847 | Copy number  Discordant read pairs (41 read pairs)  Junction sequence (7 reads) |

**AG.** GCR structures from *pif1Δ* *yku80Δ* uGCR strains

| ***No.*** | ***Sample**** | ***GCR description*** | ***GCR evidence***** |
| --- | --- | --- | --- |
| 1 | PGSP4932 | *De novo* telomere addition chrV L 34,339-58_34,339-55 | Copy number  Discordant read pairs (89 read pairs)  Junction sequence (7 reads) |
| 2 | PGSP4933 | *De novo* telomere addition chrV L 34,339-649_34,339-647 | Copy number  Discordant read pairs (60 read pairs)  Junction sequence (9 reads) |
| 3 | PGSP4934 | *De novo* telomere addition chrV L 41,041_41,046 | Copy number  Discordant read pairs (55 read pairs)  Junction sequence |
| 4 | PGSP4935 | *De novo* telomere addition chrV L 26,831_26,839 | Copy number  Discordant read pairs (69 read pairs)  Junction sequence (9 reads) |
| 5 | PGSP4936 | *De novo* telomere addition chrV L 40,758_40,762 | Copy number  Discordant read pairs (80 read pairs)  Junction sequence (5 reads) |
| 6 | PGSP4937 | *De novo* telomere addition chrV L 38,575_38,578 | Copy number  Discordant read pairs (76 read pairs)  Junction sequence (4 reads) |
| 7 | PGSP4938 | *De novo* telomere addition chrV L 42,138_42,141 | Copy number  Discordant read pairs (69 read pairs)  Junction sequence (4 reads) |
| 8 | PGSP4939 | *De novo* telomere addition chrV L 35,916_35,919 | Copy number  Discordant read pairs (83 read pairs)  Junction sequence (5 reads) |
| 9 | PGSP4940 | *De novo* telomere addition chrV L 34,339-58_34,339-55 | Copy number  Discordant read pairs (122 read pairs)  Junction sequence (16 reads) |
| 10 | PGSP4941 | *De novo* telomere addition chrV L 30,543_30,546 | Copy number  Discordant read pairs (46 read pairs)  Junction sequence (5 reads) |
| 11 | PGSP4942 | *De novo* telomere addition chrV L 35,913_35,919 | Copy number  Discordant read pairs (103 read pairs)  Junction sequence (10 reads) |
|  |  | Unrelated to GCR: chrXIV disomy | Copy number |
| 12 | PGSP4943 | *De novo* telomere addition chrV L 34,339-78_34,339-78 | Copy number  Discordant read pairs (163 read pairs)  Junction sequence (10 reads) |

**AH.** GCR structures from *sae2Δ yku80Δ* uGCR strains

| ***No.*** | ***Sample**** | ***GCR description*** | ***GCR evidence***** |
| --- | --- | --- | --- |
| 1 | PGSP4677 | Hairpin-mediated inversion chrV L 34,339-107_34,339-75 (in *can1::hisG*) | Copy number  Discordant read pairs (54 read pairs)  Junction sequence (161 reads) |
|  |  | Homology-mediated translocation between chrV L *ura3-52* and chrV R *YERWdelta22* | Copy number |
| 2 | PGSP4678 | Hairpin-mediated inversion chrV L 34,339-107_34,339-75 (in *can1::hisG*) | Copy number  Discordant read pairs (62 read pairs)  Junction sequence (152 reads) |
|  |  | Homology-mediated translocation between chrV L *ura3-52* and chrXVI R *YPRWTy1-3* | Copy number |
| 3 | PGSP4679 | Hairpin-mediated inversion chrV L 34,339-107_34,339-75 (in *can1::hisG*) | Copy number  Discordant read pairs (64 read pairs)  Junction sequence (125 reads) |
|  |  | Homology-mediated translocation between chrV L *ura3-52* and chrXIV L *YNLCTy1-1* | Copy number |
| 4 | PGSP4680 | Hairpin-mediated inversion chrV L 34,339-107_34,339-75 (in *can1::hisG*) | Copy number  Discordant read pairs (48 read pairs)  Junction sequence (122 reads) |
|  |  | Homology-mediated translocation between chrV L *ura3-52* and chrIII R *YCRWdelta11* | Copy number |
| 5 | PGSP4682 | Hairpin-mediated inversion chrV L 34,339-107_34,339-75 (in *can1::hisG*) | Copy number  Discordant read pairs (88 read pairs)  Junction sequence (188 reads) |
|  |  | Homology-mediated translocation between chrV L *ura3-52* and chrIII R *YCRWdelta8/9/10* | Copy number |
| 6 | PGSP4684 | Hairpin-mediated inversion chrV L 34,339-107_34,339-75 (in *can1::hisG*) | Copy number  Discordant read pairs (81 read pairs)  Junction sequence (185 reads) |
|  |  | Homology-mediated translocation between chrV L *ura3-52* and the unannotated chrV R “*YERWdelta27”* (chrV:449,322..449,631; Nene et al. 2018) | Copy number |
| 7 | PGSP4685 | Hairpin-mediated inversion chrV L 34,339-107_34,339-75 (in *can1::hisG*) | Copy number  Discordant read pairs (93 read pairs)  Junction sequence (236 reads) |
|  |  | Homology-mediated translocation between chrV L *ura3-52* and chrV R *YERCTy1-1* | Copy number |
|  |  | Homology-mediated inversion between chrV R *YERCdelta16* and the unannotated chrV R “*YERWdelta27”* (chrV:449,322..449,631; Nene et al. 2018) | Copy number  Discordant read pairs (299 read pairs) |
| 8 | PGSP4686 | Hairpin-mediated inversion chrV L 34,339-107_34,339-75 (in *can1::hisG*) | Copy number  Discordant read pairs (62 read pairs)  Junction sequence (166 reads) |
|  |  | Homology-mediated translocation between chrV L *ura3-52* and chrIII R *YCRWdelta8/9/10* | Copy number |
| 9 | PGSP4687 | Hairpin-mediated inversion chrV L 34,339-107_34,339-75 (in *can1::hisG*) | Copy number  Discordant read pairs (88 read pairs)  Junction sequence (200 reads) |
|  |  | Homology-mediated translocation between chrV L *ura3-52* and chrIII R *YCRWdelta8/9/10* | Copy number |
| 10 | PGSP4688 | Hairpin-mediated inversion chrV L 34,339-107_34,339-75 (in *can1::hisG*) | Copy number  Discordant read pairs (76 read pairs)  Junction sequence (170 reads) |
|  |  | Homology-mediated translocation between chrV L *ura3-52* and chrIV L *YDRWTy1-4* | Copy number |
| 11 | PGSP4689 | *De novo* telomere addition chrV L 27,851_27,852 | Copy number  Discordant read pairs (215 read pairs)  Junction sequence (11 reads) |
| 12 | PGSP4690 | Hairpin-mediated inversion chrV L 34,339-107_34,339-75 (in *can1::hisG*) | Copy number  Discordant read pairs (90 read pairs)  Junction sequence (198 reads) |
|  |  | Homology-mediated translocation between chrV L *ura3-52* and chrIII R *YCRWdelta11* | Copy number |

**AI.** GCR structures from *sae2Δ pif1Δ yku80Δ* uGCR strains

| ***No.*** | ***Sample**** | ***GCR description*** | ***GCR evidence***** |
| --- | --- | --- | --- |
| 1 | PGSP4717 | *De novo* telomere addition chrV L 34,339-752_34,339-751 (in *can1::hisG*) | Copy number  Discordant read pairs (145 read pairs)  Junction sequence (13 reads) |
| 2 | PGSP4718 | Hairpin-mediated inversion chrV L 38,072_38,086 | Copy number  Discordant read pairs (123 read pairs)  Junction sequence (27 reads) |
|  |  | *De novo* telomere addition chrV L 116,832_116,837 | Copy number  Discordant read pairs (65 read pairs)  Junction sequence (27 reads) |
|  |  | Unrelated to GCR: Increased copy number of chrII by ~1.5-fold | Copy number |
| 3 | PGSP4719 | Hairpin-mediated inversion chrV L 34,339-107_34,339-75 (in *can1::hisG*) | Copy number  Discordant read pairs (109 read pairs)  Junction sequence (166 reads) |
|  |  | *De novo* telomere addition chrV L in *ura3-52* | Copy number |
| 4 | PGSP4720 | Hairpin-mediated inversion chrV L 34,339-107_34,339-75 (in *can1::hisG*) | Copy number  Discordant read pairs (133 read pairs)  Junction sequence (228 reads) |
|  |  | *De novo* telomere addition chrV L 86,027_86,030 | Copy number  Discordant read pairs (115 read pairs)  Junction sequence (228 reads) |
| 5 | PGSP4721 | *De novo* telomere addition chrV L 34,888_34,898 | Copy number  Discordant read pairs (97 read pairs)  Junction sequence (11 reads) |
|  |  | Unrelated to GCR: Increased copy number of chrII by ~1.2-fold | Copy number |
| 6 | PGSP4722 | *De novo* telomere addition chrV L 38,291_38,294 | Copy number  Discordant read pairs (67 read pairs)  Junction sequence (9 reads) |
|  |  | Unrelated to GCR: Increased copy number of chrII by ~1.2-fold | Copy number |
| 7 | PGSP4723 | *De novo* telomere addition chrV L 34,892_34,898 | Copy number  Discordant read pairs (70 read pairs)  Junction sequence (9 reads) |
| 8 | PGSP4724 | *De novo* telomere addition chrV L 34,339-1125_34,339-1126 (in *can1::hisG*) | Copy number  Discordant read pairs (129 read pairs)  Junction sequence (9 reads) |
| 9 | PGSP4725 | *De novo* telomere addition chrV L 40,971_40,976 | Copy number  Discordant read pairs (67 read pairs)  Junction sequence (9 reads) |
|  |  | Unrelated to GCR: Increased copy number of chrII by ~1.5-fold | Copy number |
| 10 | PGSP4726 | Hairpin-mediated inversion chrV L 34,339-107_34,339-75 (in *can1::hisG*) | Copy number  Discordant read pairs (121 read pairs)  Junction sequence (196 reads) |
|  |  | *De novo* telomere addition chrV L 83,962_83,965 | Copy number  Discordant read pairs (96 read pairs)  Junction sequence (196 reads) |
| 11 | PGSP4727 | *De novo* telomere addition chrV L 28,644_28,647 | Copy number  Discordant read pairs (240 read pairs)  Junction sequence (24 reads) |
| 12 | PGSP4728 | Hairpin-mediated inversion chrV L 34,339-107_34,339-75 (in *can1::hisG*) | Copy number  Discordant read pairs (72 read pairs)  Junction sequence (153 reads) |
|  |  | Homology-mediated translocation between chrV L *YELCdelta4* and chrXVI L *YPLWdelta8* | Copy number  Discordant read pairs (117 read pairs)  Junction sequence (24 reads) |

**AJ.** GCR structures from *sgs1Δ* uGCR strains

| ***No.*** | ***Sample**** | ***GCR description*** | ***GCR evidence***** |
| --- | --- | --- | --- |
| 1 | PGSP937 | *De novo* telomere addition chrV L 34,847_34,847 | Copy number  Discordant read pairs (59 read pairs)  Junction sequence (11 reads)  *hph^-^* |
| 2 | PGSP939 | Non-homology interstitial deletion between chrV L 23,935 and 40,785 | Copy number  Discordant read pairs (230 read pairs)  Junction sequence (40 reads)  *hph^+^* |
| 3 | PGSP940 | Microhomology-mediated translocation between chrV L 37,092 and chrXI R 326,520 | Copy number  Discordant read pairs (199 read pairs)  Junction sequence (18 reads)  *hph^-^* |
| 4 | PGSP941 | *De novo* telomere addition chrV L 34,831_34,832 | Copy number  Discordant read pairs (77 read pairs)  Junction sequence (12 reads)  *hph^-^* |
| 5 | PGSP942 | *De novo* telomere addition chrV L 40,314_40,317 | Copy number  Discordant read pairs (43 read pairs)  Junction sequence (11 reads)  *hph^-^* |
| 6 | PGSP943 | *De novo* telomere addition chrV L 34,839_34,847 | Copy number  Discordant read pairs (94 read pairs)  Junction sequence (24 reads)  *hph^-^* |
| 7 | PGSP944 | Hairpin-mediated inversion chrV L 35,694_35,777 | Copy number  Discordant read pairs (208 read pairs)  Junction sequence (64 reads) |
|  |  | Homology-mediated translocation between chrV L *ura3-52* and chrV L *yel068c::URA3* | Copy number  *hph^+^* |
| 8 | PGSP945 | Microhomology-mediated translocation between chrV L 25,817-529 (in *CAN1* insertion) and chrXII 170,721 | Copy number  Discordant read pairs (326 read pairs)  Junction sequence (9 reads)  *hph^-^* |
| 9 | PGSP946 | *De novo* telomere addition chrV L 43,023_43,026 | Copy number  Discordant read pairs (71 read pairs)  Junction sequence (24 reads)  *hph^-^* |
| 10 | PGSP948 | *De novo* telomere addition chrV L 34,850_34,852 | Copy number  Discordant read pairs (71 read pairs)  Junction sequence (18 reads)  *hph^-^* |
| 11 | PGSP949 | *De novo* telomere addition chrV L 34,842_34,847 | Copy number  Discordant read pairs (40 read pairs)  Junction sequence (14 reads)  *hph^-^* |
|  |  | Unrelated to GCR: chrI disomy | Copy number |

**AK.** GCR structures from *exo1Δ* *sgs1Δ* uGCR strains

| ***No.*** | ***Sample**** | ***GCR description*** | ***GCR evidence***** |
| --- | --- | --- | --- |
| 1 | PGSP4350 | *De novo* telomere addition chrV L 34,831_34,832 | Copy number  Discordant read pairs (62 read pairs)  Junction sequence (17 reads)  *hph^-^* |
| 2 | PGSP4351 | *De novo* telomere addition chrV L 40,596_40,598 | Copy number  Discordant read pairs (105 read pairs)  Junction sequence (18 reads)  *hph^-^* |
| 3 | PGSP4352 | *De novo* telomere addition chrV L 34,842_34,847 | Copy number  Discordant read pairs (78 read pairs)  Junction sequence (5 reads)  *hph^-^* |
| 4 | PGSP4353 | *De novo* telomere addition chrV L 25,817-996_25,817-992 (in inserted *CAN1*) | Copy number  Discordant read pairs (54 read pairs)  Junction sequence (14 reads)  *hph^-^* |
| 5 | PGSP4354 | *De novo* telomere addition chrV L 40,460_40,462 | Copy number  Discordant read pairs (58 read pairs)  Junction sequence (8 reads)  *hph^-^* |
| 6 | PGSP4356 | *De novo* telomere addition chrV L 34,849_34,852 | Copy number  Discordant read pairs (60 read pairs)  Junction sequence (17 reads)  *hph^-^* |
| 7 | PGSP4357 | *De novo* telomere addition chrV L 34,833 | Copy number  Discordant read pairs (72 read pairs)  Junction sequence (20 reads)  *hph^-^* |
| 8 | PGSP4358 | *De novo* telomere addition chrV L 34,867_34,870 | Copy number  Discordant read pairs (50 read pairs)  Junction sequence (19 reads)  *hph^-^* |
| 9 | PGSP4359 | *De novo* telomere addition chrV L 34,834_34,838 | Copy number  Discordant read pairs (55 read pairs)  Junction sequence (13 reads)  *hph^-^* |
|  |  | Unrelated to GCR: Duplication of a region of chrVII bounded by *YGRWTy3-1* and *YGRWsigma6* | Copy number |
| 10 | PGSP4360 | *De novo* telomere addition chrV L 34,834_34,838 | Copy number  Discordant read pairs (76 read pairs)  Junction sequence (13 reads)  *hph^-^* |
| 11 | PGSP4361 | *De novo* telomere addition chrV L 41,652_41,654 | Copy number  Discordant read pairs (117 read pairs)  Junction sequence (20 reads)  *hph^-^* |
| 12 | PGSP4362 | *De novo* telomere addition chrV L 34,438 | Copy number  Discordant read pairs (79 read pairs)  Junction sequence (20 reads)  *hph^-^* |

**AL.** GCR structures from *sae2Δ* *exo1Δ* *yku80Δ* uGCR strains

| ***No.*** | ***Sample**** | ***GCR description*** | ***GCR evidence***** |
| --- | --- | --- | --- |
| 1 | PGSP5046 | Hairpin-mediated inversion chrV L 34,339-107_34,339-75 (in *can1::hisG*) | Copy number  Discordant read pairs (122 read pairs)  Junction sequence (116 reads) |
|  |  | Homology-mediated translocation between chrV L *ura3-52* and chrV L *yel068c::URA3* | Copy number |
| 2 | PGSP5047 | Hairpin-mediated inversion chrV L 34,339-107_34,339-75 (in *can1::hisG*) | Copy number  Discordant read pairs (43 read pairs)  Junction sequence (105 reads) |
|  |  | Homology-mediated translocation between chrV L *ura3-52* and chrV L *yel068c::URA3* | Copy number |
|  |  | Homology-mediated translocation between chrV L *HXT13* and chrXIV R *HXT17* | Copy number  Discordant read pairs (92 read pairs) |
| 3 | PGSP5048 | Hairpin-mediated inversion chrV L 34,339-107_34,339-75 (in *can1::hisG*) | Copy number  Discordant read pairs (47 read pairs)  Junction sequence (92 reads) |
|  |  | Homology-mediated translocation between chrV L *ura3-52* and chrV R “*YERWdelta27*” (chrV:449,322..449,631; Nene et al. 2018) | Copy number  Discordant read pairs (213 read pairs indicating an adjacent Ty-related sequence) |
| 4 | PGSP5049 | Hairpin-mediated inversion chrV L 40,877_40,894 | Copy number  Discordant read pairs (61 read pairs)  Junction sequence (23 reads) |
|  |  | Homology-mediated translocation between chrV L *ura3-52* and chrXVI R *YPRWTy1-3* | Copy number |
| 5 | PGSP5050 | Hairpin-mediated inversion chrV L 34,339-107_34,339-75 (in *can1::hisG*) | Copy number  Discordant read pairs (85 read pairs)  Junction sequence (122 reads) |
|  |  | Homology-mediated translocation between chrV L *ura3-52* and chrV L *yel068c::URA3* | Copy number |
| 6 | PGSP5051 | Hairpin-mediated inversion chrV L 34,339-107_34,339-75 (in *can1::hisG*) | Copy number  Discordant read pairs (35 read pairs)  Junction sequence (23 reads) |
|  |  | Homology-mediated translocation between chrV L *ura3-52* and chrV L *yel068c::URA3* | Copy number |
| 7 | PGSP5052 | Hairpin-mediated inversion chrV L 34,339-107_34,339-75 (in *can1::hisG*) | Copy number  Discordant read pairs (80 read pairs)  Junction sequence (140 reads) |
|  |  | Homology-mediated translocation between chrV L *ura3-52* and chrV L *yel068c::URA3* | Copy number |
| 8 | PGSP5053 | Hairpin-mediated inversion chrV L 34,339-107_34,339-75 (in *can1::hisG*) | Copy number  Discordant read pairs (67 read pairs)  Junction sequence (124 reads) |
|  |  | Homology-mediated translocation between chrV L *ura3-52* and chrV L *yel068c::URA3* | Copy number |
| 9 | PGSP5054 | Hairpin-mediated inversion chrV L 27,348_27,361 | Copy number  Discordant read pairs (172 read pairs)  Junction sequence (37 reads) |
|  |  | Homology-mediated translocation between chrV L *ura3-52* and chrV R *YERWdelta22* | Copy number  Discordant read pairs (250 read pairs indicating a Ty-related sequence adjacent to *YERWdelta22*) |
|  |  | Homology-mediated translocation between chrV R *YERCTy1-2* and chrVIII R *YHRCTy1-1* | Copy number |
| 10 | PGSP5055 | Hairpin-mediated inversion chrV L 34,339-107_34,339-75 (in *can1::hisG*) | Copy number  Discordant read pairs (96 read pairs)  Junction sequence (37 reads) |
|  |  | Homology-mediated translocation between chrV L *ura3-52* and chrV L *yel068c::URA3* | Copy number |
| 11 | PGSP5058 | Hairpin-mediated inversion chrV L 39,661_39,681 | Copy number  Discordant read pairs (128 read pairs)  Junction sequence (37 reads) |
|  |  | Homology-mediated translocation between chrV L *YELCdelta4* and chrXVI R *YPRCTy1-4* | Copy number  Discordant read pairs (250 read pairs indicating a Ty-related sequence adjacent to *YELCdelta4*) |
| 12 | PGSP5059 | Hairpin-mediated inversion chrV L 35,837_35,872 | Copy number  Discordant read pairs (161 read pairs)  Junction sequence (35 reads) |
|  |  | Homology-mediated translocation between chrV L *ura3-52* and chrV L *yel068c::URA3* | Copy number |

**AM.** GCR structures from *sgs1Δ yku80Δ* uGCR strains

| ***No.*** | ***Sample**** | ***GCR description*** | ***GCR evidence***** |
| --- | --- | --- | --- |
| 1 | PGSP4691 | Hairpin-mediated inversion chrV L 40,968_40,983 | Copy number  Discordant read pairs (100 read pairs)  Junction sequence (29 reads) |
|  |  | Homology-mediated translocation between chrV L *ura3-52* and the unannotated chrV R “*YERWdelta27”* (chrV:449,322..449,631; Nene et al. 2018) | Copy number  Discordant read pairs (175 read pairs)  Junction sequence (63 reads) |
| 2 | PGSP4692 | Hairpin-mediated inversion chrV L 35,685_35,968 | Copy number  Discordant read pairs (69 read pairs)  Junction sequence (xx reads) |
|  |  | Homology-mediated translocation between chrV L *ura3-52* and chrIV R *YDRWTy2-3* | Copy number |
| 3 | PGSP4693 | *De novo* telomere addition chrV L 34,849_34,852 | Copy number  Discordant read pairs (82 read pairs)  Junction sequence (20 reads) |
| 4 | PGSP4694 | Translocation between chrV L 42,094 and chrV R telomere | Copy number  Junction sequence |
|  |  | Homology-mediated inversion between chrV R *YERCdelta14* and chrV R “*YERWdelta27*” (chrV:449,322..449,631; Nene et al. 2018) | Copy number  Discordant read pairs (233 read pairs) |
| 5 | PGSP4695 | *De novo* telomere addition chrV L 25,817-642 (in inserted *CAN1*) | Copy number  Discordant read pairs (110 read pairs)  Junction sequence (2 reads) |
| 6 | PGSP4696 | Translocation of chrV L 41,669 to an unknown Ty element | Junction sequence |
|  |  | Homology-mediated translocation between unknown Ty element and *YELWdelta1* | Copy number |
|  |  | Homology-mediated translocation between *ura3-52* and *YELCdelta4* | Copy number |
|  |  | Translocation of chrV L 41,669 to an unknown Ty element | Junction sequence |
|  |  | Homology-mediated translocation between unknown Ty element and *YELWdelta1* | Copy number |
|  |  | Homology-mediated translocation between *ura3-52* and *YERCTy1-1* | Copy number  Discordant read pairs (180 read pairs) |
| 7 | PGSP4697 | *De novo* telomere addition chrV L 39,968_39,969 | Copy number  Discordant read pairs (152 read pairs)  Junction sequence (20 reads) |
| 8 | PGSP4698 | Hairpin-mediated inversion chrV L 34,339-1,219_34,339-501 (in *can1::hisG* insertion) | Copy number  Discordant read pairs (250 read pairs)  Junction sequence (46 reads) |
|  |  | Homology-mediated translocation between chrV L *ura3-52* and chrV L *yel068c::URA3* | Copy number |
| 9 | PGSP4699 | Hairpin-mediated inversion chrV L 35,581_35,596 | Copy number  Discordant read pairs (87 read pairs)  Junction sequence (28 reads) |
|  |  | Homology-mediated translocation between chrV L *YELWdelta1* and chrVII R *YGRWTy2-2* | Copy number |
| 10 | PGSP4700 | Hairpin-mediated inversion chrV L 42,277_42,290 | Copy number  Discordant read pairs (229 read pairs)  Junction sequence (39 reads) |
|  |  | Homology-mediated inversion between chrV L *ura3-52* and chrV L *YELCdelta4* | Copy number |
|  |  | Hairpin-mediated inversion chrV L 42,277_42,290 | Copy number  Discordant read pairs (229 read pairs)  Junction sequence (39 reads) |
|  |  | Homology-mediated inversion between chrV L *ura3-52* and chrIV R *YDRWTy1-4* | Copy number |
| 11 | PGSP4701 | *De novo* telomere addition chrV L 25,817-2,198_25,817-2,195 (in inserted *CAN1*) | Copy number  Discordant read pairs (199 read pairs)  Junction sequence (5 reads) |
| 12 | PGSP4702 | *De novo* telomere addition chrV L 34,842_34,847 | Copy number  Discordant read pairs (159 read pairs)  Junction sequence (20 reads) |

**AN.** GCR structures from *sae2Δ sgs1Δ yku80Δ* uGCR strains

| ***No.*** | ***Sample**** | ***GCR description*** | ***GCR evidence***** |
| --- | --- | --- | --- |
| 1 | PGSP4739 | Hairpin-mediated inversion chrV L 34,339-107_34,339-75 (in *can1::hisG*) | Copy number  Discordant read pairs (102 read pairs)  Junction sequence (137 reads) |
|  |  | Homology-mediated inversion chrV L *YELWdelta6* to *YELWdelta1* | Copy number  Discordant read pairs (289 read pairs) |
|  |  | Homology-mediated translocation chrV L *PAU2* to chrIX L *PAU14* or chrX L *PAU1*. | Junction sequence |
| 2 | PGSP4740 | Hairpin-mediated inversion chrV L 34,339-107_34,339-75 (in *can1::hisG*) | Copy number  Discordant read pairs (110 read pairs)  Junction sequence (135 reads) |
|  |  | Homology-mediated translocation between chrV L *ura3-52* and chrV L *yel068c::URA3* | Copy number |
| 3 | PGSP4742 | Hairpin-mediated inversion chrV L 25,817-568_25,817-546 | Copy number  Discordant read pairs (156 read pairs)  Junction sequence (4 reads) |
|  |  | Homology-mediated translocation between chrV L *ura3-52* and chrXV L *YOLCdelta1* | Copy number |
| 4 | PGSP4744 | Hairpin-mediated inversion chrV L 41,350_41,363 | Copy number  Discordant read pairs (100 read pairs)  Junction sequence (49 reads) |
|  |  | Homology-mediated translocation between chrV L *ura3-52* and chrVII L *YGLCdelta1* | Copy number |
| 5 | PGSP4918 | Two-step hairpin-mediated inversion chrV L 42,109_42,130 | Copy number  Discordant read pairs (78 read pairs)  Junction sequence (17 reads) |
|  |  | Homology-mediated translocation between chrV L *YELCdelta4* and chrIV R *YDRCdelta22* (in *YDRCTy1-3*) | Copy number  Discordant read pairs (51 read pairs) |
| 6 | PGSP4919 | Hairpin-mediated inversion chrV L 42,939_42,955 | Copy number  Discordant read pairs (97 read pairs)  Junction sequence (17 reads) |
|  |  | Homology-mediated translocation between chrV L *ura3-52* and chrX L *YJLCdelta3* | Copy number |
| 7 | PGSP4920 | Hairpin-mediated inversion chrV L 34,339-107_34,339-75 (in *can1::hisG*) | Copy number  Discordant read pairs (63 read pairs)  Junction sequence (47 reads) |
|  |  | Homology-mediated translocation between chrV L *YELWdelta6* and the unannotated chrXII R *“YLRWTy1-4”*; in this strain background there is a full-length Ty element, “*YLRWTy1-4*” telomeric to *YLRCdelta21* and in the opposite orientation (Liang et al. 2018) | Copy number |
| 8 | PGSP4921 | Hairpin-mediated inversion chrV L 40,968_40,983 | Copy number  Discordant read pairs (95 read pairs)  Junction sequence (17 reads) |
|  |  | Homology-mediated translocation between chrV L *YELCdelta4* and chrXVI L *YPLWdelta8* | Copy number  Discordant read pairs (86 read pairs) |
| 9 | PGSP4922 | Hairpin-mediated inversion chrV L 25,817-1,530_25,817-1,506 | Copy number  Discordant read pairs (205 read pairs)  Junction sequence (23 reads) |
|  |  | Homology-mediated translocation between chrV L *PAU2* and chrI L *PAU8* | Copy number  Discordant read pairs (105 read pairs) |
| 10 | PGSP4924 | Translocation chrV 34,343 to chrXV R 651,717 | Copy number  Discordant read pairs (362 read pairs)  Junction sequence (23 reads) |
|  |  | Hairpin-mediated inversion chrXV R at *ade2::hisG* (equivalent hairpin to chrV L 34,339-107_34,339-75 at *can1::hisG*). Translocation and inversion generate a triplication of chrXV R from 566,713 to 651,717. And inversion generates a duplication of chrV L from 34,343 to *ura3-52*. | Copy number  Discordant read pairs (123 read pairs)  Junction sequence (87 reads) |
|  |  | Homology-mediated translocation between chrV L *ura3-52* and chrXIV L *YNLCTy1-1* | Copy number |

**AO.** GCR structures from *rad52Δ* uGCR strains

| ***No.*** | ***Sample**** | ***GCR description*** | ***GCR evidence***** |
| --- | --- | --- | --- |
| 1 | PGSP329 | *De novo* telomere addition chrV L 34,834_34,847 | Copy number  Discordant read pairs (38 read pairs)  Junction sequence (8 reads) |
|  |  | Unrelated to GCR: a second copy of the chrVIII fragment between 340,154 and 455,909 | Copy number  Discordant read pairs (71 read pairs)  Junction sequence (9 reads) |
| 2 | PGSP330 | *De novo* telomere addition chrV L 41,812_41,815 | Copy number  Discordant read pairs (50 read pairs)  Junction sequence (7 reads) |
| 3 | PGSP331 | *De novo* telomere addition chrV L 34,864_34,865 | Copy number  Discordant read pairs (48 read pairs)  Junction sequence (7 reads) |
| 4 | PGSP332 | *De novo* telomere addition chrV L 28,540 | Copy number  Discordant read pairs (36 read pairs)  Junction sequence (7 reads) |
| 5 | PGSP333 | *De novo* telomere addition chrV L 25,817-587_25,817-586 | Copy number  Discordant read pairs (58 read pairs)  Junction sequence (6 reads) |
| 6 | PGSP334 | *De novo* telomere addition chrV L 40,707_40,709 | Copy number  Discordant read pairs (47 read pairs)  Junction sequence (6 reads) |
| 7 | PGSP335 | *De novo* telomere addition chrV L 34,842_34847 | Copy number  Discordant read pairs (62 read pairs)  Junction sequence (7 reads) |
| 8 | PGSP336 | *De novo* telomere addition chrV L 41,078_41,086 | Copy number  Discordant read pairs (51 read pairs)  Junction sequence (10 reads) |
| 9 | PGSP337 | *De novo* telomere addition chrV L 38,391_38,396 | Copy number  Discordant read pairs (64 read pairs)  Junction sequence (10 reads) |
| 10 | PGSP338 | Microhomology-mediate translocation between chrV L 42,062 and chrIX L 335,548 | Copy number  Discordant read pairs (113 read pairs)  Junction sequence (10 reads) |
| 11 | PGSP339 | *De novo* telomere addition chrV L 38,715_38,716 | Copy number  Discordant read pairs (85 read pairs)  Junction sequence (10 reads) |
| 12 | PGSP340 | *De novo* telomere addition chrV L 35,140_35,142 | Copy number  Discordant read pairs (61 read pairs)  Junction sequence (15 reads) |

**AP.** GCR structures from *sae2Δ* *rad52Δ* uGCR strains

| ***No.*** | ***Sample**** | ***GCR description*** | ***GCR evidence***** |
| --- | --- | --- | --- |
| 1 | PGSP4877 | Hairpin-mediated inversion chrV L 34,339-107_34,339-75 (in *can1::hisG*) | Copy number  Discordant read pairs (86 read pairs)  Junction sequence (153 read pairs) |
|  |  | Microhomology-mediated translocation between chrV 100,611 and chrXII R rDNA 460,003 most likely mediated by microhomology between mononucleotide T repeats | Copy number  Discordant read pairs (218 read pairs) |
| 2 | PGSP4878 | Hairpin-mediated inversion chrV L 27,569_27,583 | Copy number  Discordant read pairs (83 read pairs)  Junction sequence (25 reads) |
|  |  | Microhomology-mediated translocation between chrV 151556 and chrV R 482740 | Copy number  Discordant read pairs (237 read pairs)  Junction sequence (22 read pairs) |
| 3 | PGSP4881 | Microhomology-mediated translocation chrV L 40,241 and chrII R 232,118 | Copy number  Discordant read pairs (294 read pairs)  Junction sequence (27 reads) |
| 4 | PGSP4882 | Hairpin-mediated inversion chrV L 34,339-107_34,339-75 (in *can1::hisG*) | Copy number  Discordant read pairs (96 read pairs)  Junction sequence (163 reads) |
|  |  | *De novo* telomere addition chrV L 68,851_68,856 | Copy number  Discordant read pairs (103 read pairs)  Junction sequence (15 reads) |
| 5 | PGSP4883 | Hairpin-mediated inversion chrV L 34,339-107_34,339-75 (in *can1::hisG*) | Copy number  Discordant read pairs (110 read pairs)  Junction sequence (171 reads) |
|  |  | Microhomology-mediated translocation chrV L 60,298 and chrXVI L 155,535 | Copy number  Discordant read pairs (381 read pairs)  Junction sequence (43 reads) |
| 6 | PGSP4884 | Hairpin-mediated inverted duplication chrV L 29,418_29,435 | Copy number  Discordant read pairs (269 read pairs)  Junction sequence (56 reads) |
|  |  | Hairpin-mediated inverted duplication chrV L 51,934_51,962 | Copy number  Discordant read pairs (131 read pairs)  Junction sequence (32 reads) |
|  |  | Hairpin-mediated inverted duplication chrV L 29,418_29,435 | Copy number  Discordant read pairs (269 read pairs)  Junction sequence (56 reads) |
|  |  | Microhomology-mediated translocation chrV L 76,938 and chrV R 436,682 | Copy number  Discordant read pairs (288 read pairs)  Junction sequence (29 reads) |
| 7 | PGSP4885 | *De novo* telomere addition chrV L 26,384_26,388 | Copy number  Discordant read pairs (120 read pairs)  Junction sequence (10 reads) |
| 8 | PGSP4886 | Microhomology-mediated translocation between chrV L 32,099 (in inserted *CAN1* sequence) and chrVII R 1,003,287 | Copy number  Discordant read pairs (279 read pairs)  Junction sequence (36 reads) |
| 9 | PGSP4887 | Hairpin-mediated inversion chrV L 42,939_42,955 | Copy number  Discordant read pairs (98 read pairs)  Junction sequence (32 reads) |
|  |  | Homology-mediated translocation between the tRNA genes chr V L *IMT4* and chrXVI R *IMT2* | Copy number  Discordant read pairs (318 read pairs)  Junction sequence |
| 10 | PGSP4888 | *De novo* telomere addition chrV L 34,842_34,847 | Copy number  Discordant read pairs (81 read pairs)  Junction sequence (4 reads) |
| 11 | PGSP4889 | Hairpin-mediated inversion chrV L 34,339-107_34,339-75 (in *can1::hisG*) | Copy number  Discordant read pairs (112 read pairs)  Junction sequence (32 reads) |
|  |  | *De novo* telomere addition chrV L 68,851_68,854 | Copy number  Discordant read pairs (127 read pairs)  Junction sequence (13 reads) |
| 12 | PGSP4890 | Hairpin-mediated inversion chrV L 34,339-107_34,339-75 (in *can1::hisG*) | Copy number  Discordant read pairs (95 read pairs)  Junction sequence (184 reads) |
|  |  | Microhomology-mediated translocation between chrV L 127,669 and chrIII L 1,517 | Copy number  Discordant read pairs (330 read pairs)  Junction sequence (33 reads) |

*Sample name in square brackets corresponds to library name.

**For GCRs that retain the telomeric end of chromosome V typically by resolving an inverted duplication by a *ura3-52/URA3* homology-mediated recombination event, retention of the *hph* marker (*hph^+^*) provides additional evidence for the rearrangement. For GCRs that lose the telomeric end of chromosome V, loss of the *hph* marker (*hph^-^*) provides additional evidence for the rearrangement.
