# Supplementary material for: Mechanisms underlying genome instability mediated by formation of foldback inversions in Saccharomyces cerevisiae
Source: eLife. 2020 Aug 7;9:e58223. doi: 10.7554/eLife.58223 (PMC7467729; doi:10.7554/eLife.58223)
Supplement: Supplementary file 4. [file elife-58223-supp4.docx]

**Supplementary File 4. *De novo* telomere addition junction sequences**

**chrV L 25,817-2,793_25,817-2,790 (in *CAN1* insertion)** – PGSP464 [bzg124] (*rad10*)

CACCACACCCACACCCACACACCACACCCA:CACA:AATTAGCAGAAAGAAGAGTGGTTGCGAACAGAGTAAACC

|||| |||||||||||||||||||||||||||||||||||||||

:CACA:AATTAGCAGAAAGAAGAGTGGTTGCGAACAGAGTAAACC

:chrV 33708

**chrV L 25,817-2,220_25,817-2,214 (in *CAN1* insertion)** – PGSP4907 (*slx1*)

CACACCACACACCCACACACCACAC:CCACACC:TCTGACCAACGCCGGCCCAGTGGGCGCT

||||||| ||||||||||||||||||||||||||||

:CCACACC:TCTGACCAACGCCGGCCCAGTGGGCGCT

:chrV 33135

**chrV L 25,817-2,208_25,817-2,204 (in inserted *CAN1*)** – PGSP231 (*mus81*)

ACACCACACCCACACCACACCCACACCCACAC:CCAAC:GCCGGCCCAGTGGGCGCTCTTATATCA

||||| |||||||||||||||||||||||||||

:CCAAC:GCCGGCCCAGTGGGCGCTCTTATATCA

:chrV 33123

**chrV L 25,817-2,198_25,817-2,195 (in inserted *CAN1*)** – PGSP4701 (*sgs1 yku80*)

ACCACACCCACACCACACCCACACCACACCCACA:CCCA:GTGGGCGCTCTTATATCATATTTATTTATGGGTT

|||| ||||||||||||||||||||||||||||||||||

:CCCA:GTGGGCGCTCTTATATCATATTTATTTATGGGTT

:chrV 33113

**chrV L 25817-1749_25817-1748 (in inserted *CAN1*)** – PGSP5096 (*sae2 exo1* chrV:25,817-1,749 gRNA)

CACACCACACCACACCCACACACAC:CC:TGGGGTCCAGGTATAATATCTAAGGATAAAAAC

|| |||||||||||||||||||||||||||||||||

:CC:TGGGGTCCAGGTATAATATCTAAGGATAAAAAC

:chrV 32664

**chrV L 25,817-1,321_25,817-1,320 (in inserted *CAN1*)** – PGSP4613 (*sae2 pol32*)

ACACCCACACCACACCCACACCACACCCACACA:CC:TAAATTCCTGTCAAGGACCACCAAAGGTG

|| |||||||||||||||||||||||||||||

:CC:TAAATTCCTGTCAAGGACCACCAAAGGTG

:chrV 32236

**chrV L 25,817-1,263_25,817-1,263** **(in inserted *CAN1*)** – PGSP4566 [bzg118] (*pol32*)

CCCACACACCACACCCACACACCCACAC:C:TGCTGCATTTGGCGCTTTGGCTTACATGGAGACATCTACTGG

| ||||||||||||||||||||||||||||||||||||||||||

:C:TGCTGCATTTGGCGCTTTGGCTTACATGGAGACATCTACTGG

:chrV 32178

**chrV L 25,817-1,103_25,817-1,103 (in inserted *CAN1*)** – PGSP4569 [bzg104] (*rrm3*)

CCCACACACCCACACCCACA:C:TCTCGTGACGAGTTACCATTTAAAGCTAAATTAATGCCCGGCTTGGC

| |||||||||||||||||||||||||||||||||||||||||||||||

:C:TCTCGTGACGAGTTACCATTTAAAGCTAAATTAATGCCCGGCTTGGC

:chrV 32018

**chrV L 25,817-996_25,817-992 (in inserted *CAN1*)** – PGSP4353 (*sgs1*)

ACCCACACCCACACACCCACACCCA:CACCA:AAATTCAATGGTGTTAGCTTTGCTGCCGCCTATATCT

||||| |||||||||||||||||||||||||||||||||||||

:CACCA:AAATTCAATGGTGTTAGCTTTGCTGCCGCCTATATCT

:chrV 31911

**chrV L 25,817-642 (in inserted *CAN1*)** – PGSP4695 (*sgs1 yku80*)

ACCACACCCACACAC::TCACCACAACAATCTTGCTCCCGAAAAGAAAGCAGA

||||||||||||||||||||||||||||||||||||

::TCACCACAACAATCTTGCTCCCGAAAAGAAAGCAGA

:chrV 31557

**chrV L 25,817-639_25,817-635 (in *CAN1* insertion)** - PGSP783 [bzg095] (*exo1*)

ACACCCACACCCACACCCACAC:CCACA:ACAATCTTGCTCCCGAAAAGAAAGCAGACGGAGTAGAAGC

||||| ||||||||||||||||||||||||||||||||||||||||

:CCACA:ACAATCTTGCTCCCGAAAAGAAAGCAGACGGAGTAGAAGC

:chrV 31554

**chrV L 25,817-636_25,817-635 (in *CAN1* insertion)** – PGSP5078 (*sae2 mus81* chrV:25,817-1,749 gRNA)

CACCACACC:CA:ACAATCTTGCTCCCGAAAAGAAAGCAGACGGAGT

|| ||||||||||||||||||||||||||||||||||

:CA:ACAATCTTGCTCCCGAAAAGAAAGCAGACGGAGT

:chrV 31551

**chrV L 25,817-587_25,817-586 (in *CAN1* insertion)** – PGSP333 (*rad52*)

CCACACCACACCCACACCACACCAC:AC:TCCTTCAGACCTTCAAGTATATATATATATATA

|| |||||||||||||||||||||||||||||||||

:AC:TCCTTCAGACCTTCAAGTATATATATATATATA

:chrV 31502

**chrV L 25,817-491_25,817-490 (in *CAN1* insertion)** – PGSP4894 (*yen1*)

CCACACCCACACACACCACACCCAC:AC:TCATTTTTCGTTATCTTCACGTCACCCGAACCT

|| |||||||||||||||||||||||||||||||||

:AC:TCATTTTTCGTTATCTTCACGTCACCCGAACCT

:chrV 31406

**chrV L 26,329_26,330** – PGSP462 [bzg122] (*rad10*)

ACCCACACCCACCACACCCACAC:AC:TGTATATCTGTGCATGGTTGACTAATTGTTGTTTGATGGCCTGAAGTTC

|| |||||||||||||||||||||||||||||||||||||||||||||||||

:AC:TGTATATCTGTGCATGGTTGACTAATTGTTGTTTGATGGCCTGAAGTTC

:chrV 26329

**chrV L 26,384_26,388** – PGSP756 [bzg113] (*pol32*)

ACACCACACCCACACCACACCCACACCCA:CACAC:TCTGCAAATTATCCCGACATTCAGACGTACACG

||||| |||||||||||||||||||||||||||||||||

:CACAC:TCTGCAAATTATCCCGACATTCAGACGTACACG

:chrV 26384

**chrV L 26,384_26,388** – PGSP4705 (*sae2 pif1*)

CCCACACACACCACACCCACACACCACACCCA:CACAC:TCTGCAAATTATCCCGACATTCAGACGTAC

||||| ||||||||||||||||||||||||||||||

:CACAC:TCTGCAAATTATCCCGACATTCAGACGTAC

:chrV 26384

**chrV L 26,384_26,388** – PGSP227 (*mus81*)

CACACCACACCCACACACACCACACCCACACC:CACAC:TCTGCAAATTATCCCGACATTCAGACGTAC

||||| ||||||||||||||||||||||||||||||

:CACAC:TCTGCAAATTATCCCGACATTCAGACGTAC

:chrV 26384

**chrV L 26,384_26,388** – PGSP4885 (*sae2 rad52*)

CCACACCCACACCCACACCCACACC:CACAC:TCTGCAAATTATCCCGACATTCAGACGTAC

||||| ||||||||||||||||||||||||||||||

:CACAC:TCTGCAAATTATCCCGACATTCAGACGTAC

:chrV 26384

**chrV L 26,401_26,403** – PGSP4579 [bzg131] (*rad10*)

ACACCCACACCCACACCCACACCCACA:CCC:GACATTCAGACGTACACGTACAAAACACGAATGCTAC

||| |||||||||||||||||||||||||||||||||||||

:CCC:GACATTCAGACGTACACGTACAAAACACGAATGCTAC

:chrV 26401

**chrV L 26,401_26,403** – PGSP4912 (*slx1*)

CACACCCACACACACCACACCCACA:CCC:GACATTCAGACGTACACGTACAAAACACGAAT

||| ||||||||||||||||||||||||||||||||

:CCC:GACATTCAGACGTACACGTACAAAACACGAAT

:chrV 26401

**chrV L 26,581_26,583** - PGSP781 [bzg093] (*exo1*)

CCCACACACCCACACCCACACACCAC:ACC:TTCGCGAACAGTATACTGGAGAGTGTTGCGACAAGATAT

||| |||||||||||||||||||||||||||||||||||||||

:ACC:TTCGCGAACAGTATACTGGAGAGTGTTGCGACAAGATAT

:chrV 26581

**chrV L 26,678_26,681** – PGSP4597 [bzg147] (*sae2 rrm3*)

CACACCCACACACCCACACACCCACA:CCCA:GACCCATCTCCACCTTTTGCAAGCACGAACCACGTAG

|||| ||||||||||||||||||||||||||||| ||||||

:CCCA:GACCCATCTCCACCTTTTGCAAGCACGAAGAACGTAG

:chrV 26678

**chrV L 26831_26839** – PGSP4935 (*pif1 yku80*)

CCACACCCACACACCACACCCACAC:CCAAAAAAC:GGTTATGCCTCCGGAAGTAGCCACCA

||||||||| ||||||||||||||||||||||||||

:CCAAAAAAC:GGTTATGCCTCCGGAAGTAGCCACCA

:chrV 26831

**chrV L 27,423_27,427** – PGSP4703 (*sae2 pif1*)

CACCACACCCACACCCACACCCACAC:ACCCC:GAAGTTCATCGTTTACAATGTGCCGTAAAGAACAC

||||| |||||||||||||||||||||||||||||||||||

:ACCCC:GAAGTTCATCGTTTACAATGTGCCGTAAAGAACAC

:chrV 27423

**chrV L 27,423_27,427** – PGSP4910 (*slx1*)

CACCCACACCCACACCCACACCCAC:ACCCC:GAAGTTCATCGTTTACAATGTGCCGTAAAG

||||| ||||||||||||||||||||||||||||||

:ACCCC:GAAGTTCATCGTTTACAATGTGCCGTAAAG

:chrV 27423

**chrV L 27,759_27,761** – PGSP4572 [bzg107] (*rrm3*)

CACACCACACCCACACCCACACCACAC:CCA:ACGCTAACCACATCTTCTCCAGCACCTTCTTACATTG

||| |||||||||||||||||||||||||||||||||||||

:CCA:ACGCTAACCACATCTTCTCCAGCACCTTCTTACATTG

:chrV 27759

**chrV L 27,932_27,934** – PGSP 4898 (*yen1*)

CACACCCACACACCCACACCACACC:CAC:TTTCCAGGCGTACGCGACTTCGTCGTACTCCC

||| ||||||||||||||||||||||||||||||||

:CAC:TTTCCAGGCGTACGCGACTTCGTCGTACTCCC

:chrV 27932

**chrV L 28540** – PGSP332 (*rad52*)

TGTGGGTGTGTGGGGGTGGTGTG::TCGGGTGTGTATTGGTTCCATTCACCTTAGCGGGT

|||||||||||||||||||||||||||||||||||

::TCGGGTGTGTATTGGTTCCATTCACCTTAGCGGGT

:chrV 28540

**chrV L 27,851_27,852** – PGSP4689 (*sae2 yku80*)

ACCCACACCACACCACACCCACACACCACAC:CC:GCCCGATCTACCGTGTCGCATTATTCTTCTCAC

|| |||||||||||||||||||||||||||||||||

:CC:GCCCGATCTACCGTGTCGCATTATTCTTCTCAC

:chrV 27851

**chrV L 28,643_28,647** – PGSP4577 [bzg129] (*rad10*)

CACCCACACCCACACCCACACACAC:ACCAC:TGTACATGCTATGGGAAATAAAGTACTCGAGAC

||||| |||||||||||||||||||||||||||||||||

:ACCAC:TGTACATGCTATGGGAAATAAAGTACTCGAGAC

:chrV 28643

**chrV L 28,644_28,647** – PGSP4727 (*sae2 yku80 pif1*)

CACACCCACACACCCACAC:CCAC:TGTACATGCTATGGGAAATAAAGTACTCGAGACATCCATTGACAC

|||| |||||||||||||||||||||||||||||||||||||||||||||

:CCAC:TGTACATGCTATGGGAAATAAAGTACTCGAGACATCCATTGACAC

:chrV 28644

**chrV L 28,683_28,685** – PGSP222 (*mus81*)

ACCACACCCACACCACACCCACACCACAC:CCA:TTGACACCTTGGGACTTGATTCAAGATAGAGGTATC

||| ||||||||||||||||||||||||||||||||||||

:CCA:TTGACACCTTGGGACTTGATTCAAGATAGAGGTATC

:chrV 28683

**chrV L 28,840_28,841** – PGSP226 (*mus81*)

CACCACACCCACACCACACCCACACCCACAC:CC:TCCTTGTATTCATTTGTTTCTGTTATCGTTGGTA

|| ||||||||||||||||||||||||||||||||||

:CC:TCCTTGTATTCATTTGTTTCTGTTATCGTTGGTA

:chrV 28840

**chrV L 29,259_29,261** - PGSP777 [bzg089] (*exo1*)

ACCCACACACACCACACCCACACCC:ACA:TTCATTACAACTTATACCTGGGGAACACCAGAAAGAATAG

||| ||||||||||||||||||||||||||||||||||||||||

:ACA:TTCATTACAACTTATACCTGGGGAACACCAGAAAGAATAG

:chrV 29259

**chrV L 29,511_29,513** – PGSP4715 (*sae2 pif1*)

ACACCCACACACCACACCCACACCACACC:CAC:TTTACAAGCAGTAAAGATAGGAAAGATTAATATTATC

||| |||||||||||||||||||||||||||||||||||||

:CAC:TTTACAAGCAGTAAAGATAGGAAAGATTAATATTATC

:chrV 29511

**chrV L 30,170_30,176** – PGSP4916 (*slx1*)

ACCCACACACCACACCCACACACCC:ACAAAAA:TTTCCATGGGGAATGTGGTTAGCATGTT

||||||| ||||||||||||||||||||||||||||

:ACAAAAA:TTTCCATGGGGAATGTGGTTAGCATGTT

:chrV 30170

**chrV L 30543_30546** – PGSP4941 (*pif1 yku80*)

CCCACACCACACCCACACACCACAC:CCAC:TGTCAGTACAATTATAATCATACTTATAACG

|||| |||||||||||||||||||||||||||||||

:CCAC:TGTCAGTACAATTATAATCATACTTATAACG

:chrV 30543

**chrV L 30,742_30,745** – PGSP4710 (*sae2 pif1*)

AACCCACACACCCACACACCACACCCACA:CCCA:ATAATTATGCAATAACCAATACATCCACCAAATG

|||| ||||||||||||||||||||||||||||||||||

:CCCA:ATAATTATGCAATAACCAATACATCCACCAAATG

:chrV 30742

**chrV L 30,844_30,847** – PGSP4842 (wt chrV:30843-gRNA)

ACACCCACACCACACCCACACCCAC:ACCC:TGGTATGTTCTCTTGCCAGCAAGAGTCAAGT

|||| |||||||||||||||||||||||||||||||

:ACCC:TGGTATGTTCTCTTGCCAGCAAGAGTCAAGT

:chrV 30844

**chrV L 30,844_30,847** – PGSP4843 (wt chrV:30843-gRNA)

ACCCACACCCACACACCCACACCAC:ACCC:TGGTATGTTCTCTTGCCAGCAAGAGTCAAGT

|||| |||||||||||||||||||||||||||||||

:ACCC:TGGTATGTTCTCTTGCCAGCAAGAGTCAAGT

:chrV 30844

**chrV L 30,844_30,847** – PGSP4844 (wt chrV:30843-gRNA)

CACACCACACCCACACCACACCCAC:ACCC:TGGTATGTTCTCTTGCCAGCAAGAGTCAAGT

|||| |||||||||||||||||||||||||||||||

:ACCC:TGGTATGTTCTCTTGCCAGCAAGAGTCAAGT

:chrV 30844

**chrV L 30,844_30,847** – PGSP4845 (wt chrV:30843-gRNA)

CCCACACACCACACCACACCCACAA:ACCC:TGGTATCTTCTCTTGCCAGCCAGAGTCAAGT

|||| |||||||||||||||||||||||||||||||

:ACCC:TGGTATGTTCTCTTGCCAGCAAGAGTCAAGT

:chrV 30844

**chrV L 30,844_30,847** – PGSP4846 (wt chrV:30843-gRNA)

CACACACCACACCCACACACACCAC:ACCC:TGGTATGTTCTCTTGCCAGCAAGAGTCAAGT

|||| |||||||||||||||||||||||||||||||

:ACCC:TGGTATGTTCTCTTGCCAGCAAGAGTCAAGT

:chrV 30844

**chrV L 30,844_30,847** – PGSP4847 (wt chrV:30843-gRNA)

CCACACCCACACACCACACCCACAC:ACCC:TGGTATGTTCTCTTGCCAGCAAGAGTCAAGT

|||| |||||||||||||||||||||||||||||||

:ACCC:TGGTATGTTCTCTTGCCAGCAAGAGTCAAGT

:chrV 30844

**chrV L 30,844_30,847** – PGSP5029 (wt hs-del chrV:30843-gRNA)

ACACACCCACCC:TGGTATGTTCTCTTGCCAGCAAGAGTCAAGT

|||| |||||||||||||||||||||||||||||||

:ACCC:TGGTATGTTCTCTTGCCAGCAAGAGTCAAGT

:chrV 30844

**chrV L 30,844_30,847** – PGSP5030 (wt hs-del chrV:30843-gRNA)

CACACCCACACCACACCACACCCAC:ACCC:TGGTATGTTCTCTTGCCAGCAAGAGTCAAGT

|||| |||||||||||||||||||||||||||||||

:ACCC:TGGTATGTTCTCTTGCCAGCAAGAGTCAAGT

:chrV 30844

**chrV L 30,844_30,847** – PGSP5032 (wt hs-del chrV:30843-gRNA)

CACACCCACACCACACCCACACCAC:ACCC:TGGTATGTTCTCTTGCCAGCAAGAGTCAAGT

|||| |||||||||||||||||||||||||||||||

:ACCC:TGGTATGTTCTCTTGCCAGCAAGAGTCAAGT

:chrV 30844

**chrV L 30,844_30,847** – PGSP5033 (wt hs-del chrV:30843-gRNA)

CCCACACCCACACCCACACACCCAC:ACCC:TGGTATGTTCTCTTGCCAGCAAGAGTCAAGT

|||| |||||||||||||||||||||||||||||||

:ACCC:TGGTATGTTCTCTTGCCAGCAAGAGTCAAGT

:chrV 30844

**chrV L 30,890_30,890** – PGSP5028 (wt hs-del chrV:30843-gRNA)

AGCAAGAGACAAGTTAATGACAATC:A:GTCTTAACGTCCAATCAACTATAAATCCGAGGGC

| ||||||||||||||||||||||||||||||||||

:A:GTCTTAACGTCCAATCAACTATAAATCCGAGGGC

:chrV 30890

**chrV L 34,339-1,125_34,339-1,126 (in inserted hisG sequence)**- PGSP4725 (*sae2 yku80 pif1*)

CCCACACACACCACACCACAC:CC:TACAGAACCCAAAATATCAACGCATTACGTAGGCCTGATAAG

|| ||||||||||||||||||||||||||||||||||||||||||

:CC:TACAGAACCCAAAATATCAACGCATTACGTAGGCCTGATAAG

:chrV 34339-1125

**chrV L 34,339-752_34,339-751 (in inserted hisG sequence)**- PGSP4717 (*sae2 pif1 yku80*)

ACCACACCCACACACCACACCCACACACCCACA:CC:TGAATCACGCCCTGAATACGGGTCAGCAATT

|| |||||||||||||||||||||||||||||||

:CC:TGAATCACGCCCTGAATACGGGTCAGCAATT

**chrV L 34339-649_34339-647 (in inserted hisG sequence)** – PGSP4933 (*pif1 yku80*)

CACCCACACCCACACACCCACACAC:CCA:GCCCCGCGCGCGGCGCGACTTCGACAGAACCA

||| ||||||||||||||||||||||||||||||||

:CCA:GCCCCGCGCGCGGCGCGACTTCGACAGAACCA

:chrV 34339-649

**chrV L 34,339-645_34,339-643 (in inserted hisG sequence)**- PGSP4709 (*sae2 pif1*)

ACACACACCCACACCACACCCACACACCCACA:CCC:CGCGCGCGGCGCGACTTCGACAGAACCATTTAACAGACACG

||| |||||||||||||||||||||||||||||||||||||||||

:CCC:CGCGCGCGGCGCGACTTCGACAGAACCATTTAACAGACACG

:chrV 34339-645

**chrV L 34,339-645_34,339-643 (in inserted hisG sequence)**- PGSP4711 (*sae2 pif1*)

CACACCCACACACCACACCACACACCACAC:CCC:GCGCGCGGCGCGACTTCGACAGAACCATTTAACAGACACGATTT

||| |||||||||||||||||||||||||||||||||||||||||

:CCC:CGCGCGCGGCGCGACTTCGACAGAACCATTTAACAGACACG

:chrV 34339-645

**chrV L 34,339-497 (in inserted hisG sequence)**- PGSP775 [bzg097] (*exo1*)

CCACACACCCACACACCCACACACC::TCAACCGGTGTTGCCAGCGATAAACGGCAGCCGCCGAAGTCAA

|||||||||||||||||||||||||||||||||||||||||||

::TCAACCGGTGTTGCCAGCGATAAACGGCAGCCGCCGAAGTCAA

::chrV 34339-497

**chrV L 34,339-494_34,339-492 (in inserted hisG sequence)**- PGSP4599 [bzg149] (*sae2 rrm3*)

CCCACACCACACCACACCCACACCCAC:ACC:GGTGTTGCCAGCGATAAACGGCAGCCGCCGAAGTC

||| |||||||||||||||||||||||||||||||||||

:ACC:GGTGTTGCCAGCGATAAACGGCAGCCGCCGAAGTC

:chrV 34339-494

**chrV L 34,339-344_34,339-341 (in inserted hisG sequence)**- PGSP773 [bzg085] (*exo1*)

ACCCACACCCCACACCCACACACACCCAC:ACCA:GACCCGGAATGTCATCATCACGCACGCGCAGG

|||| ||||||||||||||||||||||||||||||||

:ACCA:GACCCGGAATGTCATCATCACGCACGCGCAGG

:chrV 34339-344

**chrV L 34,339-78_34,339-78 (in inserted hisG sequence)**- PGSP757 [bzg114] (*pol32*)

CACCCACACACCCACACACACCACACCCACACA:C:TTTCTCATGCGTTCATGCACCACTGGAAGATC

| ||||||||||||||||||||||||||||||||

:C:TTTCTCATGCGTTCATGCACCACTGGAAGATC

:chrV 34339-78

**chrV L 34,339-78_34,339-78 (in inserted hisG sequence)**- PGSP4564 [bzg116] (*pol32*)

CCCACACACACACCACACCCACAC:C:TTTCTCATGCGTTCATGCACCACTGGAAGATC

| ||||||||||||||||||||||||||||||||

:C:TTTCTCATGCGTTCATGCACCACTGGAAGATC

:chrV 34339-78

**chrV L 34,339-78_34,339-78 (in inserted hisG sequence)**- PGSP4567 [bzg119] (*pol32*)

ACCACCCACACCCACACCCACACCCCC:C:TTTCTCATGCGTTCATGCACCACTGGAAGATCTGAATT

| ||||||||||||||||||||||||||||||||||||||

:C:TTTCTCATGCGTTCATGCACCACTGGAAGATCTGAATT

:chrV 34339-78

**chrV L 34,339-78_34,339-78 (in inserted hisG sequence)** – PGSP4943 (*pif1 yku80*)

CACCCACACACCACACCCACACACA:C:TTTCTCATGCGTTCATGCACCACTGGAAGATC

| ||||||||||||||||||||||||||||||||

:C:TTTCTCATGCGTTCATGCACCACTGGAAGATC

:chrV 34339-78

**chrV L 34,339-59_34,339-55 (in inserted hisG sequence)**- PGSP4707 (*sae2 pif1*)

CACACCCACACACCACACCCACAC:ACCAC:TGGAAGATCTGAATTCTTGAAGACGAAAGGGCCTCGTGA

||||| |||||||||||||||||||||||||||||||||||||||

:ACCAC:TGGAAGATCTGAATTCTTGAAGACGAAAGGGCCTCGTGA

:chrV 34,339-59

**chrV L 34,339-58_34,339-55 (in inserted hisG sequence)**- PGSP4941 (*pif1 yku80*)

CAGACACCACACCCACACACCACACCCACACCCACACCACAC:CCAC:TGGAAGATCTGAATTCTTGAAG

|||| ||||||||||||||||||||||

:CCAC:TGGAAGATCTGAATTCTTGAAG

:34,339-58

**chrV L 34,339-58_34,339-55 (in inserted hisG sequence)** - PGSP4932 (*pif1 yku80*)

CACCCACACACCACACCCACACACCACACCCACAC:CCAC:TGGAAGATCTGAATTCTTGAAGACGAAAG

|||| |||||||||||||||||||||||||||||

:CCAC:TGGAAGATCTGAATTCTTGAAGACGAAAG

:chrV 34,339-58

**chrV L 34,339-58_34,339-55 (in inserted hisG sequence)** – PGSP4901 (*yen1*)

ACACACCACACCCACACACCACACCCACACCACAC:CCAC:TGGAAGATCTGAATTCTTGAAGACGAAAG

|||| |||||||||||||||||||||||||||||

:CCAC:TGGAAGATCTGAATTCTTGAAGACGAAAG

:chrV 34,339-58

**chrV L 34,397_34,398** - PGSP778 [bzg090] (*exo1*)

CCACACCCACACCCACACAC:CC:AACGGCAAATGCTAAGCTATTTCCAAGGGTTTGTGCCCATACAC

|| ||||||||||||||||||||||||||||||||||||||||||||

:CC:AACGGCAAATGCTAAGCTATTTCCAAGGGTTTGTGCCCATACAC

:chrV 34397

**chrV L 34,404_34,405** – PGSP4675 (*sae2* *exo1*)

CACACCCACACACCCACACA:CA:AATGCTAAGCTATTTCCAAGGGTTTGTGCCCATACACACGATAT

|| ||||||||||||||||||||||||||||||||||||||||||||

:CA:AATGCTAAGCTATTTCCAAGGGTTTGTGCCCATACACACAATAT

:chrV 34404

**chrV L 34,438** – PGSP4362 (*exo1 sgs1*)

ACACACCCACACACAC::TACACACAATATTTTACTCCGTGTTCCATCCTA

|||||||||||||||||||||||||||||||||

::TACACACAATATTTTACTCCGTGTTCCATCCTA

:chrV 34438

**chrV L 34,441_34,445** – PGSP4666 (*sae2* *exo1*)

CCACACACACCACACACACCCACACACCACACCCACACCCAC:ACACA:ATATTTTACTCCGTGTTCCATC

||||| ||||||||||||||||||||||

:ACACA:ATATTTTACTCCGTGTTCCATC

:chrV 34441

**chrV L 34,713_34,715** – PGSP4840 (wild-type chrV:34470-gRNA)

CACCCACACCCCACACACCCACACA:CCC:TACGAACCAGCTATTACTAGGCTGGGCAAAAT

||| ||||||||||||||||||||||||||||||||

:CCC:TACGAACCAGCTATTACTAGGCTGGGCAAAAT

:chrV 34713

**chrV L 34,831_34,832** – PGSP755 [bzg117] (*pol32*)

ACACACCACACCCACACACACCACACCC:AC:TCCACAACAACACCCTCCACTGCAGGTCCTTCATCCACGCC

|| |||||||||||||||||||||||||||||||||||||||||

:AC:TCCACAACAACACCCTCCACTGCAGGTCCTTCATCCACGCC

:chrV 34831

**chrV L 34,831_34,832** – PGSP463 [bzg123] (*rad10*)

CACACCCACACCCACACACAC:AC:TCCACAACAACACCCTCCACTGCAGGTCCTTCATCCACGCCCACTCCTAGTAGT

|| ||||||||||||||||||||||||||||||||||||||||||||||||||||||

:AC:TCCACAACAACACCCTCCACTGCAGGTCCTTCATCCACGCCAAATCCTAGTAGT

:chrV 34831

**chrV L 34,831_34,832** – PGSP941 (*sgs1*)

ACCACACCCACACCACACCCACAC:AC:TCCACAACAACACCCTCCACTGCAGGTCCTTCATCCACGCCAAA

|| ||||||||||||||||||||||||||||||||||||||||||||

:AC:TCCACAACAACACCCTCCACTGCAGGTCCTTCATCCACGCCAAA

:chrV 34831

**chrV L 34,831_34,832** – PGSP4350 (*exo1 sgs1*)

CACCACACCCACACAC:AC:TCCACAACAACACCCTCCACTGCAGGTCCTTCATCCACG

|| |||||||||||||||||||||||||||||||||||||||

:AC:TCCACAACAACACCCTCCACTGCAGGTCCTTCATCCACG

:chrV 34831

**chrV L 34,831_34,832** – PGSP4839 (wild-type chrV:34470-gRNA)

ACCCAACCACACCCACACCACACCC:AC:TCCACAACAACACCCTCCACTGCAGGTCCTTCA

|| |||||||||||||||||||||||||||||||||

:AC:TCCACAACAACACCCTCCACTGCAGGTCCTTCA

:chrV 34831

**chrV L 34,831_34,832** - PGSP5068 (*exo1 yku80*)

CCACACACACACACCACACCCACAC:AC:TCCACAACAACACCCTCCACTGCAGGTCCTTCA

|| |||||||||||||||||||||||||||||||||

:AC:TCCACAACAACACCCTCCACTGCAGGTCCTTCA

:chrV 34831

**chrV L 34,833** – PGSP4357 (*exo1 sgs1*)

CCACACCCACACCCACACACCCCACACCCACA::TCCACAACAACACCCTCCACTGCAGGTCCTTCATC

|||||||||||||||||||||||||||||||||||

::TCCACAACAACACCCTCCACTGCAGGTCCTTCATC

::chrV 34833

**chrV L 34,833** – PGSP4838 (wild-type chrV:34470-gRNA)

CACCACACCCACACCACACACCACA::TCCACAACAACACCCTCCACTGCAGGTCCTTCATC

|||||||||||||||||||||||||||||||||||

::TCCACAACAACACCCTCCACTGCAGGTCCTTCATC

:chrV 34833

**chrV L 34,834_34,838** – PGSP755 [bzg112] (*pol32*)

CCACACCCACAC:CCACA:ACAACACCCTCCACTGCAGGTCCTTCATCCACGCCAAATCCTAGTAGT

||||| ||||||||||||||||||||||||||||||||||||||||||||||||

:CCACA:ACAACACCCTCCACTGCAGGTCCTTCATCCACGCCAAATCCTAGTAGT

:chrV 34834

**chrV L 34,834_34,838** – PGSP461 [bzg121] (*rad10*)

CCCACACCCACACACCCACACCACAC:CCACA:ACAACACCCTCCACTGCAGGTCCTTCATCCACGCCA

||||| ||||||||||||||||||||||||||||||||||||

:CCACA:ACAACACCCTCCACTGCAGGTCCTTCATCCACGCCA

:chrV 34834

**chrV L 34,834_34,838** – PGSP4359 (*exo1 sgs1*)

ACCACACCCACCCACACCCACACCCACAC:CCACA:ACAACACCCTCCACTGCAGGTCCTTCATCCACGCCAAA

||||| ||||||||||||||||||||||||||||||||||||||

:CCACA:ACAACACCCTCCACTGCAGGTCCTTCATCCACGCCAAA

:chrV 34834

**chrV L 34,834_34,838** – PGSP4360 (*exo1 sgs1*)

CCCACACACACACCCACAC:CCACA:ACAACACCCTCCACTGCAGGTCCTTCATCCAC

||||| ||||||||||||||||||||||||||||||||

:CCACA:ACAACACCCTCCACTGCAGGTCCTTCATCCAC

:chrV 34834

**chrV L 34,834_34,838** – PGSP4660 (*sae2 mus81*)

ACACACACACCACACCCACACACCACACCCACACACCACACCCACAC:CCACA:ACAACACCCTCCACTGCAGGTCC

||||| |||||||||||||||||||||||

:CCACA:ACAACACCCTCCACTGCAGGTCC

:chrV 34834

**chrV L 34834_34847** – PGSP5071 (*exo1 yku80*)

ACACCCACACACCCACACACCACAC:CCACAACAACACCC:TCCACTGCAGGTCCTTCATCC

|||||||||||||| |||||||||||||||||||||

:CCACAACAACACCC:TCCACTGCAGGTCCTTCATCC

:chrV 34834

**chrV L 34,834_34,847** – PGSP329 (*rad52*)

CACCCACACCACACCCACACCACAC:CCACAACAACACCC:TCCACTGCAGGTCCTTCATCC

|||||||||||||| |||||||||||||||||||||

:CCACAACAACACCC:TCCACTGCAGGTCCTTCATCC

:chrV 34834

**chrV L 34,834_34,847** – PGSP4904 (*slx1*)

CCCACAC:CCACAACAACACCC:TCCACTGCAGGTCCTTCATCC

|||||||||||||| |||||||||||||||||||||

:CCACAACAACACCC:TCCACTGCAGGTCCTTCATCC

:chrV 34834

**chrV L 34,839_34,847** – PGSP943 (*sgs1*)

ACCACACCCACACACCACACCCACAC:ACCACACCC:TCCACTGCAGGTCCTTCATCCACGCCAAATCCTAGTAGTAAC

||||||||| ||||||||||||||||||||||||||||||||||||||||||

:ACAACACCC:TCCACTGCAGGTCCTTCATCCACGCCAAATCCTAGTAGTAAC

:chrV 34839

**chrV L 34,839_34,847** – PGSP4895 (*yen1*)

CACACCCACACCCACACCCACACCC:ACAACACCC:TCCACTGCAGGTCCTTCATCCACGCC

||||||||| ||||||||||||||||||||||||||

:ACAACACCC:TCCACTGCAGGTCCTTCATCCACGCC

:chrV 34839

**chrV L 34,842_34,847** – PGSP758 [bzg115] (*pol32*)

ACACCCACACCCACACACCC:ACACCC:TCCACTGCAGGTCCTTCATCCACGCCAAATCCTAGTAGTA

|||||| ||||||||||||||||||||||||||||||||||||||||

:ACACCC:TCCACTGCAGGTCCTTCATCCACGCCAAATCCTAGTAGTA

:chrV 34842

**chrV L 34,842_34,847** – PGSP949 (*sgs1*)

CACACACCACACCACACCCACACCCACACCC:ACACCC:TCCACTGCAGGTCCTTCATCCACGCCA

|||||| |||||||||||||||||||||||||||

:ACACCC:TCCACTGCAGGTCCTTCATCCACGCCA

:chrV 34842

**chrV L 34,842_34,847** – PGSP4573 [bzg108] (*rrm3*)

ACACCCACACCCACACCCAC:ACACCC:TCCACTGCAGGTCCTTCATCCACGCCAAATCCTAGCAGTA

|||||| ||||||||||||||||||||||||||||||||||||||||

:ACACCC:TCCACTGCAGGTCCTTCATCCACGCCAAATCCTAGTAGTA

:chrV 34842

**chrV L 34,842_34,847** – PGSP4702 (*sgs1 yku80*)

ACACCCACACACACCCACACAC:ACACCC:TCCACTGCAGGTCCTTCATCCACGCCAAAT

|||||| ||||||||||||||||||||||||||||||

:ACACCC:TCCACTGCAGGTCCTTCATCCACGCCAAAT

:chrV 34842

**chrV L 34,842_34,847** – PGSP4649 (*mus81*)

ACACCCACACACCACACCCACACCCAC:ACACCC:TCCACTGCAGGTCCTTCATCCACGCCAAATCC

|||||| ||||||||||||||||||||||||||||||||

:ACACCC:TCCACTGCAGGTCCTTCATCCACGCCAAATCC

:chrV 34842

**chrV L 34,842_34,847** – PGSP4352 (*exo1 sgs1*)

CACACACCACACCCACACCCACCACACCCACACACC:ACACCC:TCCACTGCAGGTCCTTCATCCACG

|||||| ||||||||||||||||||||||||

:ACACCC:TCCACTGCAGGTCCTTCATCCACG

:chrV 34842

**chrV L 34,842_34,847** – PGSP4826 (sae2 chrV:34470-gRNA)

ACACCCACACACCACACCCACACCC:ACACCC:TCCACTGCAGGTCCTTCATCCACGCCAAA

|||||| |||||||||||||||||||||||||||||

:ACACCC:TCCACTGCAGGTCCTTCATCCACGCCAAA

:chrV 34842

**chrV L 34,842_34,847 –** PGSP335 (*rad52*)

ACACCCACACCCACACCCACACACC:ACACCC:TCCACTGCAGGTCCTTCATCCACGCCAAA

|||||| |||||||||||||||||||||||||||||

:ACACCC:TCCACTGCAGGTCCTTCATCCACGCCAAA

:chrV 34842

**chrV L 34,842_34,847** – PGSP4888 (*sae2 rad52*)

CCACACCCACACCCACACCACACCC:ACACCC:TCCACTGCAGGTCCTTCATCCACGCCAAA

|||||| |||||||||||||||||||||||||||||

:ACACCC:TCCACTGCAGGTCCTTCATCCACGCCAAA

:chrV 34842

**chrV L 34,847_34,847** – PGSP754 [bzg111] (*pol32*)

ACACCCACACCCACACCCACA:C:TCCACTGCAGGTCCTTCATCCACGCCAAATCCTAGTAGT

| |||||||||||||||||||||||||||||||||||||||

:C:TCCACTGCAGGTCCTTCATCCACGCCAAATCCTAGTAGT

:chrV 37847

**chrV L 34,847_34,847** – PGSP937 (*sgs1*)

ACACCCACACACACCCACA:C:TCCACTGCAGGTCCTTCATCCACGCCAAATCCTAGAAGTAACAC

| ||||||||||||||||||||||||||||||||||||||||||||

:C:TCCACTGCAGGTCCTTCATCCACGCCAAATCCTAGTAGTAACAC

:chrV 34847

**chrV L 34,849_34,852** – PGSP4356 (*exo1 sgs1*)

ACCCACACCCACACACCACAC:CCAC:TGCAGGTCCTTCATCCACGCCAAATCCTAGTA

|||| ||||||||||||||||||||||||||||

:CCAC:TGCAGGTCCTTCATCCACGCCAAATCCTAGTA

:chrV 34849

**chrV L 34,849_34,852** – PGSP4693 (*sgs1 yku80*)

CACACCACACCCACACCCACACACCCACAC:CCAC:TGCAGGTCCTTCATCCACGCCAAAT

|||| |||||||||||||||||||||||||

:CCAC:TGCAGGTCCTTCATCCACGCCAAAT

:chrV 34849

**chrV L 34,850_34,852** – PGSP948 (*sgs1*)

CCACACCCACACCCACGCCACACCCACACCCA:CAC:TGCAGGTCCTTCATCCACGCCAAATCCTAGAAG

||| |||||||||||||||||||||||||||||||||

:CAC:TGCAGGTCCTTCATCCACGCCAAATCCTAGTAG

:chrV 34850

**chrV L 34,849_34,852** – PGSP4822 (*sae2* chrV:34470-gRNA)

CCACACCCACACCCACACACCACAC:CCAC:TGCAGGTCCTTCATCCACGCCAAATCCTAGT

|||| |||||||||||||||||||||||||||||||

:CCAC:TGCAGGTCCTTCATCCACGCCAAATCCTAGT

:chrV 34849

chrV **L 34,849_34,852** – PGSP5061 (*exo1 yku80*)

ACACACCACACCACACCCACACCCA:CCAC:TGCAGGTCCTTCATCCACGCCAAATCCTAGT

|||| |||||||||||||||||||||||||||||||

:CCAC:TGCAGGTCCTTCATCCACGCCAAATCCTAGT

:chrV 34849

**chrV L 34,860_34,861** - PGSP774 [bzg086] (*exo1*)

CACACCCACACACCACACCCACACAC:CC:TTCATCCACGCCAAATCCTAGTAGTAACACCACACCAACT

|| ||||||||||||||||||||||||||||||||||||||||

:CC:TTCATCCACGCCAAATCCTAGTAGTAACACCACACCAACT

:chrV 34860

**chrV L 34,860_34,861** – PGSP4836 (wild-type chrV:34470-gRNA)

ACCCACACCCACACCCACACCCACA:CC:TTCATCCACGCCAAATCCTAGTAGTAACACCAC

|| |||||||||||||||||||||||||||||||||

:CC:TTCATCCACGCCAAATCCTAGTAGTAACACCAC

:chrV 34860

**chrV L 34,864_34,865** – PGSP331 (*rad52*)

ACAACACCCTCCACTGCAGGTCCTA:CA:TCCACGCCAAATCCTAGTAGTAACACCACACCA

|| |||||||||||||||||||||||||||||||||

:CA:TCCACGCCAAATCCTAGTAGTAACACCACACCA

:chrV 34864

**chrV L 34,867_34,870** – PGSP4358 (*sgs1 exo1*)

ACACCACACCCACACCACACCACAC:CCAC:GCCAAATCCTAGTAGTAACACCACACCAACTCAT

|||| ||||||||||||||||||||||||||||||||||

:CCAC:GCCAAATCCTAGTAGTAACACCACACCAACTCAT

:chrV 34867

**chrV L 34,867_34,870** – PGSP422 [bzg098] (*rrm3*), PGSP423 [bzg099] (*rrm3*)

ACACCCACACCACACCCACACCACAC:CCAC:GCCAAATCCTAGTAGTAACACCACACCAACTCATCCTAC

|||| |||||||||||||||||||||||||||||||||||||||

:CCAC:GCCAAATCCTAGTAGTAACACCACACCAACTCATCCTAC

:chrV 34867

**chrV L 34,868_34,870** - PGSP780 [bzg092] (*exo1*)

CCACACCCACACACACCACACCCACA:CAC:GCCAAATCCTAGTAGTAACACCACACCAACTCATCCTAC

||| |||||||||||||||||||||||||||||||||||||||

:CAC:GCCAAATCCTAGTAGTAACACCACACCAACTCATCCTAC

:chrV 34868

**chrV L 34,868_34,870** – PGSP4896 (*yen1*)

ACCACACCCACACACCACACCCACA:CAC:GCCAAATCCTAGTAGTAACACCACACCAACTC

||| ||||||||||||||||||||||||||||||||

:CAC:GCCAAATCCTAGTAGTAACACCACACCAACTC

:chrV 34868

**chrV L 34,872_34,874** – PGSP427 [bzg103] (*rrm3*)

CACCACACCCACACCCACACCACAC:CCA:AATCCTAGTAGTAACACCACACCAACTCATCCTACATCCGA

:|||:|||||||||||||||||||||||||||||||||||||||||

:CCA:AATCCTAGTAGTAACACCACACCAACTCATCCTACATCCGA

:chrV 34872

**chrV L 34,878_34,879** – PGSP465 [bzg125] (*rad10*)

CACACCCACACCCACACCCACA:CC:TAGTAGTAACACCACACCAACTCATCCTACATCCGAAAAGGATACAA

|| |||||||||||||||||||||||||||||||||||||||||||||||

:CC:TAGTAGTAACACCACACCAACTCATCCTACATCCGAAAAGGATACAA

:chrV 34878

**chrV L 34,878_34,879** – PGSP467 [bzg127] (*rad10*)

CCCACACCACACCCACACCCACACCCACA:CC:TAGTAGTAACACCACACCAACTCATCCTACATCCGAAAAGG

|| |||||||||||||||||||||||||||||||||||||||||

:CC:TAGTAGTAACACCACACCAACTCATCCTACATCCGAAAAGG

:chrV 34878

**chrV L 34,888_34,898** – PGSP4576 [bzg128] (*rad10*)

ACCCACACACCCACACAC:ACACCACACCA:ACTCATCCTACATCCGAAAAGGATACAAAGG

||||||||||| |||||||||||||||||||||||||||||||

:ACACCACACCA:ACTCATCCTACATCCGAAAAGGATACAAAGG

:chrV 34888

**chrV L 34,888_34,898** – PGSP4721 (*sae2 yku80 pif1*)

CACCCACACCCACACACCACACCCAC:ACACCACACCA:ACTCATCCTACATCCGAAAAGGATACAAAG

||||||||||| ||||||||||||||||||||||||||||||

:ACACCACACCA:ACTCATCCTACATCCGAAAAGGATACAAAG

:chrV 34888

**chrV L 34,888_34,898** – PGSP4623 (*sae2 pol32*)

CACCACACCCACACCCACACCC:ACACCACACCA:ACTCATCCTACATCCGAAAAGGATACAAAGG

||||||||||| |||||||||||||||||||||||||||||||

:ACACCACACCA:ACTCATCCTACATCCGAAAAGGATACAAAGG

:chrV 34888

**chrV L 34,891_34,900** – PGSP4824 (*sae2* chrV:34470-gRNA)

CACACACCACACCCACACACCACAC:CCACACCAAC:TCATCCTACATCCGAAAAGGATACA

|||||||||| |||||||||||||||||||||||||

:CCACACCAAC:TCATCCTACATCCGAAAAGGATACA

:chrV 34891

**chrV L 34,892_34,898** – PGSP4723 (*sae2 yku80 pif1*)

CACCCACACACCACACCCA:CACACCA:ACTCATCCTACATCCGAAAAGGATACAA

||||||| ||||||||||||||||||||||||||||

:CACACCA:ACTCATCCTACATCCGAAAAGGATACAA

:chrV 34892

**chrV L 35,140_35,142** – PGSP340 (*rad52*)

ACCCACACCCACACCCACACCCACA:CCC:TGTCGTCTGTAAATTTGAAGAAGATTATTGAT

||| ||||||||||||||||||||||||||||||||

:CCC:TGTCGTCTGTAAATTTGAAGAAGATTATTGAT

:chrV 35140

**chrV L 35,187_35,193** – PGSP225 (*mus81*)

CCACACACACCACACCCACACACCACACCCACAC:CCAACCA:TGATGAGTATAGTCCCTTACATTGAT

||||||| ||||||||||||||||||||||||||

:CCAACCA:TGATGAGTATAGTCCCTTACATTGAT

:chrV 35187

**chrV L 35,388_35,391** – PGSP4841 (wild-type chrV:34470-gRNA)

CACACACACCACACCCACACCACAC:CCAC:TAATGGCTAGTGACTGTCAATCTTATGTTAC

|||| |||||||||||||||||||||||||||||||

:CCAC:TAATGGCTAGTGACTGTCAATCTTATGTTAC

:chrV 35388

**chrV L 35,542_35,545** – PGSP223 (*mus81*)

CCACACCCACACCCACACACACCACACCCAC:ACCC:GGATTCTAGAACTACATCTTTTAGTTCTACCAGT

|||| ||||||||||||||||||||||||||||||||||

:ACCC:GGATTCTAGAACTACATCTTTTAGTTCTACCAGT

:chrV 35542

**chrV L 35,913_35,919** – PGSP4942 (*pif1 yku80*)

CACCACACCCACACACACCACACCC:ACACCAA:TAATAACAAAAGATTTAGTGAATAATGA

||||||| ||||||||||||||||||||||||||||

:ACACCAA:TAATAACAAAAGATTTAGTGAATAATGA

:chrV 35913

**chrV L 35,916_35,919** – PGSP4939 (*pif1 yku80*)

CCACACCCACACCACACCCACACAC:CCAA:TAATAACAAAAGATTTAGTGAATAATGATAA

|||| |||||||||||||||||||||||||||||||

:CCAA:TAATAACAAAAGATTTAGTGAATAATGATAA

:chrV 35916

**chrV L 36,214_36,216** – PGSP228 (*mus81*)

ACACCCACACCACACCCACAACCACACCCAC:ACC:TAAACGAATTAGGAGAATTTAAGGTAATTAATAG

||| ||||||||||||||||||||||||||||||||||

:ACC:TAAACGAATTAGGAGAATTTAAGGTAATTAATAG

:chrV 36214

**chrV L 36,424_36,430** – PGSP4655 (*sae2 mus81*)

ACACCACACCCACACCACACCCACACACCA:CACACCC:TTCTCTTCTGTCTTGCCTTCTAATGAATAAC

||||||| |||||||||||||||||||||||||||||||

:CACACCC:TTCTCTTCTGTCTTGCCTTCTAATGAATAAC

:chrV 36424

**chrV L 37,086_37,089** –PGSP2219 (*pif1*)

ACACCCACACCACACCCACA:CCCA:TTTAACGCGCTTGGGTTCAAACAGATCCATTTCTTGATC

|||| |||||||||||||||||||||||||||||||||||||||

:CCCA:TTTAACGCGCTTGGGTTCAAACAGATCCATTTCTTGATC

:chrV 37086

**chrV L 38,004_38,005** – PGSP466 [bzg126] (*rad10*)

CCCACACCCACACCCACACACCACACCCAC:AC:TTTGTAGAGAGTAAATCGGATTTAATCTTTGCTAATT

|| |||||||||||||||||||||||||||||||||||||

:AC:TTTGTAGAGAGTAAATCGGATTTAATCTTTGCTAATT

:chrV 38004

**chrV L 38,086_38,091** – PGSP2225 (*pif1*)

CCACACCACACCCACACCAC:ACCCA:GTTGCGGCTTGTTCTTAATGTTTTTAGCCTTCGAAGCCTACTC

||||| |||||||||||||||||||||||||||||||||||||||||||

:ACCCA:GTTGCGGCTTGTTCTTAATGTTTTTAGCCTTCGAAGCATACTC

:chrV 38086

**chrV L 38,086_38,090** – PGSP4836 (wild-type chrV:34470-gRNA)

ACCACACCCACACACCCACACCCAC:ACCCA:GTTGCGGCTTGTTCTTAATGTTTTTAGCCT

||||| ||||||||||||||||||||||||||||||

:ACCCA:GTTGCGGCTTGTTCTTAATGTTTTTAGCCT

:chrV 38086

**chrV L 38,291_38,294** – PGSP4722 (*sae2 yku80 pif1*)

ACACCACACCCACACACCAC:ACCC:TGCCCAGCGTCAATAGACTTTGGTTGATTGAA

|||| ||||||||||||||||||||||||||||||||

:ACCC:TGCCCAGCGTCAATAGACTTTGGTTGATTGAA

:chrV 38291

**chrV L 38,297_38,300** – PGSP2217 (*pif1*)

ACACCACCACACCCACACACCACA:CCCA:GCGTCAATAGACTTTGGTTGATTGAACCAGCTTCTTTGGCA

|||| |||||||||||||||||||||||||||||||||||||||||

:CCCA:GCGTCAATAGACTTTGGTTGATTGAACCAGCTTCTTTGGCA

:chrV 38297

**chrV L 38,391_38,396** – PGSP337 (*rad52*)

CCCACACCACACCCACACACCACAC:CCACAA:GATTCATTTTGGAAATTCTAAATAGTTCA

|||||| |||||||||||||||||||||||||||||

:CCACAA:GATTCATTTTGGAAATTCTAAATAGTTCA

:chrV 38391

**chrV L 38,543_38,545** - PGSP776 [bzg088] (*exo1*)

ACCACACCCACACACCCACACCCACA:CCC:TCCATAGCATTTGTTATGTGAAATTCTTGCAAATTCTGG

||| |||||||||||||||||||||||||||||||||||||||

:CCC:TCCATAGCATTTGTTATGTGAAATTCTTGCAAATTCTGG

:chrV 38543

**chrV L 38,543_38,545** – PGSP4706 (*sae2* *pif1*)

CCACACCCACACACCACACCCACACA:CCC:TCCATAGCATTTGTTATGTGAAATTCTTGCAAATTCTGG

||| |||||||||||||||||||||||||||||||||||||||

:CCC:TCCATAGCATTTGTTATGTGAAATTCTTGCAAATTCTGG

:chrV 38543

**chrV L 38,575_38,578** – PGSP4937 (*pif1 yku80*)

ACACACCACACCCACACA:CAAA:TTCTGGATGTATATACCATTAGTTTGGTCAT

|||| |||||||||||||||||||||||||||||||

:CAAA:TTCTGGATGTATATACCATTAGTTTGGTCAT

:chrV 38575

**chrV L 38,715_38,716** – PGSP339 (*rad52*)

ACCACACCCACACACCACACCACAC:CC:TGTTGTTGTTGATACTGTTGCTTGATGGTATTC

|| |||||||||||||||||||||||||||||||||

:CC:TGTTGTTGTTGATACTGTTGCTTGATGGTATTC

:chrV 38715

**chrV L 39,155_39,157** – PGSP223 (*mus81*)

CCCACACCCACACCACACCCACACCCACACAC:CCA:TATACCAGTACGGTGCAATTGTAACCTTTAAT

||| ||||||||||||||||||||||||||||||||

:CCA:TATACCAGTACGGTGCAATTGTAACCTTTAAT

:chrV 39155

**chrV L 39,251_39,252** – PGSP2216 (*pif1*)

CCACACCCACACACCACACCCACAC:CC:GAAGACTTTGTCCACTGTGTATCTCTTGGCATTCATCTGAG

|| |||||||||||||||||||||||||||||||||||||||||

:CC:GAAGACTTTGTCCACTGTGTATCTCTTGGCATTCATTTGAG

:chrV 39251

**chrV L 39,363_39,369** – PGSP2222 (*pif1*)

ACACACCACACCCACACAC:CCACAAC:GGAGCTTTTCATACTAATTTCCCTTTCATTCCTTCGT

:CCACAAC:GGAGCTTTTCATACTAATTTCCCTTTCATTCCTTC

:chrV 39363

**chrV L 39,659_39,264** – PGSP2224 (*pif1*)

ACCCACACCCACACACCACACCACACCCAC:ACACCC:GCTGTTGAGAAGCGGGTCACAAAGGAAGTCTTT

|||||| |||||||||||||||||||||||||||||||||

:ACACCC:GCTGTTGAGAAGCGGGTCACAAAGGAAGTCTTT

:chrV 39659

**chrV L 39,682_39,685** – PGSP4568 [bzg120] (*pol32*)

ACACCCACACCCACACCCACACACCACACCCACA:CACA:AAGGAAGTCTTTAGATGATGTTATTGTTGCGG

|||| ||||||||||||||||||||||||||||||||

:CACA:AAGGAAGTCTTTAGATGATGTTATTGTTGCGG

:chrV 39,682

**chrV L 39,718_39,719** – PGSP3614 (*yku80*)

CCACACCCACACACCCACACCCACACA:CC:TTGTTTGTTTGTTTTGTTTATTTCGCGTACCTAATACAT

|| |||||||||||||||||||||||||||||||||||||||

:CC:TTGTTTGTTTGTTTTGTTTATTTCGCGTACCTAATACAT

:chrV 39718

**chrV L 39,749_39,750** – PGSP229 (*mus81*)

ACCCACACCCACACACACACCACACCCACACCCACACCACAC:CC:TAATACATCGTCACCACACACGAAAACGAAA

|| |||||||||||||||||||||||||||||||

:CC:TAATACATCGTCACCACACACGAAAACGAAA

:chrV 39749

**chrV L 39,762_39,771** – PGSP2223 (*pif1*)

ACCCACACCCACACACCA:CACCACACAC:GAAAACGAAAACATTTGATCAGATAAGTGAT

|||||||||| |||||||||||||||||||||||||||||||

:CACCACACAC:GAAAACGAAAACATTTGATCAGATAAGTGAT

:chrV 39762

**chrV L 39,764_39,771** – PGSP4713 (*sae2 pif1*)

CCACACCCACACCCACACCACACCCACAC:CCACACAC:GAAAACGAAAACATTTGATCAGATAAGTGAACTGCAAA

|||||||| ||||||||||||||||||||||||||||||||||||||

:CCACACAC:GAAAACGAAAACATTTGATCAGATAAGTGATCTGCAAA

:chrV 39764

**chrV L 39,764_39,771** – PGSP2214 (*pif1*)

CACACCCACACACCACACCCACACCACAC:CCACACAC:GAAAACGAAAACATTTGATCAGATAAGTGATCTGCAAA

|||||||| ||||||||||||||||||||||||||||||||||||||

:CCACACAC:GAAAACGAAAACATTTGATCAGATAAGTGATCTGCAAA

:chrV 39764

**chrV L 39,765_39,771** – PGSP2221 (*pif1*)

CACACACCACACCCA:CACACAC:GAAAACGAAAACATTTGATCAGATAAGTGAT

||||||| |||||||||||||||||||||||||||||||

:CACACAC:GAAAACGAAAACATTTGATCAGATAAGTGAT

:chrV 39765

**chrV L 39,968_39,969** – PGSP4697 (*sgs1 yku80*)

ACACCCACACACACAC:AC:TCCGACTTGTAACCTCGAGACGCCTAAGGAA

|| |||||||||||||||||||||||||||||||

:AC:TCCGACTTGTAACCTCGAGACGCCTAAGGAA

:chrV 39968

**chrV L 39,982_39,983** - PGSP782 [bzg094] (*exo1*)

CACCCACACCCACACCACACCCACAC:CC:TCGAGACGCCTAAGGAAAGAAAAAAGAAAAAAAAAGCAG

|| |||||||||||||||||||||||||||||||||||||||

:CC:TCGAGACGCCTAAGGAAAGAAAAAGAAAAAAAAAAGCAG

:chrV 39982

**chrV L 40,186_40,190** – PGSP5062 (*exo1 yku80*)

CCACACCCACACCCACACACCCAC:ACAAA:TCTTGACCACCACCGTTGTAGATTAAAACA

||||| ||||||||||||||||||||||||||||||

:ACAAA:TCTTGACCACCACCGTTGTAGATTAAAACA

:chrV 40186

**chrV L 40,197_40,204** – PGSP4714 (*sae2 pif1*)

ACCCACACACCACACCCACACCACACCCACAC:CCACCACC:GTTGTAGATTAAAACATTTGGAGTGTCT

|||||||| ||||||||||||||||||||||||||||

:CCACCACC:GTTGTAGATTAAAACATTTGGAGTGTCT

:chrV 40197

**chrV L 40,314_40,317** – PGSP942 (*sgs1*)

ACACCACACCCACACACACCACACCCACA:CCCA:ATTCGAAAAATTCACTATCAGAACCTGGTTGCA

|||| |||||||||||||||||||||||||||||||||

:CCCA:ATTCGAAAAATTCACTATCAGAACCTGGTTGCA

:chrV 40314

**chrV L 40,403_40,406** – PGSP4653 (*sae2 mus81*)

CACCACACCCACACACCCACACACCACACCACACCCAC:ACCC:GATAAAGTGGCGGTGGCGTCATCAC

:ACCC:GATAAAGTGGCGGTGGCGTCATCAC

:chrV 40403

**chrV L 40,460_40,462** – PGSP4354 (*exo1 sgs1*)

CACCACACCACACACCACACACCACACCCACAC:ACC:TGGGGCGAAAACGTCGACACACTTACCCCAG

||| |||||||||||||||||||||||||||||||

:ACC:TGGGGCGAAAACGTCGACACACTTACCCCAG

:chrV 40460

**chrV L 40,596_40,598** – PGSP4351 (*exo1 sgs1*)

CACCCACACCCACACACACACCACAC:CCA:GCAGCCACGGCAAAGTGAATACCGACTTCAACGGC

||| |||||||||||||||||||||||||||||||||||

:CCA:GCAGCCACGGCAAAGTGAATACCGACTTCAACGGC

:chrV 40596

**chrV L 40,707_40,709** – PGSP334 (*rad52*)

CACACACCACACCCACACACCCACA:CCC:TTTTTCTTTTCTTGGGCTTCTTTTTGGTGCGC

||| ||||||||||||||||||||||||||||||||

:CCC:TTTTTCTTTTCTTGGGCTTCTTTTTGGTGCGC

:chrV 40707

**chrV L 40,726_40,726** – PGSP2215 (*pif1*)

ACCCACACACCCACACACACACCACACCCACACA:C:TTCTTTTTGGTGCGCCTTTGCGGCATATTCGAC

| |||||||||||||||||||||||||||||||||

:C:TTCTTTTTGGTGCGCCTTTGCGGCATATTCGAC

:chrV 40726

**chrV L 40,758_40,762** – PGSP2220 (*pif1*)

CCACACCCACACCCACACACCACACCCAC:ACACC:TTTGACGACATCAGACATGGTACCAGACCCGTTT

||||| ||||||||||||||||||||||||||||||||||

:ACACC:TTTGACGACATCAGACATGGTACCAGACCCGTTT

:chrV 40758

**chrV L 40,758_40,762** – PGSP4936 (*pif1 yku80*)

CACACCACACCCACACACCACACCC:ACACC:TTTGACGACATCAGACATGGTACCAGACCC

||||| ||||||||||||||||||||||||||||||

:ACACC:TTTGACGACATCAGACATGGTACCAGACCC

:chrV 40758

**chrV L 40,971_40,976** – PGSP4725 (*sae2 yku80* *pif1*)

CCCACACACCACACCCACACACC:ACACCC:GTGTCAATAACATAGGACGTGACACCGCGACCGGC

|||||| |||||||||||||||||||||||||||||||||||

:ACACCC:GTGTCAATAACATAGGACGTGACACCGCGACCGGC

:chrV 40971

**chrV L 41,041_41,046** – PGSP4935 (*pif1 yku80*)

ACACACCAC:ACCCCA:GGTTGAGGCGCTCTCTGTGGGAAATACGG

|||||| |||||||||||||||||||||||||||||

:ACCCCA:GGTTGAGGCGCTCTCTGTGGGAAATACGG

:chrV 41041

**chrV L 41,078_41,086** – PGSP336 (*rad52*)

CCACACCCACACCCACACCCACACA:CAACCCCCA:TGGGGCGCTATTTTGAGTGTCAAATT

||||||||| ||||||||||||||||||||||||||

:CAACCCCCA:TGGGGCGCTATTTTGAGTGTCAAATT

:chrV 41078

**chrV L 41,207_41,209** – PGSP224 (*mus81*)

CACCCACACCACACCCACACACCACACACCAC:ACC:GGAGAAAAGATTATCGATGTTGAATGAGTCCTGG

||| ||||||||||||||||||||||||||||||||||

:ACC:GGAGAAAAGATTATCGATGTTGAATGAGTCCTGG

:chrV 41207

**chrV L 41,538_41,539** – PGSP2218 (*pif1*)

CCACACCCACACACCCACACAC:CC:TTTCATCTTCTTCTCCTCCACCTTATTTTCGTGGCA

|| ||||||||||||||||||||||||||||||||||||

:CC:TTTCATCTTCTTCTCCTCCACCTTATTTTCGTGGCA

:chrV 41538

**chrV L 41,578_41,582** – PGSP230 (*mus81*)

ACCCACACCCACACACCACACCCACACCACACCCA:CACCC:TTATGGTGAGGCTTCTTCTTCTTATCA

||||| |||||||||||||||||||||||||||

:CACCC:TTATGGTGAGGCTTCTTCTTCTTATCA

:chrV 41578

**chrV L 41,578_41,582** – PGSP4913 (*slx1*)

CCACACCACACCCACACCACACCCA:CACCC:TTATGGTGAGGCTTCTTCTTCTTATCATCA

||||| ||||||||||||||||||||||||||||||

:CACCC:TTATGGTGAGGCTTCTTCTTCTTATCATCA

:chrV 41578

**chrV L 41,652_41,654** – PGSP4361 (*exo1 sgs1*)

ACCCACACACCACACCCACACCCACACACACA:CCC:TTTCCTCCCTCGTGGGCGCTCCCATGAGAGC

||| |||||||||||||||||||||||||||||||

:CCC:TTTCCTCCCTCGTGGGCGCTCCCATGAGAGC

:chrV 41652

**chrV L 41,812_41,815** – PGSP330 (*rad52*)

ACCACACCCACACCCACACCCACAC:CCAC:TTTTCTGGGTCTCTCGTGGTGATCTTCCTTG

|||| |||||||||||||||||||||||||||||||

:CCAC:TTTTCTGGGTCTCTCGTGGTGATCTTCCTTG

:chrV 41812

**chrV L 42138_42141** – PGSP4938 (*pif1 yku80*)

CACACACCACACCCACACACCACAC:CCAC:TGCAGTGGCATTACTGAGCCCAAATTTTGCT

|||| |||||||||||||||||||||||||||||||

:CCAC:TGCAGTGGCATTACTGAGCCCAAATTTTGCT

:chrV 42138

**chrV L 42,064_42,066** – PGSP4893 (*yen1*)

ACCCCACACCCACACCACACCCACA:CCC:TTTTATATTCATCGCGCTCTTTATCGCGGGTG

||| ||||||||||||||||||||||||||||||||

:CCC:TTTTATATTCATCGCGCTCTTTATCGCGGGTG

:chrV 42064

**chrV L 42160_42165** – PGSP5064 (*exo1 yku80*)

ACCCACACACCACACCCACACCACA:CCCAAA:TTTTGCTTTTTCAACAAGTCACCTAAATT

|||||| |||||||||||||||||||||||||||||

:CCCAAA:TTTTGCTTTTTCAACAAGTCACCTAAATT

:chrV 42160

**chrV L 42,185_42,188** – PGSP420 [bzg096] (*rrm3*)

CCCACACCCACACACCACACCCACA:CACC:TAAATTTCCAAAGCCGAAAGCCCTGCTACTCTGCTAAC

:CACC:TAAATTTCCAAAGCCGAAAGCCCTGCTACTCTGCTAAC

:chrV 42185

**chrV L 42,677_42,680** – PGSP425 [bzg101] (*rrm3*)

CACACCCACACACCACACCCACACA:CCCC:TTTTCTCCCCCCCTGTTTCTCGTCCATCTCTATCTGTC

|||| ||||||||||||||||||||||||||||||||||||||

:CCCC:TTTTCTCCCCCCCTGTTTCTCGTCCATCTCTATCTGTC

:chrV 42677

**chrV L 42,807_42,810** – PGSP1993 (*sae2*)

ACCACACCCACACCCACACCACACCCACACCCAC:ACAC:TCTTCTGCAGAGGATTTTCGTACACAACGG

|||| ||||||||||||||||||||||||||||||

:ACAC:TCTTCTGCAGAGGATTTTCGTACACAACGG

:chrV 42807

**chrV L 43,023_43,026** –PGSP946 (*sgs1*)

CACCACACCCACACACCACACCCACACCCAC:ACCC:TTACTTGCACGATTAGCAGATTGTTCAGAGAGC

|||| |||||||||||||||||||||||||||||||||

:ACCC:TTACTTGCACGATTAGCAGATTGTTCAGAGAGC

:chrV 43023

**chrV L 43,023_43,026** – PGSP5060 (*exo1 yku80*)

CACACACACCACACCCACACACCAC:ACCC:TTACTTGCACGATTAGCAGATTGTTCAGAGA

|||| |||||||||||||||||||||||||||||||

:ACCC:TTACTTGCACGATTAGCAGATTGTTCAGAGA

:chrV 43023

**chrV L 68,851_68,854** – PGSP4889 (*sae2 rad52*)

GAGTAAAAGTGAGTGTAAGCCTAGGAAAGGA:GGTG:TGGGTGTGGTGTGGGTGTGGTGTGG

||||||||||||||||||||||||||||||| ||||

GAGTAAAAGTGAGTGTAAGCCTAGGAAAGGA:GGTG:

chrV 68854:

**chrV L 68,851_68,856** – PGSP4882 (*sae2 rad52*)

GTAAAAGTGAGTGTAAGCCTAGGAAAGGA:GGTGGT:GTGTGGGTGTGGGTGTGGTGTGGG

||||||||||||||||||||||||||||| ||||||

GTAAAAGTGAGTGTAAGCCTAGGAAAGGA:GGTGGT:

chrV 68856:

**chrV L 75,107_75,110** – PGSP5070 (*exo1 yku80*)

AAAGGCGAAGAAACTTGGAATAGTGTCTTCA:GTGG:GTGTGGTGTGGGTGTGGTGTGTGGG

||||||||||||||||||||||||||||||| ||||

AAAGGCGAAGAAACTTGGAATAGTGTCTTCA:GTGG:

chrV 75110:

**chrV L 83,962_83,965** –PGSP4720 (*sae2 pif1 yku80*)

GGTTTCGACATCCACGTCTCTCCAGTAAACCCAGGA:GTGG:GTGTGGTGTGGGTGTGGTGTGTGTG

|||||||||||||||||||||||||||||||||||| ||||

GGTTTCGACATCCACGTCTCTCCAGTAAACCCAGGA:GTGG:

chrV 83965:

**chrV L 86,027_86,030** –PGSP4720 (*sae2 pif1 yku80*)

CGGTGACACTAAGGATGATGTCAAGGCTCCAGAA:GGTG:TGTGGGTGTGGTGTGTGGTGTGGTGTGTGTG

|||||||||||||||||||||||||||||||||| ||||

CGGTGACACTAAGGATGATGTCAAGGCTCCAGAA:GGTG:
 chrV 86030:

**chrV L 107,567_107,571** –PGSP4704 (*sae2 pif1*)

GTTGAAGTTAATTTAGCGGCTATCCCAT:TGGGT:GTGGTGTGTGGGTGTGTGGGTGTGGGTGTGGTGTG

|||||||||||||||||||||||||||| |||||

GTTGAAGTTAATTTAGCGGCTATCCCAT:TGGGT:

chrV 107571:

**chrV L 116,832_116,837** –PGSP4718 (*sae2 pif1 yku80*)

ACGCATTGGGTCAACAGTATAGAACCGTGGATGA:TGTGGT:GTGGGTGTGGGTGTGGTG

|||||||||||||||||||||||||||||||||| ||||||

ACGCATTGGGTCAACAGTATAGAACCGTGGATGA:TGTGGT:

chrV 116837:

**chrV L 140,749_140,750** –PGSP4622 (*sae2 pol32*)

GTATGGGGTATGGGGGCATCATCGATTGTTCTCCGGTAAC:GG:GTGTGGGTGTGGTGTGTGGGT

|||||||||||||||||||||||||||||||||||||||| ||

GTATGGGGTATGGGGGCATCATCGATTGTTCTCCGGTAAC:GG:

chrV 140749:

*Junctions are sorted based on the coordinates of the telomere-like sequences that the adjacent to the telomere (sequence between the colons).
