# Supplementary material for: Mechanisms underlying genome instability mediated by formation of foldback inversions in Saccharomyces cerevisiae
Source: eLife. 2020 Aug 7;9:e58223. doi: 10.7554/eLife.58223 (PMC7467729; doi:10.7554/eLife.58223)
Supplement: Supplementary file 5. [file elife-58223-supp5.docx]

**Supplementary File 5.** **Micro- and non-homology-mediated translocation junction sequences**

PGSP232 (*mus81*)

chrXII 459803-:

AGCATAGGAAGCCAAGAAACTAGAAAAAAAAAA:AAAAAAAAA-AAAAAAAAAAG:

||||||||||||||||||||||||||||||||| ||||||||| |||||||||||

**AGCATAGGAAGCCAAGAAACTAGAAAAAAAAAA:AAAAAAAAAGAAAAAAAAAAG:CAGCTGAAATTTTTCTAAATGA**

||| ||||||||||||||||| ||||||||||||||||||||||

:AAAGAAAAAGAAAAAAAAAAG:CAGCTGAAATTTTTCTAAATGA

:chrV 39999+

PGSP329 (*rad52*) – junction for circularization of chrVIII (unrelated to GCR)

chrVIII 455,909+:

TTGAATCTGGAAAATCCGCACAGGCGCGCCAGCA:TA:

|||||||||||||||||||||||||||||||||| ||

**TTGAATCTGGAAAATCCGCACAGGCGCGCCAGCA:TA:TATCCATTTCAATGTCTGGCTCATCTTCGTCCTC**

|| ||||||||||||||||||||||||||||||||||

:TA:TATCCATTTCAATGTCTGGCTCATCTTCGTCCTC

:chrVIII 340,154+

PGSP338 (*rad52*)

ChrIX 335548+:

AGTTTAAAGAAGAATGAAGTTTAATAA:CCCCCTTTT:

||||||||||||||||||||||||||| |||||||||

**AGTTTAAAGAAGAATGAAGTTTAATAA:CCCCCTTTT:ATATTCATCGCGCTCTTTATCGCGGGTGTGTTTG**

||||||||| ||||||||||||||||||||||||||||||||||

:CCCCCTTTT:ATATTCATCGCGCTCTTTATCGCGGGTGTGTTTG

:chrV 42062+

PGSP424 [bzg100] (*rrm3*)

chrX 475615+:

CTTCCAATCTGACATTGAACTACATCCTGAGCA:ACCGAG:

||||||||||||||||||||||||||||||||| ||||||

**CTTCCAATCTGACATTGAACTACATCCTGAGCA:ACCGAG:TGTAAATAGAGTATTTTCTAACTTCATCTTTGCTT**

|||||| |||||||||||||||||||||||||||||||||||

:ACCGAG:TGTAAATAGAGTATTTTCTAACTTCATCTTTGCTT

:chrV 41921+

PGSP779 [bzg091] (*exo1*)

chrX 728598-:

TTCATCAAGAAAAATGCAGACAGGAAGGTACT:TTC-:

|||||||||||||||||||||||||||||||| |||

**TTCATCAAGAAAAATGCAGACAGGAAGGTACT:TTC-:GTCACGCAGTCCTTGGGTGAAATGGCTACATTCA**

||| ||||||||||||||||||||||||||||||||||

:TTCT:GTCACGCAGTCCTTGGGTGAAATGGCTACATTCA

:chrV 33065- (in CAN1 insertion)

PGSP940 (*sgs1*)

chrXI 326520+:

TAACCCAGACTCTTGGGTCGAAGAACTTCTTGTTTGGC:T:

|||||||||||||||||||||||||||||||||||||| |

**TAACCCAGACTCTTGGGTCGAAGAACTTCTTGTTTGGC:T:AACGCGCTTGGGTTCAAACAGATCCATTTCTTGAT**

| |||||||||||||||||||||||||||||||||||

:T:AACGCGCTTGGGTTCAAACAGATCCATTTCTTGAT

:chrV 37092+

PGSP945 (*sgs1*)

chrXII 170721-:

TCTCGGGGAACATATAC:ATATATATATATATATATATATGTATAT:

||||||||||||||||| |||||||||||||||||||| ||||||

**TCTCGGGGAACATATAC:ATATATATATATATATATAT--GTATAT:GTGTACATTTTCACG**

|||||||||||||||||||| |||||| ||||||||||||||||

:ATATATATATATATATATAT--GTATAT:GTGTACATTTTCACG

:chrV 31480-

PGSP3606 (*yku80*)

chrV 466011-:

CGTCTACTCCTTCCCCATCACATTTTTCT:AAA:

||||||||||||||||||||||||||||| |||

**CGTCTACTCCTTCCCCATCACATTTTTCT:AAA:GACTTTTTGGGACAAATTTTGGAATGTTGTA**

||| |||||||||||||||||||||||||||||||

:AAA:GACTTTTTGGGACAAATTTTGGAATGTTGTA

:chrV 31733- (CAN1 insertion)

PGSP3606 (*yku80*)

chrX 81482+:

TTTTGAACAAGATTACAAGCAATACCAA:AA:

|||||||||||||||||||||||||||| ||

**TTTTGAACAAGATTACAAGCAATACCAA:AA:CATGTTATGCTTGTTAATAAGCTGCTCAAA**

|| ||||||||||||||||||||||||||||||

:AA:CATGTTATGCTTGTTAATAAGCTGCTCAAA

:chrV 36848+

PGSP3612 (*yku80*)

chrX 81482+:

TTTTGAACAAGATTACAAGCAATACCAA:AA:

|||||||||||||||||||||||||||| ||

**TTTTGAACAAGATTACAAGCAATACCAA:AA:CATGTTATGCTTGTTAATAAGCTGCTCAAA**

|| ||||||||||||||||||||||||||||||

:AA:CATGTTATGCTTGTTAATAAGCTGCTCAAA

:chrV 36848+

PGSP3613 (*yku80*)

chrX 81482+:

TTTTGAACAAGATTACAAGCAATACCAA:AA:

|||||||||||||||||||||||||||| ||

**TTTTGAACAAGATTACAAGCAATACCAA:AA:CATGTTATGCTTGTTAATAAGCTGCTCAAA**

|| ||||||||||||||||||||||||||||||

:AA:CATGTTATGCTTGTTAATAAGCTGCTCAAA

:chrV 36848+

PGSP3616 (*yku80*)

chrXIV 763417-:

TATAGCCCGTCAGTAATACCTTGGTCTAATGAATGT:CTGCAAAC:

|||||||||||||||||||||||||||||||||||| ||||||||

**TATAGCCCGTCAGTAATACCTTGGTCTAATGAATGT:CTGCAAAC:CCCAGAAAATCCGTTCCAAGAGCC**

|||||||| ||||||||||||||||||||||||

:CTGCAAAC:CCCAGAAAATCCGTTCCAAGAGCC

:chrV 32538- (in CAN1 insertion)

PGSP3681 [bzg074] (*sae2 tel1*)

Delta sequence:

GGAATCCCAACAATTATCTCAACATTCACATATTTCTCA:T:

||||||||||||||||||||||||||||||||||||||| |

**GGAATCCCAACAATTATCTCAACATTCACATATTTCTCA:T:TTTTATACCTTTAACTATCATCATCAATAATTTCTTT**

| |||||||||||||||||||||||||||||||||||||

:T:TTTTATACCTTTAACTATCATCATCAATAATTTCTTT

:chrXII 818470+

PGSP4065 [bzg155] (*sae2 rrm3*) – generates a circular chrV

chrV 556943+:

CATATATGAACCACGAACAACGGAAAATATA:GAA:

||||||||||||||||||||||||||||||| |||

**CATATATGAACCACGAACAACGGAAAATATA:GAA:AAGAAAATAAAACTAGTATTATGATGACAAACCAACGCAAAGG**

||| |||||||||||||||||||||||||||||||||||||||||||

:GAA:AAGAAAATAAAACTAGTATTATGATGACAAACCAACGCAAAGG

:chrV 30395+

PGSP4592 (*sae2 rad10*)

chrVII 251283+:

TGTTTGGGGCCCCGACTCCA:AT:

|||||||||||||||||||| ||

**TGTTTGGGGCCCCGACTCCA:AT:GGTATTAATTTCAACACATTTAAAAACTACA**

|| |||||||||||||||||||||||||||||||

:AT:GGTATTAATTTCAACACATTTAAAAACTACA

:chrV 34522

PGSP4598 [bzg148] (*sae2 rrm3*)

chrV 31598+ in inserted CAN1:

TCATCGATAAAAATAAATATACTGAGATTATA:GTAAGCTC:

|||||||||||||||||||||||||||||||| ||| ||||

**TCATCGATAAAAATAAATATACTGAGATTATA:GTATGCTC:TCTTCACAAGAAGCAAGTCAGGCTGCCATAGATTTGA**

|||||||| |||||||||||||||||||||||||||||||||||||

:GTATGCTC:TCTTCACAAGAAGCAAGTCAGGCTGCCATAGATTTGA

:2 micron 5888-

PGSP4601 [bzg151] (*sae2 rrm3*)

2 micron 2464-:

TTCTCACGTTTTCATGTCAAGCATATTGGG:CATC:

|||||||||||||||||||||||||||||| ||||

**TTCTCACGTTTTCATGTCAAGCATATTGGG:CATC:ATTGGAACTTTCATGCTTAGGCTTCATGCCCTTTCCTCCCT**

|||| |||||||||||||||||||||||||||||||||||||||||

:CATC:ATTGGAACTTTCATGCTTAGGCTTCATGCCCTTTCCTCCCT

:chrV 41620+

PGSP4612 (*sae2 pol32*)

repetitive subtelomeric sequence:

(for example) chrXII 1,068,740-:

CTTCTTGGCCAGTCCCGTAAGCCCAA:TACGCTG:

|||||||||||||||||||||||||| ||| |||

**CTTCTTGGCCAGTCCCGTAAGCCCAA:TAC-CTG:ATAATTGAGTGTGTTCGTCATAATTTTTA**

||| ||| |||||||||||||||||||||||||||||

:TAC-CTG:ATAATTGAGTGTGTTCGTCATAATTTTTA

:chrV 30090+

PGSP4621 (*sae2 pol32*)

chrV 477329-:

CCATAATGGTGTTAGTAAAAAAATAGGTGGATTTTTCA:GAAGA:

|||||||||||||||||||||||||||||||||||||| |||||

**CCATAATGGTGTTAGTAAAAAAATAGGTGGATTTTTCA:GAAGA:GTCTATTAGGAATGCCGAAACATTTGATAAAA**

||||| ||||||||||||||||||||||||||||||||

:GAAGA:GTCTATTAGGAATGCCGAAACATTTGATAAAA

:chrV 36135+

PGSP4650 (*sae2 mus81*)

chrI 135925+:

ACCATGCCCAGGATGATAGTGCCAGTATAGTGTCCGGG:CCT:

|||||||||||||||||||||||||||||||||||||| |||

**ACCATGCCCAGGATGATAGTGCCAGTATAGTGTCCGGG:CCT:GTCGTCTGTAAATTTGAAGAAGATTATTGATGT**

||| |||||||||||||||||||||||||||||||||

:CCT:GTCGTCTGTAAATTTGAAGAAGATTATTGATGT

:chrV 35141+

PGSP4656 (*sae2 mus81*)

Ty element:

ATATTTTTTAGTGGATGTCATATCAGAGTCCGCTGAGG::

||||||||||||||||||||||||||||||||||||||

**ATATTTTTTAGTGGATGTCATATCAGAGTCCGCTGAGG::TCCTGCTCGAGTTCAAATATTCATGTCATCAACAATCGT**

|||||||||||||||||||||||||||||||||||||||

::TCCTGCTCGAGTTCAAATATTCATGTCATCAACAATCGT

:chrV 35652+

PGSP4663 (*sae2 mus81*)

chrXIV 333145+:

GCAATGGGTCATATTCAAGCGGGTT:AAGG:

||||||||||||||||||||||||| ||||

**GCAATGGGTCATATTCAAGCGGGTT:AAGG:GCAGAACTCTTTGAATATCATTAC**

|||| ||||||||||||||||||||||||

:AAGG:GCAGAACTCTTTGAATATCATTAC

:chrV 31124- (in CAN1 insert)

PGSP4741 (*sae2 sgs1 yku80*)

chrV 30951- (inserted CAN1):

ACTCAGCCGTTCGCGATCAAAAATGCTGGTA:

||||||||||||||||||||||||||||||| <-- inserted junction sequence -->

ACTCAGCCGTTCGCGATCAAAAATGCTGGTA:AGGGCGAATTCCAGCACACTGGCGGCCGTG:CTAGCATT:

||||||||||||||||||||||||||||||| |||||||||||||||||||||||||||||| ||||||||

**ACTCAGCCGTTCGCGATCAAAAATGCTGGTA:AGGGCGAATTCCAGCACACTGGCGGCCGTG:CTAGTATT:ATGATGACAAACCA**

|||||||| ||||||||||||||

:CTAGTATT:ATGATGACAAACCA

:chrV 30410+

PGSP4771 (*sae2-S267A*)

chrV 94494+:

TCAAATTGCAGCACCCTTTAAAGGGTCAGACGTAGAACAT:CA:

|||||||||||||||||||||||||||||||||||||||| ||

**TCAAATTGCAGCACCCTTTAAAGGGTCAGACGTAGAACAT:CA:TGTGGTAGTGGGATTAGAGTGGTAGGGTAAGTATA**

|| |||||||||||||||||||||||||||||||||||

:CA:TGTGGTAGTGGGATTAGAGTGGTAGGGTAAGTATA

:chrXIV 7018-

PGSP4789 (*sae2-MT9*)

chrXII 457225-, 466362-:

TGACAATAAATAACGATACAGGGCCCATTCGGGTCT:TGTAATT:

|||||||||||||||||||||||||||||||||||| |||||||

**TGACAATAAATAACGATACAGGGCCCATTCGGGTCT:TGTAATT:TTTAACTATTGGTATATGTGTCCGTGACC**

||||||| |||||||||||||||||||||||||||||

:TGTAATT:TTTAACTATTGGTATATGTGTCCGTGACC

:chrV 42392+

PGSP4823 (*sae2 chrV:34,470-gRNA*)

chrVII 1081940-:

ATTTCACGAAATGATGGCACTATAGCACGTCCCTTGTTT:C:

||||||||||||||||||||||||||||||||||||||| |

**ATTTCACGAAATGATGGCACTATAGCACGTCCCTTGTTT:C:AGAAGGTTCTAAGATTAAATACGAGTTTCCGCCAAAT**

| |||||||||||||||||||||||||||||||||||||

:C:AGAAGGTTCTAAGATTAAATACGAGTTTCCGCCAAAT

:chrV 34471+

PGSP4825 (*sae2 chrV:34,470-gRNA*)

chrI 225635-:

CCAACCATTTGCACTTGCTTCATTTCACAAC:TTTCCGGCAAAT:

||||||||||||||||||||||||||||||| |||||| |||||

**CCAACCATTTGCACTTGCTTCATTTCACAAC:TTTCCGCCAAAT:AATTTGAAAAATCATGGTATTAATTTC**

|||||||||||| |||||||||||||||||||||||||||

:TTTCCGCCAAAT:AATTTGAAAAATCATGGTATTAATTTC

:chrV 34497+

PGSP4827 (*sae2 chrV:34,470-gRNA*)

Repetitive subtelomeric Y’ region:

AATTTCCTTCACTCTCCAACTTCTCTGCTCGAATCT:C:

|||||||||||||||||||||||||||||||||||| |

**AATTTCCTTCACTCTCCAACTTCTCTGCTCGAATCT:C:AGAAGGTTCTAAGATTAAATACGAGTTTCCGCCAAATA**

| ||||||||||||||||||||||||||||||||||||||

:C:AGAAGGTTCTAAGATTAAATACGAGTTTCCGCCAAATA

:chrV 34471+

PGSP4870 (*sae2 slx1*)

chrV 141668+:

TGTGAATTAGACAGCCTGTTCACCGGTAGCCTTTTGATTG:T:

|||||||||||||||||||||||||||||||||||||||| |

**TGTGAATTAGACAGCCTGTTCACCGGTAGCCTTTTGATTG:T:ACTGAACCGATTCAATTGAACAATAAGCACGACCTT**

| ||||||||||||||||||||||||||||||||||||

:T:ACTGAACCGATTCAATTGAACAATAAGCACGACCTT

:chrX 474022+

PGSP4878 (*sae2 rad52*)

chrV 151556+:

ATATTGATTACACCGTACTTCTTTTCAATGCGTAA:ACAAC:

||||||||||||||||||||||||||||||||||| |||||

**ATATTGATTACACCGTACTTCTTTTCAATGCGTAA:ACAAC:GATGCTGTAGACGCAGTAGTTGTCTTATGAGACGTGG**

||||| |||||||||||||||||||||||||||||||||||||

:ACGAC:GATGCTGTAGACGCAGTAGTTGTCTTATGAGACGTGG

:chrV 482740+

PGSP4881 (*sae2 rad52*)

chrII 232118+:

TATCCCATTATTTAATTCCAATG:ATCGAACATTATGTCTTTT:

||||||||||||||||||||||| |||||||| ||||| |||

**TATCCCATTATTTAATTCCAATG:ATCGAACAAAATGTCCTTT:GTACTGTAATGAATTAGCTTC**

||||||||||||||||||| |||||||||||||||||||||

:ATCGAACAAAATGTCCTTT:GTACTGTAATGAATTAGCTTC

:chrV 40241+

PGSP4883 (*sae2 rad52*)

chrV 60298+:

ACAAGCAATAATATAAGACAATTCGCCGGTAGTAT:TAT:

||||||||||||||||||||||||||||||||||| |||

**ACAAGCAATAATATAAGACAATTCGCCGGTAGTAT:TAT:TAATACTATTAAATTGCAATTATCATTT**

||| ||||||||||||||||||||||||||||

:TAT:TAATACTATTAAATTGCAATTATCATTT

:chrXVI 155535-

PGSP4884 (*sae2 rad52*)

chrV 76938+:

TCCACGGCATACTCATTGAGT:GCAGGAGGCTCTTT:

||||||||||||||||||||| ||||||||||||||

**TCCACGGCATACTCATTGAGT:GCAGGAGGCTCTTT:TTTGATCTTGAATATCATTTGAGTA**

|||||||||||||| |||||||||||||||||||||||||

:GCAGGAGATTCTTT:TTTGATCTTGAATATCATTTGAGTA

:chrV 436682+

PGSP4886 (*sae2 rad52*)

ChrVII 1003287-:

AGATCCCATCTAGGGGCTGGATGTATAATGCCTA:TGGTGTTG:

|||||||||||||||||||||||||||||||||| ||||||||

**AGATCCCATCTAGGGGCTGGATGTATAATGCCTA:TGGTGTTG:CAGGCTTTTTTGCATGGTTATTTATCTCAATC**

|||||||| ||||||||||||||||||||||||||||||||

:TGGTGTTG:CAGGCTTTTTTGCATGGTTATTTATCTCAATC

:ChrV 32099+ (in inserted CAN1 sequence)

PGSP4887 (*sae2 rad52*)

AGAATTTGTTATTGTGTTAATAAATAAAGTTAACATCCAGTTCTTTCAAGTTGGCTAAGC

||||||||||||||||||||||||||||||||||||||||||||||||||||||||||||

**AGAATTTGTTATTGTGTTAATAAATAAAGTTAACATCCAGTTCTTTCAAGTTGGCTAAGC**

|||

:AGC

:chrXVI 338919

GCCGTGGCGCAGTGGAAGCGCGCAGGGCTCATAACCCTGATGTCCTCGGATCGAAACCGA

||||||||||||||||||||||||||||||||||||||||||||||||||||||||||||

**GCCGTGGCGCAGTGGAAGCGCGCAGGGCTCATAACCCTGATGTCCTCGGATCGAAACCGA**

||||||||||||||||||||||||||||||||||||||||||||||||||||||||||||

GCCGTGGCGCAGTGGAAGCGCGCAGGGCTCATAACCCTGATGTCCTCGGATCGAAACCGA

chrV 100221+:

GCGGCGCTAA-TTT-TTCATTTCTTTTT:

|||||||||| ||| |||||||||||||

**GCGGCGCTAAATTTATTTTTTTCTTTTTACTCTTCATTTTTTTTCCAGGTCGGTGAT**

|||||||||||||||||||||||||||||||||||||||||||||||||||||||||

GCGGCGCTAAATTTATTTTTTTCTTTTTACTCTTCATTTTTTTTCCAGGTCGGTGAT

PGSP4890 (*sae2 rad52*)

chrV 127669+:

CTAATCCGGAACCTTTAGTAGTAATACTAGC:CTCAGAT:

||||||||||||||||||||||||||||||| |||||||

**CTAATCCGGAACCTTTAGTAGTAATACTAGC:CTCAGAT:ACCGTACAGCCACTGTTTATAATTGTTG**

||| ||| ||||||||||||||||||||||||||||

:CTC-GAT:ACCGTACAGCCACTGTTTATAATTGTTG

:chrIII 1517-

PGSP4900 (*yen1*)

chrXIV 709088-:

TAGACGGTGATCTTCTCCTACCCGTCCC:AGTCCTGG:

|||||||||||||||||||||||||||| ||||||||

**TAGACGGTGATCTTCTCCTACCCGTCCC:AGTCCTGG:ATACCGCCAGCCTCAGAGGTGGACAAGGAGGTGTCTT**

|||||||| |||||||||||||||||||||||||||||||||||||

:AGTCCTGG:ATACCGCCAGCCTCAGAGGTGGACAAGGAGGTGTCTT

:chrV 41236+

PGSP4924 (*sae2 sgs1 yku80*)

ChrXV 651717+:

TACGACTTCTAAGGTTATTTTGAATAGAATA:GTTAGCTTGAAG-GAC:

||||||||||||||||||||||||||||||| |||||||||||| |||

**TACGACTTCTAAGGTTATTTTGAATAGAATA:GTTAGCTTGAAGCGAC:TTTCTTTCTCTACTAAA**

|||||||||||||||| |||||||||||||||||

:GTTAGCTTGAAGCGAC:TTTCTTTCTCTACTAAA

:chrV 34343+

PGSP4974 (*sae2 chrV:25,817-1,749 gRNA*)

chrXIV 139457+:

:GGAGAAATCCAGGAGCCTGGGGGCCAGGCATCATCTCCAGTGATAAAAGTGAAGG:

|||||||||||||||||||||||||||| || || || | ||||||| |||||

**:GGAGAAATCCAGGAGCCTGGGGGCCAGGTATAATATCTAAGGATAAAAACGAAGG:GAGGTTCTTAGGTT**

||||||| ||||| |||||||| |||||||||||||||||||||||||||||||| ||||||||||||||

:GGAGAAACCCAGGTGCCTGGGGTCCAGGTATAATATCTAAGGATAAAAACGAAGG:GAGGTTCTTAGGTT

:chrV 32679-

PGSP5001 (*sae2 hs-del*)

ChrVI 220696-:

TCGTGCTTTCCACAAGTACAGATTG:AAG:

||||||||||||||||||||||||| |||

**TCGTGCTTTCCACAAGTACAGATTG:AAG:CTTCCATCCAAGCATCCGCTAAAACTCATGCAAG**

||| ||||||||||||||||||||||||||||||||||

:AAG:CTTCCATCCAAGCATCCGCTAAAACTCATGCAAG

:ChrV 29062+

PGSP5018 (*sae2 chrV:34,339-110 gRNA*)

ura3-52 Ty element:

TTTCGGTCAACCTGTTATCGTCAATGATCACAACCCTA:A:

|||||||||||||||||||||||||||||||||||||| |

**TTTCGGTCAACCTGTTATCGTCAATGATCACAACCCTA:A:GCTATTCAATGTCTGTATTCATCGATAAGC**

| ||||||||||||||||||||||||||||||

:A:GCTATTCAATGTCTGTATTCATCGATAAGC

:chrV 152468+

PGSP5022 (*wt chrV:34,470 gRNA*)

chrIII 295060-:

CCTTATGTCATTGTTTAACACAGTATAATGA:T:

||||||||||||||||||||||||||||||| |

**CCTTATGTCATTGTTTAACACAGTATAAAGA:T:TCAACATGTTATGCTTGTTAATAAGCTGCTCAAATTGATCA**

| |||||||||||||||||||||||||||||||||||||||||

:T:TCAACATGTTATGCTTGTTAATAAGCTGCTCAAATTGATCA

:chrV 36845+

PGSP5025 (*wt chrV:34,470 gRNA*)

Ty-related sequence:

CCTTAGAAGTAACCGAAGCACAGGCGC:TA:

||||||||||||||||||||||||||| ||

**CCTTAGAAGTAACCGAAGCACAGGCGC:TA:AATACGAGTTTCCGCCAAATAATTTGAAAAATCATGG**

|| |||||||||||||||||||||||||||||||||||||

:TA:AATACGAGTTTCCGCCAAATAATTTGAAAAATCATGG

:chrV 34487+

PGSP5026 (*wt chrV:34,470 gRNA*)

Ty-related sequence:

GGACTTCCTTAGAAGTAACCGAAGCACAGGCGCTA:CC:

||||||||||||||||||||||||||||||||||| ||

**GGACTTCCTTAGAAGTAACCGAAGCACAGGCGCTA:CC:GCCAAATAATTTGAAAAATCATGGTATTAATTTCAACA**

|| ||||||||||||||||||||||||||||||||||||||

:CC:GCCAAATAATTTGAAAAATCATGGTATTAATTTCAACA

:ChrV 34500+

PGSP5031 (*wt chrV:30,843 gRNA*)

Subtelomeric, e.g. chrV 5818-:

GACAAGAAATCCATCAATATAAACAAAAGATTGTCCAG:TTTCCGTCTTATC:

|||||||||||||||||||||||||||||||||||||| |||||| ||||||

**GACACGAAATCCATCAATATAAACAAAAGATTGTCCAG:TTTCCGCCTTATC:CGGCCTACAGAACCCAAA**

||||||||||||| ||||||||||||||||||

:TTTCCGCCTTATC:CGGCCTACAGAACCCAAA

:chrV L 34339-1140 (in can1::hisG)

PGSP5037 (*sae2 chrV:30,843 gRNA*)

Ty related sequence:

ACTCACAGCTTTTGAAAGGACATTTCTC:AGT TGCTCA:

|||||||||||||||||||||||||||| ||| ||||||

**ACTCACAGCTTTTGAAAGGACATTTCTC:GGT-TGCTCA:CTTGCCAGCAAGAGTCAAGTTA**

||| || || ||||||||||||||||||||||

:GGTATGTTCT:CTTGCCAGCAAGAGTCAAGTTA

:ChrV 30849+

PGSP5038 (*sae2 chrV:30,843 gRNA*)

Repetitive subtelomeric sequence:

TACTACATCAAAACGCATATTCCCTAGAAAAA:

||||||||||||||||||||||||||||||||

**TACTACATCAAAACGCATATTCCCTAGAAAAA:TT:CCCTGGTATGTTCTCTTGCCAGCAAGAGTCAAGTTAATGA**

||||||||||||||||||||||||||||||||||||||||

:CCCTGGTATGTTCTCTTGCCAGCAAGAGTCAAGTTAATGA

:chrV 30845+

PGSP5042 (*sae2 chrV:34,470 gRNA*)

Ty-related sequence:

GGGTACGGCCCATTCTGTGGTGAATGTGA::

|||||||||||||||||||||||||||||

**GGGTACGGCCCATTCTGTGGTGAATGTGA::CTAAGATTAAATACGAGTTTCCGCCAAATAATTTGAAAAATCATG**

**|||||||||||||||||||||||||||||||||||||||||||||**

::CTAAGATTAAATACGAGTTTCCGCCAAATAATTTGAAAAATCATG

:chrV 34480+

PGSP5044 (*sae2 chrV:34,470 gRNA*)

Ty-related sequence:

ACTATCGTCTATCAACTAATAGTTA:TAAATA:

||||||| ||||||||||||||||| ||||||

**CTCGACTATCGACTATCAACTAATAGTTA:TAAATA:CGAGTTTCCGCCAAATA**

|||||| |||||||||||||||||

:TAAATA:CGAGTTTCCGCCAAATA

:ChrV 34487+

PGSP5076 *(sae2 mus81 chrV:25,817-1,749 gRNA*)

chrIII 174064-:

AGAACCAGTTGTTTTGACAAGAAATGGTGAAG:G:

|||||||||||||||||||||||||||||||| |

**AGAACCAGTTGTTTTGACAAGAAATGGTGAAG:G:CCTGGGGTCCAGGTATAATATCTAAGGATAAAAACGAAGG**

| ||||||||||||||||||||||||||||||||||||||||

:G:CCTGGGGTCCAGGTATAATATCTAAGGATAAAAACGAAGG

:chrV 32665- (in inserted CAN1)

PGSP5077 *(sae2 mus81 chrV:25,817-1,749 gRNA*)

chrXV 130138+:

ATAGATGCCATAGACTACTTACATTCCAACGGTATTATTCA:

|||||||||||||||||||||||||||||||||||||||||

**ATAGATGCCATAGACTACTTACATTCCAACGGTATTATTCA::CCTGGGGTCCAGGTATAATATCTAAGGATAAAAAC**

|||||||||||||||||||||||||||||||||||

::CCTGGGGTCCAGGTATAATATCTAAGGATAAAAAC

:chrV 32664- (in inserted CAN1)

PGSP5079 *(sae2 mus81 chrV:25,817-1,749 gRNA*)

chrXV 1027898+:

CCCATTCAATTGCAGCAAGTTGTCATTTGAAGTTTCAT:TGCCT:

|||||||||||||||||||||||||||||||||||||| |||||

**CCCATTCAATTGCAGCAAGTTGTCATTTGAAGTTTCAT:TGCCT:GGGGTCCAGGTATAATATCTAAGGATAAAA**

||||| ||||||||||||||||||||||||||||||

:TGCCT:GGGGTCCAGGTATAATATCTAAGGATAAAA

:chrV 32666- (in inserted CAN1)

PGSP5081 *(sae2 mus81 chrV:25,817-1,749 gRNA*)

chrXII 459,512-, 468,649-:

GTGAAAAAGCAAAAGCAATAGTGCATTGTGATGTG::

|||||||||||||||||||||||||||||||||||

**GTGAAAAAGCAAAAGCAATAGTGCATTGTGATGTG::TGGGGTCCAGGTATAATATCTAAGGATAAAAACGAAGGG**

|||||||||||||||||||||||||||||||||||||||

::TGGGGTCCAGGTATAATATCTAAGGATAAAAACGAAGGG

:chrV 32,662- (in inserted CAN1)

PGSP5083 *(sae2 mus81 chrV:25,817-1,749 gRNA*)

chrVII 145,590-:

ATCGTCACCCCAATCCTTATTTCTTGCAGACCTT:TGCGCT:

|||||||||||||||||||||||||||||||||| ||||||

**ATCGTCACCCCAATCCTTATTTCTTGCAGACCTT:TGCGCT:GGGGTCCAGGTATAATATCTAAGGATAAAAACGAAGG**

||| || |||||||||||||||||||||||||||||||||||||

:TGC-CT:GGGGTCCAGGTATAATATCTAAGGATAAAAACGAAGG

:chrV 32,666- (in inserted CAN1)

PGSP5085 *(sae2 mus81 chrV:25,817-1,749 gRNA*)

chrIV 79,993-:

TATAAACTGAGTTCTCGCTGGTGAATACGGAG::

||||||||||||||||||||||||||||||||

**TATAAACTGAGTTCTCGCTGGTGAATACGGAG::GCCTGGGGTCCAGGTATAATATCTAAGGATAAAAACGAAG**

||||||||||||||||||||||||||||||||||||||||

::GCCTGGGGTCCAGGTATAATATCTAAGGATAAAAACGAAG

:chrV 32,665- (in inserted CAN1)

PGSP5086 *(sae2 exo1 chrV:25,817-1,749 gRNA*)

chrXIV 139490+ (in LYP1):

GTGATAAAAGTGAAGGCCGTTTTCTCGGATGGGTC:TCCTCCTTGATTAA:TGCTGCATTTACGTACCAAGGTACT

||||||||||||||||||||||||||||||||||| |||||||||||||| ||||| || || | |||||||||

**GTGATAAAAGTGAAGGCCGTTTTCTCGGATGGGTC:TCCTCTTTGATTAA:CGCTGCCTTCACATTTCAAGGTACT**

||||| |||||||||||||| |||||||||||||||||||||||||

TGGGTT:TCCTCTTTGATTAA:CGCTGCCTTCACATTTCAAGGTACT

:chrV 32605- (in inserted *CAN1*)
