# Supplementary material for: Mechanisms underlying genome instability mediated by formation of foldback inversions in Saccharomyces cerevisiae
Source: eLife. 2020 Aug 7;9:e58223. doi: 10.7554/eLife.58223 (PMC7467729; doi:10.7554/eLife.58223)
Supplement: Supplementary file 6. [file elife-58223-supp6.docx]

**Supplementary File 6.** **Interstitial deletion junction sequences**

PGSP752 [bzg110] (*pol32*)

ChrV 8879+:

ATGAACAATAATTAACACGAGAATTTAAACCATACTCGGCCG::

||||||||||||||||||||||||||||||||||||||||||

**ATGAACAATAATTAACACGAGAATTTAAACCATACTCGGCCG::AATCCAGGTGCATTTTAAGAAACTTATCTGCTGGG**

|||||||||||||||||||||||||||||||||||

::AATCCAGGTGCATTTTAAGAAACTTATCTGCTGGG

:ChrV 27385+

PGSP939 (*sgs1*)

ChrV 23,935+:

CAAGGAAAGGAACACGACAAATGATTGTC:

|||||||||||||||||||||||||||||

**CAAGGAAAGGAACACGACAAATGATTGTC:CCAGACCCGTTTGATCTCAAGACTTTCACCGCAACAACG**

|||||||||||||||||||||||||||||||||||||||

:CCAGACCCGTTTGATCTCAAGACTTTCACCGCAACAACG

:ChrV 40785+

PGSP3607 (*yku80*)

ChrV 13549+:

TTACCAATATGGGATCAATAACTTTACTTTTG:AAGATCTT:

|||||||||||||||||||||||||||||||| |||| |||

**TTACCAATATGGGATCAATAACTTTACTTTTG:AAGAGCTT:AGAACGGAGTTTGAATTGACAAATTTT**

|||||||| |||||||||||||||||||||||||||

:AAGAGCTT:AGAACGGAGTTTGAATTGACAAATTTT

:ChrV 31202- (CAN1 insertion)

PGSP3608 (*yku80*)

ChrV 5473+:

GGTTCGTCTTCAACAAACGTCAG:GAA-CTTTTTTAC-A-TTTTTCCC:

||||||||||||||||||||||| ||| ||||||||| | ||||||||

**GGTTCGTCTTCAACAAACGTCAG:GAA-CTTTTTTCCCACTTTTTCCC:AGTGAAATAGTTTCTTATTGGCTTTT**

||| |||||||||||||||||||| ||||||||||||||||||||||||||

:GAAGCTTTTTTCCCACTTTTTCCC:AGTGAAATAGTTTCTTATTGGCTTTT

:ChrV 27270+

PGSP4575 [bzg109] (*rrm3*)

ChrV 18499+:

GTTTATTTCTTGTAAGAGTCGAAACTAAACCT:TAGTTG:

|||||||||||||||||||||||||||||||| ||||||

**GTTTATTTCTTGTAAGAGTCGAAACTAAACCT:TAGTTG:TTTAATTGTAAAAACAGTGTAGATTTCAATTTTTTAAT**

||| || ||||||||||||||||||||||||||||||||||||||

:TAG-TG:TTTAATTGTAAAAACAGTGTAGATTTCAATTTTTTAAT

:ChrV 37590+

PGSP4578 [bzg130] (*rad10*)

ChrV 24041+:

TAAGGAAGGTGCTTTTGCAACACTTTGCCCCTTCGTTGTG:AAG:

|||||||||||||||||||||||||||||||||||||||| |||

**TAAGGAAGGTGCTTTTGCAACACTTTGCCCCTTCGTTGTG:AAG:TAGGCTCTGTCATCGCTCAACGTGGAAGCTCCG**

||| |||||||||||||||||||||||||||||||||

:AAG:TAGGCTCTGTCATCGCTCAACGTGGAAGCTCCG

:ChrV 40500+

PGSP4587 [bzg139] (*rad10*) – *CEN5* deletion

ChrV 151043+:

ACAGTGAACTTTTACTTGTTTCTTTTGTTTAACCACTAGTATG::

|||||||||||||||||||||||||||||||||||||||||||

**ACAGGGAACTTTTACTTGTTTCTTTTGTTTAACCACTAGTATG::CTATTAAGTTGTAAAACAATCGCGAAAAAAAAAA**

||||||||||||||||||||||||||||||||||

::CTATTAAGTTGTAAAACAATCGCGAAAAAAAAAA

:ChrV 153003+

PGSP4609 (*sae2 pol32*)

ChrV 15946+:

AATCTACCCAAAATGTCTCGAGCATCTTGATAATTACA:G:

|||||||||||||||||||||||||||||||||||||| |

**AATCTACCCAAAATGTCTCGAGCATCTTGATAATTACA:G:CAACTTGAAACCAGAAAGACCATCAAAGGTTTCATTT**

| |||||||||||||||||||||||||||||||||||||

:G:CAACTTGAAACCAGAAAGACCATCAAAGGTTTCATTT

:ChrV 36019+

PGSP4853 (*sae2 yen1*), deletion of *CEN5*

ChrV 153677+:

GAAAGATGACCTGAACTGATAAAAATTGTGGG:T:

|||||||||||||||||||||||||||||||| |

**GAAAGATGACCTGAACTGATAAAAATTGTGGG:T:GCGCTTATACGATCAAGCAATGTAAACAGTACAAA**

| |||||||||||||||||||||||||||||||||||

:T:GCGCTTATACGATCAAGCAATGTAAACAGTACAAA

:ChrV 153642+

PGSP4905 (*slx1*)

ChrV 25256+:

ATACCTGACAACGTAGCAGCGAGCCTTAGTTCATCTGCTCA:GG:

||||||||||||||||||||||||||||||||||||||||| ||

**ATACCTGACAACGTAGCAGCGAGCCTTAGTTCATCTGCTCA:GG:CTTTCTCATGCGTTCATGCACCACT**

|| |||||||||||||||||||||||||

:GG:CTTTCTCATGCGTTCATGCACCACT

:ChrV 34339-80

**GGAAGATCTGAATTCTTGAAGACGAAAGGGCCTCGTGATACGCCGGGGGATCC:CTGGGTTAGCTTGAAG**

||||||||||||||||||||||||||||||||||||||||||||||||||||| ||||||||||||||||

GGAAGATCTGAATTCTTGAAGACGAAAGGGCCTCGTGATACGCCGGGGGATCC:CTGGGTTAGCTTGAAG

:ChrV 34339+

PGSP4908 (*slx1*)

ChrV 363+:

TATCAAATCTACCGTCTGGAACATCATCGCTATCCAGCTCTTT:GTGA-ACCGCTACC:

||||||||||||||||||||||||||||||||||||||||||| |||| ||||| |||

**TATCAAATCTACAGTCTGGAACATCATCGCTATCCAGCTCTTT:GTGACACCGCGACC:GGCATCATCATCGTAGAGA**

|||||||||||||| |||||||||||||||||||

:GTGACACCGCGACC:GGCATCATCATCGTAGAGA

:ChrV 40995+

PGSP4909 (*slx1*)

ChrV 528+:

CGAAATGTTTTATTGTAGAACAGCCCTAT:CAGCATCGAGAGG:

||||||||||||||||||||||||||||| ||||| || ||||

**CGAAATGTTTTATTGTAGAACAGCCCTAT:CAGCAACGTGAGG:GGAAGCCATTGAGGTACCCGATAAAGTG**

||||||||||||| ||||||||||||||||||||||||||||

:CAGCAACGTGAGG:GGAAGCCATTGAGGTACCCGATAAAGTG

:ChrV 40375+

PGSP4914 (*slx1*)

ChrV 23966+:

CAAATGATTGTCAATAGTATGTTTCTCTGAAGGCACA:GGTTAC:

||||||||||||||||||||||||||||||||||||| ||||||

**CAAATGATTGTCAATAGTATGTTTCTCTGAAGGCACA:GGTTAC:AGACCATTACAATCACCGCGCACAGTTGCTGTA**

|||||| |||||||||||||||||||||||||||||||||

:GGTTAC:AGACCATTACAATCACCGCGCACAGTTGCTGTA

:ChrV 27113+

PGSP4915 (*slx1*)

ChrV 3634+:

ACGTCTTCCGCATTCCGAGTACTAGAG:AATGAAC:

||||||||||||||||||||||||||| |||||||

**ACGTCTTCCGCATTCCGAGTACTAGAG:AATGAAC:CCAGTTGCGGCTTGTTCTTAATGTTTTTAGCCTTCG**

||||||| ||||||||||||||||||||||||||||||||||||

:AATGAAC:CCAGTTGCGGCTTGTTCTTAATGTTTTTAGCCTTCG

:ChrV 38081+

PGSP4917 (*slx1*)

ChrV 528+:

GAAATGTTTTATTGTAGAACAGCCCTAT:CAGCATCGAGAGG:

|||||||||||||||||||||||||||| |||||||||||||

GAAATGTTTTATTGTAGAACAGCCCTAT:CAGCAACGTGAGG:GGAAGCCATTGAGGTACCCGATAAAGTGGCGGTG

||||||||||||| ||||||||||||||||||||||||||||||||||

:CAGCAACGTGAGG:GGAAGCCATTGAGGTACCCGATAAAGTGGCGGTG

:ChrV 40375+

PGSP4967 (*sae2* chrV:35,709 gRNA – deletion of *CEN5*)

ChrV 151941+:

CCGAGATATGTTTTATTTAAGAACTATGAATCTGTAAAT:

|||||||||||||||||||||||||||||||||||||||

**CCGAGATATGTTTTATTTAAGAACTATGAATCTGTAAAT:TT:GTTGTTAGCTATAAGGAGATTTTTAGCTACGAACAT**

||||||||||||||||||||||||||||||||||||

:GTTGTTAGCTATAAGGAGATTTTTAGCTACGAACAT

:ChrV 152305+

PGSP4998 (*sae2 hs-del*)

ChrV 3699+:

CATTGAATCCAGCGCTCGGAAGCTCGTTCCTGC:AATAAA:

||||||||||||||||||||||||||||||||| ||||||

**CATTGAATCCAGCGCTCGGAAGCTCGTTCCTGC:AATAAA:TGATAAGTTACTTGAAAAAAGAAATCACAATCAT**

||| || ||||||||||||||||||||||||||||||||||

:AAT-AA:TGATAAGTTACTTGAAAAAAGAAATCACAATCAT

:ChrV 35940+

PGSP4999 (*sae2 hs-del*)

Chr V 1726+:

TACTTTCAGTGGTAGTAGCATTAGTGTTGGAGTTGG:

||||||||||||||||||||||||||||||||||||

**TACTTTCAGTGGTAGTAGCATTAGTGTTGGAGTTGG:GCTTGCTTCTGTATCTATACTCAACGTTACTTTCTGG**

|||||||||||||||||||||||||||||||||||||

:GCTTGCTTCTGTATCTATACTCAACGTTACTTTCTGG

:ChrV 39606+

PGSP5024 (*wt chrV:34,470 gRNA*)

ChrV 24295+:

AATAACGGTGTCCGAGTCCTTTCCCTC:TCATGTTTTTT-TTTCAACTACA:

||||||||||||||||||||||||||| ||||| | || ||||||| |||

**AATAACGGTGTCCGAGTCCTTTCCCTC:TCATGGTATTAATTTCAAC-ACA:TTTAAAAACTACATCATAACGAA**

||||||||||||||||||| ||| |||||||||||||||||||||||

:TCATGGTATTAATTTCAAC-ACA:TTTAAAAACTACATCATACCGAA

:ChrV 34520+

PGSP5040 (*sae2 chrV:34,470 gRNA*)

chrV 6656+:

CTTCGCATACCATTGTCATAGAATCTCACACTGACGCAT:GATTAAA-ACGA:

||||||||||||||||||||||||||||||||||||||| ||||||| ||||

**CTTCGCATACCATTGTCATAGAATCTCACACTGACGCAT:GATTAAATACGA:GTTTCCGCCAAATAATTTGAAAAAT**

|||||||||||| |||||||||||||||||||||||||

:GATTAAATACGA:GTTTCCGCCAAATAATTTGAAAAAT

:chrV 34484+

PGSP5065 *(exo1 yku80*)

chrV 16,194+:

TTTCCAGCGGATGACACCACT:TGCCACA-GTTG:

||||||||||||||||||||| ||||||| ||||

**TTTCCAGCGGATGACACCACT:TGCCACAAGTTG:ATCACTTTTAAGTATGGTACATATAGG**

|||||||||||| |||||||||||||||||||||||||||

:TGCCACAAGTTG:ATCACTTTTAAGTATGGTACATATAGG

:chrV 34,575+

PGSP5074 *(sae2 mus81 chrV:25,817-1,749 gRNA*)

chrV 116323- (in inserted URA3):

CAAGATATCCACATGTGTTTTTAGTAAACAAATTT:TGGG:

||||||||||||||||||||||||||||||||||| ||||

**CAAGATATCCACATGTGTTTTTAGTAAACAAATTT:TGGG:GTCCAGGTATAATATCTAAGGATAAAAACGAAGGGAGG**

|||| ||||||||||||||||||||||||||||||||||||||

:TGGG:GTCCAGGTATAATATCTAAGGATAAAAACGAAGGGAGG

:chrV 32662- (in inserted CAN1)

PGSP5075 *(sae2 mus81 chrV:25,817-1,749 gRNA*)

chrV 22578+:

GGACTCTCCATTGTGCAGTGTTATCGTATTTTCTCGT:A:

||||||||||||||||||||||||||||||||||||| |

**GGACTCTCCATTGTGCAGTGTTATCGTATTTTCTCGT:A:AGAACTGTTAAAGACCCAGTACGAAAATTTTTCCATAA**

| ||||||||||||||||||||||||||||||||||||||

:A:AGAACTGTTAAAGACCCAGTACGAAAATTTTTCCATAA

:chrV 27619+

PGSP5090 *(sae2 exo1 chrV:25,817-1,749 gRNA*)

chrV 116148- (in inserted URA3):

AGCTTTCGACATGATTTATCTTCGTTTCCTG::

|||||||||||||||||||||||||||||||

**AGCTTTCGACATGATTTATCTTCGTTTCCTG::GGGGTCCAGGTATAATATCTAAGGATAAAAACGAAGGGAGGCTCT**

|||||||||||||||||||||||||||||||||||||||||||||

CCT-::GGGGTCCAGGTATAATATCTAAGGATAAAAACGAAGGGAGGTTCT

:chrV 32661- (in inserted CAN1)
