# Supplementary material for: Mechanisms underlying genome instability mediated by formation of foldback inversions in Saccharomyces cerevisiae
Source: eLife. 2020 Aug 7;9:e58223. doi: 10.7554/eLife.58223 (PMC7467729; doi:10.7554/eLife.58223)
Supplement: Supplementary file 7. [file elife-58223-supp7.docx]

**Supplementary File 7. *S. cerevisiae* strains.**

| ***Strain*** | ***Genotype*** | ***Reference*** |
| --- | --- | --- |
| RDKY6677 | **MATa** *leu2∆1 his3∆200 trp1∆63 lys2∆Bgl hom3-10 ade2::hisG ade8 ura3-52 can1::hisG iYEL072::hphNT1* *yel068c::CAN1/URA3* | (Putnam et al. 2009) |
| RDKY6729 | RDKY6677 *exo1Δ::HIS3* | (Putnam et al. 2009) |
| RDKY8032 | RDKY6677 *exo1Δ::TRP1 sgs1Δ::HIS3* | (Putnam et al. 2014) |
| RDKY9771 | RDKY6677 *exo1Δ::HIS3 yku80Δ::kanMX4* | This study |
| RDKY6686 | RDKY6677 *mre11Δ::HIS3* | (Putnam et al. 2009) |
| HZY2771 | RDKY6677 *cir0* *mre11-H125N::TRP1* | (Liang et al. 2018) |
| RDKY6731 | RDKY6677 *mus81Δ::HIS3* | (Putnam et al. 2009) |
| RDKY6894 | RDKY6677 *pif1Δ::HIS3* | (Putnam et al. 2014) |
| RDKY9773 | RDKY6677 *pif1Δ::HIS3 yku80Δ::kanMX4* | This study |
| RDKY6703 | RDKY6677 *pol32Δ::TRP1* | (Putnam et al. 2009) |
| RDKY6734 | RDKY6677 *rad10Δ::HIS3* | (Putnam et al. 2009) |
| RDKY6691 | RDKY6677 *rad52Δ::HIS3* | (Putnam et al. 2009) |
| RDKY6735 | RDKY6677 *rrm3Δ::TRP1* | (Putnam et al. 2009) |
| RDKY6687 | RDKY6677 *sgs1Δ::HIS3* | (Putnam et al. 2009) |
| RDKY9445 | RDKY6677 *sgs1Δ::HIS3 yku80Δ::kanMX4* | This study |
| RDKY6738 | RDKY6677 *slx1Δ::kanMX4* | (Putnam et al. 2009) |
| RDKY6761 | RDKY6677 *tel1Δ::HIS3* | (Putnam et al. 2009) |
| RDKY9506 | RDKY6677 *yen1Δ::HIS3* | This study |
| RDKY8006 | RDKY6677 *yku80Δ::HIS3* | (Putnam et al. 2014) |
| RDKY6737 | RDKY6677 *sae2Δ::TRP1* | (Putnam et al. 2009) |
| RDKY9734 | RDKY6677 *sae2Δ::TRP1 hotspotΔ* | This study |
| RDKY9472 | RDKY6677 *sae2-S267A* | This study |
| RDKY9496 | RDKY6677 *sae2-MT9* | This study |
| RDKY8020 | RDKY6677 *sae2Δ::TRP1 exo1Δ::HIS3* | (Putnam et al. 2014) |
| RDKY9777 | RDKY6677 *sae2Δ::TRP1 exo1Δ::HIS3 yku80Δ::kanMX4* | This study |
| RDKY9392 | RDKY6677 *sae2Δ::kanMX4 mus81Δ::HIS3* | This study |
| RDKY9447 | RDKY6677 *sae2Δ::TRP1 pif1Δ::HIS3* | This study |
| RDKY9779 | RDKY6677 *sae2Δ::TRP1 pif1Δ::HIS3 yku80Δ::kanMX4* | This study |
| RDKY9390 | RDKY6677 *sae2Δ::kanMX4 pol32Δ::HIS3* | This study |
| RDKY9123 | RDKY6677 *sae2Δ::TRP1 rad10Δ::HIS3* | This study |
| RDKY9504 | RDKY6677 *sae2Δ::TRP1 rad52Δ::HIS3* | This study |
| RDKY9125 | RDKY6677 *sae2Δ::TRP1 rrm3Δ::HIS3* | This study |
| RDKY9775 | RDKY6677 *sae2Δ::TRP1 sgs1Δ::HIS3 yku80Δ::kanMX4* | This study |
| RDKY9502 | RDKY6677 *sae2Δ::TRP1 slx1Δ::HIS3* | This study |
| RDKY8018 | RDKY6677 *sae2Δ::TRP1 tel1Δ::HIS3* | (Putnam et al. 2014) |
| RDKY9500 | RDKY6677 *sae2Δ::TRP1 yen1Δ::HIS3* | This study |
| RDKY9443 | RDKY6677 *sae2Δ::TRP1 yku80Δ::kanMX4* | This study |
